# Enantioselective access to chiral aliphatic amines and alcohols via Ni-catalyzed hydroalkylations

**DOI:** 10.1038/s41467-021-22983-7

**Published:** 2021-05-13

**Authors:** Shan Wang, Jian-Xin Zhang, Tian-Yi Zhang, Huan Meng, Bi-Hong Chen, Wei Shu

**Affiliations:** Shenzhen Grubbs Institute and Department of Chemistry, Guangdong Provincial Key Laboratory of Catalysis, Southern University of Science and Technology, Shenzhen, 518055 China

**Keywords:** Asymmetric catalysis, Stereochemistry

## Abstract

Chiral aliphatic amine and alcohol derivatives are ubiquitous in pharmaceuticals, pesticides, natural products and fine chemicals, yet difficult to access due to the challenge to differentiate between the spatially and electronically similar alkyl groups. Herein, we report a nickel-catalyzed enantioselective hydroalkylation of acyl enamines and enol esters with alkyl halides to afford enantioenriched α-branched aliphatic acyl amines and esters in good yields with excellent levels of enantioselectivity. The operationally simple protocol provides a straightforward access to chiral secondary alkyl-substituted amine and secondary alkyl-substituted alcohol derivatives from simple starting materials with great functional group tolerance.

## Abstract (web summary)

Chiral aliphatic amine and alcohol derivatives are difficult to access due to the challenge to differentiate between spatially and electronically similar alkyl groups. Here the authors show a nickel-catalyzed enantioselective hydroalkylation of acyl enamines and enol esters with alkyl halides to afford enantioenriched α-branched aliphatic acyl amines and esters in good yields with excellent enantioselectivity.

## Introduction

**Fig. 1:**
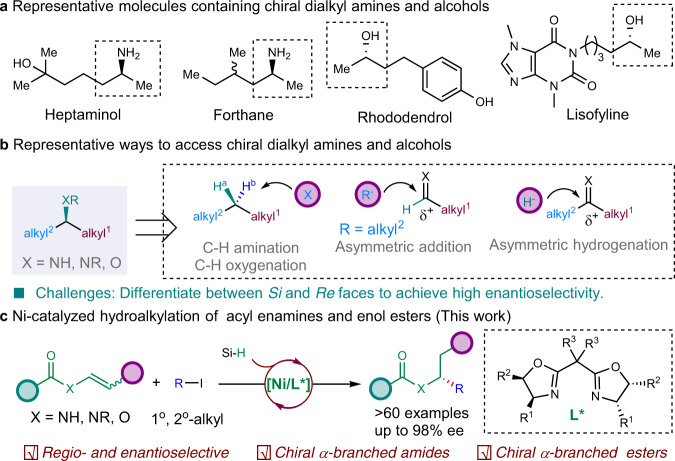
Impetus for the development of the reaction. **a** Representative molecules containing chiral secondary alkyl-substituted amines and alcohols. **b** Representative ways to access chiral secondary alkyl-substituted amines and alcohols. **c** Ni-catalyzed hydroalkylation of acyl enamines and enol esters.

Chiral aliphatic amines and alcohols are widespread substructures in pharmaceutical molecules, natural products and organic materials, and serve as common chiral building blocks for other functional groups and value-added molecule synthesis^1–3^. Additionally, over half of small-molecule drugs are the derivatives of chiral aliphatic amines and alcohols among the top 200 best-selling drugs (Fig. 1a)^4^. Thus, the enantioselective synthesis of pure aliphatic amines and alcohols has been recognized as a long-term interest in chemistry community. Over the past decades, significant progress has been made in this field enabled by enantioselective C–H amination/oxygenation^5–8^, addition of alkyl organometallic reagents to imines or aldehydes^9–13^, and hydrogenation of imines, enamines, ketones, or enol esters^14–19^. However, chiral catalysts have difficulty in identifying different faces of prochiral centers bearing two alkyl groups with similar steric and electronic properties^20^. Thus, these methods are typically applied to build chiral aliphatic amines and alcohols with the stereogenic center adjacent to aryl or carbonyl groups (Fig. 1b)^14,21–24^. To control the enantioselectivity of asymmetric reactions for regular secondary alkyl-substituted amines and alcohols still remains a formidable challenge. In 2020, Zhou group reported a breakthrough in Ir-catalyzed asymmetric hydrogenation of dialkyl ketones to afford chiral aliphatic alcohols with good enantioselectivity enabled by a rationally designed bulky PNP ligand^25^. Buchwald developed a seminal work on Cu–H-catalyzed hydroamination of internal alkenes to achieve chiral dialkyl amines^26,27^. In 2016, Fu group reported a pioneer work on Ni–H-catalyzed racemic hydrofunctionalizations of alkenes with aryl or alkyl halides^28^, which have become a promising alternative for traditional asymmetric C–C cross-coupling reaction to construct saturated stereogenic carbon centers^28–35^. The use of readily available and bench-stable alkenes as a masked nucleophile in the presence of silane circumvents the use of stoichiometric and often sensitive organometallic reagents, which usually require time-consuming preformation^36,37^. The abundance of alkene as well as the mild conditions significantly enhanced the scope and functional group tolerance of this strategy^38–41^. Fu group reported the seminal work on the anti-Markovnikov hydroalkylation of alkenes with activated secondary alkyl halides to build a stereogenic center originating from alkyl halides^42–45^. The use of unactivated alkyl halides to build stereogenic center originating from alkenes remains elusive due to the reversible Ni–H insertion onto alkenes and the propensity of chain-walking^46,47^. Recently, our group developed the Ni–H-catalyzed hydroalkylation of acrylates via anti-Markovnikov hydrometalation, giving the enantioenriched α-tertiary amides by forging a stereogenic center originating from acrylates^48^. In 2021, Hu group reported a hydroalkylation of vinyl boronates to give chiral secondary alkyl boronates enabled by the anchoring effect of boron^49^. These examples showcased the feasibility of building a stereogenic carbon center originating from alkenes via Ni-catalyzed hydroarylation^36–38^ and hydroalkylation^48–51^ of alkenes.

As part of our continuous interest in the enantioselective hydrofunctionalizations of alkenes, we envisioned the use of alkene adjacent to nitrogen or oxygen to undergo enantioselective hydroalkylation would furnish enantioenriched secondary aliphatic amine and alcohol derivatives (Fig. 1c). Here, we report the Ni–H-catalyzed regio- and enantioselective hydroalkylation of acyl enamines and enol esters with alkyl iodides to forge a stereogenic carbon center next to nitrogen or oxygen originating from alkenes in high enantioselectivity, providing a unified protocol for rapid access to chiral secondary alkyl-substituted amine and alcohol derivatives which are difficult to access otherwise^50–52^.

## Results

### Reaction optimization

To test the feasibility of the reaction, we set out to identify the reaction parameters using acyl enamine **1a** with 1-iodo-3-phenylpropane **2a** as substrate in the presence of silane. (Table 1 and Tables S1–13; for more details on the condition optimization, please see the Supplementary information). First, a wide range of chiral ligands were tested for this reaction using NiBr_2_.glyme (10 mol%) as the nickel catalyst precursor, trimethoxysilane (TMS) as hydride source, and potassium phosphate monohydrate as base in diethyl ether at room temperature (Table 1, entries 1–9 and Table S1). When pyridine-oxazolidine ligand (**L1** or **L2**) was used, the desired hydroalkylation product **3a** was obtained in 54% and 29% yields with low enantiomeric excesses (2% and 15%), respectively (Table 1, entries 1 and 2). Ph-Box ligands (**L3–L6**) could catalyze the reaction, giving **3a** in low yields with low enantioselectivities (Table 1, entries 3–6). Increasing the steric hindrance at α-position to oxygen increased the enantioselectivity of **3a** to 50% *ee* (Table 1, entries 5 and 6). Modifying the methyl group on **L4** to bulkier groups significantly improved the enantioselectivity of **3a** (Table 1, entries 7–9). The use of **L9** delivered **3a** in 20% yield with 90% *ee*. Using diethoxymethylsilane (DEMS) as hydride source slightly increased the enantioselectivity of **3a** to 94% (Table 1, entry 11). Next, the solvent for the reaction was evaluated. The use of *N*,*N*-dimethylacetamide (DMA) or *N*,*N*-dimethylformaldehyde (DMF) dramatically increased the efficiency of the reaction, delivering **3a** in up to 99% yield with diminished enantiomeric excess (Table 1, entries 12 and 13). The mixing of ether with DMA or DMF could increase the enantioselectivity of **3a** without erasing the efficiency of the reaction (Table 1, entries 14 and 15). Further optimization of the nickel precursor and reaction temperature improved the yield and enantioselectivity of **3a** (Table 1, entries 16–18). The use of Ni(COD)_2_ (10 mol%), **L9** (12 mol%), dimethoxymethylsilane (DMMS) (3 equiv.) in Et_2_O and DMF (3:1) gave **3a** in 93% yield with 92% *ee* (Table 1, entry 19).

**Table 1:** Condition evaluation of the reaction.


Entry	Ni cat.	L^*^	Si-H	Solvent	Yield (*ee*)^a^
1	NiBr_2_.glyme	**L1**	TMS	Et_2_O	54% (2%)
2	NiBr_2_.glyme	**L2**	TMS	Et_2_O	29% (15%)
3	NiBr_2_.glyme	**L3**	TMS	Et_2_O	32% (19%)
4	NiBr_2_.glyme	**L4**	TMS	Et_2_O	56% (13%)
5	NiBr_2_.glyme	**L5**	TMS	Et_2_O	29% (50%)
6	NiBr_2_.glyme	**L6**	TMS	Et_2_O	24% (16%)
7	NiBr_2_.glyme	**L7**	TMS	Et_2_O	62% (82%)
8	NiBr_2_.glyme	**L8**	TMS	Et_2_O	51% (85%)
9	NiBr_2_.glyme	**L9**	TMS	Et_2_O	20% (90%)
10	NiBr_2_.glyme	**L9**	TES	Et_2_O	14% (94%)
11	NiBr_2_.glyme	**L9**	DEMS	Et_2_O	24% (94%)
12	NiBr_2_.glyme	**L9**	DEMS	DMA	99% (73%)
13	NiBr_2_.glyme	**L9**	DEMS	DMF	56% (58%)
14	NiBr_2_.glyme	**L9**	DEMS	Et_2_O^b^	98% (77%)
15	NiBr_2_.glyme	**L9**	DEMS	Et_2_O^c^	99% (84%)
16	Ni(COD)_2_	**L9**	DEMS	Et_2_O^c^	99% (88%)
17^d^	Ni(COD)_2_	**L9**	DEMS	Et_2_O^c^	94% (92%)
18^d,e^	Ni(COD)_2_	**L9**	DEMS	Et_2_O^c^	99% (92%)
19^d,e^	Ni(COD)_2_	**L9**	DMMS	Et_2_O^c^	93%^f^ (92%)

^a^The reaction was conducted using **1a** (0.1 mmol) and **2a** (0.2 mmol) in 1 mL of solvent under indicated conditions for 12 h unless otherwise stated. Yield was determined by GC using *n*-dodecane as internal standard. The enantiomeric excess was determined by HPLC using a chiral stationary phase. L* = chiral ligand. TMS = trimethoxysilane. TES = triethoxysilane. DEMS = diethoxymethylsilane. DMMS = dimethoxymethylsilane. ^b^Et_2_O/DMA = 3:1. DMA = *N*,*N*-dimethylacetamide. ^c^Et_2_O/DMF = 3:1. Et_2_O = diethyl ether. DMF = *N*,*N*-dimethylformaldehyde. ^d^The reaction was run at 0 °C. ^e^The reaction was run for 24 h. ^f^Isolated yield after flash chromatography.

### Substrate scope of dialkyl amides

**Fig. 2:**
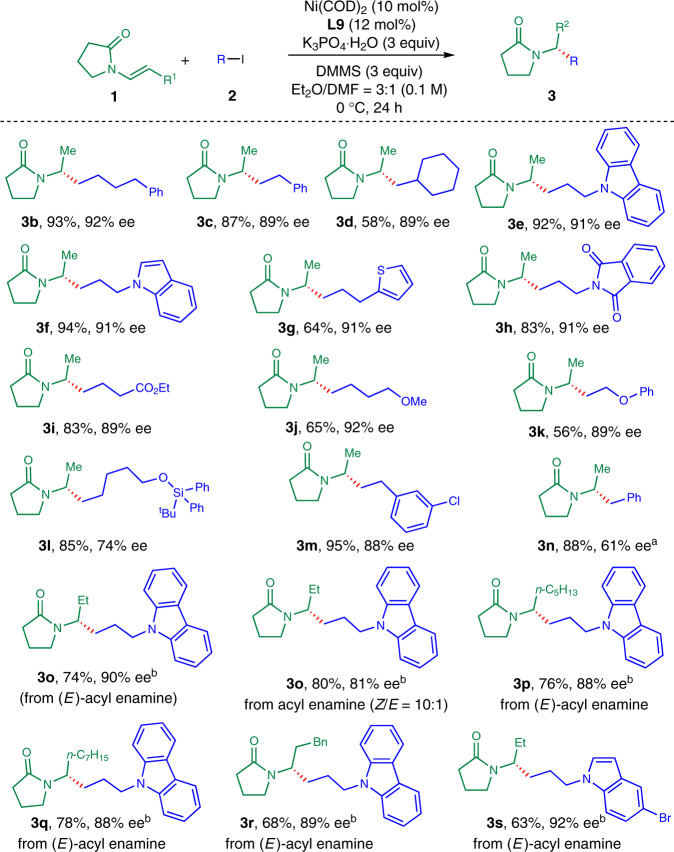
Scope for alkyl iodides of tertiary acyl enamines and internal acyl enamines. For reaction conditions, see Table 1, entry 19 unless otherwise stated. ^a^Benzyl bromide was used. ^b^The reaction was conducted using **1** (0.2 mmol), **2** (0.6 mmol) at 45 °C for 16 h.

With the optimized conditions in hand, we turned to evaluate the scope of this reaction. First, we tested different alkyl iodides with tertiary acyl enamine **1a** (Fig. 2). Then, 4-phenylbutyliodide was converted to chiral amide **3b** in 93% yield with 92% *ee*. 2-Phenyl-1-iodoethane and α-branched alkyl iodides could be transformed into corresponding amine derivatives (**3c** and **3d**) in 87% and 58% yields with 89% *ee*. Heterocyclic compounds, such as carbazoles, indoles, and thiophenes, worked well in the reaction, furnishing the regio- and enantioselective hydroalkylation products (**3e**–**3g**) in 64–94% yields with 91% *ee*. Other functional groups, such as amides, esters, ethers were also compatible under the reaction conditions, delivering the desired chiral amine derivatives (**3h**–**3k**) in 56–83% yields with 89–92% *ee*. Moreover, silylethers and arylchlorides were tolerated in the reaction, giving the desired products (**3l** and **3m**) in 85% and 95% yields with 74% and 88% *ee*, leaving chemical handles for further elaboration. Benzyl bromide was successfully converted to corresponding amide **3n** in 88% yield with moderate enantiomeric excess. Second, internal acyl enamines were examined. Internal acyl enamines with diverse substituents could be converted to corresponding hydroalkylated products in good yields with excellent enantioselectivities. Acyl (*E*)-1-propenamine reacted to give corresponding dialkyl amide **3o** in 74% yield with 90% *ee*. Alternatively, acyl (*Z*)-1-propenamine gave **3o** in 80% yield with 81% *ee* under the same conditions. Longer alkyl chain- and benzyl-substituted internal acyl enamines were all good substrates for this reaction, affording corresponding amine derivatives (**3p**–**3r**) in 68–78% yields with 88–89% *ee*. Bromoindole containing alkyl iodide could be coupled with internal acyl enamine to deliver **3s** in 63% yield with 92% *ee*.

**Fig. 3:**
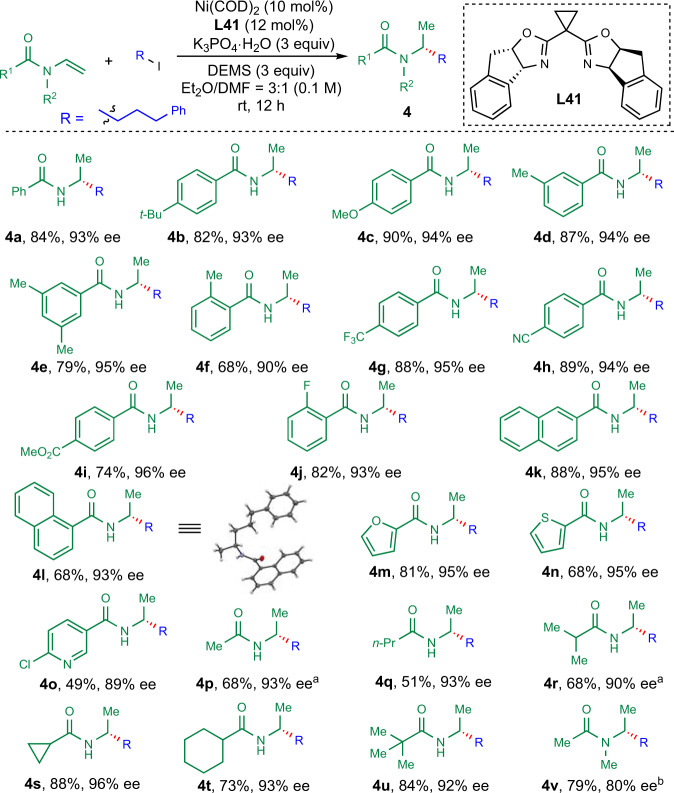
Scope for secondary acyl enamines. The reaction was run on 0.2 mmol of acyl enamine and 0.4 mmol of alkyl iodide using DEMS (0.6 mmol) under indicated conditions unless otherwise stated. ^a^DMMS was used instead of DEMS. ^b^**L4** was used as the ligand.

Next, the scope of secondary acyl enamines was tested (Fig. 3). A wide range of secondary acyl enamines were well-tolerated in this reaction, forming a myriad of enantioenriched amides in good efficiency with excellent levels of enantioselectivity in the presence of **L41**. Various aromatic amides were good substrates for this reaction (**4a**–**4o**). Electron-donating substituted aromatic acyl enamines could be converted to corresponding hydroalkylated products (**4a**–**4f**) in 68–90% yields with 90–95% *ee*. Electron-withdrawing substituents, such as trifluoromethyl, cyano, ester, fluoride, were well-tolerated under the reaction conditions, giving the desired products (**4g**–**4j**) in 74–89% yields with 93–96% *ee*. Fused aromatic and heteroaromatic acyl enamines, including naphthalene, furan, thiophene, and pyridine, were transformed into corresponding chiral amides (**4k**–**4o**) in 49–88% yields with 89–95% *ee*. The structure and absolute configuration of the product was determined by the X-ray diffraction analysis of **4l**. Aliphatic acyl enamines were also tested (**4p**–**4v**). Linear and α-branched aliphatic acyl enamines with acidic α-proton, such as methyl, *n*-propyl, isopropyl, cyclopropyl, cyclohexyl, were all good substrates for this hydroalkylation reaction, affording corresponding chiral amides (**4p**–**4t**) in 51–88% yields with 90–96% *ee*. α-Tertiary alkyl acyl enamines reacted to give **4u** in 84% yield with 92% *ee*. N-methyl aliphatic acyl enamine was converted to **4v** in 79% yield with 80% *ee*.

**Fig. 4:**
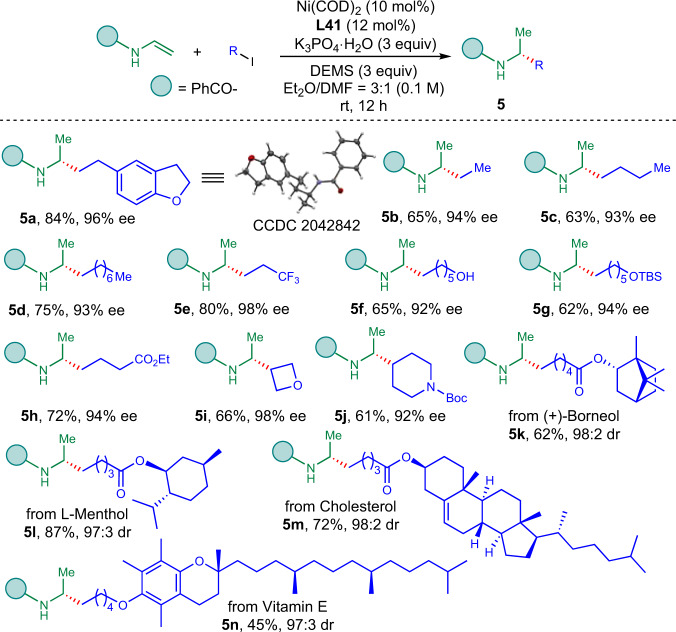
Scope for alkyl iodides with secondary acyl enamines. The reaction was conducted on 0.2 mmol of acyl enamine and 0.4 mmol of alkyl iodide (2.0 equiv.) under indicated conditions.

Then the scope for alkyl iodide for secondary acyl enamines was examined (Fig. 4). Secondary acyl enamines gave better enantioselectivity using the analogue ligand **L41**. Then, 5-(2-Iodoethyl)-2,3-dihydrobenzofuran was successfully hydroalkylated to give **5a** in 84% yield with 96% *ee*. The structure and absolute configuration of **5a** was further determined by the X-ray diffraction analysis. It is noteworthy that the minimal structurally different secondary alkyl-substituted amine derivative **5b** was obtained by this protocol in 65% yield with 94% *ee*. Other 1-iodoalkanes were also successfully converted to corresponding amine derivatives (**5c**–**5e**) in 63–80% yields with 93–98% *ee*. Chiral aminoalcohol and aminoester derivatives (**5f**–**5h**) were obtained in 62–72% yields with 92–94% *ee*. Cyclic secondary alkyl iodides were also reactive under the reaction conditions to furnish the desired products **5i** and **5j** in 66% and 61% yields with 98% and 92% *ee*. To demonstrate the robustness and usefulness of this protocol, we applied this reaction to late-stage functionalization of natural product derivatives. (+)-Borneol, L-menthol, cholesterol, and vitamin E derived acyl enamines could be transformed to give corresponding chiral amides (**5k**–**5n**) in 45–87% yields with 97:3 to 98:2 dr.

### Substrate scope of dialkyl esters

**Fig. 5:**
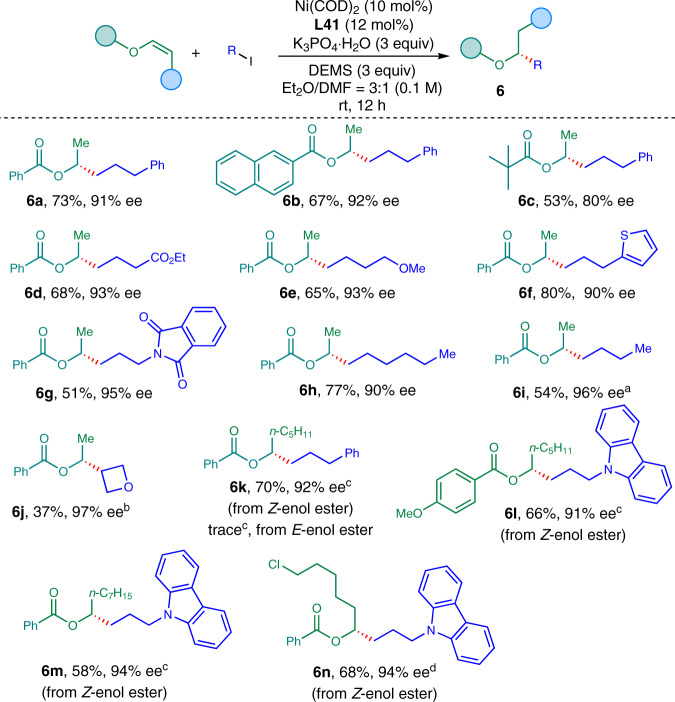
Scope for the hydroalkylation of enol esters. The reaction was conducted on 0.2 mmol of enol ester (1.0 equiv.) and alkyl iodide (2.0 equiv.) using potassium phosphate monohydrate (3.0 equiv.) as base in Et_2_O/DMF = 3:1 (0.1 M) at room temperature for 12 h unless otherwise stated. ^a^5.0 equiv. of RI was used. ^b^3.0 equiv. of RI was used. ^c^The reaction was conducted using alkyl iodide (3.0 equiv.) and potassium carbonate (3.0 equiv.) as base in Et_2_O/NMP = 3:1 (0.2 M) at 45 °C for 16 h. ^d^The reaction was conducted using alkyl iodide (3.0 equiv.) and potassium carbonate (3.0 equiv.) as base in the presence of *tert*-butanol (4.0 equiv.) in Et_2_O/NMP = 3:1 (0.2 M) at 45 °C for 16 h. NMP = N-methyl pyrrolidone.

Next, enol esters were tested under the reaction conditions. To our delight, various enol esters could be tolerated and a wide range of chiral aliphatic alcohol derivatives were obtained in high enantioselectivity, which are difficult to access otherwise (Fig. 5). Aromatic or aliphatic acid-derived enol esters were all good substrates for this reaction, furnishing corresponding chiral esters (**6a**–**6c**) in 53–73% yields with 80–92% *ee*. Alkyl iodides containing ester, ether, thiophene, amide could be transformed to corresponding chiral alcohol derivatives (**6d**–**6g**) in 51–80% yields with 90–95% *ee*. Notably, 1-iodohexane and 1-iodobutane were successfully involved in the reaction to give octan-2-ol (**6h**) and hexan-2-ol (**6i**) derivatives in 77% and 54% yields with 90% and 96% *ee*, respectively. Secondary alkyl iodide was tolerated in the reaction, furnishing the desired product (**6j**) in synthetic useful yields with 97% *ee*. Moreover, internal enol esters were well-tolerated in the reaction. Long-chain alkyl-substituted internal enol esters were successfully converted to corresponding chiral esters (**6k**–**6m**) in 58–70% yields with 91–94% *ee*. Chloro-containing alkyl-substituted internal enol ester underwent the desired hydroalkylation reaction to give **6n** in 68% yield with 94% *ee*. The absolute configuration of the chiral ester was confirmed to be *R* by comparison to literature^53–55^. Furthermore, literature procedures proved unprotected chiral aliphatic amines and alcohols could be obtained via hydrolysis without erosion of enantioselectivities^53,54^, which further enhanced the synthetic utility of this method.

### Mechanistic consideration

**Fig. 6:**
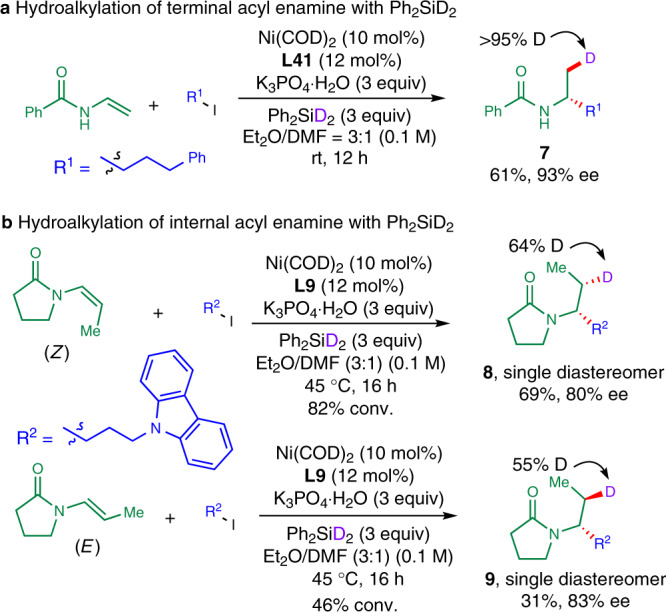
Mechanistic probe for the reaction. **a** Hydroalkylation of terminal acyl enamine with Ph_2_SiD_2_. **b** Hydroalkylation of internal acyl enamine with Ph_2_SiD_2_.

Then, we carried out the reaction using deuterated silane (Ph_2_SiD_2_)^32^ under otherwise identical to standard conditions (Fig. 6). The reaction of terminal acyl enamine with 3-phenyl-1-iodopropane in the presence of Ph_2_SiD_2_ afforded deuterated hydroalkylation product **7** in 61% yield with 93% *ee* (Fig. 6a). Only one deuterium incorporation (>95% D) was exclusively delivered to β*-*position to nitrogen of amide **7**. No deuterium incorporation was found at α-position to nitrogen of **7**. Next, the reactions of internal acyl enamine of both configurations were tested (Fig. 6b). The reaction of (*E*)-acyl enamine was slightly slower and delivered a lower yield and higher enantioselectivity of **9** in comparison to the generation of **8** from (*Z*)-acyl enamine^50,51^. These results indicated that Ni–H insertion onto acyl enamines to form alkyl-Ni species might be irreversible and enantio-determining.

**Fig. 7:**
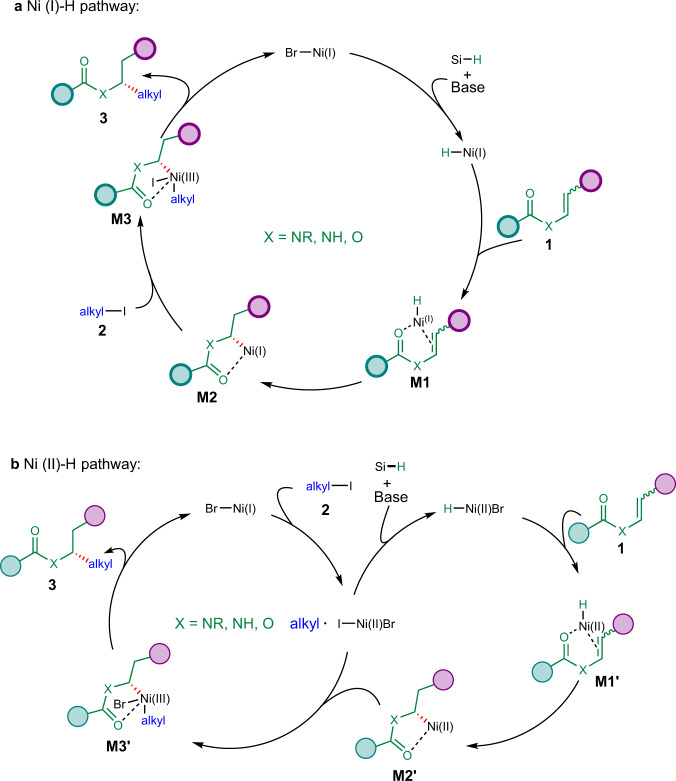
Proposed mechanism for the reaction. Ligand is omitted for clarity. **a** Ni(I)–H pathway. **b** Ni(II)–H pathway.

Based on the mechanistic results and literature precedence^28–34,42–45,48,49^, two tentative mechanistic pathways are proposed and depicted in Fig. 7. In one possibility (Fig. 7a), nickel hydride species could be generated from ligated Ni(I) precursor in the presence of a silane and a base. Ni–H would coordinate with acyl enamines or enol esters (**1**) to give **M1**, which could undergo regio- and enantioselective hydrometalation to generate alkyl nickel intermediate **M2**. This **M2** could oxidize an alkyl iodide (**2**) to form Ni(III) intermediate **M3**, which could undergo reductive elimination to give the final product **3** and regenerate Ni(I) catalyst. In the other possibility (Fig. 7b), ligated Ni(I) precursor undergoes single electron transfer with an alkyl iodide (**2**) to give an alkyl radical and Ni(II) intermediate. The latter could generate Ni(II)-H in the presence of a silane and a base, which could coordinate with **1** with the assistance of carbonyl group to form **M1ʹ**. With regio- and enantioselective hydrometalation, **M1ʹ** generates alkyl nickel intermediate **M2ʹ**, which could rebound with the alkyl radical to form Ni(III) intermediate **M3ʹ**. **M3ʹ** undergoes reductive elimination to deliver the final product **3** and regenerate Ni(I) species.

## Discussion

In summary, a unified protocol for Ni-catalyzed hydroalkylation of acyl enamines and enol esters with alkyl iodides under mild conditions was developed. The use of chiral BOX-based ligand enables the direct access of chiral secondary alkyl-substituted amine and alcohol derivatives in good yields with excellent levels of enantioselectivity, providing a straightforward alternative to pure aliphatic amine and alcohol derivatives which are traditionally challenging to access.

## Methods

### General procedure for hydroalkylation of tertiary acyl enamines

In a nitrogen-filled glovebox, Ni(COD)_2_ (5.5 mg, 0.02 mmol, 10 mol%) and **L9** (21.8 mg, 0.024 mmol, 12 mol%) were dissolved in solvent (2 mL, Et_2_O: DMF = 3:1) in a Schlenk tube with screw-cap equipped with a magnetic stirrer. The mixture was stirred at room temperature for 10 min, then alkyl halide (0.4 mmol), tertiary acyl enamine (0.2 mmol), and K_3_PO_4_·H_2_O (0.6 mmol) were added sequentially. The mixture was cooled to 0 °C before DMMS (74 μL, 0.6 mmol, 3 equiv.) was added dropwise. The resulting mixture was stirred at 0 °C for 12–24 h (for **3o**–**s**, stirred at 45 °C). After completion of the reaction, the mixture was filtered through a pad of silica gel and washed with ethyl acetate (3 × 15 mL). The filtrate was washed with water (15 mL). The organic phase was dried over Na_2_SO_4_, filtered, concentrated under reduced pressure, purified by flash chromatography with silica gel to give the pure product.

### General procedure for hydroalkylation of secondary acyl enamines

In a nitrogen-filled glovebox, Ni(COD)_2_ (5.5 mg, 0.02 mmol, 10 mol%) and **L41** (8.4 mg, 0.024 mmol, 12 mol%) were dissolved in solvent (2 mL, Et_2_O: DMF = 3:1) in a Schlenk tube with screw-cap equipped with a magnetic stirrer. The mixture was stirred at room temperature for 10 min, then alkyl halide (0.4 mmol), acyl enamine (0.2 mmol), and K_3_PO_4_·H_2_O (0.6 mmol) were added sequentially. The mixture was stirred at room temperature for another 5 min before DEMS (98 μL, 0.6 mmol, 3 equiv.) was added dropwise. The resulting mixture was stirred at room temperature for 12–24 h. After completion of the reaction, the mixture was filtered through a pad of silica gel and washed with ethyl acetate (3 × 15 mL). The filtrate was washed with water (15 mL). The organic phase was dried over Na_2_SO_4_, filtered, concentrated under reduced pressure, purified by flash chromatography with silica gel to give the pure product.

### General procedure for hydroalkylation of enol esters

In a nitrogen-filled glovebox, Ni(COD)_2_ (5.5 mg, 0.02 mmol, 10 mol%) and **L41** (8.4 mg, 0.024 mmol, 12 mol%) were dissolved in solvent (2 mL, Et_2_O: DMF = 3:1) in a Schlenk tube with screw-cap equipped with a magnetic stirrer. The mixture was stirred at room temperature for 10 min, then alkyl halide (0.4 mmol) was added and the mixture was stirred for another 5 min, followed by the sequential addition of enol esters (0.2 mmol) and K_3_PO_4_·H_2_O (0.6 mmol). The mixture was stirred at room temperature for 5 min before DEMS (98 μL, 0.6 mmol) was added dropwise. The resulting mixture was stirred at room temperature for 16–20 h. After completion of the reaction, the mixture was filtered through a pad of silica gel and washed with ethyl acetate (3 × 15 mL). The filtrate was washed with water (15 mL). The organic phase was dried over Na_2_SO_4_, filtered, concentrated under reduced pressure, and purified by flash chromatography with silica gel to give the pure product.

## Supplementary information

Supplementary Information

## Data Availability

The authors declare that all other data supporting the findings of this study are available within the article and Supplementary information files, and also are available from the corresponding author upon reasonable request. The X-ray crystallographic coordinates for structures reported in this study have been deposited at the Cambridge Crystallographic Data Centre (CCDC), under deposition numbers of CCDC 2042842 and CCDC 2042844. These data can be obtained free of charge from The Cambridge Crystallographic Data Centre via www.ccdc.cam.ac.uk/data_request/cif.
