# Supplementary material for: Enantioselective access to chiral aliphatic amines and alcohols via Ni-catalyzed hydroalkylations
Source: Nat Commun. 2021 May 13;12:2771. doi: 10.1038/s41467-021-22983-7 (PMC8119980; doi:10.1038/s41467-021-22983-7)
Supplement: Supplementary file 1 — Supplementary Information [file 41467_2021_22983_MOESM1_ESM.pdf]

Enantioselective Access to Chiral Aliphatic Amines and Alcohols via Ni-Catalyzed Hydroalkylations

Shan Wang,[†] Jian-Xin Zhang,[†] Tian-Yi Zhang,[†] Huan Meng,[†] Bi-Hong Chen,[†] and Wei Shu[†,*]

[†]*Shenzhen Grubbs Institute and Department of Chemistry, Guangdong Provincial Key Laboratory of Catalysis, Southern University of Science and Technology, Shenzhen 518055, Guangdong, P. R. China*

[*]E-mail: shuw@sustech.edu.cn

Supplementary Note 1 S2

Supplementary Methods S3

Supplementary Tables S9

Supplementary Note 2 S19

- **Characterization of Products** S19
- **Mechanistic Studies** S50
- **X-Ray Diffraction Data of 4l and 5a** S57

Supplementary Figures S61

- **^{1}H NMR, ^{13}C NMR and ^{19}F NMR Spectra of Products** S61
- **HPLC Traces of Products** S132

Supplementary References S205

Supplementary Note 1

General Information

NMR spectra were recorded on 400 MHz or 600 MHz Bruker spectrometers. Chemical shifts are given in ppm. The spectra are calibrated to the residual ^{1}H and ^{13}C signals of the solvents. Multiplicities are abbreviated as follows: singlet (s), doublet (d), triplet (t), quartet (q), doublet-doublet (dd), quintet (quint), septet (sept), multiplet (m), and broad (br). High-pressure liquid chromatography (HPLC) was performed on Agilent 1200 Series chromatographs using a chiral column (25 cm) as noted for each compound. Enantiomer excess was determined by HPLC analysis employing Darcel Chiracel OD-H, AD-H, AS-H, IA and OJ-H column. High-resolution electrospray ionization and electronic impact mass spectrometry was performed on a Finnigan MAT 900 (Thermo Finnigan, San Jose, CA; USA) double focusing magnetic sector mass spectrometer. A mass accuracy $\leq$ 2 ppm was obtained in the peak matching acquisition mode by using a solution containing 2 <l PEG200, 2 <l PPG450, and 1.5 mg NaOAc (all obtained from Sigma-Aldrich, CH-Buchs) dissolved in 100 mL MeOH (HPLC Supra grade, Scharlau, E-Barcelona) as internal standard.

Materials: Unless otherwise noted, commercial reagents were purchased from Energy-Chemical Limited, J&K, Adamas-beta®, Aladdin, Macklin Reagent, Bidepharm and used directly without further purification. Ether was distilled over CaH_2 and stored under nitrogen atmosphere.

Supplementary Methods

Supplementary Method 1: General Procedure for the Preparation of Acyl Enamines

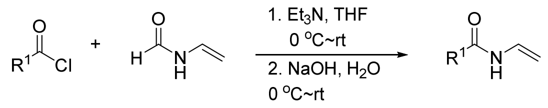

Acyl enamines were prepared following literature procedure.[1] N-vinylformamide (10 mmol, 1.0 equiv), triethylamine (12 mmol, 1.2 equiv), and anhydrous THF (30 mL) were added to a three-necked round-bottomed flask equipped with an overhead stirrer, an addition funnel, and a nitrogen balloon. The mixture was cooled to 0 °C in an icewater bath. Freshly distilled aryl chloride (11.5 mmol, 1.15 equiv) was loaded into the addition funnel and slowly added at a rate such that temperature was maintained below 5 °C over 1 h. A solution of 5 N NaOH (30 mmol/6 mL H_2O) was then slowly added at 0-5 °C over 2 h. The water layer was removed and extracted with EtOAc. The organic layers were combined, washed with a solution of NaCl, and dried over anhydrous Na_2SO_4. After filtering off insoluble, the solvents were evaporated under reduced pressure using rotary evaporation, the residue was purified by chromatography on silica gel to afford the products. Characterization data is consistent with reported data.

Supplementary Method 2: General Procedure for the Preparation of Internal Acyl Enamines[6]

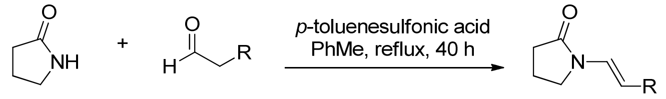

A solution of 10 mmol of the appropriate lactam or amide, 10 mmol of the desired aldehyde, and 5 mg of p-toluenesulfonic acid in 15-20 mL of dry toluene was heated at reflux with water removal by a Dean-Stark trap for 10-24 h until no more water was collected. The solution was then cooled to room temperature, and washed with 10 mL of ether. The organic phases were combined and dried ($MgSO_4$), filtered and concentrated. The residue was then either recrystallized from ethyl acetate or purified by flash chromatography on silica gel. Characterization data is consistent with reported

data.

Supplementary Method 3: General Procedure for the Preparation of Terminal Enol Esters

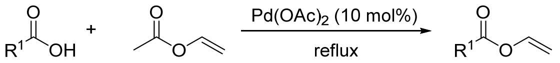

Enol esters were prepared following literature procedure.[2] To a pressure tube were added acid (10 mmol, 1.0 equiv), $Pd(OAc)_2$ (0.22 g, 1.0 mmol, 0.1 equiv) and vinyl acetate (20 mL). The reaction mixture was heated to reflux and stirred for 8 h at 80 ºC. After cooling down to room temperature, the mixture was filtered through a short pad of Celite and washed with CH_2Cl_2. The filtrate was concentrated under reduced pressure, and the residue was purified by column chromatography on silica gel with gradient of petroleum ether/ethyl acetate to afford products. Characterization data is consistent with reported data.

Supplementary Method 4: General Procedure for the Preparation of Internal Enol Esters[7]

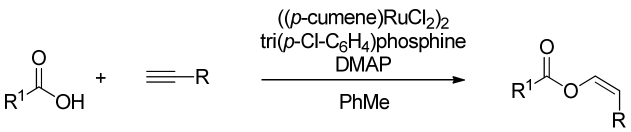

A solution of ((*p*-cumene)$RuCl_2$)$_2$ (30.6 mg, 0.05 mmol), tri(*p*-Cl-C_6H_4)phosphine (54.8 mg, 0.15 mmol) and DMAP (24.4 mg, 0.20 mmol) in dry toluene (4 ml) was added to a solution of benzoic acid (588 mg, 5.00 mmol) and alkyne (6.50 mmol) in dry toluene (16 ml). The mixture was stirred for 16 h at 60 °C. After complete conversion (GC), usually 16 h, the mixture was cooled and filtered over a small plug of silica gel. The solvent was removed and the crude mixture was purified by column chromatography on silica gel with gradient of petroleum ether/ethyl acetate to afford products. Characterization data is consistent with reported data.

Supplementary Method 5: Preparation of Chiral Ligand L9:

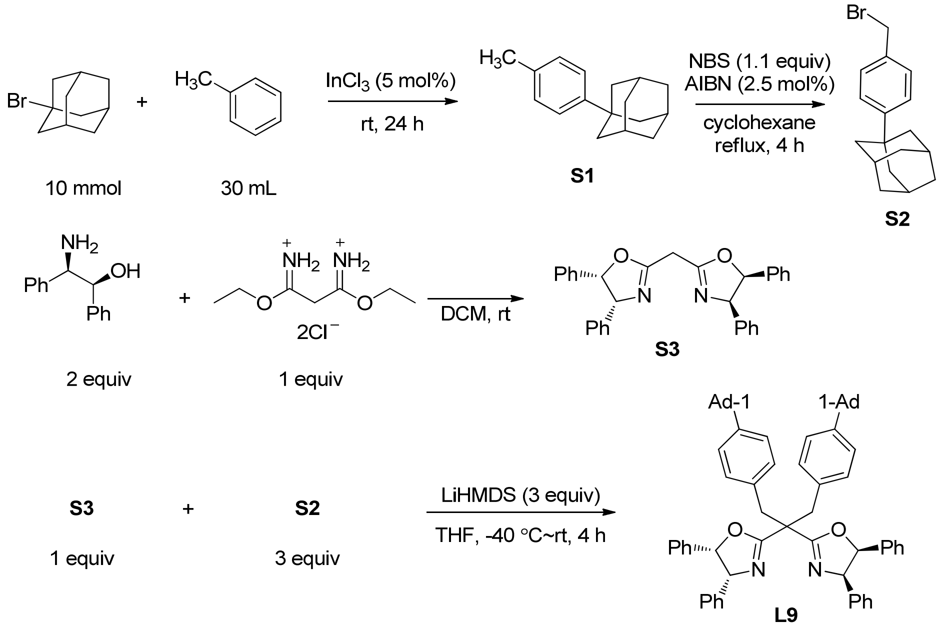

Compound S1 was synthesized according to literature.[3] Under argon atmosphere 1-bromoadamantane (10 mmol), toluene (35 mL) and a catalytic amount of $InCl_3$ (5 mol%) added to a round bottom flask. After stirring for 24 h at room temperature, the mixture was washed by water (3×50 mL) until the pH was neutralized. Then the mixture was extracted with ether spirit (3×50 mL). The organic layer was dried over anhydrous MgSO4 and concentrated under vacuum. The solid residue was filtered in a small column with hexane give the **compound S1** as crystalline solid in 93% yield.

Compound S2 was synthesized according to literature.[3] **Compound S1** (4.42 mmol), N-bromosuccinimide (NBS, 4.86 mmol), 2.2-azobisisobutyronitrile (AIBN, 0.11 mmol) and cyclohexane (7 mL) were added to a round bottom flask at room temperature. The mixture was stirred and refluxed for 2 h. The reaction was monitored by TLC. Then the mixture was allowed to cool to room temperature and water (30 mL) was added and reside was extracted with ethyl acetate (3×10 mL), dried over anhydrous $MgSO_4$ and concentrated under vacuum. The residue was filtered in a small silica column and to give the title **compound S2** as white solid in 85% yield.

Compound S3 was synthesized according to literature.[4] A mixture of (1*R*,2*S*)-2-amino-1,2-diphenylethan-1-ol (10.0 mmol) and diethyl malonimidate dihydrochloride (10.0 mmol) in CH_2Cl_2 was stirred at room temperature for 24 hours. Water was added and

the organic layer was separated, dried over Na_2SO_4, filtered and concentrated in vacuo. The residue was purified by chromatography on silica gel to give **compound S3** as a white solid in 81% yield.

To a flask with **compound S3** (10.0 mmol) in dry THF (30 mL) was added LiHMDS (30.0 mmol) at -40 °C and stirred at this temperature for 0.5 h, then **compound S2** (30 mmol) was added. After the addition, the cooling bath was removed and the reaction mixture was allowed to warm to room temperature to continue reaction for 3 h. After the reaction was completed, the reaction mixture was quenched with saturated aqueous NH_4Cl and the aqueous layer was extracted with diethyl ether (3 × 50 mL). The combined organic layer was washed with saturated aqueous brine, dried over anhydrous Na_2SO_4, filtered and concentrated in vacuum. The residue was purified by chromatography on silica gel to give the **L9** as a white solid in 75% yield.[5]

(4*R*,4'*R*,5*S*,5'*S*)-2,2'-(1-(4-((1*r*,3*R*,5*S*)-adamantan-1-yl)phenyl)-3-(4-((1*s*,3*R*)-adamantan-1-yl)phenyl)propane-2,2-diyl)bis(4,5-diphenyl-4,5-dihydrooxazole) (L9)

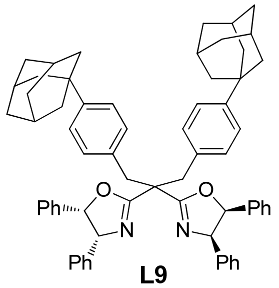

L9

White solid, 48% yield.

^{1}H NMR (400 MHz, $CDCl_3$) δ 7.46 (d, *J* = 8.4 Hz, 2H), 7.37 (d, *J* = 8.4 Hz, 2H), 7.02 – 6.86 (m, 10H), 5.77 (d, *J* = 10.4 Hz, 1H), 5.41 (d, *J* = 10.4 Hz, 1H), 3.83 (d, *J* = 14.0 Hz, 1H), 3.51 (d, *J* = 14.0 Hz, 1H), 2.12 (s, 3H), 1.96 (d, *J* = 3.2 Hz, 6H), 1.84 – 1.76 (m,6H), 1.57 (s, 22H).

^{13}C NMR (151 MHz, $CDCl_3$) δ 168.26, 150.12, 137.47, 135.92, 133.95, 130.62, 128.13, 127.64, 127.47, 127.06, 127.02, 124.78, 86.27, 73.69, 49.56, 43.42, 39.81, 37.00, 36.17, 29.15.

HRMS (ESI-TOF) Calcd for $C_{65}H_{66}N_2O_2$ $(M+H)^+$ 907.5197. Found 907.5194.

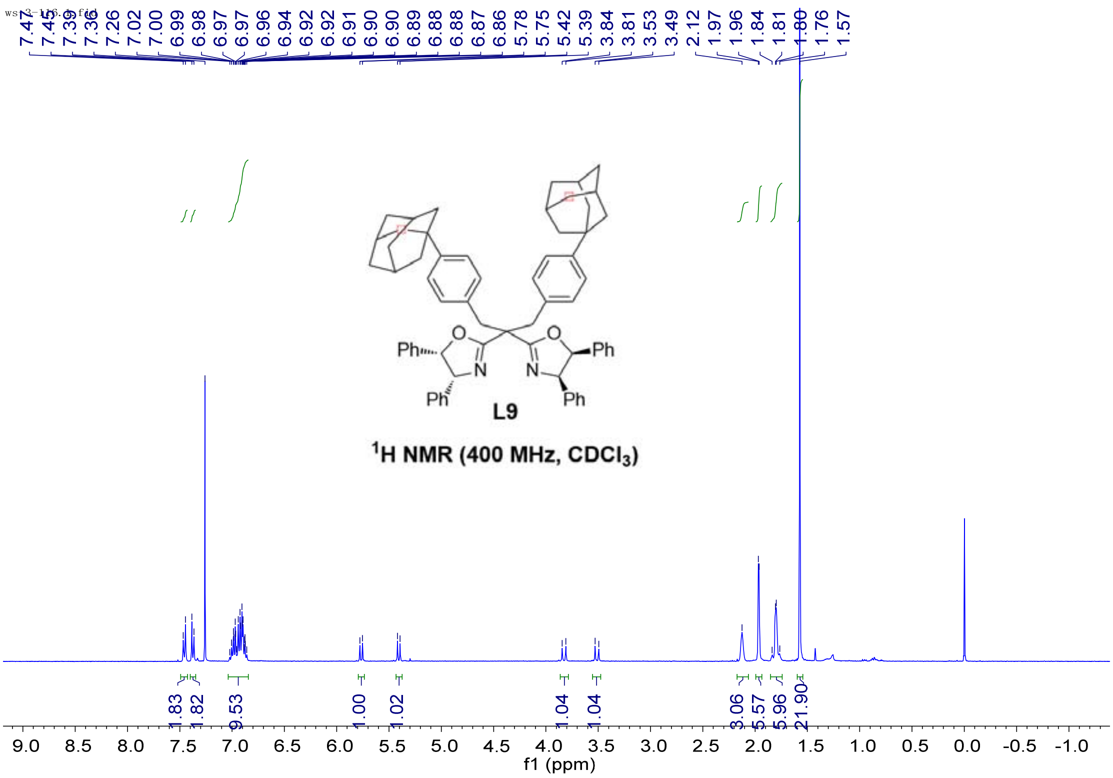

Supplementary Figure 1 ^{1}H NMR (400 MHz, $CDCl_3$) of **L9**

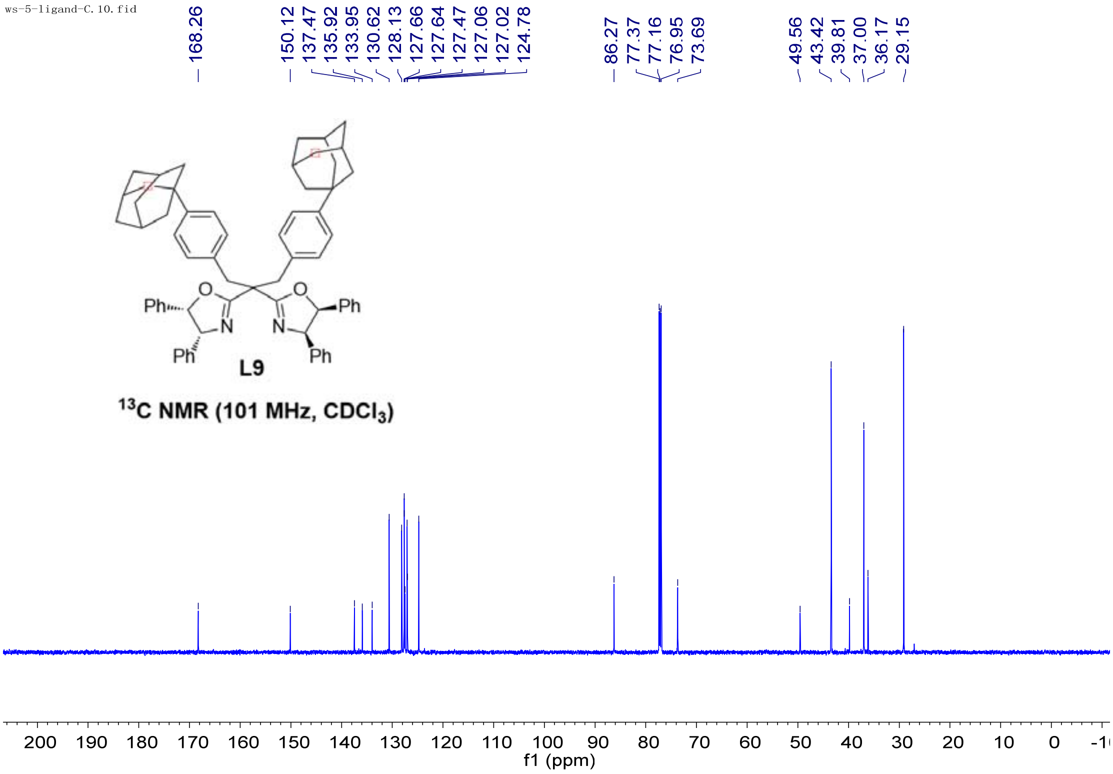

Supplementary Figure 2 ^{13}C NMR (101 MHz, $CDCl_3$) of **L9**

General Procedures for Hydroalkylations

Supplementary Method 6: (**3a-3n**): In a nitrogen-filled glovebox, Ni(COD)$_2$ (5.5 mg, 0.02 mmol, 10 mol%) and (1*S*, 2*R*)-**L9** (21.8 mg, 0.024 mmol, 12 mol%) were dissolved

in solvent (2 mL, Et_2O: DMF = 3:1) in Schlenk tube with screw-cap equipped with a magnetic stirrer. The mixture was stirred at room temperature for 10 min, then alkyl halides (0.4 mmol, 2 equiv), acyl enamines (0.2 mmol, 1 equiv), K_3PO_4•H_2O (0.6 mmol, 3 equiv) were sequentially added, the mixture was cooled to 0 °C before silane (0.6 mmol, 3 equiv) was added dropwise. The resulting mixture was stirred at 0 °C for 24 h. The mixture was filtered through a pad of silica gel and washed with ethyl acetate (3 × 15 mL), then washed with water (15 mL). The organic phase was dried over Na_2SO_4, filtered, concentrated under reduced pressure, purified by flash chromatography with silica gel to give the pure product.

Supplementary Method 7: (4a-4v): In a nitrogen-filled glovebox, Ni(COD)$_2$ (5.5 mg, 0.02 mmol, 10 mol%) and (1*S*, 2*R*)-**L41** (8.4 mg, 0.024 mmol, 12 mol%) were dissolved in solvent (2 mL, Et_2O: DMF = 3:1) in Schlenk tube with screw-cap equipped with a magnetic stirrer. The mixture was stirred at room temperature for 10 min, then alkyl halides (0.4 mmol, 2 equiv), acyl enamines (0.2 mmol, 1 equiv), K_3PO_4•H_2O (0.6 mmol, 3 equiv) were sequentially added, the mixture was stirred at room temperature for another 5 minutes before silane (0.6 mmol, 3 equiv) was added dropwise. The resulting mixture was stirred at room temperature for 12 h. The mixture was filtered through a pad of silica gel and washed with ethyl acetate (3 × 15 mL), then washed with water (15 mL). The organic phase was dried over Na_2SO_4, filtered, concentrated under reduced pressure, purified by flash chromatography with silica gel to give the pure product.

Supplementary Method 8: (5a-5m, **6a-6j)**: In a nitrogen-filled glovebox, Ni(COD)$_2$ (5.5 mg, 0.02 mmol, 10 mol%) and (1*S*, 2*R*)-**L41** (8.4 mg, 0.024 mmol, 12 mol%) were dissolved in solvent (2 mL, Et_2O: DMF = 3:1) in Schlenk tube with screw-cap equipped with a magnetic stirrer. The mixture was stirred at room temperature for 10 min, then alkyl halides (0.4 mmol, 2 equiv) was added and the mixture was stirred for another 5 minutes, followed by the sequential addition of acyl enamines/enol esters (0.2 mmol, 1 equiv), K_3PO_4•H_2O (0.6 mmol, 3 equiv). The mixture was stirred at room temperature for 5 minutes before silane (0.6 mmol, 3 equiv) was added dropwise. The resulting mixture was stirred at room temperature for 20 h. The mixture was filtered through a

pad of silica gel and washed with ethyl acetate (3 × 15 mL), then washed with water (15 mL). The organic phase was dried over Na_2SO_4, filtered, concentrated under reduced pressure, purified by flash chromatography with silica gel to give the pure product.

Supplementary Method 9: (3o-3s): In a nitrogen-filled glovebox, $Ni(COD)_2$ (5.5 mg, 0.02 mmol, 10 mol%) and (1*S*, 2*R*)-**L9** (21.8 mg, 0.024 mmol, 12 mol%) were dissolved in solvent (2 mL, Et_2O: DMF = 3:1) in Schlenk tube with screw-cap equipped with a magnetic stirrer. The mixture was stirred at room temperature for 10 min, then acyl enamines (0.2 mmol, 1 equiv), alkyl halides (0.6 mmol, 3 equiv), K_3PO_4•H_2O (0.6 mmol, 3 equiv) were sequentially added, The mixture was stirred at room temperature for 5 minutes before silane (0.6 mmol, 3 equiv) was added dropwise. The resulting mixture was stirred at 45 °C for 16 h. The mixture was filtered through a pad of silica gel and washed with ethyl acetate (3 × 15 mL), then washed with water (15 mL). The organic phase was dried over Na_2SO_4, filtered, concentrated under reduced pressure, purified by flash chromatography with silica gel to give the pure product.

Supplementary Method 10: (6k-6n): In a nitrogen-filled glovebox, $Ni(COD)_2$ (5.5 mg, 0.02 mmol, 10 mol%) and (1*S*, 2*R*)-**L41** (8.4 mg, 0.024 mmol, 12 mol%) were dissolved in solvent (1 mL, Et_2O: NMP = 3:1) in Schlenk tube with screw-cap equipped with a magnetic stirrer. The mixture was stirred at room temperature for 10 min, then alkyl halides (0.6 mmol, 3 equiv) was added and the mixture was stirred for another 5 minutes, followed by the sequential addition of enol esters (0.2 mmol, 1 equiv), K_2CO_3 (0.6 mmol, 3 equiv). The mixture was stirred at room temperature for 5 minutes before silane (0.6 mmol, 3 equiv) was added dropwise. The resulting mixture was stirred at °C for 16 h. The mixture was filtered through a pad of silica gel and washed with ethyl acetate (3 × 15 mL), then washed with water (15 mL). The organic phase was dried over Na_2SO_4, filtered, concentrated under reduced pressure, purified by flash chromatography with silica gel to give the pure product.

Supplementary Tables

Supplementary Table 1 Effect of Ligand

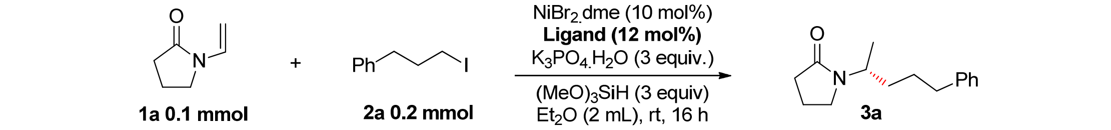

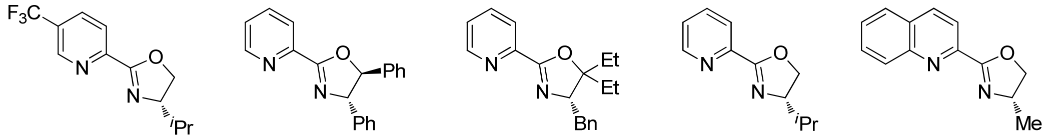

L10, 35% yield, 2% ee **L11**, trace yield **L12**, 80% yield, 2% ee **L13**, 6% yield **L14**, 28% yield, 17% ee

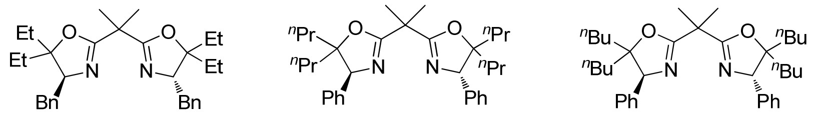

L15, 37% yield, 20% ee **L16**, 53% yield, 67% ee **L17**, 33% yield, 68% ee

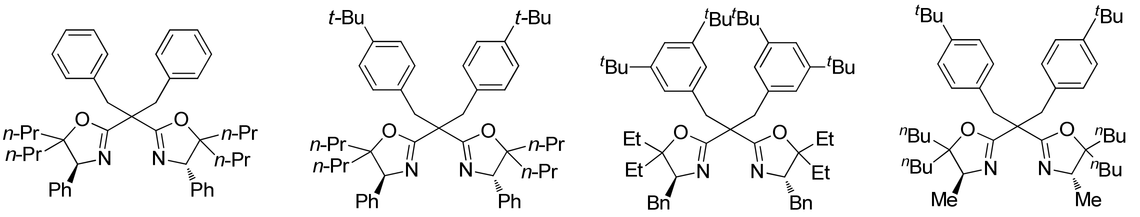

L18, 23% yield, 25% ee **L19**, 20% yield, 35% ee **L20**, trace yield **L21**, 15% yield, 67% ee

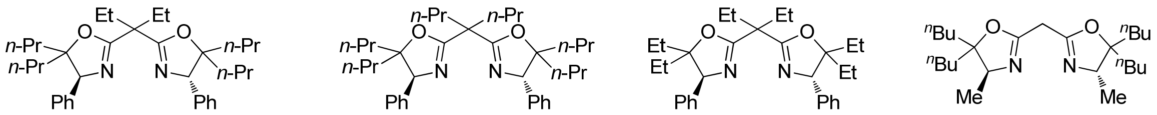

L22, 33% yield, 53% ee **L23**, 32% yield, 51% ee **L24**, 27% yield, 47% ee **L25**, 56% yield, 21% ee

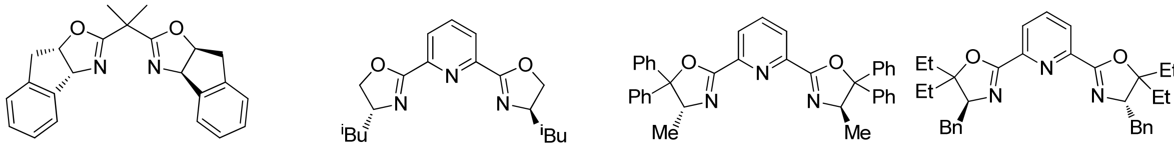

L26, trace yield **L27**, < 10% yield **L28**, < 10% yield **L29**, 30% yield, 5% ee

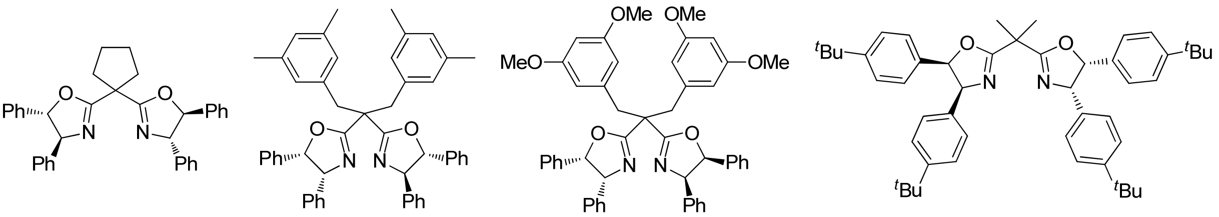

L30, 23% yield, 9% ee **L31**, 37% yield, 29% ee **L32**, 15% yield, 84% ee **L33**, 66% yield, 23% ee

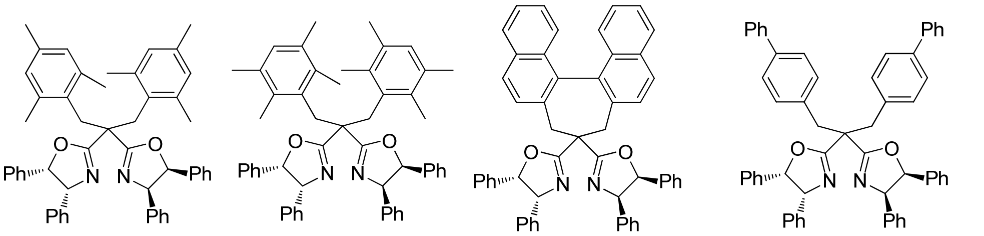

L34, 10% yield, 75% ee **L35**, 25% yield, 50% ee **L36**, 17% yield, 33% ee **L37**, 11% yield, 70% ee

The yield was determined by GC analysis with *n*-dodecane as internal standard. The ee value determined by HPLC analysis on a chiral stationary phase.

Supplementary Table 2 Effect of Silane

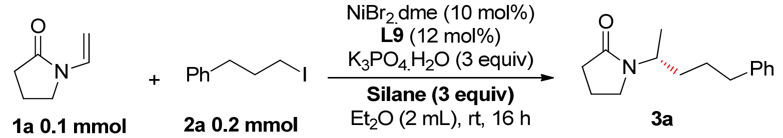

1a 0.1 mmol **2a 0.2 mmol** **3a**

entry	Silane	Yield (%)	ee (%)
1	$(MeO)_3SiH$	17	90
2	**$(EtO)_3SiH$**	14	**94**
3	Ph_3SiH	3	--
4	Ph_2SiH_2	56	64
5	**DEMS**	24	**94**
6□	$(MeO)_2MeSiH$	31	90
7	PMHS	9	82
8□	$(Me_2SiH)_2O$	22	60

The yield was determined by GC analysis with *n*-dodecane as internal standard. The ee value was determined by HPLC analysis on a chiral stationary phase.

Supplementary Table 3 Effect of Additive

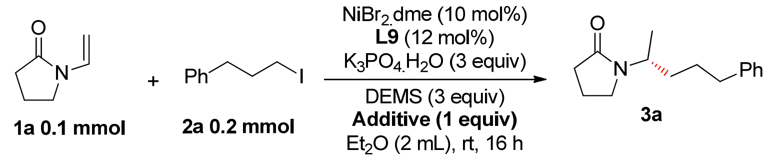

entry	Additive	Yield (%)	ee (%)
1	H_2O	37	76
2	MeOH	64	74
3	EtOH	60	87
4	^{n}PrOH	67	82
5	^{n}BuOH	64	89
6	1-PenOH	38	92
7	1-HexOH	39	95
8	$CpCH_2OH$	47	88
9	$CyCH_2OH$	42	82
10	$PhCH_2OH$	47	91
11	^{i}PrOH	45	93
12	HFIP	47	35
13	^{i}BuOH	36	92
14	^{i}PenOH	44	92
15	CyOH	35	65
**16**	**^{t}BuOH**	**48**	**93**
17	^{t}PenOH	21	96

The yield was determined by GC analysis with *n*-dodecane as internal standard. The ee value was determined by HPLC analysis on a chiral stationary phase.

Supplementary Table 4 Effect of Solvent

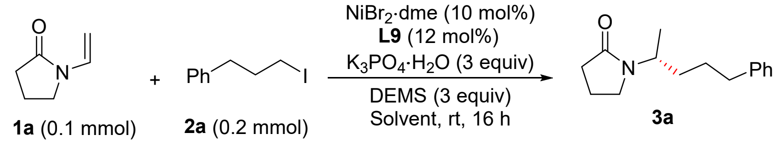

entry	Solvent (2 mL)	Yield (%)	ee (%)
1	Et_2O	24	94
2	$^nPr_2O$	13	92
3	$^nBu_2O$	3	80
4	^{t}BuOMe	41	88
5	$^iPr_2O$	10	92
6	PhOMe	25	70
7	CPME	3	--
8	**DMA**	**>99**	**73**
9	glyme	62	66
10	diglyme	85	69
11	PhMe	18	57
12	DMF	56	58
13	CH_3CN	99	5
14	DMI	50	67
15	NMP	99	36
16	DMSO	78	3
17	$PhCF_3$	97	75
18	PhCl	34	76
19	MeOH	0	--
20	EtOH	trace	--
21	^{i}PrOH	57	83
22	^{t}BuOH	77	75
23	^{n}BuOH	76	50
24	^{i}PenOH	79	55
25	CyOH	0	--
26	HFIP	0	--

The yield was determined by GC analysis with *n*-dodecane as internal standard. The ee value was determined by HPLC analysis on a chiral stationary phase.

Supplementary Table 5 Effect of Co-solvent

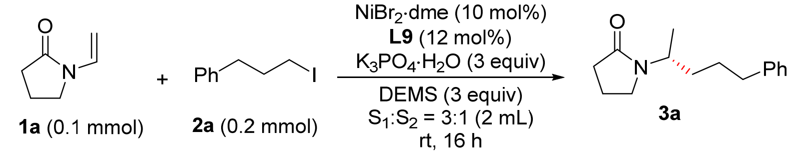

entry	S1:S2	Yield (%)	ee (%)
1	Et_2O:DMA	98	77
**2**	**Et_2O:DMF**	**99**	**84**
3	Et_2O:DMPU	trace	--
4	Et_2O:DCE	44	69
5	Et_2O:DMI	97	80
6	Et_2O:CH_3CN	60	76
7	Et_2O:Diglyme	46	82
8	^{t}BuOMe:DMF	98	84
9	THF:DMF	99	77
10	2-Me-THF:DMF	94	81
11	Dioxane:DMF	85	75
12	diglyme:DMF	90	77

The yield was determined by GC analysis with *n*-dodecane as internal standard. The ee value was determined by HPLC analysis on a chiral stationary phase.

Supplementary Table 6 Effect of Solvent Ratio

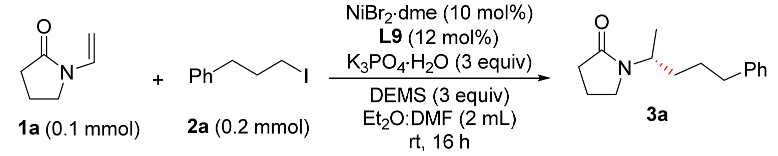

entry	Et_2O:DMF	Yield (%)	ee (%)
1	1 : 19	70	56
2	1 : 9	61	57
3	1 : 6	86	53
4	1 : 4	78	60
5	1 : 2	99	65
6	1 : 1	99	75
7	2 : 1	99	80
8	4 : 1	99	83
9	6 : 1	99	82
10	8 : 1	99	81
11	10: 1	87	77

The yield was determined by GC analysis with *n*-dodecane as internal standard. The ee value was determined by HPLC analysis on a chiral stationary phase.

Supplementary Table 7 Effect of Concentration

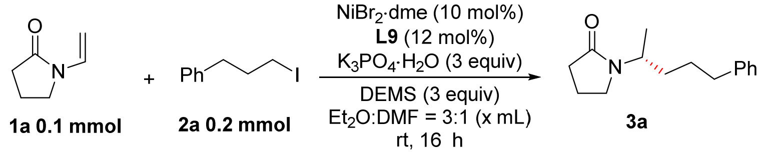

entry	Solvent (x mL)	Yield (%)	ee (%)
1	0.5 mL	>99	85
**2**	**1.0 mL**	**>99**	**86**
3	2.0 mL	>99	84
4	3.0 mL	>99	81
5	4.0 mL	96	77
6	5.0 mL	89	76

The yield was determined by GC analysis with *n*-dodecane as internal standard. The ee value was determined by HPLC analysis on a chiral stationary phase.

Supplementary Table 8 Effect of Catalyst

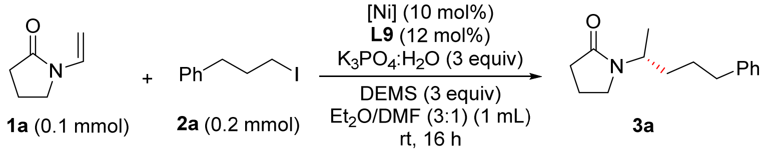

entry	[Ni]	Yield (%)	ee (%)
1	Ni(COD)$_2$	>99	88
2	NiCl$_2$·dme	>99	86
3	Ni(ClO$_4$)$_2$	>99	87

The yield was determined by GC analysis with *n*-dodecane as internal standard. The ee value was determined by HPLC analysis on a chiral stationary phase.

Supplementary Table 9 Effect of Base

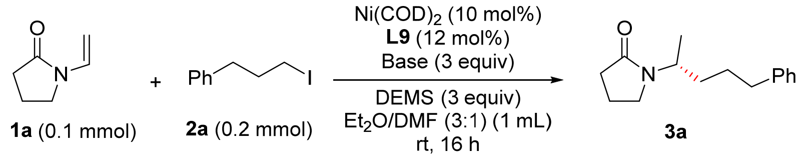

1a (0.1 mmol) + **2a** (0.2 mmol) → **3a**

entry	Base	Yield (%)	ee (%)
1	K_3PO_4	26	83
2	$K_3PO_4.3H_2O$	>99	88
3	$K_3PO_4.H_2O$	>99	88
4	K_2CO_3	40	85
5	Cs_2CO_3	18	--

The yield was determined by GC analysis with *n*-dodecane as internal standard. The ee value was determined by HPLC analysis on a chiral stationary phase.

Supplementary Table 10 Effect of Substrate Ratio

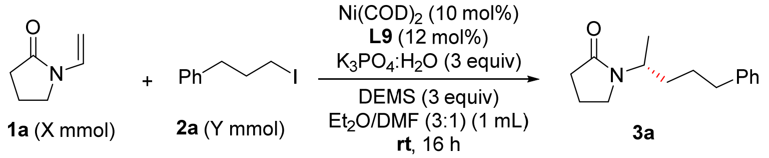

1a (X mmol) + **2a** (Y mmol) → **3a**

entry	X	Y	Yield (%)	ee (%)
1	0.1	0.1	83	88
2	0.1	0.2	>99	88
3	0.1	0.3	>99	88
4	0.2	0.1	77	88

The yield was determined by GC analysis with *n*-dodecane as internal standard. The ee value was determined by HPLC analysis on a chiral stationary phase.

Supplementary Table 11 Effect of Temperature and Silane

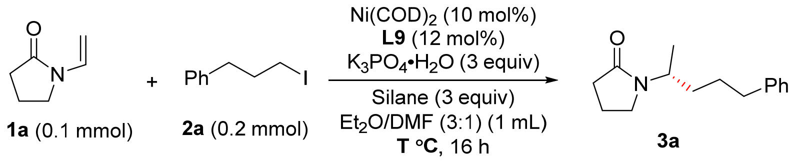

entry	T	Silane	Yield (%)	ee (%)
1	-10 °C	DEMS	63	93
2	0 °C	DEMS	94(81%[a])	92
3	rt	DEMS	>99	88
4	40	DEMS	>99	86
5	rt	DMMS	>99	89
6[b]	0 °C	DMMS	>99(93%[a])	92

The yield was determined by GC analysis with *n*-dodecane as internal standard. The ee value was determined by HPLC analysis on a chiral stationary phase. [a] Isolated yield. [b] The reaction time was 24 h.

Supplementary Table 12 Ligand Effect for Secondary Acyl Enamine

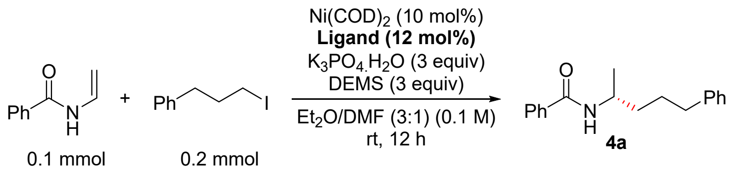

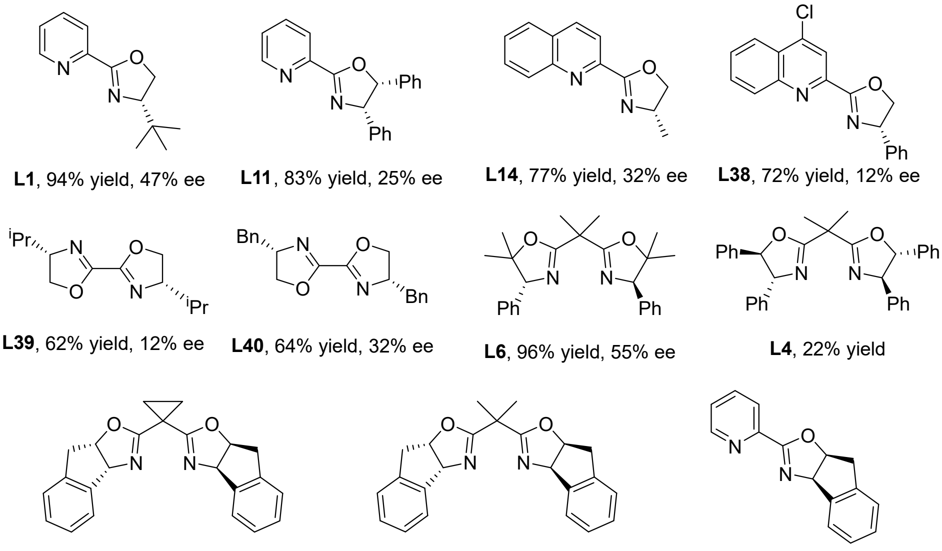

L1, 94% yield, 47% ee

L11, 83% yield, 25% ee

L14, 77% yield, 32% ee

L38, 72% yield, 12% ee

L39, 62% yield, 12% ee

L40, 64% yield, 32% ee

L6, 96% yield, 55% ee

L4, 22% yield

L41, 84% yield[a], 93% ee

L26, >95% yield, 88% ee

L42, >95% yield, 40% ee

The yield was determined by GC analysis with *n*-dodecane as internal standard. The ee value was determined by HPLC analysis on a chiral stationary phase. [a] Isolated yield.

Supplementary Table 13 Optimization of Internal Acyl Enamines

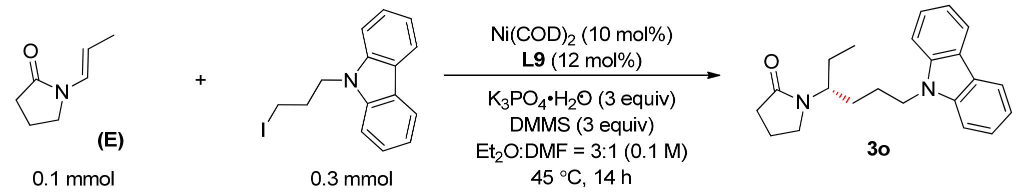

Entry	Variation	yield (**3o**)	ee
1	none	74% isolated yield	90%
2	2 equiv alkyl iodide	52%	91%
3	rt, DMF as the solvent	n.d.	--
4	rt, DMA as the solvent	n.d.	--
5	rt, Et_2O as the solvent	40%	88%
6	K_2CO_3 as the base	25%	85%
7	5 equiv *t*BuOH was added	66%	90%
8	10 mol% $NiBr_2$•DME, DMA as the solvent, rt	23%	--
9	2 equiv alkyl iodide, heated at 50 °C	72%	88%
10	heated at 50 °C	75%	88%

Yield was determined by ^{1}H NMR analysis, using 0.1 mmol 1,3,5-trimethoxybenzene as internal standard. The ee value was determined by HPLC analysis on a chiral stationary phase.

Supplementary Table 14 Optimization of Internal Enol Esters

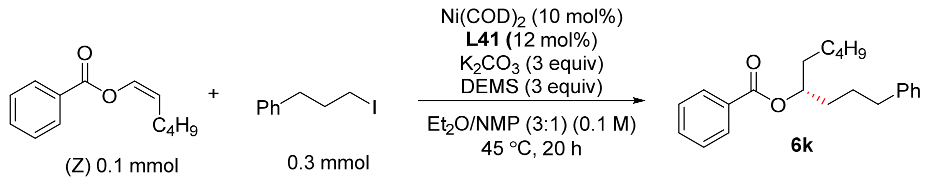

entry	Variation	yield of **6k**	ee
1	none	70	92
2	Et_2O/DMPU(3:1) as solvent	65	82
3	Et_2O/DMF(3:1) as solvent	60	94
4	Et_2O as solvent	trace	--
5	NMP as solvent	<20%	--
6	$K_3PO_4.H_2O$ as base, Et_2O/DMF(3:1) as solvent	44	98
7	KF as base	<10%	--
8	Na_2CO_3 as base	trace	--
9	4 equiv *t*-BuOH was added	61	94
10	4 equiv H_2O was added	60	95

Yield was determined by ^{1}H NMR analysis, using 0.1 mmol 1,3,5-trimethoxybenzene as internal standard. The ee value was determined by HPLC analysis on a chiral stationary phase.

Supplementary Note 2

Characterization of Products

(*R*)-1-(5-phenylpentan-2-yl)pyrrolidin-2-one (3a)

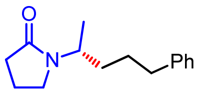

3a

Obtained as colorless oil (42.9 mg, 93% yield, 92% ee).

^{1}H NMR (600 MHz, $CDCl_3$) δ 7.28 – 7.26 (m, 2H), 7.19 – 7.15 (m, 3H), 4.27 – 4.22 (m, 1H), 3.28 – 3.25 (m, 1H), 3.17 – 3.13 (m, 1H), 2.68 – 2.64 (m, 1H), 2.60 – 2.55 (m, 1H), 2.38 (t, *J* = 8.4 Hz, 3H), 2.00 – 1.94 (m, 2H), 1.67 – 1.38 (m, 4H), 1.09 (d, *J* = 6.6 Hz, 3H).

^{13}C NMR (151 MHz, $CDCl_3$) δ 174.8, 142.3, 128.5, 128.4, 125.9, 46.4, 41.8, 35.6, 33.5, 31.7, 28.3, 18.3, 18.2.

HRMS (ESI-TOF) Calcd for $C_{15}H_{22}NO$ $(M+H)^+$ 232.1696. Found 232.1695.

HPLC (AD-H, 0.46*25 cm, 5 μm, hexane/isopropanol = 98/2, flow rate = 1 mL/min, detection at 210 nm) retention time = 11.2 min (major) and 13.5 min (minor).

(*R*)-1-(6-phenylhexan-2-yl)pyrrolidin-2-one (3b)

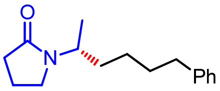

3b

Obtained as colorless oil (45.6 mg, 93% yield, 92% ee).

^{1}H NMR (600 MHz, $CDCl_3$) δ 7.29 – 7.21 (m, 2H), 7.18 – 7.09 (m, 3H), 4.31 – 4.10 (m, 1H), 3.33 – 3.23 (m, 1H), 3.21 – 3.14 (m, 1H), 2.57 (t, *J* = 7.8 Hz, 3H), 2.35 (t, *J* = 7.8 Hz, 3H), 1.87 – 1.98 (m, 2H), 1.72 – 1.51 (m, 2H), 1.49 – 1.39 (m, 2H), 1.33 – 1.17 (m, 2H), 1.08 (d, *J* = 6.6 Hz, 3H).

^{13}C NMR (151 MHz, $CDCl_3$) δ 174.7, 142.5, 128.3, 125.7, 46.5, 41.8, 35.8, 33.8, 31.6, 31.1, 26.0, 18.24, 18.17.

HRMS (ESI-TOF) Calcd for $C_{16}H_{24}NO$ $(M+H)^+$ 246.1852. Found 246.1852.

HPLC (AD-H, 0.46*25 cm, 5 μm, hexane/isopropanol = 99/1, flow rate = 1 mL/min, detection at 210 nm) retention time = 33.4 min (minor) and 37.5 min (major).

(*R*)-1-(4-phenylbutan-2-yl)pyrrolidin-2-one (3c)

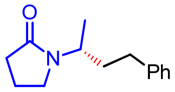

3c

Obtained as colorless oil (37.7 mg, 87% yield, 89% ee).

^{1}H NMR (400 MHz, $CDCl_3$) δ 7.28 – 7.20 (m, 2H), 7.18 – 7.10 (m, 3H), 4.31 – 4.22 (m, 1H), 3.30 – 3.25 (m, 1H), 3.22 – 3.16 (m, 1H), 2.63 – 2.55 (m, 1H), 2.53 – 2.43 (m, 1H), 2.34 (t, J = 7.6 Hz, 3H), 2.00 – 1.61 (m, 4H), 1.11 (d, J = 6.8 Hz, 3H).

^{13}C NMR (101 MHz, $CDCl_3$) δ 174.84, 141.82, 128.5, 128.4, 126.0, 46.8, 41.9, 35.8, 33.2, 31.7, 18.4, 18.2.

HRMS (ESI-TOF) Calcd for $C_{14}H_{20}NO$ $(M+H)^+$ 218.1539. Found 218.1539.

HPLC (AD-H, 0.46*25 cm, 5 μm, hexane/isopropanol = 98/2, flow rate = 1 mL/min, detection at 254 nm) retention time = 11.9 min (major) and 13.1 min (minor).

(*R*)-1-(1-cyclohexylpropan-2-yl)pyrrolidin-2-one (3d)

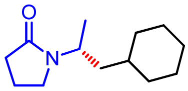

3d

Obtained as colorless oil (24.2 mg, 58% yield, 89% ee).

^{1}H NMR (400 MHz, $CDCl_3$) δ 4.42 – 4.24 (m, 1H), 3.31 – 3.20 (m, 2H), 2.44 – 2.30 (m, 2H), 2.05 – 1.91 (m, 2H), 1.89 – 1.78 (m, 1H), 1.68 – 1.60 (m, 4H), 1.42 – 1.35 (m, 1H), 1.26 – 1.12 (m, 5H), 1.07 (d, J = 6.8 Hz, 3H), 1.01 – 0.74 (m, 2H).

^{13}C NMR (151 MHz, $CDCl_3$) δ 174.6, 44.0, 41.81, 41.75, 34.7, 33.8, 33.0, 31.8, 26.6, 26.4, 26.3, 18.6, 18.3.

HRMS (ESI-TOF) Calcd for $C_{13}H_{24}NO$ $(M+H)^+$ 210.1852. Found 210.1853.

HPLC (AD-H, 0.46*25 cm, 5 μm, hexane/isopropanol = 99/1, flow rate = 1 mL/min, detection at 210 nm) retention time = 17.6 min (major) and 19.9 min (minor).

(*R*)-1-(5-(9*H*-carbazol-9-yl)pentan-2-yl)pyrrolidin-2-one (3e)

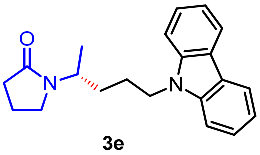

3e

Obtained as white solid (58.9 mg, 92% yield, 91% ee).

^{1}H NMR (400 MHz, $CDCl_3$) δ 7.94 (d, *J* = 7.6 Hz, 2H), 7.34 – 7.21 (m, 4H), 7.12 – 7.01 (m, 2H), 4.25 – 4.09 (m, 3H), 2.99 – 2.94 (m, 2H), 2.74 – 2.68 (m, 2H), 2.24 – 2.11 (m, 2H), 1.77 – 1.54 (m, 4H), 1.30 (q, *J* = 7.6 Hz, 2H), 0.88 (d, *J* = 6.8 Hz, 3H).

^{13}C NMR (101 MHz, $CDCl_3$) δ 175.0, 140.5, 125.8, 123.0, 120.4, 118.9, 108.8, 45.9, 42.5, 41.5, 31.6, 31.1, 25.6, 18.4, 18.0.

HRMS (ESI-TOF) Calcd for $C_{17}H_{23}N_2O$ $(M+H)^+$ 321.1961. Found 321.1961.

HPLC (AD-H, 0.46*25 cm, 5 µm, hexane/isopropanol = 95/5, flow rate = 1 mL/min, detection at 254 nm) retention time = 20.9 min (major) and 28.6 min (minor).

(*R*)-1-(5-(1*H*-indol-1-yl)pentan-2-yl)pyrrolidin-2-one (3f)

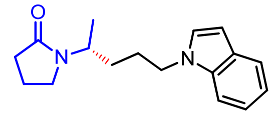

3f

Obtained as colorless oil (50.7 mg, 94% yield, 91% ee).

^{1}H NMR (600 MHz, $CDCl_3$) δ 7.62 (d, *J* = 7.8 Hz, 1H), 7.34 (d, *J* = 8.4 Hz, 1H), 7.22 – 7.17 (m, 1H), 7.10 – 7.08 (m, 2H), 6.48 (d, *J* = 3.0 Hz, 1H), 4.29 – 4.23 (m, 1H), 4.21 – 4.17 (m, 1H), 4.13 – 4.08 (m, 1H), 3.20 – 3.12 (m, 1H), 2.96 – 2.91 (m, 1H), 2.39 – 2.29 (m, 2H), 1.96 – 1.87 (m, 1H), 1.84 – 1.76 (m, 3H), 1.43 – 1.38 (m, 2H), 1.07 (d, *J* = 6.8 Hz, 3H).

^{13}C NMR (151 MHz, $CDCl_3$) δ 175.0, 136.0, 128.8, 128.0, 121.6, 121.1, 119.4, 109.5, 101.2, 45.94, 45.91, 41.6, 31.6, 31.0, 27.0, 18.4, 18.1.

HRMS (ESI-TOF) Calcd for $C_{17}H_{23}N_2O$ $(M+H)^+$ 271.1805. Found 271.1803.

HPLC (AD-H, 0.46*25 cm, 5 µm, hexane/isopropanol = 90/10, flow rate = 1 mL/min, detection at 210 nm) retention time = 8.9 min (major) and 10.5 min (minor).

(*R*)-1-(5-(thiophen-2-yl)pentan-2-yl)pyrrolidin-2-one (3g)

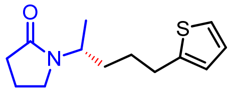

3g

Obtained as colorless oil (30.3 mg, 64% yield, 91% ee).

^{1}H NMR (400 MHz, $CDCl_3$) δ 7.10 (dd, *J* = 5.2, 1.6 Hz, 1H), 6.90 – 6.89 (m, 1H), 6.77 – 6.76 (m, 1H), 4.28 – 4.19 (m, 1H), 3.30 – 3.23 (m, 1H), 3.20 – 3.15 (m, 1H), 2.90 – 2.76 (m, 2H), 2.41 – 2.37 (m, 2H), 2.00 – 1.93 (m, 3H), 1.70 – 1.41 (m, 4H), 1.10 (d, *J*

= 6.8 Hz, 3H).

^{13}C NMR (101 MHz, $CDCl_3$) δ 174.9, 145.1, 126.8, 124.4, 123.1, 46.4, 41.9, 33.2, 31.6, 29.6, 28.6, 18.3, 18.2.

HRMS (ESI-TOF) Calcd for $C_{13}H_{20}NOS$ $(M+H)^+$ 238.1260. Found 238.1260.

HPLC (AD-H, 0.46*25 cm, 5 μm, hexane/isopropanol = 90/10, flow rate = 1 mL/min, detection at 254 nm) retention time = 10.3 min (major) and 12.3 min (minor).

(*R*)-2-(4-(2-oxopyrrolidin-1-yl)pentyl)isoindoline-1,3-dione (3h)

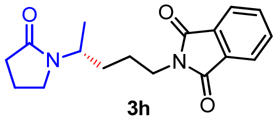

3h

Obtained as colorless oil. (49.8 mg, 83% yield, 91% ee)

^{1}H NMR (400 MHz, $CDCl_3$) δ 7.85 – 7.80 (m, 2H), 7.73 – 7.68 (m, 2H), 4.29 – 4.18 (m, 1H), 3.67 (t, *J* = 6.8 Hz, 2H), 3.32 – 3.18 (m, 2H), 2.40 – 2.35 (m, 2H), 2.06 – 1.93 (m, 2H), 1.69 – 1.45 (m, 4H), 1.11 (d, *J* = 6.8 Hz, 3H).

^{13}C NMR (151 MHz, $CDCl_3$) δ 174.8, 168.4, 134.0, 132.2, 123.3, 46.3, 41.8, 37.8, 31.6, 31.2, 25.8, 18.3, 18.2.

HRMS (ESI-TOF) Calcd for $C_{17}H_{21}N_2O_3$ $(M+H)^+$ 301.1547. Found 301.1546.

HPLC (AS-H, 0.46*25 cm, 5 μm, hexane/isopropanol = 80/20, flow rate = 1 mL/min, detection at 210 nm) retention time = 34.3 min (major) and 12.3 min (minor).

(*R*)-ethyl 5-(2-oxopyrrolidin-1-yl)hexanoate (3i)

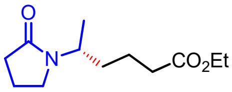

3i

Obtained as colorless oil (37.8 mg, 83% yield, 89% ee).

^{1}H NMR (400 MHz, $CDCl_3$) δ 4.26 – 4.15 (m, 1H), 4.10 (q, *J* = 8.0 Hz, 2H), 3.33 – 3.22 (m, 2H), 2.45 – 2.18 (m, 4H), 2.04 – 1.93 (m, 2H), 1.60 – 1.39 (m, 3H), 1.24 (t, *J* = 7.2 Hz, 3H), 1.11 (d, *J* = 6.8 Hz, 3H).

^{13}C NMR (151 MHz, $CDCl_3$) δ 174.9, 173.6, 60.4, 46.4, 41.8, 33.8, 33.4, 31.7, 21.8, 18.3, 18.2, 14.3.

HRMS (ESI-TOF) Calcd for $C_{13}H_{24}NO_3$ $(M+H)^+$ 228.1594. Found 228.1594.

HPLC (AD-H, 0.46*25 cm, 5 μm, hexane/isopropanol = 98/2, flow rate = 1 mL/min,

detection at 210 nm) retention time = 15.6 min (major) and 18.6 min (minor).

(*R*)-1-(6-methoxyhexan-2-yl)pyrrolidin-2-one (3j)

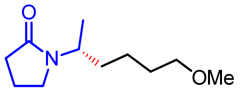

3j

Obtained as colorless oil (25.9 mg, 65% yield, 92% ee).

^{1}H NMR (400 MHz, $CDCl_3$) δ 4.22 – 4.13 (m, 1H), 3.38 – 3.20 (m, 7H), 2.37 (t, *J* = 8.0 Hz, 2H), 2.01 – 1.93 (m, 2H), 1.63 – 1.36 (m, 4H), 1.33 – 1.20 (m, 2H), 1.09 (d, *J* = 6.8 Hz, 3H).

^{13}C NMR (151 MHz, $CDCl_3$) δ 174.8, 72.7, 58.6, 46.6, 41.9, 31.7, 29.4, 23.2, 18.2, 18.2.

HRMS (ESI-TOF) Calcd for $C_{11}H_{22}NO_2$ $(M+H)^+$ 200.1645. Found 200.1645.

HPLC (AD-H, 0.46*25 cm, 5 µm, hexane/isopropanol = 98/2, flow rate = 1 mL/min, detection at 210 nm) retention time = 11.4 min (major) and 13.2 min(minor).

(*R*)-1-(4-phenoxybutan-2-yl)pyrrolidin-2-one (3k)

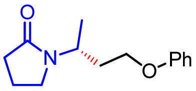

3k

Obtained as colorless oil (26.1 mg, 56% yield, 89% ee).

^{1}H NMR (600 MHz, $CDCl_3$) δ 7.22 – 7.16 (m, 2H), 6.87 – 6.85 (m, 1H), 6.80 (d, *J* = 8.0 Hz, 2H), 4.37 – 4.32 (m, 1H), 3.92 – 3.89 (m, 1H), 3.86 – 3.81 (m, 1H), 3.33 – 3.24 (m, 2H), 2.30 (t, *J* = 6.0 Hz, 1H), 2.00 – 1.85 (m, 4H), 1.14 (d, *J* = 6.8 Hz, 3H).

^{13}C NMR (151 MHz, $CDCl_3$) δ 174.9, 158.8, 129.6, 120.9, 114.6, 65.5, 44.8, 42.4, 33.6, 31.7, 18.4, 18.3.

HRMS (ESI-TOF) Calcd for $C_{14}H_{20}NO_2$ $(M+H)^+$ 234.1489. Found 234.1488.

HPLC (AD-H, 0.46*25 cm, 5 µm, hexane/isopropanol = 99/1, flow rate = 1 mL/min, detection at 254 nm) retention time = 41.1 min (major) and 44.8 min (minor).

(*R*)-1-(7-((*tert*-butyldiphenylsilyl)oxy)heptan-2-yl)pyrrolidin-2-one (3l)

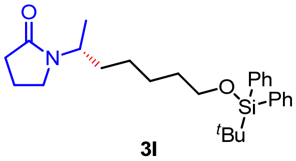

3l

Obtained as colorless oil (74.3 mg, 85% yield, 74% ee).

^{1}H NMR (400 MHz, $CDCl_3$) δ 7.67 – 7.65 (m, 4H), 7.45 – 7.32 (m, 6H), 4.21 – 4.12 (m, 1H), 3.64 (t, J = 6.8 Hz, 2H), 3.34 – 3.18 (m, 2H), 2.38 (t, J = 8.0 Hz, 2H), 2.03 – 1.87 (m, 3H), 1.57 – 1.51 (m, 2H), 1.45 – 1.28 (m, 4H), 1.29 – 1.15 (m, 2H), 1.09 (d, J = 6.8 Hz, 3H), 1.04 (s, 9H).

^{13}C NMR (101 MHz, $CDCl_3$) δ 174.7, 135.6, 134.2, 129.6, 127.7, 63.9, 46.8, 41.9, 34.1, 32.5, 31.7, 27.0, 26.3, 25.7, 19.3, 18.24, 18.21.

HRMS (ESI-TOF) Calcd for $C_{27}H_{40}NO_2Si$ $(M+H)^+$ 438.2823. Found 438.2823.

HPLC (AD-H, 0.46*25 cm, 5 µm, hexane/isopropanol = 99/1, flow rate = 1 mL/min, detection at 254 nm) retention time = 13.3 min (major) and 14.8 min (minor).

(*R*)-1-(4-(3-chlorophenyl)butan-2-yl)pyrrolidin-2-one (3m)

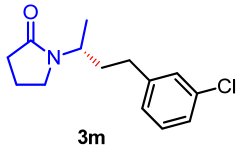

3m

Obtained as colorless oil (47.7 mg, 95% yield, 88% ee).

^{1}H NMR (400 MHz, $CDCl_3$) δ 7.21 – 7.14 (m, 3H), 7.06-7.03 (m, 1H), 4.35 – 4.21 (m, 1H), 3.34 – 3.28(m, 1H), 3.24 – 3.18 (m, 1H), 2.63 – 2.55 (m, 1H), 2.52 – 2.45 (m, 1H), 2.43 – 2.33 (m, 2H), 2.05 – 1.84 (m, 2H), 1.84 – 1.67 (m, 2H), 1.14 (d, J = 6.8 Hz, 3H).

^{13}C NMR (101 MHz, $CDCl_3$) δ 174.9, 143.8, 134.2, 129.7, 128.4, 126.7, 126.2, 46.7, 41.9, 35.5, 32.8, 31.6, 18.4, 18.1.

HRMS (ESI-TOF) Calcd for $C_{14}H_{19}ClNO$ $(M+H)^+$ 252.1150. Found 252.1150.

HPLC (OJ-H, 0.46*25 cm, 5 µm, hexane/isopropanol = 95/5, flow rate = 1 mL/min, detection at 254 nm) retention time = 11.4 min (major) and 15.6 min (minor).

(*R*)-1-(1-phenylpropan-2-yl)pyrrolidin-2-one (3n)

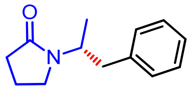

3n

Obtained as colorless oil (36.7 mg, 88% yield, 61% ee).

^{1}H NMR (400 MHz, $CDCl_3$) δ 7.27 – 7.20 (m, 2H), 7.19 – 7.12 (m, 3H), 4.51 – 4.42 (M, 1H), 3.30 – 3.17 (m, 2H), 2.82 – 2.68 (m, 2H), 2.35 – 2.15 (m, 2H), 1.97 – 1.78

(m, 2H), 1.12 (d, J = 6.8 Hz, 3H).

^{13}C NMR (101 MHz, $CDCl_3$) δ 174.6, 138.4, 129.1, 128.5, 126.5, 47.9, 42.7, 40.5, 31.6, 18.3, 17.6.

HRMS (ESI-TOF) Calcd for $C_{13}H_{18}NO$ $(M+H)^+$ 204.1383. Found 204.1383.

HPLC (OJ-H, 0.46*25 cm, 5 μm, hexane/isopropanol = 98/2, flow rate = 1 mL/min, detection at 210 nm) retention time = 13.1 min (major) and 14.9 min (minor).

(*R*)-1-(6-(9*H*-carbazol-9-yl)hexan-3-yl)pyrrolidin-2-one (3o)

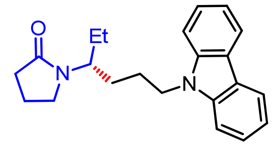

3o

Obtained as white solid (49.4 mg, 74% yield, 90% ee from (*E*) acyl enamine).

Obtained as white solid (53.4 mg, 80% yield, 81% ee from (*Z*) acyl enamine).

^{1}H NMR (600 MHz, $CDCl_3$) δ 8.09 (d, J = 9.6 Hz, 2H), 7.47 – 7.45 (m, 2H), 7.42 (d, J = 7.8 Hz, 2H), 7.24 – 7.21 (m, 2H), 4.41 – 4.36 (m, 1H), 4.32 – 4.28 (m, 1H), 4.09 – 4.04 (m, 1H), 3.05 – 3.01 (m, 1H), 2.82 – 2.78 (m, 1H), 2.42 – 2.31 (m, 2H), 1.92 – 1.70 (m, 4H), 1.54 – 1.47 (m, 1H), 1.43 – 1.31 (m, 3H), 0.78 (t, J = 7.2 Hz, 3H).

^{13}C NMR (151 MHz, $CDCl_3$) δ 175.8, 140.5, 125.8, 122.9, 120.4, 118.9, 108.9, 51.9, 42.5, 41.4, 31.6, 29.3, 25.7, 25.4, 18.2, 10.9.

HRMS (ESI-TOF) Calcd for $C_{22}H_{27}N_2O$ $(M+H)^+$ 335.2118. Found 335.2114.

HPLC (AD-H, 0.46*25 cm, 5 μm, hexane/isopropanol = 95/5, flow rate = 1 mL/min, detection at 210 nm) retention time = 18.5 min (major) and 28.3 min (minor). (from (*E*) acyl enamine).

HPLC (AD-H, 0.46*25 cm, 5 μm, hexane/isopropanol = 95/5, flow rate = 1 mL/min, detection at 210 nm) retention time = 18.5 min (major) and 28.0 min (minor). (from (*Z*) acyl enamine).

(*R*)-1-(1-(9*H*-carbazol-9-yl)nonan-4-yl)pyrrolidin-2-one (3p)

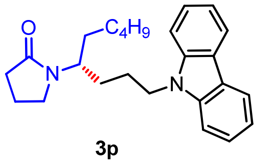

3p

Obtained as colorless oil (57.1 mg, 76% yield, 88% ee). (from (*E*) acyl enamine).

^{1}H NMR (600 MHz, $CDCl_3$) δ 8.10 (d, *J* = 7.6 Hz, 2H), 7.50 – 7.40 (m, 4H), 7.3-7.21 (m, 2H), 4.40 – 4.35 (m, 1H), 4.33 – 4.28 (m, 1H), 4.17 – 4.11 (m, 1H), 3.05 – 3.01 (m, 1H), 2.82 – 2.78 (m, 1H), 2.42 – 2.28 (m, 2H), 1.92 – 1.70 (m, 4H), 1.53 – 1.36 (m, 2H), 1.35 – 1.29 (m, 2H), 1.28 – 1.14 (m, 6H), 0.85 (t, *J* = 4.6 Hz, 3H).

^{13}C NMR (151 MHz, $CDCl_3$) δ 175.6, 140.5, 125.8, 122.9, 120.4, 118.9, 108.9, 50.4, 42.5, 41.5, 32.7, 31.9, 31.6, 29.6, 29.4, 29.3, 26.3, 25.4, 22.8, 18.2, 14.2.

HRMS (ESI-TOF) Calcd for $C_{25}H_{33}N_2O$ $(M+H)^+$ 377.2587. Found 377.2577.

HPLC (AD-H, 0.46*25 cm, 5 µm, hexane/isopropanol = 90/10, flow rate = 1 mL/min, detection at 210 nm) retention time = 8.7 min (major) and 12.3 min (minor).

(*R*)-1-(1-(9*H*-carbazol-9-yl)undecan-4-yl)pyrrolidin-2-one (3q)

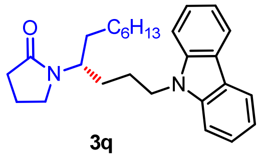

3q

Obtained as colorless oil (63.2 mg, 78% yield, 88% ee). (from (*E*) acyl enamine).

^{1}H NMR (400 MHz, $CDCl_3$) δ 8.10 (d, *J* = 7.8Hz, 2H), 7.49 – 7.39 (m, 4H), 7.24 – 7.20 (m, 2H), 4.43 – 4.24 (m, 2H), 4.19 – 4.09 (m, 1H), 3.06 – 3.00 (m, 1H), 2.83 – 2.77 (m, 1H), 2.45 – 2.26 (m, 2H), 1.93 – 1.70 (m, 4H), 1.56 – 1.01 (m, 14H), 0.86 (t, *J* = 6.8 Hz, 3H).

^{13}C NMR (151 MHz, $CDCl_3$) δ 175.6, 140.5, 125.8, 123.0, 120.5, 118.9, 108.9, 50.4, 42.5, 41.5, 32.7, 31.9, 31.6, 29.6, 29.4, 29.3, 26.3, 25.4, 22.8, 18.2, 14.2.

HRMS (ESI-TOF) Calcd for $C_{27}H_{37}N_2O$ $(M+H)^+$ 405.2900. Found 405.2889.

HPLC (AD-H, 0.46*25 cm, 5 µm, hexane/isopropanol = 90/10, flow rate = 1 mL/min, detection at 210 nm) retention time = 7.9 min (major) and 12.8 min (minor).

(*R*)-1-(6-(9*H*-carbazol-9-yl)-1-phenylhexan-3-yl)pyrrolidin-2-one (3r)

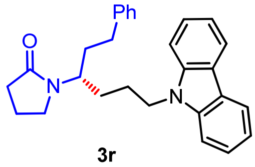

3r

Obtained as white solid (55.8 mg, 68% yield, 89% ee). (from (*E*) acyl enamine).

^{1}H NMR (600 MHz, $CDCl_3$) δ 8.09 (d, *J* = 7.8 Hz, 2H), 7.45 (m, 2H), 7.41 – 7.39 (m, 2H), 7.31 – 7.20 (m, 4H), 7.16 (t, *J* = 7.4 Hz, 1H), 7.11 (d, *J* = 6.7 Hz, 1H), 4.41 –

4.27 (m, 2H), 4.27 – 7.21 (m, 1), 3.02 – 2.98 (m, 1H), 2.81 – 2.77 (m, 1H), 2.56 – 2.51 (m, 1H), 2.46 – 2.41 (m, 1H), 2.35 – 2.31(m, 2H), 1.92 – 1.62 (m, 6H), 1.53 – 1.39 (m, 2H).

^{13}C NMR (151 MHz, $CDCl_3$) δ 175.7, 141.6, 140.5, 128.5, 128.3, 126.0, 125.8, 122.9, 120.4, 118.9, 108.8, 50.4, 42.3, 41.5, 34.3, 32.9, 31.5, 29.6, 25.2, 18.0.

HRMS (ESI-TOF) Calcd for $C_{28}H_{31}N_2O$ $(M+H)^+$ 411.2431. Found 411.2418.

HPLC (AD-H, 0.46*25 cm, 5 µm, hexane/isopropanol = 90/10, flow rate = 1 mL/min, detection at 210 nm) retention time = 16.6 min (major) and 26.4 min (minor).

(*R*)-1-(6-(5-bromo-1*H*-indol-1-yl)hexan-3-yl)pyrrolidin-2-one (3s)

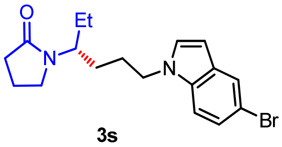

3s

Obtained as colorless oil (44.9 mg, 63% yield, 92% ee). (from (*E*) acyl enamine).

^{1}H NMR (400 MHz, $CDCl_3$) δ 7.73 (d, *J* = 1.1 Hz, 1H), 7.30 – 7.19 (m, 2H), 7.08 (d, *J* = 3.2 Hz, 1H), 6.41 (D, *J* = 3.2 Hz, 1H), 4.21 – 4.15 (M, 1H), 4.11 – 3.99 (m, 2H), 3.13 – 3.07 (m, 1H), 2.93 – 2.87 (m, 1H), 2.45 – 2.34 (m, 2H), 2.02 – 1.82 (m, 2H), 1.80 – 1.66 (m, 2H), 1.49 – 1.30 (m, 4H), 0.80 (t, *J* = 7.4 Hz, 3H).

^{13}C NMR (151 MHz, $CDCl_3$) δ 175.8, 134.7, 130.4, 129.1, 124.4, 123.5, 112.7, 111.0, 100.8, 51.7, 45.9, 41.5, 31.5, 29.1, 26.8, 25.6, 18.3, 10.9.

HRMS (ESI-TOF) Calcd for $C_{18}H_{24}BrN_2O$ $(M+H)^+$ 363.1067. Found 363.1055.

HPLC (AD-H, 0.46*25 cm, 5 µm, hexane/isopropanol = 90/10, flow rate = 1 mL/min, detection at 210 nm) retention time = 9.7 min (major) and 11.5 min (minor).

(*R*)-N-(5-phenylpentan-2-yl)benzamide (4a)

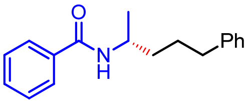

4a

Obtained as white solid (44.8 mg, 84% yield, 93% ee).

^{1}H NMR (400 MHz, $CDCl_3$) δ 7.70 (d, *J* = 6.8 Hz, 2H), 7.50 – 7.43 (m, 1H), 7.39 – 7.36 (m 2H), 7.28 – 7.21 (m, 2H), 7.16 – 7.13 (m, 3H), 5.82 (d, *J* = 8.4 Hz, 1H), 4.26 – 4.16 (m, 1H), 2.67 – 2.56 (m, 2H), 1.73 – 1.65 (m, 1H), 1.56 – 1.53 (m, 1H), 1.20 (d, *J*

= 6.8 Hz, 3H).

^{13}C NMR (151 MHz, $CDCl_3$) δ 167.0, 142.3, 135.1, 131.4, 128.7, 128.6, 128.5, 126.94, 125.93, 45.7, 36.7, 35.8, 28.0, 21.2.

HPLC (OJ-H, 0.46*25 cm, 5 μm, hexane/isopropanol = 90/10, flow rate = 1 mL/min, detection at 254 nm) retention time = 15.7 min (major) and 18.5 min (minor).

(*R*)-4-(*tert*-butyl)-N-(5-phenylpentan-2-yl)benzamide (4b)

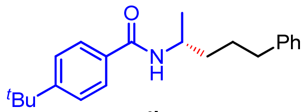

4b

Obtained as white solid (52.9 mg, 82% yield, 93% ee).

^{1}H NMR (600 MHz, $CDCl_3$) δ 7.51 (d, *J* = 8.4 Hz, 2H), 7.27 (d, *J* = 8.4 Hz, 2H), 7.12 – 7.09 (m, 2H), 7.06 – 6.98 (m, 3H), 5.69 (d, *J* = 8.4 Hz, 1H), 4.11 – 4.04 (m, 1H), 2.52 – 2.44 (m, 2H), 1.61 – 1.51 (m, 2H), 1.44 – 1.37 (m, 2H), 1.17 (s, 9H), 1.05 (d, *J* = 6.8 Hz, 2H).

^{13}C NMR (101 MHz, $CDCl_3$) δ 166.9, 154.9, 142.3, 132.2, 128.5, 128.4, 126.8, 125.9, 125.6, 45.6, 36.7, 35.8, 35.0, 31.3, 28.0, 21.2.

HRMS (ESI-TOF) Calcd for $C_{22}H_{30}NO$ $(M+H)^+$ 324.2322. Found 324.2321.

HPLC (AD-H, 0.46*25 cm, 5 μm, hexane/isopropanol = 95/5, flow rate = 1 mL/min, detection at 210 nm) retention time = 18.0 min (major) and 23.4 min (minor).

(*R*)-4-methoxy-N-(5-phenylpentan-2-yl)benzamide (4c)

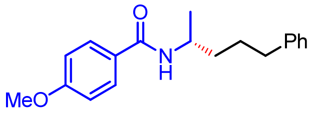

4c

Obtained as white solid. (53.4 mg, 90% yield, 94% ee)

^{1}H NMR (600 MHz, $CDCl_3$) δ 7.68 (d, *J* = 8.4 Hz, 2H), 7.26 – 7.23 (m, 2H), 7.19 – 7.12 (m, 3H), 6.88 (d, *J* = 8.4 Hz, 2H), 5.81 (d, *J* = 7.8 Hz, 1H), 4.23 – 4.17 (m, 1H), 3.82 (s, 3H), 2.66 – 2.58 (m, 2H), 1.71 – 1.66 (m, 1H), 1.58 – 1.52 (m, 1H), 1.19 (d, *J* = 6.6 Hz, 3H).

^{13}C NMR (151 MHz, $CDCl_3$) δ 166.5, 162.2, 142.3, 128.7, 128.6, 128.4, 127.3, 125.9, 113.8, 55.5, 45.6, 36.8, 35.8, 28.0, 21.3.

HRMS (ESI-TOF) Calcd for $C_{19}H_{24}NO_2$ $(M+H)^+$ 298.1802. Found 298.1801.

HPLC (AD-H, 0.46*25 cm, 5 µm, hexane/isopropanol = 90/10, flow rate = 1 mL/min, detection at 254 nm) retention time = 14.5 min (major) and 16.7 min (minor).

(*R*)-3-methyl-N-(5-phenylpentan-2-yl)benzamide (4d)

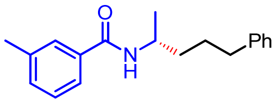

4d

Obtained as white solid (48.9 mg, 87% yield, 94% ee).

^{1}H NMR (400 MHz, $CDCl_3$) δ 7.48 (s, 1H), 7.44 – 7.42 (m, 1H), 7.25 – 7.16 (m, 4H), 7.14 – 7.07 (m, 3H), 5.78 (d, *J* = 8.4 Hz, 1H), 4.23 – 4.10 (m, 1H), 2.61 – 2.52 (m, 2H), 2.32 (s, 3H), 1.68 – 1.61 (m, 2H), 1.55 – 1.44 (m, 2H), 1.15 (d, *J* = 6.6 Hz, 3H).

^{13}C NMR (101 MHz, $CDCl_3$) δ 167.2, 142.3, 138.5, 135.0, 132.1, 128.5, 128.5, 128.4, 127.7, 125.9, 123.9, 45.6, 36.7, 35.8, 28.0, 21.4, 21.2.

HRMS (ESI-TOF) Calcd for $C_{19}H_{24}NO$ $(M+H)^+$ 282.1852. Found 282.1852.

HPLC (OJ-H, 0.46*25 cm, 5 µm, hexane/isopropanol = 95/5, flow rate = 1 mL/min, detection at 210 nm) retention time = 25.2 min (major) and 27.8 min (minor).

(*R*)-3,5-dimethyl-N-(5-phenylpentan-2-yl)benzamide (4e)

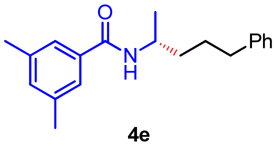

4e

Obtained as white solid (46.6 mg, 79% yield, 95% ee).

^{1}H NMR (400 MHz, $CDCl_3$) δ 7.25 (s, 2H), 7.28 – 7.17 (m, 2H), 7.13 – 7.08 (m, 3H), 7.04 (s, 1H), 5.72 (d, *J* = 8.4 Hz, 1H), 4.20 – 4.13 (m, 1H), 2.64 – 2.52 (m, 2H), 2.28 (s, 6H), 1.71 – 1.60 (m, 2H), 1.53 – 1.44 (m, 2H), 1.15 (d, *J* = 6.6 Hz, 3H).

^{13}C NMR (101 MHz, $CDCl_3$) δ 167.3, 142.3, 138.3, 135.1, 133.0, 128.6, 128.4, 125.9, 124.7, 45.6, 36.7, 35.8, 28.0, 21.3, 21.2.

HRMS (ESI-TOF) Calcd for $C_{20}H_{26}NO$ $(M+H)^+$ 296.2009. Found 296.2009.

HPLC (AS-H, 0.46*25 cm, 5 µm, hexane/isopropanol = 90/10, flow rate = 1 mL/min, detection at 210 nm) retention time =12.8 min (major) and 24.2 min (minor).

(*R*)-2-methyl-N-(5-phenylpentan-2-yl)benzamide (4f)

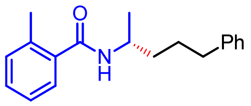

4f

Obtained as white solid (38.2 mg, 68% yield, 90% ee).

^{1}H NMR (600 MHz, $CDCl_3$) δ 7.26 – 7.22 (m, 4H), 7.19 – 7.11 (m, 6H), 5.49 (d, *J* = 8.4 Hz, 1H), 4.21 – 4.14 (m, 1H), 2.68 – 2.55 (m, 2H), 2.38 (s, 3H), 1.73 – 1.65 (m, 2H), 1.55 – 1.44 (m, 2H), 1.18 (d, *J* = 6.6 Hz, 3H).

^{13}C NMR (101 MHz, $CDCl_3$) δ 169.63, 142.23, 137.07, 135.84, 131.00, 129.76, 128.52, 128.44, 126.61, 125.91, 125.80, 45.44, 36.61, 35.75, 28.05, 21.22, 19.79.

HRMS (ESI-TOF) Calcd for $C_{19}H_{24}NO$ $(M+H)^+$ 282.1852. Found 282.1852.

HPLC (AS-H, 0.46*25 cm, 5 μm, hexane/isopropanol = 90/10, flow rate = 1 mL/min, detection at 210 nm) retention time = 15.4 min (major) and 20.4 min (minor).

(*R*)-N-(5-phenylpentan-2-yl)-4-(trifluoromethyl)benzamide (4g)

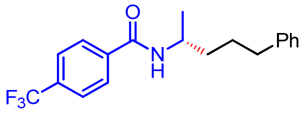

4g

Obtained as white solid (58.9 mg, 88% yield, 95% ee).

^{1}H NMR (600 MHz, $CDCl_3$) δ 7.80 (d, *J* = 8.4 Hz, 2H), 7.66 (d, *J* = 8.4 Hz, 2H), 7.26 – 7.21 (m, 2H), 7.18 – 7.11 (m, 3H), 5.83 (d, *J* = 8.4 Hz, 1H), 4.27 – 4.15 (m, 1H), 2.67 – 2.59 (m, 2H), 1.71 – 1.67 (m, 2H), 1.58 – 1.55 (m, 2H), 1.22 (d, *J* = 6.6 Hz, 3H).

^{13}C NMR (151 MHz, $CDCl_3$) δ 165.7, 142.2, 138.4, 133.2 (q, *J* = 37.7 Hz), 128.6, 128.5, 127.4, 126.0, 125.7 (q, *J* = 4.5 Hz), 123.8 (q, *J* = 273.3 Hz), 46.1, 36.6, 35.8, 28.0, 21.1.

^{19}F NMR (564 MHz, $CDCl_3$) δ -62.92 (s, 3F).

HRMS (ESI-TOF) Calcd for $C_{19}H_{21}F_3NO$ $(M+H)^+$ 336.1570. Found 336.1569.

HPLC (OJ-H, 0.46*25 cm, 5 μm, hexane/isopropanol = 95/5, flow rate = 1 mL/min, detection at 210 nm) retention time = 23.1 min (major) and 29.8 min (minor).

(*R*)-4-cyano-N-(5-phenylpentan-2-yl)benzamide (4h)

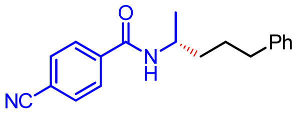

4h

Obtained as white solid (51.9 mg, 89% yield, 94% ee).

^{1}H NMR (600 MHz, $CDCl_3$) δ 7.79 (d, *J* = 8.4 Hz, 2H), 7.69 (d, *J* = 8.4 Hz, 2H), 7.28 – 7.22 (m, 2H), 7.19 – 7.11 (m, 3H), 5.89 (d, *J* = 8.4 Hz, 1H), 4.22 – 4.18 (m, 1H), 2.66 – 2.58 (m, 2H), 1.70 – 1.66 (m, 2H), 1.58 – 1.54 (m, 2H), 1.21 (d, *J* = 6.6 Hz, 3H).

^{13}C NMR (151 MHz, $CDCl_3$) δ 165.2, 142.1, 139.0, 132.5, 128.53, 128.52, 127.7, 126.0, 118.2, 115.0, 46.2, 36.5, 35.7, 28.0, 21.1.

HRMS (ESI-TOF) Calcd for $C_{19}H_{20}N_2O$ $(M+H)^+$ 293.1648. Found 293.1649.

HPLC (AD-H, 0.46*25 cm, 5 μm, hexane/isopropanol = 90/10, flow rate = 1 mL/min, detection at 210 nm) retention time = 14.9 min (major) and 18.5 min (minor).

(*R*)-ethyl 4-((5-phenylpentan-2-yl)carbamoyl)benzoate (4i)

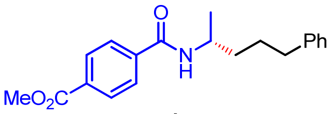

4i

Obtained as white solid (50.3 mg, 74% yield, 96% ee).

^{1}H NMR (400 MHz, $CDCl_3$) δ 8.05 (d, *J* = 8.4 Hz, 2H), 7.75 (d, *J* = 8.4 Hz, 2H), 7.28 – 7.21 (m, 2H), 7.18 – 7.11 (m, 3H), 5.85 (d, *J* = 8.4 Hz, 1H), 4.36 – 4.16 (m, 1H), 3.91 (s, 3H), 2.66 – 2.58 (m, 2H), 1.73 – 1.65 (m, 2H), 1.58 – 1.52 (m, 2H), 1.21 (d, *J* = 6.6 Hz, 3H).

^{13}C NMR (101 MHz, $CDCl_3$) δ 166.4, 166.1, 142.2, 139.0, 132.7, 129.9, 128.53, 128.48, 127.0, 126.0, 52.5, 46.0, 36.6, 35.7, 28.0, 21.1.

HRMS (ESI-TOF) Calcd for $C_{20}H_{24}NO_3$ $(M+H)^+$ 326.1756. Found 326.1756.

HPLC (AD-H, 0.46*25 cm, 5 μm, hexane/isopropanol = 95/5, flow rate = 1 mL/min, detection at 210 nm) retention time = 25.6 min (major) and 30.1 min (minor).

(*R*)-2-fluoro-N-(5-phenylpentan-2-yl)benzamide (4j)

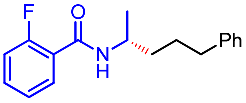

4j

Obtained as white solid (46.7 mg, 82% yield, 93% ee).

¹H NMR (400 MHz, $CDCl_3$) δ 7.38– 7.33 (m, 2H), 7.31 – 7.26 (m, 1H), 7.23 – 7.14 (m, 2H), 7.11 – 7.04 (m, 3H), 5.72 (d, *J* = 8.4 Hz, 1H), 4.17 – 4.07 (m, 1H), 2.60 – 2.45 (m, 2H), 1.65 – 1.56 (m, 2H), 1.50 – 1.42 (m, 2H), 1.13 (d, *J* = 6.6 Hz, 3H).

¹³C NMR (101 MHz, $CDCl_3$) δ 165.7 (d, J = 2.5 Hz), 162.9 (d, J = 247.7 Hz), 142.2, 137.4 (d, J = 6.6 Hz), 130.3 (d, J = 7.9 Hz), 128.54, 128.48, 126.0, 122.4 (d, J = 3.0 Hz), 118.4 (d, J = 21.3 Hz), 114.4 (d, J = 22.8 Hz), 45.9, 36.6, 35.8, 28.0, 21.1.

¹⁹F NMR (376 MHz, $CDCl_3$) δ -104.79 – -114.80 (m).

HRMS (ESI-TOF) Calcd for $C_{18}H_{21}FNO$ $(M+H)^+$ 286.1602. Found 286.1601.

HPLC (AS-H, 0.46*25 cm, 5 μm, hexane/isopropanol = 90/10, flow rate = 1 mL/min, detection at 210 nm) retention time = 13.4 min (major) and 19.9 min (minor).

(*R*)-N-(5-phenylpentan-2-yl)-2-naphthamide (4k)

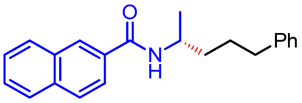

4k

Obtained as white solid (55.8 mg, 88% yield, 95% ee).

¹H NMR (600 MHz, $CDCl_3$) δ 8.16 (s, 1H), 7.85 – 7.79 (m, 3H), 7.73 (dd, *J* = 8.4, 1.8 Hz, 1H), 7.50 – 7.44 (m, 2H), 7.23 – 7.17 (m, 2H), 7.13 – 7.08 (m, 3H), 5.94 (d, *J* = 8.4 Hz, 1H), 4.27 – 4.19 (m, 1H), 2.64 – 2.56 (m, 2H), 1.71 – 1.66 (m, 2H), 1.60 – 1.51 (m, 2H), 1.20 (d, *J* = 6.6 Hz, 3H).

¹³C NMR (101 MHz, $CDCl_3$) δ 167.1, 142.3, 134.8, 132.7, 132.3, 129.0, 128.6, 128.5, 128.45, 127.8, 127.6, 127.3, 126.8, 125.9, 123.7, 45.9, 36.7, 35.8, 28.1, 21.2.

HRMS (ESI-TOF) Calcd for $C_{22}H_{23}NO$ $(M+H)^+$ 318.1852. Found 318.1852

HPLC (AD-H, 0.46*25 cm, 5 μm, hexane/isopropanol = 95/5, flow rate = 1 mL/min, detection at 210 nm) retention time = 36.3 min (major) and 45.7 min (minor).

(*R*)-N-(5-phenylpentan-2-yl)-1-naphthamide (4l)

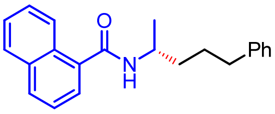

4l

Obtained as white solid (43.1 mg, 68% yield, 93% ee).

¹H NMR (600 MHz, $CDCl_3$) δ 8.11 (d, *J* = 8.4 Hz, 1H), 7.77 – 7.67 (m, 2H), 7.41 –

7.33 (m, 3H), 7.28 (t, *J* = 7.2 Hz, 1H), 7.14 – 7.10 (m, 2H), 7.05 – 7.02 (m, 3H), 5.61 (d, *J* = 9.0 Hz, 1H), 4.23 – 4.16 (m, 1H), 2.58 – 2.49 (m, 2H), 1.65 – 1.59 (m, 2H), 1.46 – 1.43 (m, 2H), 1.13 (d, *J* = 6.6 Hz, 3H).

^{13}C NMR (101 MHz, $CDCl_3$) δ 169.1, 142.2, 135.1, 133.8, 130.4, 130.2, 128.54, 128.46, 128.4, 127.2, 126.5, 125.9, 125.5, 124.8, 124.7, 45.7, 36.6, 35.8, 28.1, 21.3.

HRMS (ESI-TOF) Calcd for $C_{22}H_{24}NO$ $(M+H)^+$ 318.1852. Found 318.1853

HPLC (AD-H, 0.46*25 cm, 5 μm, hexane/isopropanol = 95/5, flow rate = 1 mL/min, detection at 210 nm) retention time = 22.6 min (major) and 28.5 min (minor).

(*R*)-N-(5-phenylpentan-2-yl)furan-2-carboxamide (4m)

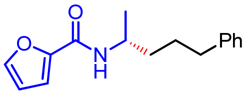

4m

Obtained as white solid (41.6 mg, 81% yield, 95% ee).

^{1}H NMR (600 MHz, $CDCl_3$) δ 7.34 (d, *J* = 1.2 Hz, 1H), 7.22 – 7.18 (m, 2H), 7.12 – 7.08 (m, 3H), 7.02 (d, *J* = 3.6 Hz, 1H), 6.41 (q, *J* = 1.8 Hz, 1H), 6.03 (d, *J* = 8.4 Hz, 1H), 4.17 – 4.08 (m, 1H), 2.63 – 2.50 (m, 2H), 1.69 – 1.58 (m, 2H), 1.54 – 1.44 (m, 2H), 1.14 (d, *J* = 6.6 Hz, 3H).

^{13}C NMR (101 MHz, $CDCl_3$) δ 157.9, 148.3, 143.7, 142.2, 128.5, 128.4, 125.9, 114.1, 112.2, 45.0, 36.6, 35.7, 27.9, 21.2.

HRMS (ESI-TOF) Calcd for $C_{16}H_{20}NO_2$ $(M+H)^+$ 258.1489. Found 258.1489.

HPLC (AD-H, 0.46*25 cm, 5 μm, hexane/isopropanol = 99/1, flow rate = 1 mL/min, detection at 254 nm) retention time = 34.6 min (major) and 42.5 min (minor).

(*R*)-N-(5-phenylpentan-2-yl)thiophene-2-carboxamide (4n)

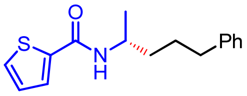

4n

Obtained as white solid (37.1 mg, 68% yield, 95% ee).

^{1}H NMR (600 MHz, $CDCl_3$) δ 7.43 (d, *J* = 4.8 Hz, 2H), 7.26 – 7.23 (m, 2H), 7.17 – 7.13 (m, 3H), 7.04 (t, *J* = 4.2 Hz, 1H), 5.67 (d, *J* = 7.8 Hz, 1H), 4.21 – 4.14 (m, 1H), 2.66 – 2.58 (m, 2H), 1.73 – 1.65 (m, 2H), 1.57 – 1.48 (m, 2H), 1.20 (d, *J* = 6.6 Hz, 3H).

^{13}C NMR (151 MHz, $CDCl_3$) δ 161.3, 142.2, 139.5, 129.8, 128.6, 128.5, 127.84, 127.7, 125.9, 45.8, 36.6, 35.8, 28.0, 21.2.

HRMS (ESI-TOF) Calcd for $C_{16}H_{20}NOS$ $(M+H)^+$ 274.1260. Found 274.1260.

HPLC (OJ-H, 0.46*25 cm, 5 µm, hexane/isopropanol = 90/10, flow rate = 1 mL/min, detection at 254 nm) retention time = 20.3 min (major) and 30.4 min (minor).

(*R*)-N-(5-phenylpentan-2-yl)thiophene-2-carboxamide (4o)

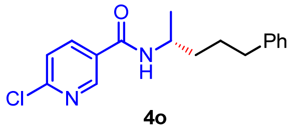

4o

Obtained as white solid (29.6 mg, 49% yield, 89% ee).

^{1}H NMR (400 MHz, $CDCl_3$) δ 8.63 (s, 1H), 7.97 (d, J = 8.4 Hz, 1H), 7.35 (s, 1H), 7.27 – 7.17 (m, 2H), 7.16 – 7.05 (m, 3H), 5.82 (d, J = 8.4 Hz, 1H), 4.21 – 4.10 (m, 1H), 2.63 – 2.51 (m, 2H), 1.71 – 1.42 (m, 4H), 1.17 (d, J = 6.8 Hz, 3H).

^{13}C NMR (101 MHz, $CDCl_3$) δ 164.0, 154.2, 147.9, 142.0, 138.1, 129.6, 128.5 (d, J = 1.3 Hz), 126.0, 124.4, 46.2, 36.4, 35.7, 28.0, 21.1.

HRMS (ESI-TOF) Calcd for $C_{17}H_{20}ClN_2O$ $(M+H)^+$ 303.1259. Found 303.1260

HPLC (AD-H, 0.46*25 cm, 5 µm, hexane/isopropanol = 95/5, flow rate = 1 mL/min, detection at 210 nm) retention time = 17.3 min (major) and 19.3 min (minor).

(*R*)-N-(5-phenylpentan-2-yl)acetamide (4p)

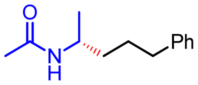

4p

Obtained as white solid (27.9 mg, 68% yield, 93% ee).

^{1}H NMR (400 MHz, $CDCl_3$) δ 7.27 – 7.20 (m, 2H), 7.16 – 7.12 (m, 3H), 5.15 (s, 1H), 4.06 – 3.87 (m, 1H), 2.65 – 2.50 (m, 2H), 1.91 (s, 3H), 1.67 – 1.56 (m, 3H), 1.44 – 1.39 (m, 2H), 1.08 (d, J = 6.8 Hz, 3H).

^{13}C NMR (151 MHz, $CDCl_3$) δ 169.6, 142.3, 128.5, 128.4, 125.9, 45.4, 36.5, 35.8, 28.0, 23.6, 21.1.

HRMS (ESI-TOF) Calcd for $C_{13}H_{20}NO$ $(M+H)^+$ 206.1539. Found 206.1540.

HPLC (OJ-H, 0.46*25 cm, 5 µm, hexane/isopropanol = 95/5, flow rate = 1 mL/min, detection at 210 nm) retention time = 17.9 min (major) and 30.0 min (minor).

(*R*)-N-(5-phenylpentan-2-yl)butyramide (4q)

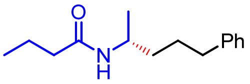

4q

Obtained as white solid (23.7 mg, 51% yield, 93% ee).

^{1}H NMR (400 MHz, $CDCl_3$) δ 7.27 – 7.21 (m, 2H), 7.17 – 7.11 (m, 3H), 5.25 (d, *J* = 8.4 Hz, 1H), 4.07 – 3.90 (m, 1H), 2.64 – 2.52 (m, 2H), 2.08 (t, *J* = 7.2 Hz, 2H), 1.69 – 1.54 (m, 4H), 1.46 – 1.36 (m, 2H), 1.08 (d, *J* = 6.8 Hz, 3H), 0.91 (t, *J* = 7.6 Hz, 3H).

^{13}C NMR (101 MHz, $CDCl_3$) δ 172.4, 142.3, 128.5, 128.4, 125.9, 45.0, 39.1, 36.6, 35.8, 28.0, 21.2, 19.4, 13.8.

HRMS (ESI-TOF) Calcd for Chemical Formula: $C_{15}H_{24}NO$ $(M+H)^+$ 234.1852. Found 234.1853.

HPLC (OJ-H, 0.46*25 cm, 5 µm, hexane/isopropanol = 95/5, flow rate = 1 mL/min, detection at 210 nm) retention time = 10.8 min (major) and 12.3 min (minor).

(*R*)-N-(5-phenylpentan-2-yl)isobutyramide (4r)

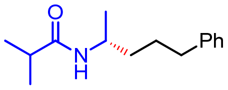

4r

Obtained as white solid (31.7 mg, 68% yield, 90% ee).

^{1}H NMR (600 MHz, $CDCl_3$) δ 7.27 – 7.22 (m, 2H), 7.18 – 7.10 (m, 3H), 5.13 (d, *J* = 8.4 Hz, 1H), 4.03 – 3.96 (m, 1H), 2.62 – 2.56 (m, 2H), 2.28 – 2.34 (m, 1H), 1.64 – 1.59 (m, 2H), 1.46 – 1.38 (m, 2H), 1.11 (dd, *J* = 6.6, 2.4 Hz, 6H), 1.08 (d, *J* = 6.8 Hz, 3H).

^{13}C NMR (151 MHz, $CDCl_3$) δ 176.4, 142.4, 128.5 (d, *J* = 13.1 Hz), 125.9, 44.8, 36.7, 35.9, 35.8, 28.0, 21.2, 19.9, 19.7.

HRMS (ESI-TOF) Calcd for $C_{15}H_{24}NO$ $(M+H)^+$ 234.1852. Found 234.1853.

HPLC (AS-H, 0.46*25 cm, 5 µm, hexane/isopropanol = 98/2, flow rate = 1 mL/min, detection at 210 nm) retention time = 11.9 min (major) and 13.8 min (minor).

(*R*)-N-(5-phenylpentan-2-yl)cyclopropanecarboxamide (4s)

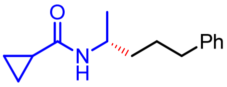

4s

Obtained as white solid (40.6 mg, 88% yield, 96% ee).

^{1}H NMR (600 MHz, $CDCl_3$) δ 7.26 – 7.25 (m, 2H), 7.18 – 7.12 (m, 3H), 5.41 (d, *J* = 7.8 Hz, 1H), 4.04 – 4.00 (m, 1H), 2.63 – 2.55 (m, 2H), 1.67 – 1.59 (m, 2H), 1.47 – 1.39 (m, 2H), 1.26 – 1.22 (m, 1H), 1.09(d, *J* = 6.8 Hz, 3H), 0.93 – 0.90 (m, 2H), 0.69 – 0.66 (m, 2 H)

^{13}C NMR (151 MHz, $CDCl_3$) δ 172.9, 142.4, 128.5, 128.4, 125.9, 45.3, 36.8, 35.8, 28.0, 21.2, 15.0, 7.11, 7.06.

HRMS (ESI-TOF) Calcd for $C_{15}H_{22}NO$ $(M+H)^+$ 232.1696. Found 232.1697.

HPLC (OJ-H, 0.46*25 cm, 5 μm, hexane/isopropanol = 95/5, flow rate = 1 mL/min, detection at 210 nm) retention time = 16.2 min (major) and 22.5 min (minor).

(*R*)-N-(5-phenylpentan-2-yl)cyclohexanecarboxamide (4t)

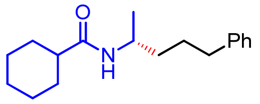

4t

Obtained as white solid (39.8 mg, 73% yield, 93% ee).

^{1}H NMR (600 MHz, $CDCl_3$) δ 7.27 – 7.23 (m, 2H), 7.18 – 7.11 (m, 3H), 5.14 (2, *J* = 7.8 Hz, 1H), 4.04 – 3.93 (m, 1H), 2.64 – 2.53 (m, 2H), 2.01 – 1.96 (m, 1H), 1.81 – 1.74 (m, 4H), 1.64 – 1.58 (m, 3H), 1.47 – 1.35 (m, 4H), 1.29 – 1.15 (m, 3H), 1.06 (d, *J* = 8.4 Hz , 3H).

^{13}C NMR (151 MHz, $CDCl_3$) δ 175.5, 142.4, 128.5, 128.4, 125.9, 45.9, 44.7, 36.7, 35.8, 30.0, 29.8, 28.0, 25.9, 25.9, 25.9, 21.2.

HRMS (ESI-TOF) Calcd for $C_{18}H_{28}NO$ $(M+H)^+$ 274.2165. Found 274.2164.

HPLC (OJ-H, 0.46*25 cm, 5 μm, hexane/isopropanol = 98/2, flow rate = 1 mL/min, detection at 210 nm) retention time = 11.1 min (major) and 15.5 min (minor).

(*R*)-N-(5-phenylpentan-2-yl)pivalamide (4u)

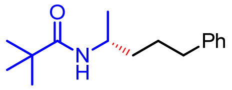

4u

Obtained as white solid (41.5 mg, 84% yield, 92% ee).

^{1}H NMR (600 MHz, $CDCl_3$) δ 7.27 – 7.23 (m, 2H), 7.17 – 7.11 (m, 3H), 5.31 (d, *J* =

7.8 Hz, 1H), 4.02 – 3.95 (m, 1H), 2.63 – 2.55 (m, 2H), 1.64 – 1.58 (m, 2H), 1.47 – 1.38 (m, 2H), 1.15 (s, 9H), 1.08 (d, *J* = 6.8 Hz, 3H).

^{13}C NMR (151 MHz, $CDCl_3$) δ 177.8, 142.4, 128.5, 128.4, 125.9, 44.8, 38.7, 36.6, 35.7, 28.0, 27.7, 21.1.

HRMS (ESI-TOF) Calcd for $C_{16}H_{26}NO$ $(M+H)^+$ 248.2009. Found 248.2008.

HPLC (OD-H, 0.46*25 cm, 5 μm, hexane/isopropanol = 99/1, flow rate = 0.5 mL/min, detection at 210 nm) retention time = 20.3min (major) and 21.8 min (minor).

(*R*)-N-methyl-N-(5-phenylpentan-2-yl)acetamide (4v)

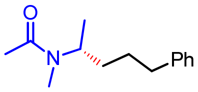

4v

Obtained as white solid (34.6 mg, 79% yield, 80% ee).

^{1}H NMR (400 MHz, $CDCl_3$) δ 7.28 – 7.20 (m, 2H), 7.18 – 7.07 (m, 3H), 4.79 – 4.70 (M, 0.5H), 3.81 – 3.68 (m, 0.5H), 2.69 (s, 1.5H), 2.66 (s, 1.5H), 2.63 – 2.48 (M, 2H), 2.04 (d, *J* = 7.0 Hz, 3H), 1.56 – 1.35 (m, 4H), 1.10 (d, *J* = 6.6 Hz, 1.5H), 1.00 (d, *J* = 6.8 Hz, 1.5H).

^{13}C NMR (101 MHz, $CDCl_3$) δ 170.8, 170.5, 142.3, 141.8, 128.5, 128.4, 128.4, 126.1, 125.8, 53.4, 47.6, 35.7, 35.6, 33.9, 33.2, 29.1, 28.4, 28.3, 25.8, 22.5, 21.8, 19.0, 18.0.

HRMS (ESI-TOF) Calcd for $C_{14}H_{22}NO$ $(M+H)^+$ 220.1696. Found 220.1696.

HPLC (OB-H, 0.46*25 cm, 5 μm, hexane/isopropanol = 98/2, flow rate = 1 mL/min, detection at 210 nm) retention time = 19.0 min (minor) and 24.2 min (major).

(*R*)-N-(4-(2,3-dihydrobenzofuran-5-yl)butan-2-yl)benzamide (5a)

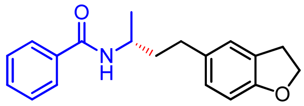

5a

Obtained as white solid (49.5 mg, 84% yield, 96% ee).

^{1}H NMR (600 MHz, $CDCl_3$) δ 7.66 (d, *J* = 6.0 Hz, 2H), 7.49 – 7.47 (m, 1H), 7.42 – 7.40 (m, 2H), 7.03 (s, 1H), 6.93 (d, *J* = 1.8 Hz, 1H), 6.70 (d, *J* = 8.4 Hz, 1H), 5.86 (d, *J* = 8.4 Hz, 1H), 4.55 – 4.46 (m, 2H), 4.29 – 4.25 (m, 1H), 3.17 – 3.05 (m, 2H), 2.71 –

2.62 (m, 2H), 1.86 (q, *J* = 7.8 Hz, 2H), 1.28 (d, *J* = 6.6 Hz, 3H).

^{13}C NMR (151 MHz, $CDCl_3$) δ 166.9, 158.5, 135.0, 133.8, 131.4, 128.6, 127.8, 127.3, 126.9, 125.1, 109.2, 71.3, 45.9, 39.1, 32.0, 29.9, 21.2.

HRMS (ESI-TOF) Calcd for $C_{19}H_{22}NO_2$ $(M+H)^+$ 296.1645. Found 296.1645.

HPLC (AS-H, 0.46*25 cm, 5 μm, hexane/isopropanol = 80/20, flow rate = 1 mL/min, detection at 210 nm) retention time = 16.2 min (major) and 19.7 min (minor).

(*R*)-N-(*sec*-butyl)benzamide (5b)

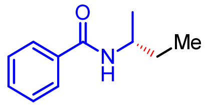

5b

Obtained as white solid (23.0 mg, 65% yield, 94% ee).

^{1}H NMR (400 MHz, $CDCl_3$) δ 7.75 (d, *J* = 8.0 Hz, 2H), 7.52 – 7.39 (m, 3H), 5.86 (s, 1H), 4.19 – 4.08 (M, 1H), 1.62-1.56 (m, 2H), 1.23 (d, *J* = 6.8 Hz, 3H), 0.97 (t, *J* = 8.0 Hz, 3H).

^{13}C NMR (101 MHz, $CDCl_3$) δ 167.1, 135.2, 131.4, 128.6, 126.9, 47.2, 29.9, 20.6, 10.5.

HPLC (AS-H, 0.46*25 cm, 5 μm, hexane/isopropanol = 90/10, flow rate = 1 mL/min, detection at 210 nm) retention time = 15.9 min (major) and 21.6 min (minor).

(*R*)-N-(hexan-2-yl)benzamide (5c)

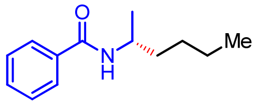

5c

Obtained as white solid (25.8 mg, 63% yield, 93% ee).

^{1}H NMR (400 MHz, $CDCl_3$) δ 7.62 (d, *J* = 8.0 Hz, 2H), 7.37 – 7.34 (m, 1H), 7.31 – 7.27 (m, 2H), 5.20 (d, *J* = 8.0 Hz ,1H), 4.1 – 4.01 (m, 1H), 1.44 – 1.38 (m, 2H), 1.25 – 1.19 (m, 4H), 1.10 (d, *J* = 6.8 Hz, 3H), 0.77 (t, *J* = 8.0 Hz, 3H).

^{13}C NMR (101 MHz, $CDCl_3$) δ 167.0, 135.2, 131.4, 128.6, 126.9, 45.9, 36.9, 28.4, 22.7, 21.2, 14.2.

HPLC (AD-H, 0.46*25 cm, 5 μm, hexane/isopropanol = 99/1, flow rate = 1 mL/min, detection at 210 nm) retention time = 30.2 min (major) and 34..3 min (minor).

(*R*)-N-(decan-2-yl)benzamide (5d)

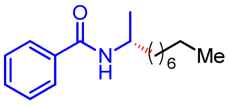

5d

Obtained as white solid (39.3 mg, 75% yield, 93% ee).

^{1}H NMR (400 MHz, $CDCl_3$) δ 7.75 (d, J = 8.0 Hz, 2H), 7.51 – 7.47 (m, 1H), 7.44 – 7.41 (m, 2H), 5.85 (d, J = 8.0 Hz, 1H), 4.24 – 4.14 (m, 1H), 1.57 – 1.51 (m, 2H), 1.42 – 1.19 (m, 14H), 0.87 (t, J = 8.0 Hz, 3H).

^{13}C NMR (101 MHz, $CDCl_3$) δ 167.0, 135.2, 131.4, 128.6, 126.9, 45.9, 37.2, 32.0, 29.67, 29.65, 29.4, 26.2, 22.8, 21.2, 14.2.

HPLC (AD-H, 0.46*25 cm, 5 μm, hexane/isopropanol = 95/5, flow rate = 1 mL/min, detection at 210 nm) retention time = 14.0 (major) and 18.0 min (minor).

(*R*)-N-(5,5,5-trifluoropentan-2-yl)benzamide (5e)

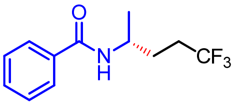

5e

Obtained as white solid (39.2 mg, 80% yield, 98% ee).

^{1}H NMR (600 MHz, $CDCl_3$) δ 7.75 (d, J = 6.0 Hz, 2H), 7.53 – 7.50 (m, 1H), 7.46 – 7.43 (m, 2H), 5.90 (d, J = 6.0 Hz, 1H), 4.32 – 4.25 (m, 1H), 2.26 – 2.15 (m, 2H), 1.88 – 1.82 (m, 1H), 1.80 – 1.75 (m, 1H), 1.30 (d, J = 6.0 Hz, 3H).

^{13}C NMR (101 MHz, $CDCl_3$) δ 167.3, 134.5, 131.8, 128.8, 127.1 (q, J = 413.7 Hz), 127.0, 44.9, 31.2 (q, J = 29.1 Hz), 29.7 (d, J = 2.9 Hz), 21.2.

^{19}F NMR (565 MHz, $CDCl_3$) δ -66.47 (t, J = 10.7 Hz).

HRMS (ESI-TOF) Calcd for $C_{12}H_{15}F_3NO$ $(M+H)^+$ 246.1100. Found 246.1100.

HPLC (OJ-H, 0.46*25 cm, 5 μm, hexane/isopropanol = 98/2, flow rate = 1 mL/min, detection at 210 nm) retention time = 15.1min (major) and 16.8 min (minor).

(*R*)-N-(8-hydroxyoctan-2-yl)benzamide (5f)

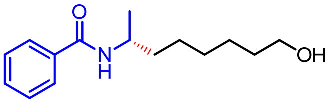

5f

Obtained as colorless oil (32.3mg, 65% yield, 92% ee).

^{1}H NMR (400 MHz, $CDCl_3$) δ 7.74 (d, J = 8.0 Hz, 2H), 7.51 – 7.47 (m, 1H), 7.44 –

7.40 (m, 2H), 5.89 (d, *J* = 8.0 Hz, 1H), 4.23 – 4.16 (m, 1H), 3.63 (t, *J* = 8.0 Hz, 2H), 1.59 – 1.51 (m, 4H), 1.43 – 1.31 (m, 6H), 1.23 (d, *J* = 8.0 Hz, 3H).

^{13}C NMR (101 MHz, $CDCl_3$) δ 167.0, 135.1, 131.4, 128.7, 126.9, 63.0, 45.8, 37.1, 32.7, 29.3, 26.1, 25.7, 21.2.

HRMS (ESI-TOF) Calcd for $C_{15}H_{24}NO_2$ $(M+H)^+$ 250.1802. Found 250.1801.

HPLC (AS-H, 0.46*25 cm, 5 μm, hexane/isopropanol = 80/20, flow rate = 1 mL/min, detection at 254 nm) retention time = 10.2 min (major) and 13.9 min (minor).

(*R*)-N-(8-((*tert*-butyldimethylsilyl)oxy)octan-2-yl)benzamide (5g)

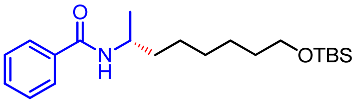

5g

Obtained as colorless oil (45.0 mg, 62% yield, 94% ee).

^{1}H NMR (400 MHz, $CDCl_3$) δ 7.74 (d, *J* = 6.0 Hz, 2H), 7.49 – 7.46 m, 1H), 7.43 – 7.39 (m, 2H), 5.91 (d, *J* = 8.4 Hz, 1H), 4.23 – 4 13 (m, 1H), 3.58 (t, *J* = 6.8 Hz, 2H), 1.56 – 1.46 (m, 4H), 1.43 – 1.29 (m, 5H), 1.22 (d, *J* = 6.8 Hz, 3H), 0.88 (s, 9H), 0.03 (s, 6H).

^{13}C NMR (101 MHz, $CDCl_3$) δ 167.0, 135.2, 131.4, 128.6, 126.9, 63.4, 45.9, 37.2, 32.9, 29.5, 26.2, 26.1, 25.9, 21.2, 18.5.

HRMS (ESI-TOF) Calcd for $C_{21}H_{38}NO_2Si$ $(M+H)^+$ 364.2666. Found 364.2665.

HPLC (AS-H, 0.46*25 cm, 5 μm, hexane/isopropanol = 95/5, flow rate = 1 mL/min, detection at 210 nm) retention time = 7.5 min (major) and 9.6 min (minor).

ethyl (*R*)-5-benzamidohexanoate (5h)

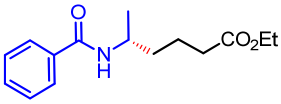

5h

Obtained as white solid (37.9 mg, 72% yield, 94% ee).

^{1}H NMR (400 MHz, $CDCl_3$) δ 7.76 (d, *J* = 6.8 Hz, 2H), 7.49 – 7.44 (m, 1H), 7.43 – 7.36 (m, 2H), 6.12 (d, *J* = 8.4 Hz, 1H), 4.24 – 4.19 (m, 1H), 4.11 (q, *J* = 7.2 Hz, 2H), 2.35 – 2.31 (m, 2H), 1.74 – 1.63 (m, 2H), 1.61 – 1.53 (m, 2H), 1.25 – 1.22 (m, 6H).

^{13}C NMR (101 MHz, $CDCl_3$) δ 173.7, 167.1, 135.0, 131.4, 128.6, 127.0, 60.5, 45.6, 36.3, 34.0, 21.4, 21.1, 14.3.

HRMS (ESI-TOF) Calcd for $C_{15}H_{22}NO_3$ $(M+H)^+$ 264.1594. Found 264.1592.

HPLC (AD-H, 0.46*25 cm, 5 μm, hexane/isopropanol = 95/5, flow rate = 1 mL/min, detection at 254 nm) retention time = 15.9 min (major) and 17.0 min (minor).

(*R*)-N-(1-(oxetan-3-yl)ethyl)benzamide (5i)

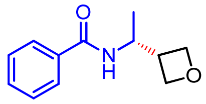

5i

Obtained as white solid (27.1 mg, 66% yield, 98% ee).

^{1}H NMR (600 MHz, $CDCl_3$) δ 7.76 (d, J = 9.6 Hz, 2H), 7.51 – 7.48 (m, 1H), 7.44 – 7.41 (m 2H), 6.17 (d, J = 8.4 Hz, 1H), 4.79 – 4.76 (m, 2H), 4.63 – 4.52 (m, 2H), 4.48 (t, J = 6.6 Hz, 1H), 3.15 – 3.09 (m, 1H), 1.19 (d, J = 7.2 Hz, 3H).

^{13}C NMR (151 MHz, $CDCl_3$) δ 167.6, 134.6, 131.7, 128.7, 127.0, 74.4, 74.3, 47.3, 40.9, 18.2.

HRMS (ESI-TOF) Calcd for $C_{12}H_{16}NO_2$ $(M+H)^+$ 206.1181. Found 206.1175.

HPLC (AD-H, 0.46*25 cm, 5 μm, hexane/isopropanol = 95/5, flow rate = 1 mL/min, detection at 210 nm) retention time = 18.3 min (minor) and 23.0 min (major).

(*R*)-4-(1-benzamidoethyl)piperidin-1-ylpivalate (5j)

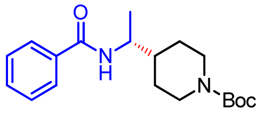

5j

Obtained as white solid (40.5mg, 61% yield, 92% ee).

^{1}H NMR (600 MHz, $CDCl_3$) δ 7.70 – 7.66 (m, 2H), 7.46 – 7.42 (m, 1H), 7.37 (t, J = 7.4 Hz, 2H), 5.84 (d, J = 8.8 Hz, 1H), 4.09 (q, J = 6.2, 5.8 Hz, 3H), 2.60 (tt, J = 13.1, 3.3 Hz, 2H), 1.65 (dd, J = 35.6, 13.2 Hz, 2H), 1.38 (s, 9H), 1.20 (td, J = 12.8, 4.5 Hz, 2H).

^{13}C NMR (101 MHz, $CDCl_3$) δ 167.1, 154.9, 134.9, 131.6, 128.7, 127.0, 79.6, 49.2, 44.0, 43.8, 41.8, 28.5, 28.4, 18.0.

HRMS (ESI-TOF) Calcd for $C_{19}H_{29}N_2O_3$ $(M+Na)^+$ 355.1992. Found 355.1991.

HPLC (OJ-H, 0.46*25 cm, 5 μm, hexane/isopropanol = 95/5, flow rate = 1 mL/min, detection at 254 nm) retention time = 16.3 min (major) and 23.5 min (minor).

(1*R*,2*S*,4*R*)-1,7,7-trimethylbicyclo[2.2.1]heptan-2-yl-(*R*)-7-benzamidooctanoate (5k)

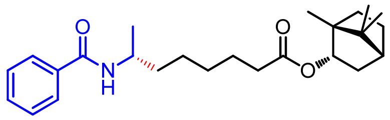

5k

Obtained as white solid (49.5 mg, 62% yield, 98:2 dr).

^{1}H NMR (600 MHz, $CDCl_3$) δ 7.75 (d, *J* = 7.2 Hz, 2H), 7.45 – 7.48 (m, 1H), 7.44 – 7.41 (m, 2H), 5.86 (d, *J* = 8.4 Hz, 1H), 4.89 – 4.86 (m, 1H), 4.24 – 4.15 (m, 1H), 2.37 – 2.29 (m, 3H), 1.96 – 1.89 (m, 1H), 1.76 – 1.70 (m, 2H), 1.68 – 1.60 (m, 3H), 1.58 – 1.51 (m, 2H), 1.43 – 1.35 (m, 4H), 1.31 – 1.18 (m, 6H), 0.94 (dd, *J* = 13.8, 3.6 Hz, 1H), 0.89 (s, 3H), 0.86 (s, 3H), 0.81 (s, 3H).

^{13}C NMR (151 MHz, $CDCl_3$) δ 174.2, 167.0, 135.1, 131.4, 128.7, 127.0, 79.8, 48.9, 45.8, 45.0, 29.2, 28.2, 27.2, 25.9, 25.1, 21.2, 19.8, 19.0, 13.6.

HRMS (ESI-TOF) Calcd for $C_{25}H_{38}NO_3$ $(M+H)^+$ 400.2846. Found 400.2846.

HPLC (AD-H, 0.46*25 cm, 5 μm, hexane/isopropanol = 95/5, flow rate = 1 mL/min, detection at 210 nm) retention time = 14.4 min (major) and 17.0 min (minor).

(1*R*,2*S*,5*R*)-2-isopropyl-5-methylcyclohexyl-(*R*)-6-benzamidoheptanoate (5l)

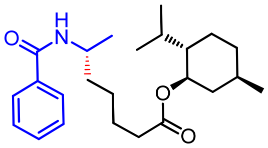

5l

Obtained as white solid (67.3mg, 87% yield, 97:3 dr).

^{1}H NMR (400 MHz, $CDCl_3$) δ 7.68 (d, *J* = 8.4 Hz, 1H), 7.42 (t, *J* = 7.2 Hz, 1H), 7.35 (t, *J* = 7.2 Hz, 2H), 5.88 (d, *J* = 6.8 Hz, 1H), 4.62 – 4.56 (m, 1H), 4.17 – 4.07 (m, 1H), 2.22 (t, *J* = 8.8 Hz, 2H), 1.88 – 1.24 (m, 14H), 1.16 (d, *J* = 6.4 Hz, 3H), 1.04 – 0.86 (m, 2H), 0.85 – 0.76 (m, 6H), 0.68 (d, *J* = 6.8 Hz, 2H).

^{13}C NMR (101 MHz, $CDCl_3$) δ 173.3, 167.0, 135.0, 131.4, 128.6, 126.9, 74.1, 47.1, 45.8, 41.0, 36.7, 34.6, 34.3, 31.4, 26.4, 25.7, 25.0, 23.5, 22.1, 21.1, 20.8, 16.4.

HRMS (ESI-TOF) Calcd for $C_{24}H_{38}NO_3$ $(M+H)^+$ 388.2852. Found 388.2843.

HPLC (OJ-H, 0.46*25 cm, 5 μm, hexane/isopropanol = 98/2, flow rate = 1 mL/min, detection at 210 nm) retention time = 8.4 min (minor) and 10.3 min (major).

(10*R*,13*R*,14*S*)-10,13-dimethyl-17-((*R*)-6-methylheptan-2-yl)-2,3,4,7,8,9,10,11,12,13,14,15,16,17-tetradecahydro-1H-cyclopenta[a]phenanthren-2-yl-(6*R*)-6-benzamidoheptanoate (5m)

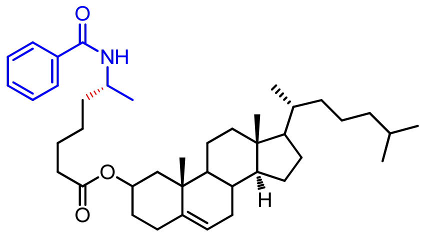

5m

Obtained as white solid (88.8 mg, 72% yield, 98:2 dr).

^{1}H NMR (400 MHz, $CDCl_3$) δ 7.75 (d, *J* = 6.8 Hz, 2H), 7.51 – 7.47 (m, 1H), 7.44 – 7.40 (m, 2H), 5.88 (d, *J* = 8.4 Hz, 1H), 5.32 (d, *J* = 5.2 Hz, 1H), 4.63 – 4.55 (m, 1H), 4.27 – 4.11 (m, 1H), 2.32 – 2.23 (m, 3H), 2.05 – 1.91 (m, 2H), 1.88 – 1.77 (m, 3H), 1.70 – 1.62 (m, 2H), 1.60 – 1.29 (m, 21H), 1.24 (d, *J* = 6.6 Hz, 3H), 1.16 – 1.07 (m, 6H), 1.00 (s, 3H), 0.91 (d, *J* = 6.8 Hz, 3H), 0.86 (dd, *J* = 6.8, 2.0 Hz, 6H).

^{13}C NMR (101 MHz, $CDCl_3$) δ 173.2, 139.7, 131.4, 128.6, 126.9, 122.7, 74.0, 56.8, 56.2, 50.1, 45.8, 42.4, 39.8, 39.6, 38.2, 37.1, 36.8, 36.7, 36.3, 35.9, 34.6, 32.01, 31.96, 28.4, 28.1, 27.9, 25.7, 25.0, 24.4, 24.0, 23.0, 22.7, 21.2, 21.1, 19.4, 18.8, 12.0.

HRMS (ESI-TOF) Calcd for $C_{41}H_{64}NO_3$ $(M+H)^+$ 618.4886. Found 618.4880.

HPLC (ASH, 0.46*25 cm, 5 μm, hexane/isopropanol = 95/5, flow rate = 1 mL/min, detection at 210 nm) retention time = 8.5 min (major) and 12.2 min (minor).

N-((*R*)-7-(((*S*)-2,5,7,8-tetramethyl-2-((4*S*,8*S*)-4,8,12-trimethyltridecyl)chroman-6-yl)oxy)heptan-2-yl)benzamide (5n)

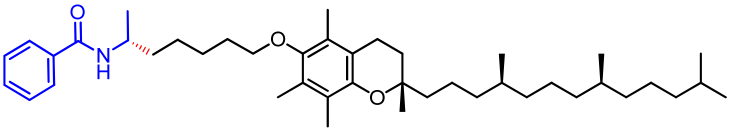

5n

Obtained as white solid (60.3 mg, 45% yield, 97:3 dr).

^{1}H NMR (600 MHz, $CDCl_3$) δ 7.75 (d, *J* = 7.2 Hz, 2H), 7.51 – 7.47 (m, 1H), 7.44 – 7.41 (m, 2H), 5.86 (d, *J* = 8.4 Hz, 1H), 4.24 – 4.20 (m, 1H), 2.60 – 2.57 (m, 4H), 2.08 (s, 3H), 1.99 (s, 3H), 1.95 (s, 3H), 1.85 – 1.70 (m, 6H), 1.61 – 1.34 (m,14H), 1.32 – 0.98 (m, 20H), 0.92 – 0.81 (m, 12H).

^{13}**C NMR** (151 MHz, $CDCl_3$) δ 172.4, 167.0, 149.5, 140.6, 135.1, 131.4, 128.7, 126.9, 126.8, 125.0, 123.1, 117.5, 75.2, 45.8, 39.5, 37.58, 37.55, 37.4, 37.1, 34.2, 32.9, 32.8, 29.4, 28.1, 26.0, 25.2, 24.9, 24.6, 22.9, 22.8, 20.7, 19.9, 19.8, 13.1, 12.3, 12.0.

HRMS (ESI-TOF) Calcd for $C_{42}H_{68}NO_3$ $(M+Na)^+$ 670.5170. Found 670.5123.

HPLC (AD-H, 0.46*25 cm, 5 μm, hexane/isopropanol = 98/2, flow rate = 0.7 mL/min, detection at 210 nm) retention time = 15.5 min (major) and 17.6 min (minor).

(*R*)-5-phenylpentan-2-ylbenzoate (6a)

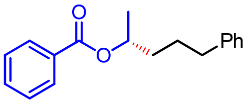

6a

Obtained as colorless oil (37.5 mg, 73% yield, 91% ee).

^{1}**H NMR** (400 MHz, $CDCl_3$) δ 8.04 – 7.94 (m, 2H), 7.56 – 7.48 (m, 1H), 7.42 – 7.38 (m, 2H), 7.30 – 7.19 (m, 2H), 7.16-7.31 (m, 3H), 5.22 – 5.10 (m, 1H), 2.62 (t, *J* = 6.8 Hz, 2H), 1.85 – 1.59 (m, 4H), 1.30 (d, *J* = 6.4 Hz, 3H).

^{13}**C NMR** (151 MHz, $CDCl_3$) δ 166.4, 142.2, 132.9, 131.0, 129.7, 128.5, 128.5, 128.4, 126.0, 71.6, 35.8, 35.7, 27.3, 20.2.

HPLC (OJ-H, 0.46*25 cm, 5 μm, hexane/isopropanol = 98/2, flow rate = 1 mL/min, detection at 210 nm) retention time = 8.2 min (minor) and 8.7 min (major).

(*R*)-5-phenylpentan-2-yl-2-naphthoate (6b)

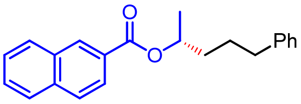

6b

Obtained as colorless oil (42.6 mg, 67% yield, 92% ee).

^{1}**H NMR** (400 MHz, $CDCl_3$) δ 8.55 (s, 1H), 8.03 (dd, *J* = 8.4, 1.6 Hz, 1H), 7.92 (d, *J* = 9.6 Hz, 1H), 7.84 (d, *J* = 8.4 Hz, 2H), 7.59 – 7.46 (m, 2H), 7.30 – 7.21 (m, 2H), 7.16 – 7.12 (m, 3H), 5.27 – 5.19 (m, 1H), 2.64 (t, *J* = 7.2 Hz, 2H), 1.87 – 1.62 (m, 4H), 1.35 (d, *J* = 6.6 Hz, 3H).

^{13}**C NMR** (101 MHz, $CDCl_3$) δ 166.5, 142.2, 135.6, 132.6, 131.0, 129.5, 128.54, 128.47, 128.3, 128.2, 127.9, 126.7, 126.0, 125.4, 71.7, 35.8 (d, *J* = 3.0 Hz), 27.4, 20.3.

HRMS (ESI-TOF) Calcd for $C_{22}H_{22}O_2Na$ $(M+Na)^+$ 341.1512. Found 341.1511.

HPLC (AD-H, 0.46*25 cm, 5 μm, hexane/isopropanol = 99/1, flow rate = 1 mL/min, detection at 254 nm) retention time = 11.9 min (major) and 14.2 min (minor).

(*R*)-N-(4-methylhexan-2-yl)benzamide (6c)

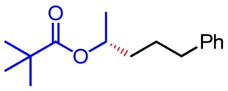

6c

Obtained as colorless oil (26.2 mg, 53% yield, 80% ee).

^{1}H NMR (400 MHz, $CDCl_3$) δ 7.27 – 7.20 (m, 2H), 7.18 – 7.10 (m, 3H), 4.91-4.83 (m, 1H), 2.68 – 2.52 (m, 2H), 1.70 – 1.45 (m, 4H), 1.20 – 1.06 (m, 11H).

^{13}C NMR (101 MHz, $CDCl_3$) δ 178.3, 142.3, 128.5, 128.4, 125.9, 70.4, 38.8, 35.8, 35.6, 27.3, 27.3, 20.0.

HRMS (ESI-TOF) Calcd for $C_{16}H_{24}O_2$ Na $(M+Na)^+$ 271.1669. Found 271.1677.

HPLC (OD-H, 0.46*25 cm, 5 μm, hexane/isopropanol = 99.8/0.2, flow rate = 1 mL/min, detection at 210 nm) retention time = 13.2 min (major) and 17.3 min (minor).

(*R*)-6-ethoxy-6-oxohexan-2-ylbenzoate (6d)

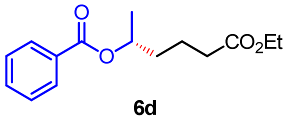

6d

Obtained as colorless oil (35.9mg, 68% yield, 93% ee).

^{1}H NMR (400 MHz, $CDCl_3$) δ 8.03 (d, *J* = 8.4 Hz, 1H), 7.56 – 7.52 (m, 1H), 7.45 – 7.41 (m, 2H), 5.20 – 5.13 (m, 1H), 4.11 (q, *J* = 7.2 Hz, 2H), 2.34 (t, *J* = 6.8 Hz, 2H), 1.84 – 1.61 (m, 4H), 1.35 (d, *J* = 6.6 Hz, 3H), 1.24 (t, *J* = 7.2 Hz, 3H).

^{13}C NMR (101 MHz, $CDCl_3$) δ 173.5, 166.3, 132.9, 130.8, 129.6, 128.4, 71.3, 60.4, 35.5, 34.1, 21.0, 20.1, 14.3.

HRMS (ESI-TOF) Calcd for $C_{15}H_{20}O_4$ Na $(M+Na)^+$ 287.1254. Found 287.1252.

HPLC (OJ-H, 0.46*25 cm, 5 μm, hexane/isopropanol = 98/2, flow rate = 1 mL/min, detection at 254 nm) retention time = 7.5 min (minor) and 7.8 min (major).

(*R*)-6-methoxyhexan-2-ylbenzoate (6e)

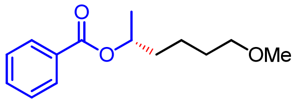

6e

Obtained as colorless oil (30.7 mg, 65% yield, 93% ee).

^{1}H NMR (600 MHz, $CDCl_3$) δ 8.04 (d, *J* = 8.4 Hz, 2H), 7.56 – 7.52 (m, 1H), 7.44 – 7.41 (m, 2H), 5.18 – 5.13 (m, 1H), 3.37 (t, *J* = 6.6 Hz, 2H), 3.31 (s, 2H), 1.81 – 1.72 (m, 1H), 1.68 – 1.58 (m, 2H), 1.53 – 1.38 (m, 1H), 1.34 (d, *J* = 6.0 Hz, 3H), 1.26 – 1.18 (m, 1H).

^{13}C NMR (151 MHz, $CDCl_3$) δ 166.3, 132.8, 131.0, 129.6, 128.4, 72.7, 71.7, 58.7, 36.0, 29.6, 22.2, 20.2.

HRMS (ESI-TOF) Calcd for $C_{14}H_{20}O_3$ Na $(M+Na)^+$ 259.1305. Found 259.1302.

HPLC (AS-H, 0.46*25 cm, 5 μm, hexane/isopropanol = 98/2, flow rate = 1 mL/min, detection at 210 nm) retention time = 4.0 min (major) and 4.5 min (minor).

(*R*)-5-(thiophen-2-yl)pentan-2-ylbenzoate (6f)

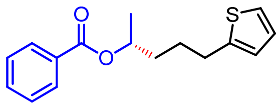

6f

Obtained as colorless oil (43.8 mg, 80% yield, 90% ee).

^{1}H NMR (600 MHz, $CDCl_3$) δ 8.04 (d, *J* = 6.6 Hz, 2H), 7.58 – 7.53 (m, 1H), 7.45 – 7.42 (m, 2H), 7.11 (d, *J* = 6.0 Hz, 1H), 6.91 (dd, *J* = 4.8, 3.0 Hz, 1H), 6.79 (d, *J* = 3.6 Hz, 1H), 5.23 – 5.16 (m, 1H), 2.88 (t, *J* = 7.2 Hz, 2H), 1.86 – 1.67 (m, 4H), 1.35 (d, *J* = 6.6 Hz, 3H).

^{13}C NMR (101 MHz, $CDCl_3$) δ 166.3, 145.0, 132.9, 130.9, 129.7, 128.4, 126.8, 124.4, 123.1, 71.4, 35.5, 29.8, 27.7, 20.2.

HRMS (ESI-TOF) Calcd for $C_{16}H_{18}O_2S$ Na $(M+Na)^+$ 297.0920. Found 297.0919.

HPLC (AD-H, 0.46*25 cm, 5 μm, hexane/isopropanol = 99/1, flow rate = 0.5 mL/min, detection at 210 nm) retention time = min10.2 (major) and 10.9 min (minor).

(*R*)-5-(1,3-dioxoisoindolin-2-yl)pentan-2-ylbenzoate (6g)

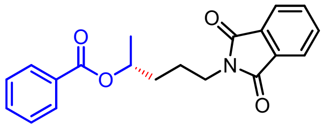

6g

Obtained as colorless oil (34.4 mg, 51% yield, 95% ee).

^{1}H NMR (600 MHz, $CDCl_3$) δ 8.01 (d, *J* = 6.6 Hz, 2H), 7.83 – 7.81 (m, 2H), 7.70 –

7.68 (m, 2H), 7.54 – 7.50 (m, 1H), 7.42 – 7.39 (m, 2H), 5.21 – 5.16 (m, 1H), 3.72 (t, *J* = 6.0 Hz, 2H), 1.83 – 1.75 (m, 3H), 1.72 – 1.67 (m, 1H), 1.34 (d, *J* = 6.0 Hz, 3H).

^{13}C NMR (151 MHz, $CDCl_3$) δ 168.5, 166.2, 134.0, 132.9, 132.2, 129.6, 128.4, 123.3, 71.1, 37.9, 33.4, 24.8, 20.1.

HRMS (ESI-TOF) Calcd for $C_{20}H_{20}NO_4$ $(M+H)^+$ 338.1387. Found 338.1385.

HPLC (AD-H, 0.46*25 cm, 5 μm, hexane/isopropanol = 98/2, flow rate = 1 mL/min, detection at 240 nm) retention time = 16.5 min (major) and 18.1 min (minor).

(*R*)-octan-2-ylbenzoate (6h)

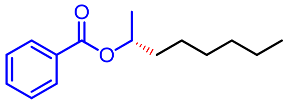

6h

Obtained as colorless oil (36.0 mg, 77% yield, 90% ee).

^{1}H NMR (400 MHz, $CDCl_3$) δ 8.04 (d, *J* = 6.8 Hz, 2H), 7.57 – 7.52 (m, 1H), 7.45 – 7.41 (m, 2H), 5.19 – 5.11 (m, 1H), 1.77 – 1.70 (m, 1H), 1.65 – 1.56 (m, 1H), 1.44 – 1.20 (m, 13H), 0.87 (t, *J* = 6.8 Hz , 3H).

^{13}C NMR (101 MHz, $CDCl_3$) δ 166.4, 132.8, 131.1, 129.6, 128.4, 71.9, 36.2, 31.9, 29.3, 25.6, 22.7, 20.2, 14.2.

HPLC (OJ-H, 0.46*25 cm, 5 μm, hexane/isopropanol = 99.5/0.5, flow rate = 1 mL/min, detection at 254 nm) retention time = 5.0 min (major) and 5.4 min (minor).

(*R*)-hexan-2-ylbenzoate (6i)

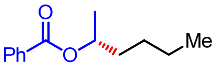

6i

Obtained as colorless oil (22.2 mg, 54% yield, 96% ee).

^{1}H NMR (400 MHz, $CDCl_3$) δ 8.05 (d, *J* = 6.8 Hz, 2H), 7.57 – 7.52 (m, 1H), 7.45 – 7.41 (m, 2H), 5.20 – 5.12 (m, 1H), 1.79 – 1.70 (m, 1H), 1.66 – 1.57 (m, 1H), 1.45 – 1.33 (m, 7H), 0.91 (t, *J* = 6.8 Hz, 3H).

^{13}C NMR (151 MHz, $CDCl_3$) δ 166.4, 132.8, 131.1, 129.6, 128.4, 71.9, 35.9, 27.7, 22.7, 20.2, 14.1.

HPLC (AD-H, 0.46*25 cm, 5 μm, hexane/isopropanol = 99.5/0.5, flow rate = 1 mL/min, detection at 210 nm) retention time = 7.3 min (major) and 8.3 min (minor).

(*R*)-1-(oxetan-3-yl)ethyl benzoate (6j)

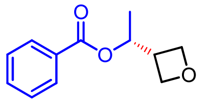

6j

Obtained as colorless oil (15.2 mg, 37% yield, 97% ee).

¹H NMR (400 MHz, $CDCl_3$) δ 8.06 (d, *J* = 7.2 Hz, 2H), 7.59 – 7.55 (m, 1H), 7.45 (t, *J* = 7.6 Hz, 2H), 5.45 – 5.38 (m, 1H), 4.79 (dt, *J* = 14.0, 6.0 Hz, 2H), 4.71 (t, *J* = 6.4 Hz, 1H), 4.53 (t, *J* = 6.4 Hz, 1H), 3.35 – 3.26 (m, 1H), 1.31 (d, *J* = 6.4 Hz, 3H).

¹³C NMR (101 MHz, $CDCl_3$) δ 166.3, 133.2, 130.4, 129.8, 128.6, 74.0, 73.3, 71.6, 40.0, 17.5.

HRMS (ESI-TOF) Calcd for $C_{12}H_{15}O_3$ $(M+H)^+$ 207.1016. Found 207.1015.

HPLC (AD-H, 0.46*25 cm, 5 μm, hexane/isopropanol = 98/2, flow rate = 1 mL/min, detection at 210 nm) retention time = 9.9 min (minor) and 13.1 min (major).

(*R*)-1-phenylnonan-4-yl benzoate (6k)

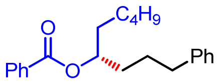

6k

Obtained as colorless oil (45.4 mg, 70% yield, 92% ee).(from (*Z*) enol ester).

¹H NMR (600 MHz, $CDCl_3$) δ 7.97 (d, *J* = 6.6 Hz, 2H), 7.49 – 7.46 (m, 1H), 7.37 – 7.35 (m, 2H), 7.20 – 7.17 (m, 2H), 7.11 – 7.08 (m, 2H), 5.12 – 5.08 (m, 1H), 2.61 – 2.52 (m, 2H), 1.68 – 1.52 (m, 6H), 1.35 – 1.12 (m, 6H), 0.80 – 0.78 (m, 3H).

¹³C NMR (151 MHz, $CDCl_3$) δ 166.5, 142.3, 132.9, 130.9, 129.7, 128.4, 125.9, 74.9, 35.9, 34.3, 33.9, 31.9, 27.2, 25.1, 22.7.

HPLC (OD-H, 0.46*25 cm, 5 μm, hexane/isopropanol = 99.5/0.5, flow rate = 1 mL/min, detection at 210 nm) retention time = 24.5 min (major) and 26.7 min (minor).

(*R*)-1-(9*H*-carbazol-9-yl)nonan-4-yl 4-methoxybenzoate (6l)

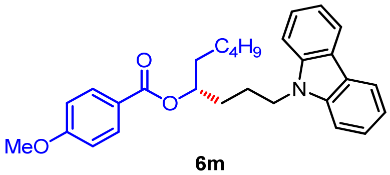

6m

Obtained as colorless oil (46.9 mg, 66% yield, 91% ee). (from (*Z*) enol ester).

¹H NMR (600 MHz, $CDCl_3$) δ 8.10 (d, *J* = 7.8 Hz, 1H), 7.98 (d, *J* = 8.9 Hz, 2H), 7.47

– 7.38 (m, 4H), 7.23 (t, J = 7.6 Hz, 2H), 6.92 (d, J = 8.8 Hz, 2H),5.21 – 5.17 (m, 1H), 4..38 – 4.31 (m, 2H), 3.86 (s, 3H), 2.02 – 1.92 (m, 2H), 1.82 – 1.70 (m, 2H), 1.68 – 1.60 (m, 1H), 1.56 – 1.51 (m, 1H), 1.36 – 1.18 (m, 6H), 0.84 (t, J = 6.8 Hz, 3H).

^{13}C NMR (151 MHz, $CDCl_3$) δ 166.3, 163.5, 140.5, 131.7, 125.8, 123.1, 123.0, 120.5, 118.9, 113.8, 108.8, 73.9, 55.6, 42.9, 34.4, 31.9, 31.8, 25.1, 24.8, 22.6, 14.1.

HRMS (ESI-TOF) Calcd for $C_{29}H_{33}NNaO_3$ $(M+Na)^+$ 466.2353. Found 466.2354.

HPLC (AS-H, 0.46*25 cm, 5 µm, hexane/isopropanol = 98/2, flow rate = 1 mL/min, detection at 254 nm) retention time = 5.9 min (major) and 6.7 min (minor).

(*R*)-1-(9*H*-carbazol-9-yl)undecan-4-yl benzoate (6m)

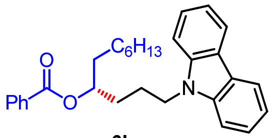

6l

Obtained as colorless oil (51.2 mg, 58% yield, 94% ee). (from (*Z*) enol ester).

^{1}H NMR (600 MHz, $CDCl_3$) δ 8.09 (d, J = 7.7 Hz, 2H), 8.02 (d, J = 7.2 Hz, 2H), 7.56 (t, J = 7.3 Hz, 1H), 7.46 – 7.36 (m, 6H), 7.22 (t, J = 7.4 Hz, 2H), 5.23 – 5.19 (m, 1H), 4.38 – 4.31 (m, 2H), 2.07 – 1.91 (m, 2H), 1.81 – 1.61 (m, 3H), 1.31 – 1.15 (m, 11H), 0.85 (t, J = 6.9 Hz, 3H).

^{13}C NMR (151 MHz, $CDCl_3$) δ 166.6, 140.5, 133.0, 130.6, 129.7, 128.5, 125.8, 123.0, 120.5, 119.0, 108.8, 74.3, 42.8, 34.4, 31.9, 31.9, 29.5, 29.24, 25.4, 24.8, 22.7, 14.2.

HRMS (ESI-TOF) Calcd for $C_{30}H_{35}NNaO_2$ $(M+Na)^+$ 464.2560. Found 464.2553.

HPLC (OD-H, 0.46*25 cm, 5 µm, hexane/isopropanol = 90/10, flow rate = 1 mL/min, detection at 210 nm) retention time = 6.9 min (minor) and 9.4 min (major).

(*R*)-1-(9*H*-carbazol-9-yl)-9-chlorononan-4-yl benzoate (6n)

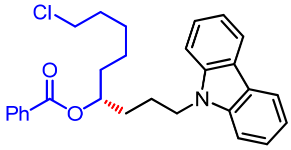

6n

Obtained as colorless oil (60.0 mg, 68% yield, 94% ee). (from (*Z*) enol ester).

^{1}H NMR (600 MHz, $CDCl_3$) δ 8.11 (d, J = 7.5 Hz, 2H), 8.03 (d, J = 7.7 Hz, 2H), 7.57

(t, J = 7.4 Hz, 1H), 7.48 – 7.38 (m, 6H), 7.24 (t, J = 7.6 Hz, 2H), 5.24 – 5.20 (m, 1H), 4.36 (t, J = 7.1 Hz, 2H), 3.47 (t, J = 6.7 Hz, 2H), 2.06 – 1.95 (m, 2H), 1.81 – 1.77 (m, 1H), 1.74 – 1.64 (m, 4H), 1.60 – 1.52 (m, 1H), 1.45 – 1.25 (m, 4H).

^{13}C NMR (151 MHz, $CDCl_3$) δ 166.5, 140.5, 133.1, 130.5, 129.7, 128.5, 125.8, 123.0, 120.5, 119.0, 108.8, 74.0, 45.0, 42.8, 34.3, 32.5, 31.8, 26.79, 24.8.

HRMS (ESI-TOF) Calcd for $C_{28}H_{31}ClNO_2$ $(M+H)^+$ 448.2038. Found 448.2040.

HPLC (AS-H, 0.46*25 cm, 5 μm, hexane/isopropanol = 98/2, flow rate = 1 mL/min, detection at 254 nm) retention time = 7.3 min (major) and 8.8 min (minor).

Mechanistic Studies

Experimental procedure:

In a nitrogen-filled glovebox, $Ni(COD)_2$ (5.5 mg, 0.02 mmol, 10 mol%) and **L41** (8.4 mg, 0.024 mmol, 12 mol%) were dissolved in solvent (2 mL, Et_2O: DMF = 3:1) in Schlenk tube with screw-cap equipped with a magnetic stirrer. The mixture was stirred at room temperature for 10 min, then (3-iodopropyl)benzene (98.4 mg, 0.4 mmol, 2.0 equiv), acyl enamide (29.4 mg, 0.2 mmol, 1.0 equiv), and K_3PO_4•H_2O (138.0 mg, 0.6 mmol, 3.0 equiv) were added sequentially. The mixture was stirred at room temperature for another 5 minutes before Ph_2SiD_2 (111.6 mg, 0.6 mmol, 3.0 equiv) was added dropwise. The resulting mixture was stirred at room temperature for 12 h. The mixture was filtered through a pad of silica gel and washed with ethyl acetate (3 × 15 mL), then washed with water (15 mL). The organic phase was dried over Na_2SO_4, filtered, concentrated under reduced pressure, purified by flash chromatography with silica gel to give the pure product.

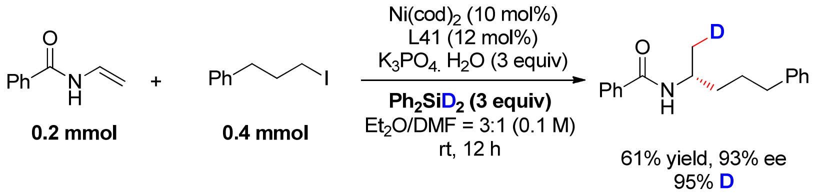

(*R*)-N-(5-phenylpentan-2-yl-1-*d*)benzamide (7)

Obtained as white solid (32.6 mg, 61% yield, 92% ee).

^{1}H NMR (400 MHz, $CDCl_3$) δ 7.70 (d, J = 6.8 Hz, 2H), 7.48 – 7.42 (m, 1H), 7.39 –

7.36 (m, 2H), 7.26 – 7.22 (m, 2H), 7.16 – 7.13 (m, 3H), 5.83 (d, J = 8.4 Hz, 1H), 4.25 – 4.16 (m, 1H), 2.67 – 2.56 (m, 2H), 1.73 – 1.65 (m, 2H), 1.57 – 1.52 (m, 2H), 1.20 – 1.69 (m, 2H).

^{13}C NMR (101 MHz, $CDCl_3$) δ 167.0, 142.2, 135.0, 131.4, 128.6, 128.5, 128.4, 126.9, 125.9, 45.7, 36.6, 35.8, 28.0, 20.9 (t, J = 19.6 Hz).

HRMS (ESI-TOF) Calcd for $C_{18}H_{20}DNO$ $(M+H)^+$ 269.1759. Found 269.1755.

HPLC (OJ-H, 0.46*25 cm, 5 μm, hexane/isopropanol = 90/10, flow rate = 1 mL/min, detection at 210 nm) retention time = 15.6 min (major) and 18.7 min (minor).

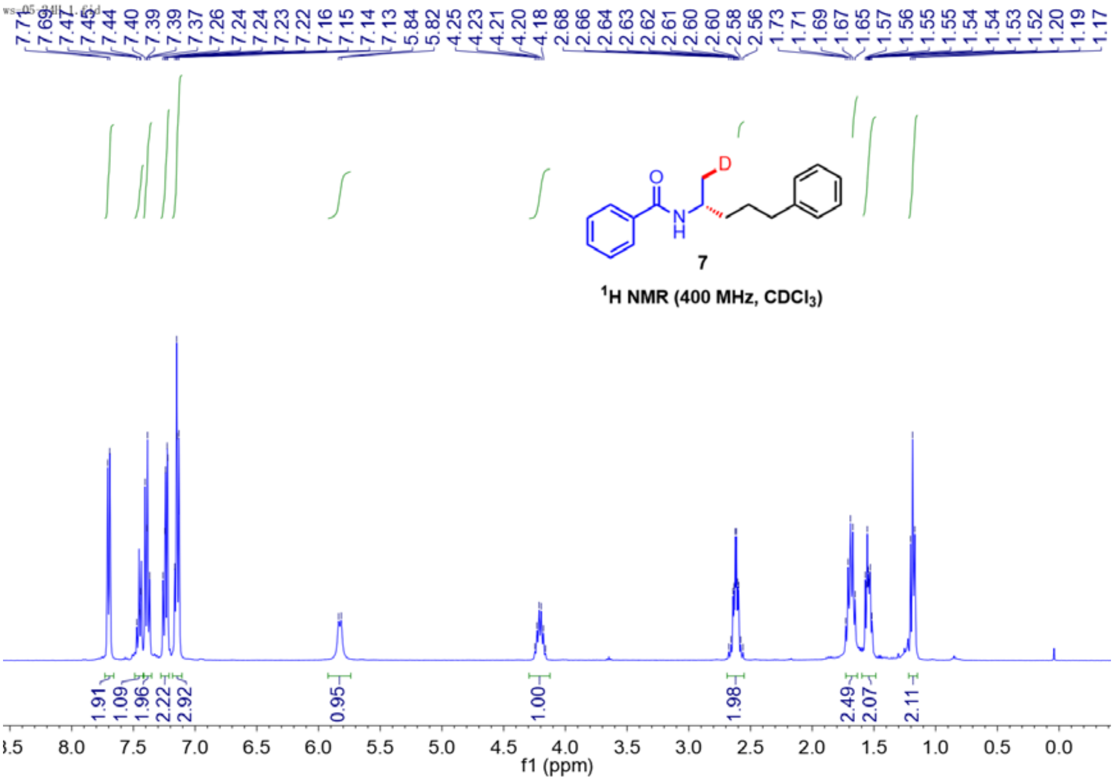

Supplementary Figure 3 ^{1}H NMR (400 MHz, $CDCl_3$) of **7**

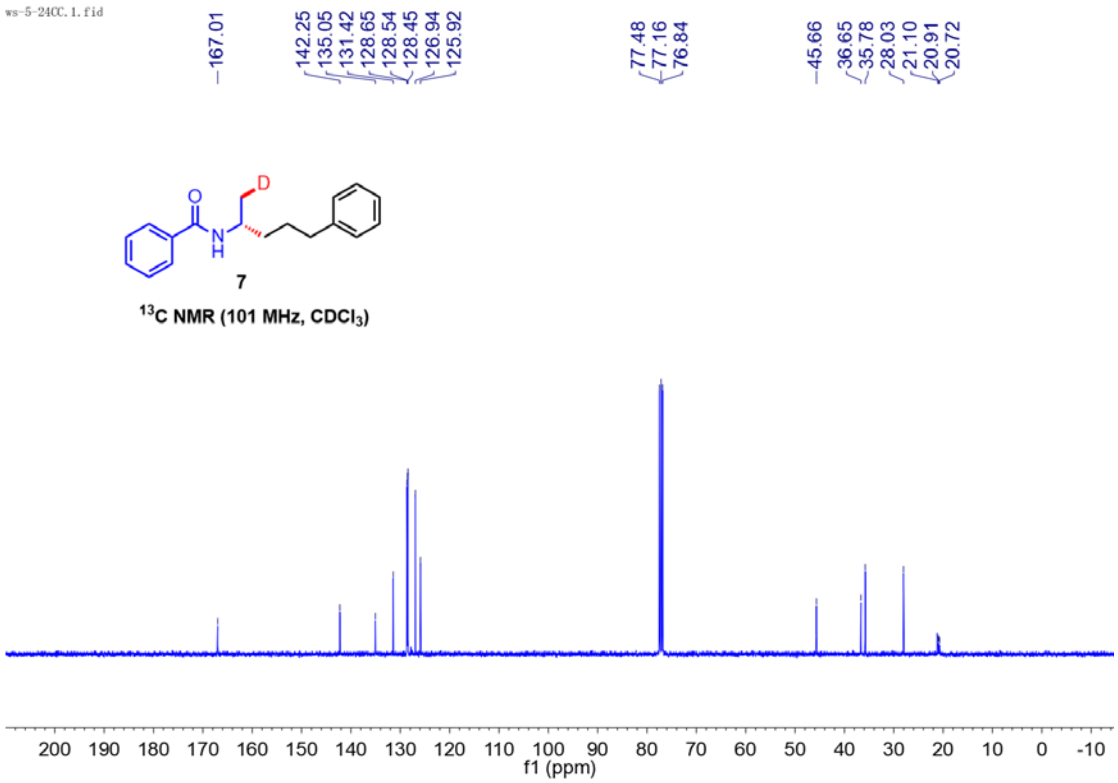

Supplementary Figure 4 ^{13}C NMR (101 MHz, $CDCl_3$) of **7**

Experimental procedure:

In a nitrogen-filled glovebox, Ni(COD)$_2$ (5.5 mg, 0.01 mmol, 10 mol%) and (1*S*, 2*R*)-**L9** (21.8 mg, 0.024 mmol, 12 mol%) were dissolved in solvent (2 mL, Et_2O: DMF = 3:1) in Schlenk tube with screw-cap equipped with a magnetic stirrer. The mixture was stirred at room temperature for 10 min, then 9-(3-iodopropyl)-9*H*-carbazole (200.0 mg, 0.6 mmol, 3.0 equiv), acyl enamine (25.2 mg, 0.2 mmol, 1.0 equiv), K_3PO_4•H_2O (138.0 mg, 0.6 mmol, 3.0 equiv), Ph_2SiD_2 (111.6 mg, 0.6 mmol, 3.0 equiv) were sequentially added. The resulting mixture was stirred at 45℃ for 16 h. The mixture was filtered through a pad of silica gel and washed with ethyl acetate (3 × 15 mL), then washed with water (15 mL). The organic phase was dried over Na_2SO_4, filtered, concentrated under reduced pressure, purified by flash chromatography with silica gel to give the pure product.

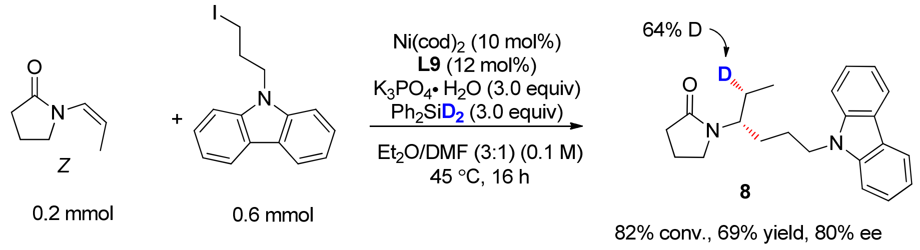

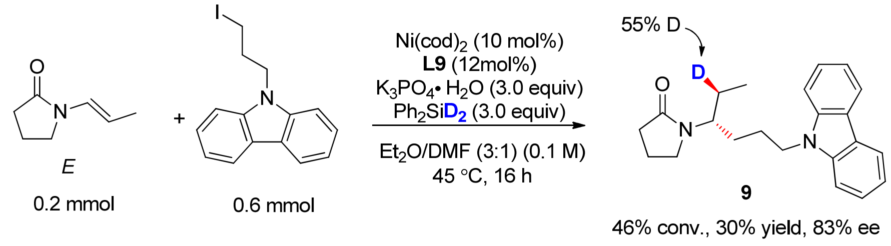

1-((2*R*,3*S*)-6-(9*H*-carbazol-9-yl)-2-*d*-hexan-3-yl)pyrrolidin-2-one (8)

Obtained as white solid (46.2 mg, 69% yield, 80% ee).

^{1}H NMR (600 MHz, $CDCl_3$) δ 8.10 (d, J = 7.7 Hz, 2H), 7.46 (t, J = 7.3 Hz, 2H), 7.42 (d, J = 8.1 Hz, 2H), 7.3 (t, J = 7.9 Hz, 2H), 4.41– 4.36 (m, 1H), 4.32 – 4.27 (m, 1H), 4.09 – 4.04 (m, 1H), 3.05 – 3.01 (m, 1H), 2.82 – 2.78 (m, 1H), 2.42 – 2.31 (m, 2H), 1.91 – 1.70 (m, 4H), 1.52 – 1.46 (m, 1H), 1.42 – 1.35 (m, 2H), 1.34 – 1.31(m, 0.37H), 0.78 (t, J = 8.0 Hz, 3H).

^{13}C NMR (151 MHz, $CDCl_3$) δ 175.7, 140.5, 125.8, 122.9, 120.4, 118.9, 108.9, 51.9, 51.8, 42.5, 41.4, 31.6, 29.3, 25.7, 25.3 (t, J = 19.6 Hz), 18.2, 10.8.

HRMS (ESI-TOF) Calcd for $C_{22}H_{26}DN_2O$ $(M+H)^+$ 336.2181. Found 336.2177.

HPLC (AD-H, 0.46*25 cm, 5 μm, hexane/isopropanol = 95/5, flow rate = 1 mL/min, detection at 210 nm) retention time = 18.1 min (major) and 28.8 min (minor).

1-((2*S*,3*S*)-6-(9*H*-carbazol-9-yl)-2-*d*-hexan-3-yl)pyrrolidin-2-one (9)

Obtained as white solid (20.1 mg, 30% yield, 83% ee).

^{1}H NMR (600 MHz, $CDCl_3$) δ 8.10 (d, J = 7.7 Hz, 2H), 7.46 (t, J = 7.3 Hz, 2H), 7.42 (d, J = 8.1 Hz, 2H), 7.3 (t, J = 7.9 Hz, 2H), 4.41– 4.36 (m, 1H), 4.32 – 4.27 (m, 1H), 4.09 – 4.04 (m, 1H), 3.05 – 3.01 (m, 1H), 2.82 – 2.78 (m, 1H), 2.42 – 2.31 (m, 2H), 1.91 – 1.70 (m, 4H), 1.52 – 1.46 (m, 1H), 1.42 – 1.35 (m, 1.46H), 1.34 – 1.31(m, 1H), 0.78 (t, J = 8.0 Hz, 3H).

^{13}C NMR (151 MHz, $CDCl_3$) δ 175.8, 140.5, 125.8, 123.0, 120.4, 118.9, 108.9, 52.0, 51.9, 42.3, 41.5, 31.6, 29.4, 25.7 25.3 (t, J = 19.6 Hz), 18.2, 10.8.

HRMS (ESI-TOF) Calcd for $C_{22}H_{26}DN_2O$ $(M+H)^+$ 336.2181. Found 336.2176

HPLC (AD-H, 0.46*25 cm, 5 μm, hexane/isopropanol = 95/5, flow rate = 1 mL/min, detection at 210 nm) retention time = 18.1 min (major) and 28.8 min (minor).

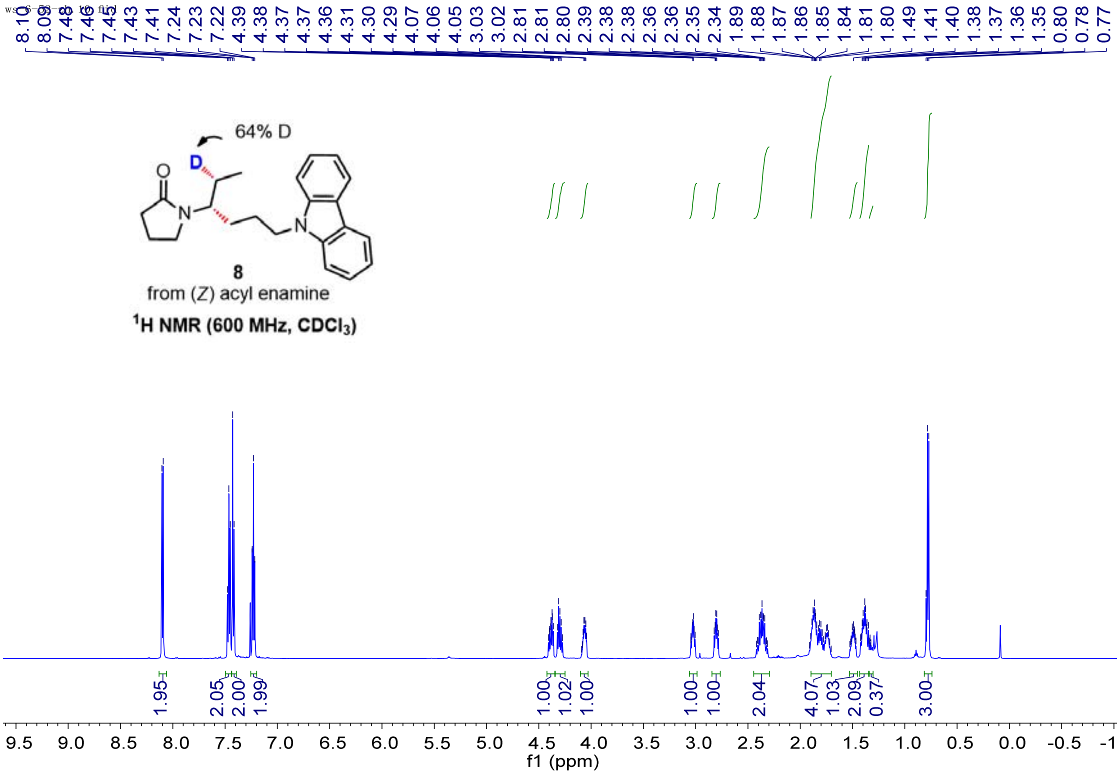

Supplementary Figure 5 ^{1}H NMR (400 MHz, $CDCl_3$) of **8**

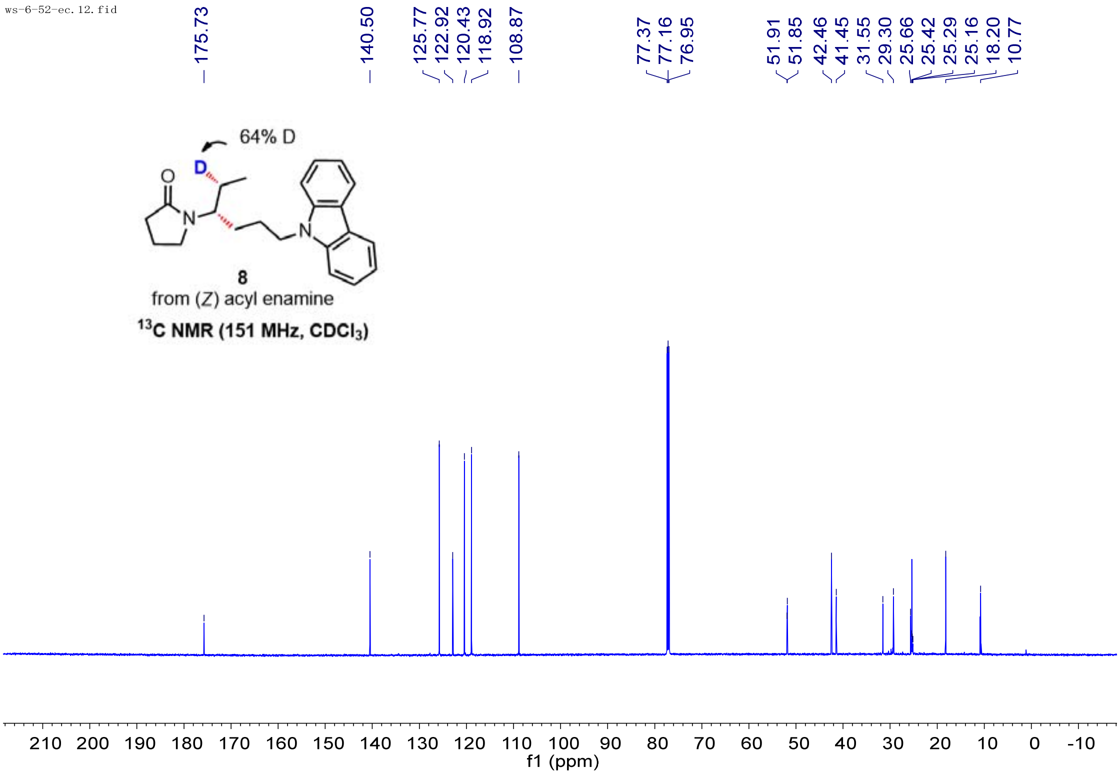

Supplementary Figure 6 ^{13}C NMR (151 MHz, $CDCl_3$) of **8**

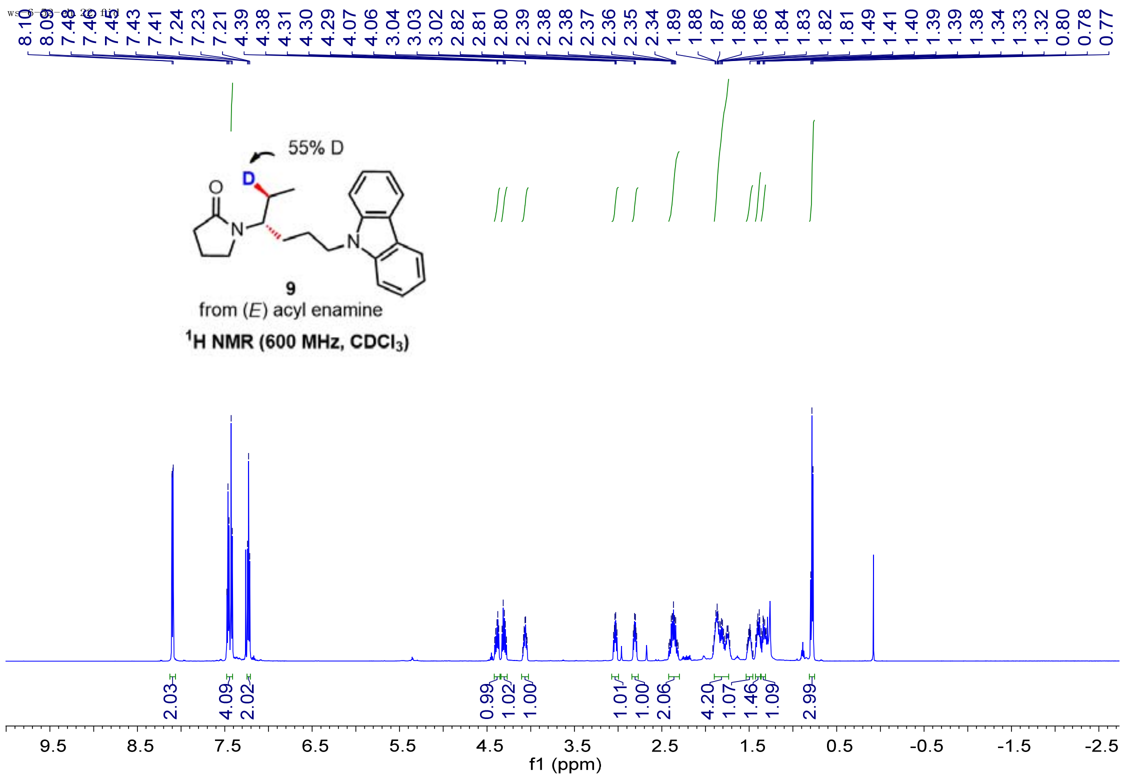

Supplementary Figure 7 ^{1}H NMR (400 MHz, $CDCl_3$) of **9**

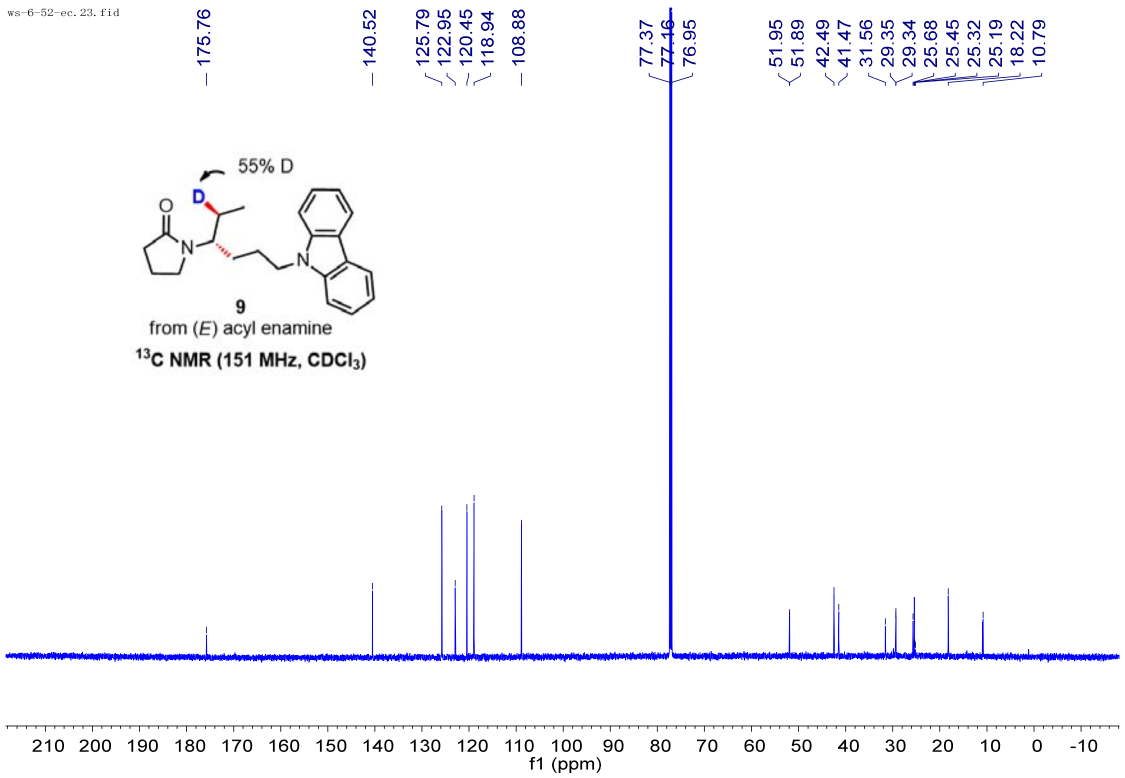

Supplementary Figure 8 ^{13}C NMR (151 MHz, $CDCl_3$) of **9**

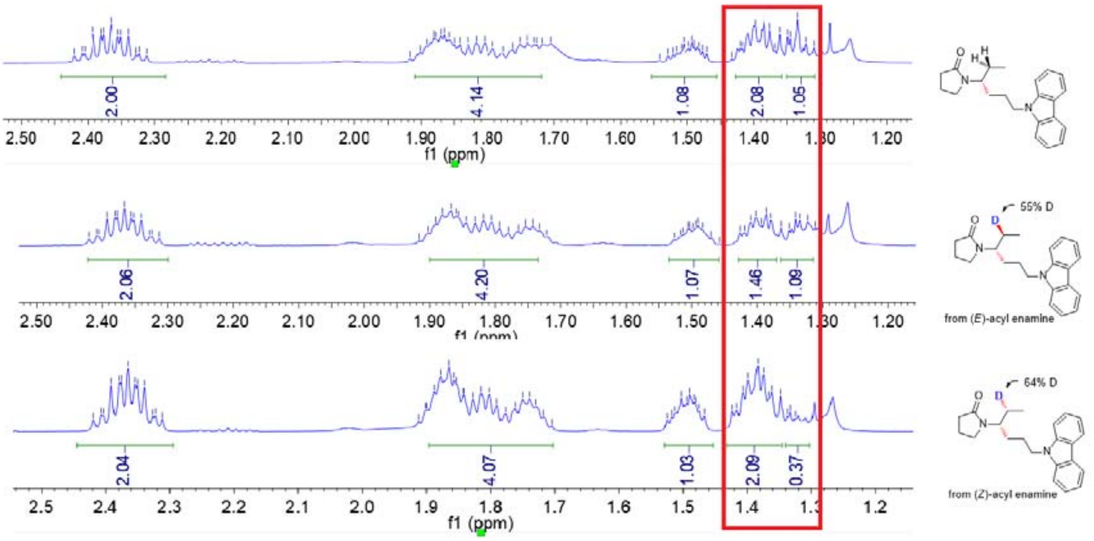

Supplementary Figure 9 Local Comparison of ^{1}H NMR Spectra Between **3o, 8** & **9**

X-Ray Diffraction Data of 4l and 5a

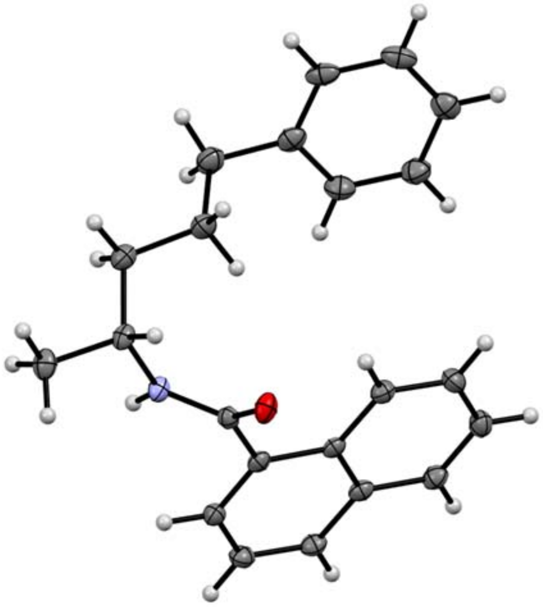

Supplementary Figure 10 X-ray structure of compound **4l** (CCDC 2042844)

Supplementary Table 15 Crystal data and structure refinement for cxy3098_0m (4l)

Identification code	cxy3098_0m
Empirical formula	$C_{22}H_{23}NO$
Formula weight	317.41
Temperature/K	100.0
Crystal system	orthorhombic
Space group	$P2_12_12_1$
a/Å	5.0313(3)
b/Å	11.7146(8)
c/Å	29.492(2)
α/°	90
β/°	90
γ/°	90

Volume/Å^3	1738.2(2)
Z	4
ρ_{calc}g/cm^3	1.213
μ/mm^{-1}	0.568
F(000)	680.0
Crystal size/mm^3	0.28 × 0.14 × 0.12
Radiation	CuKα (λ = 1.54178)
2Θ range for data collection/°	5.994 to 136.612
Index ranges	-6 ≤ h ≤ 6, -14 ≤ k ≤ 11, -35 ≤ l ≤ 35
Reflections collected	34281
Independent reflections	3163 [R_{int} = 0.0409, R_{sigma} = 0.0188]
Data/restraints/parameters	3163/0/219
Goodness-of-fit on F^2	1.052
Final R indexes [I>=2σ (I)]	R_1 = 0.0245, wR_2 = 0.0620
Final R indexes [all data]	R_1 = 0.0248, wR_2 = 0.0622
Largest diff. peak/hole / e Å^{-3}	0.15/-0.13
Flack parameter	0.03(6)

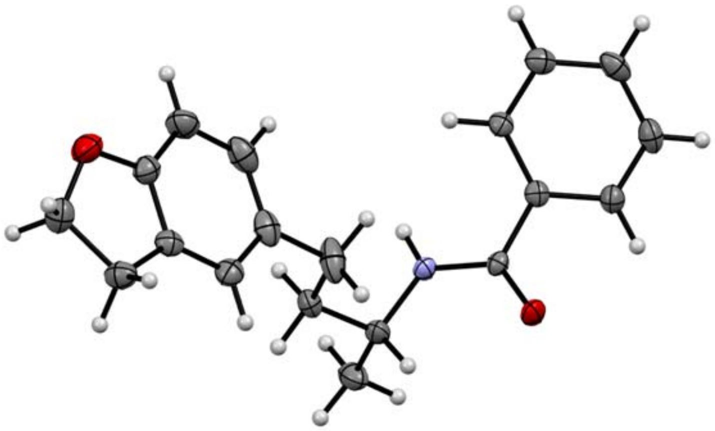

Supplementary Figure 11 X-ray structure of compound **5a** (CCDC 2042842)

Supplementary Table 16 Crystal data and structure refinement for cxy3096_0m (5a)

Identification code	cxy3096_0m
Empirical formula	$C_{19}H_{21}NO_2$
Formula weight	295.37
Temperature/K	100.0
Crystal system	monoclinic
Space group	$P2_1$
a/Å	5.0731(2)
b/Å	9.5645(3)
c/Å	16.3910(5)
α/°	90
β/°	91.7680(10)
γ/°	90
Volume/Å^3	794.94(5)
Z	2
ρ_{calc}g/cm^3	1.234
μ/mm^{-1}	0.630
F(000)	316.0
Crystal size/mm^3	0.38 × 0.38 × 0.12
Radiation	CuKα (λ = 1.54178)
2Θ range for data collection/°	5.394 to 136.616
Index ranges	$-6 \le h \le 6, -11 \le k \le 11, -19 \le l \le 19$
Reflections collected	13404
Independent reflections	2878 [R_{int} = 0.0263, R_{sigma} = 0.0238]
Data/restraints/parameters	2878/1/201

Goodness-of-fit on F^2	1.070
Final R indexes [I>=2σ (I)]	R_1 = 0.0249, wR_2 = 0.0612
Final R indexes [all data]	R_1 = 0.0253, wR_2 = 0.0614
Largest diff. peak/hole / e $Å^{-3}$	0.16/-0.12
Flack parameter	0.00(5)

Supplementary Figures

^{1}H NMR, ^{13}C NMR and ^{19}F NMR Spectra of Products

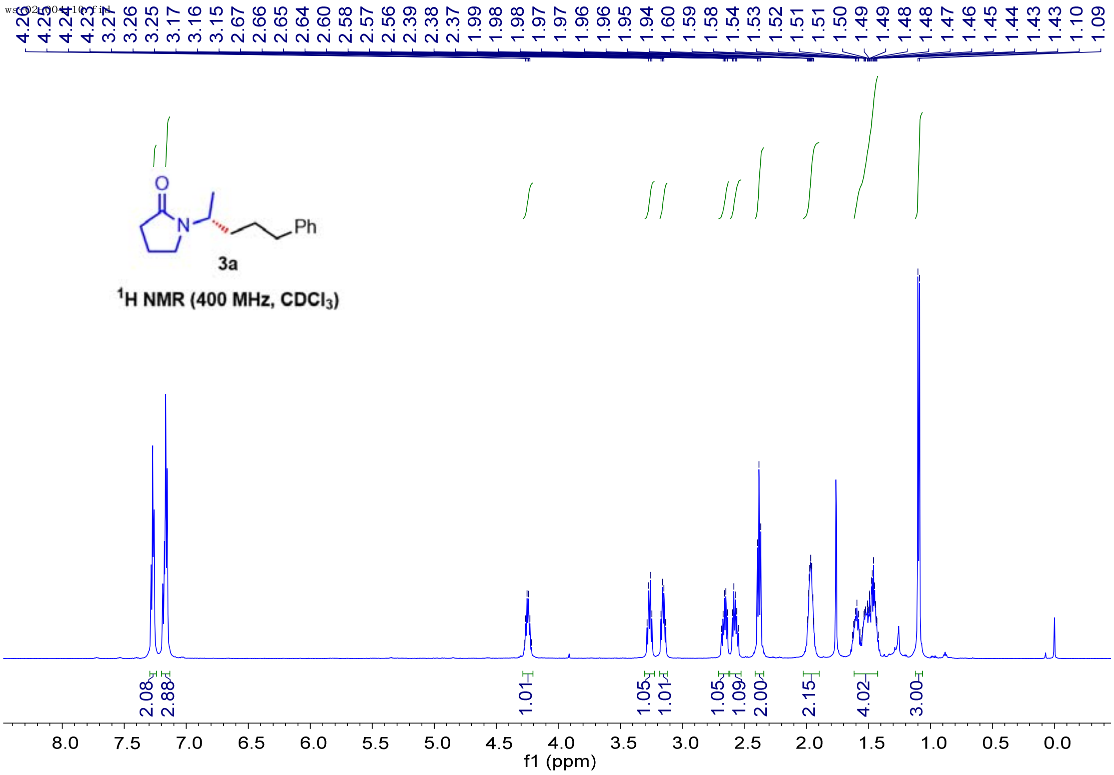

Supplementary Figure 12 ^{1}H NMR (400 MHz, $CDCl_3$) of **3a**

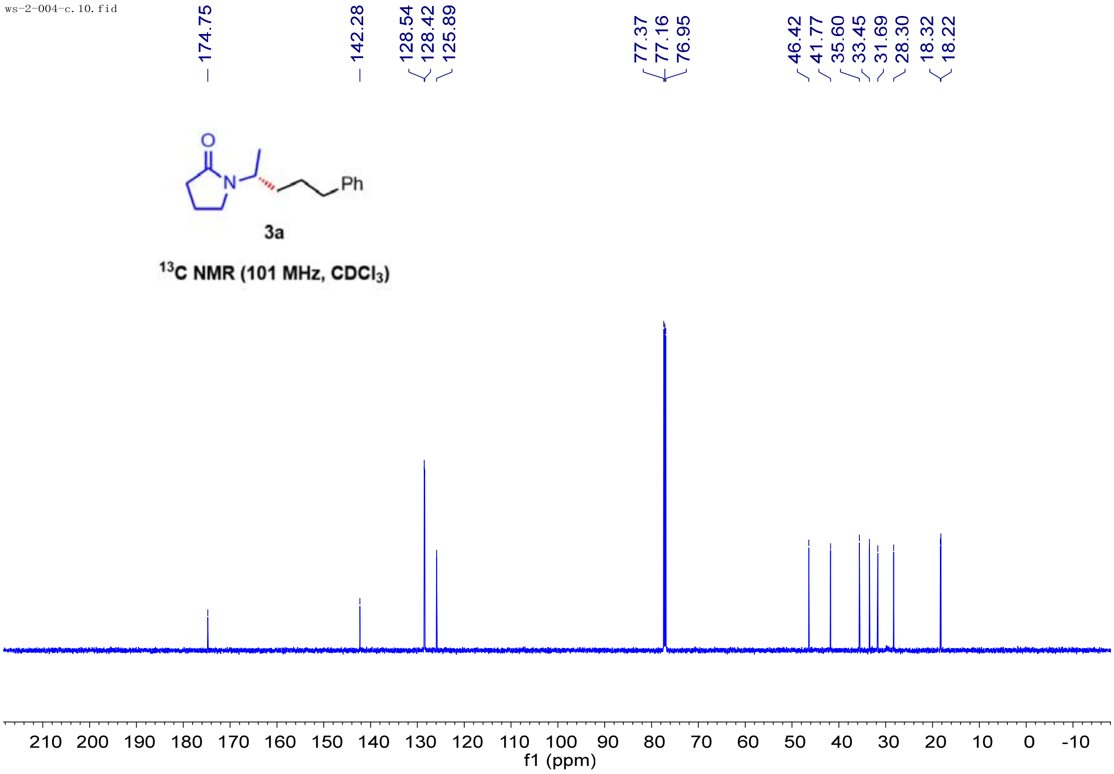

Supplementary Figure 13 ^{13}C NMR (101 MHz, $CDCl_3$) of **3a**

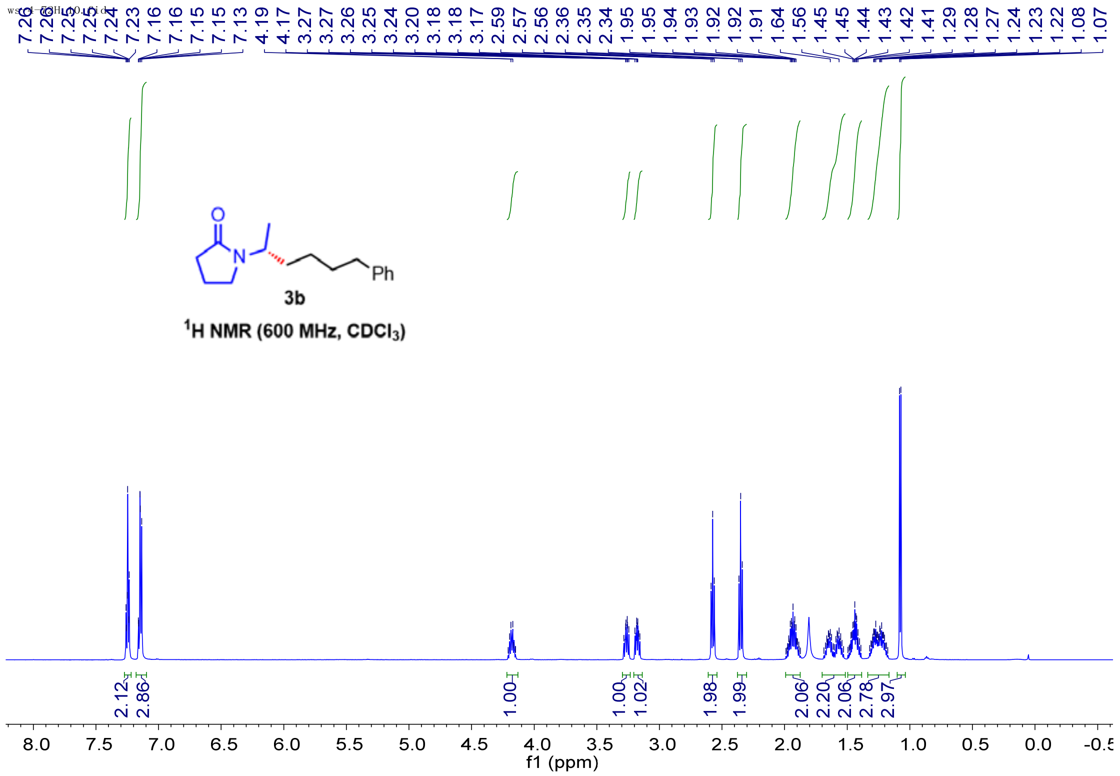

Supplementary Figure 14 ^{1}H NMR (600 MHz, $CDCl_3$) of **3b**

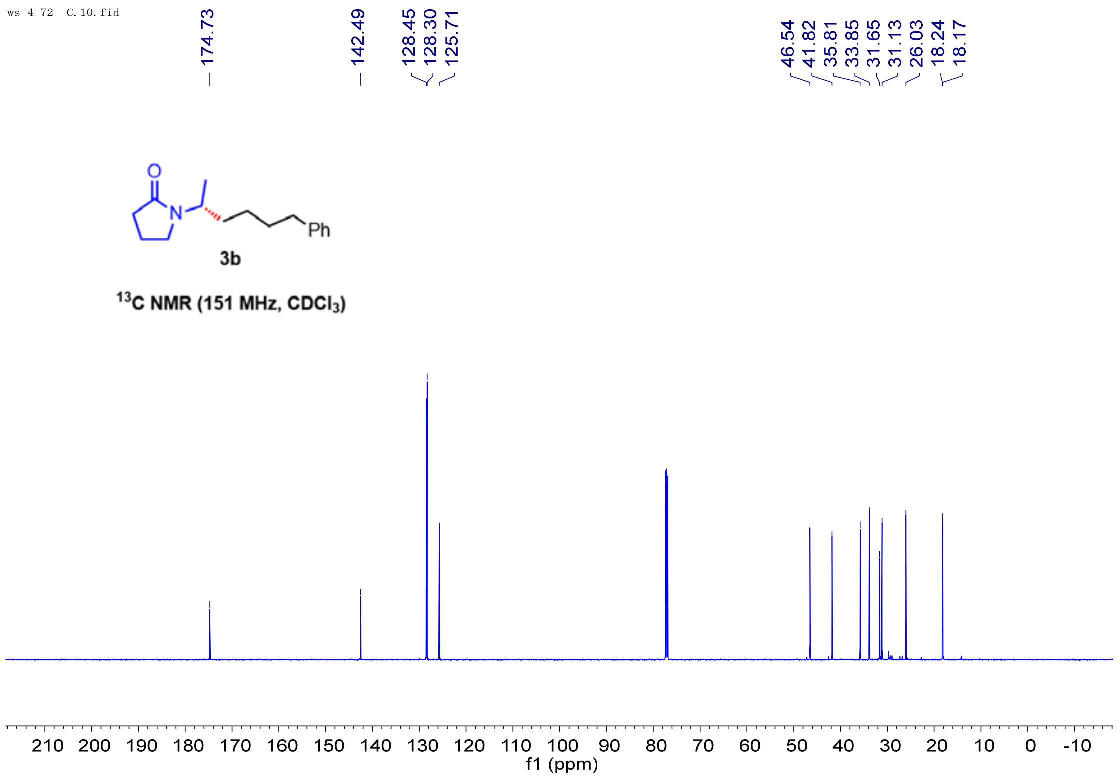

Supplementary Figure 15 ^{13}C NMR (151 MHz, $CDCl_3$) of **3b**

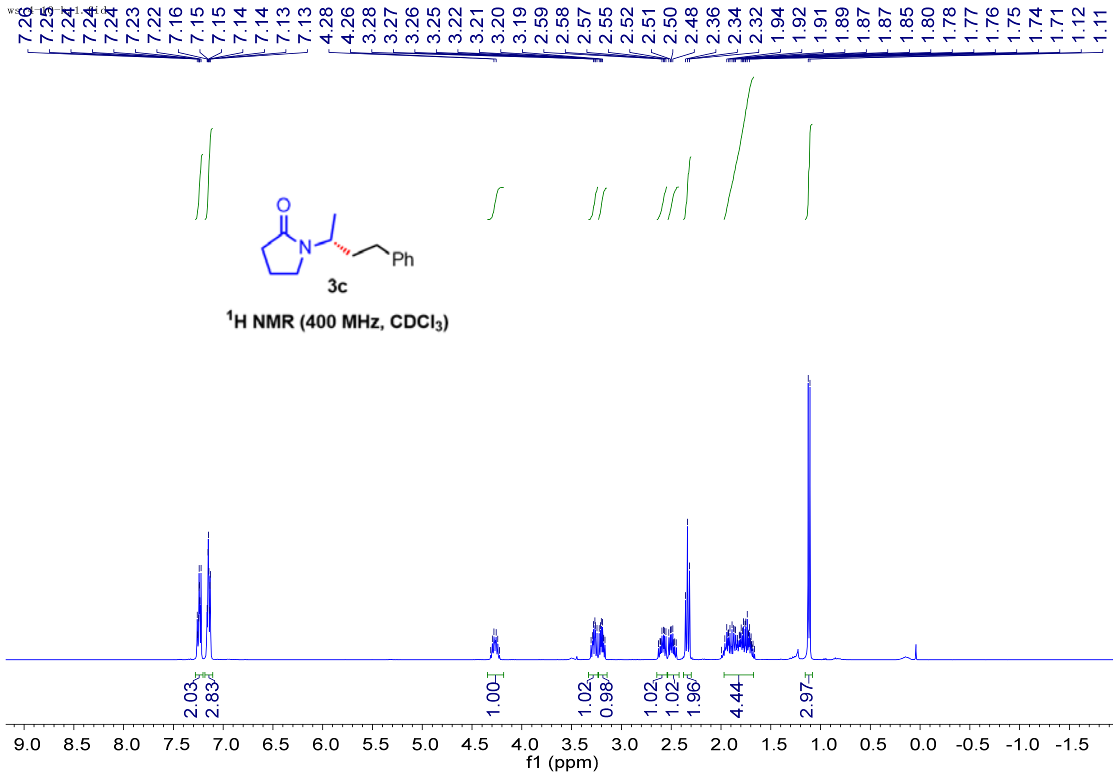

Supplementary Figure 16 ^{1}H NMR (400 MHz, $CDCl_3$) of **3c**

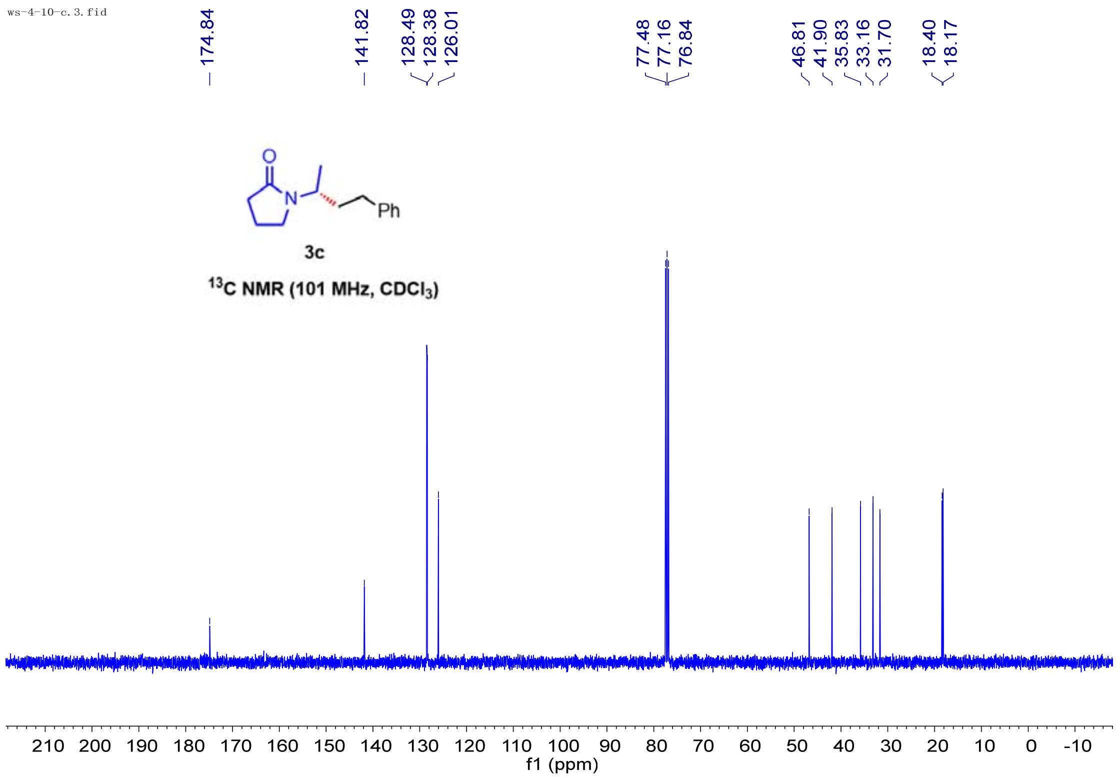

Supplementary Figure 17 ^{13}C NMR (101 MHz, $CDCl_3$) of **3c**

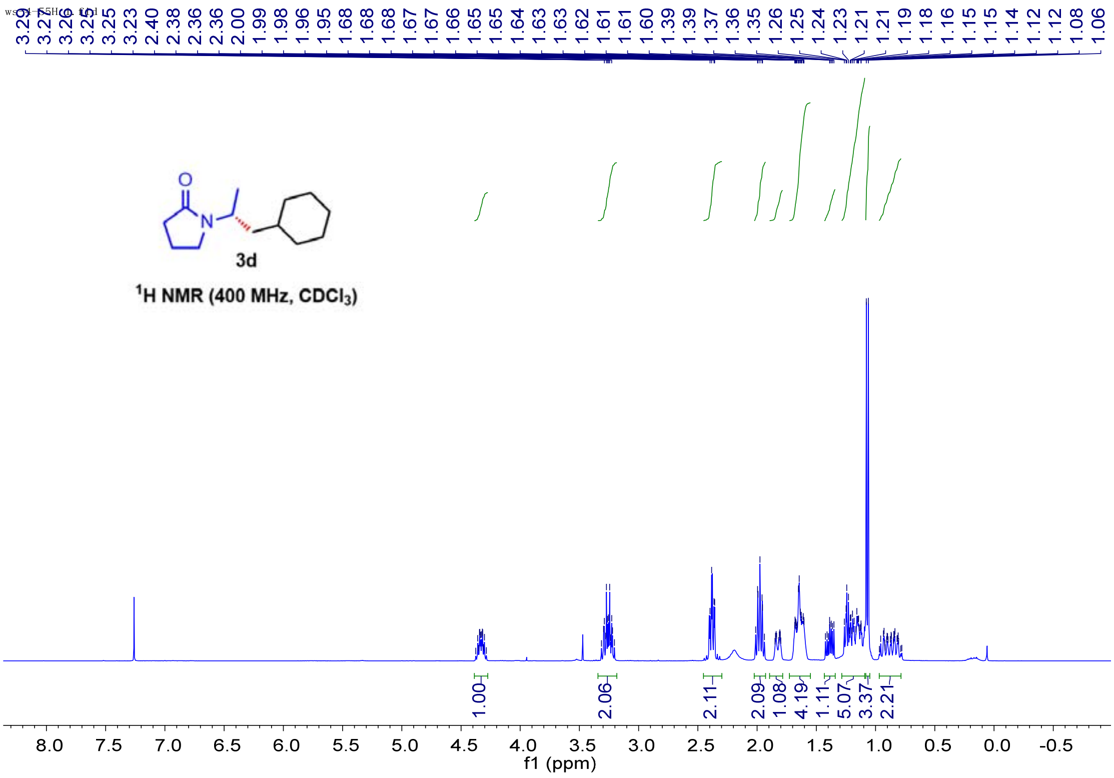

Supplementary Figure 18 ^{1}H NMR (400 MHz, $CDCl_3$) of **3d**

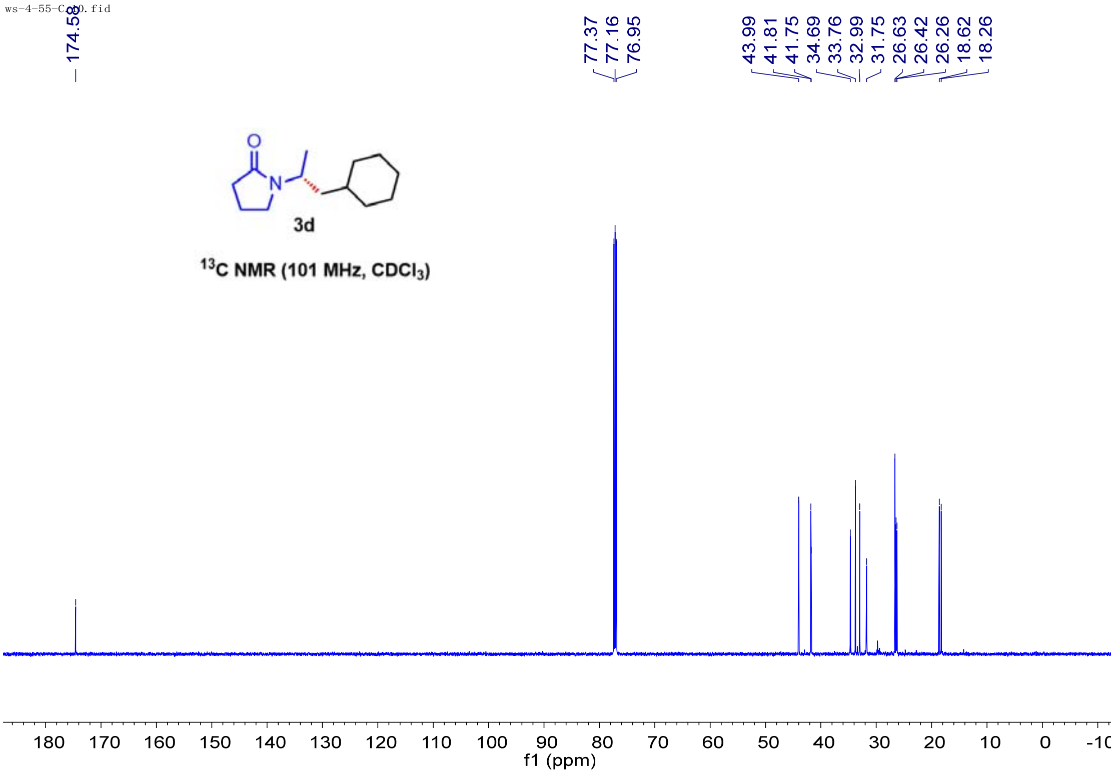

Supplementary Figure 19 ^{13}C NMR (101 MHz, $CDCl_3$) of **3d**

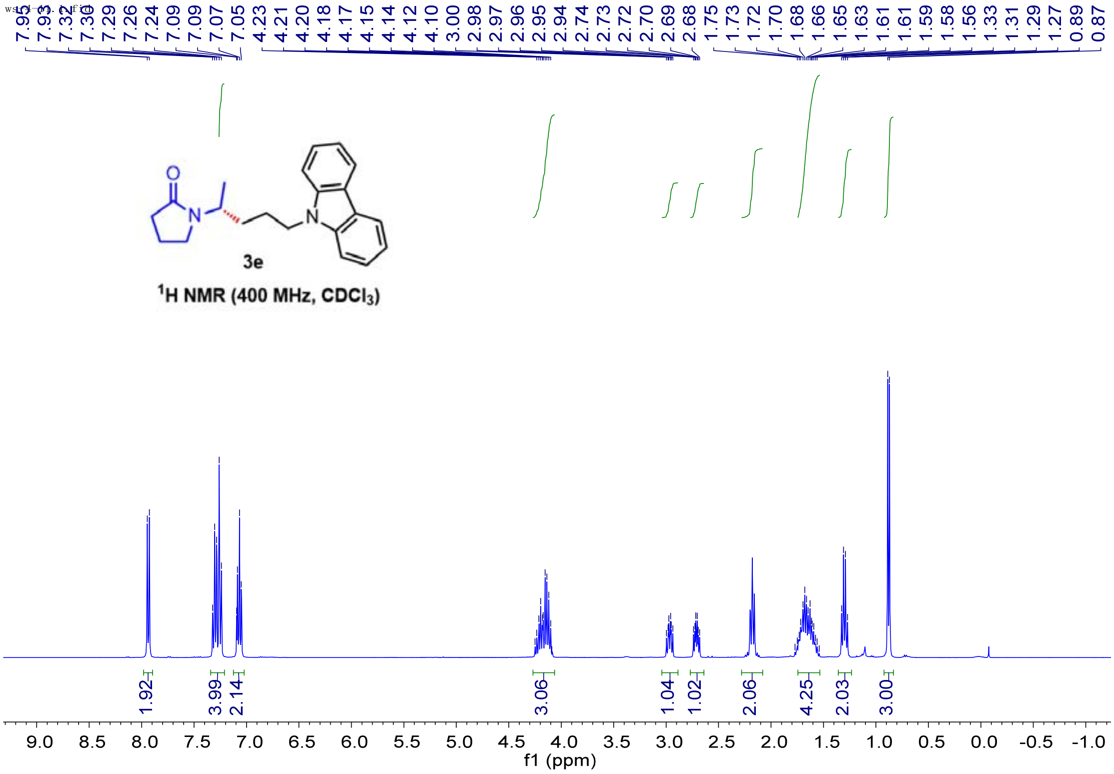

Supplementary Figure 20 ^{1}H NMR (400 MHz, $CDCl_3$) of **3e**

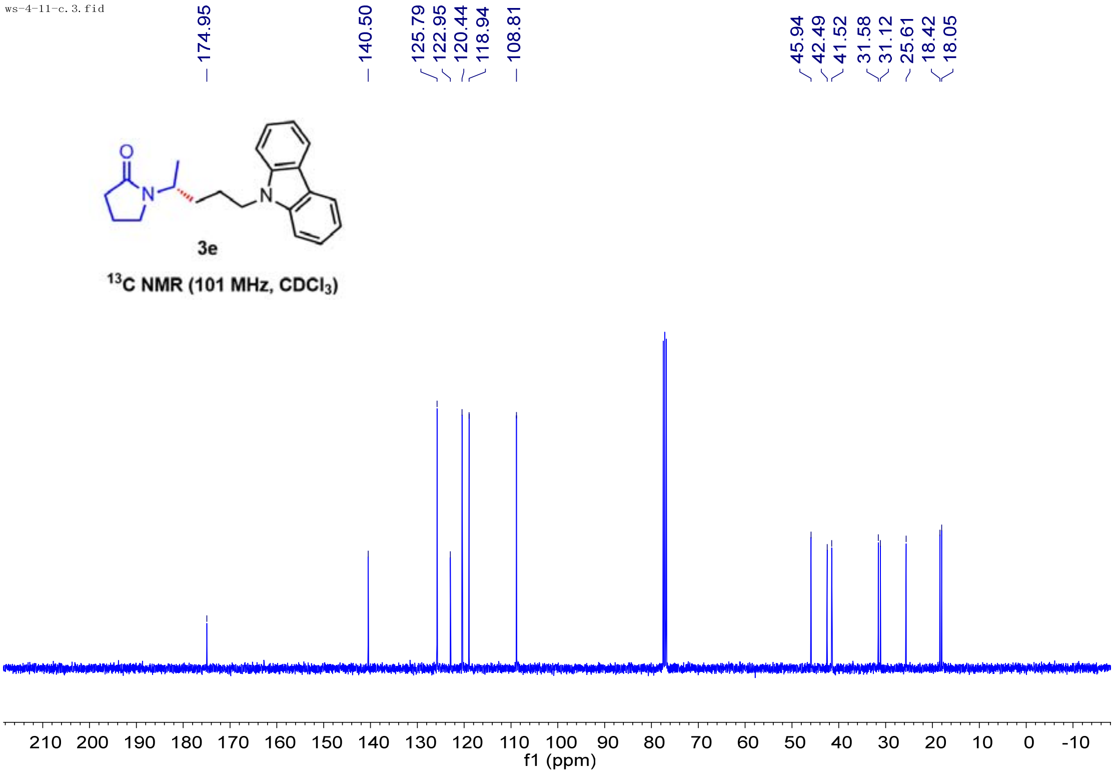

Supplementary Figure 21 ^{13}C NMR (101 MHz, $CDCl_3$) of **3e**

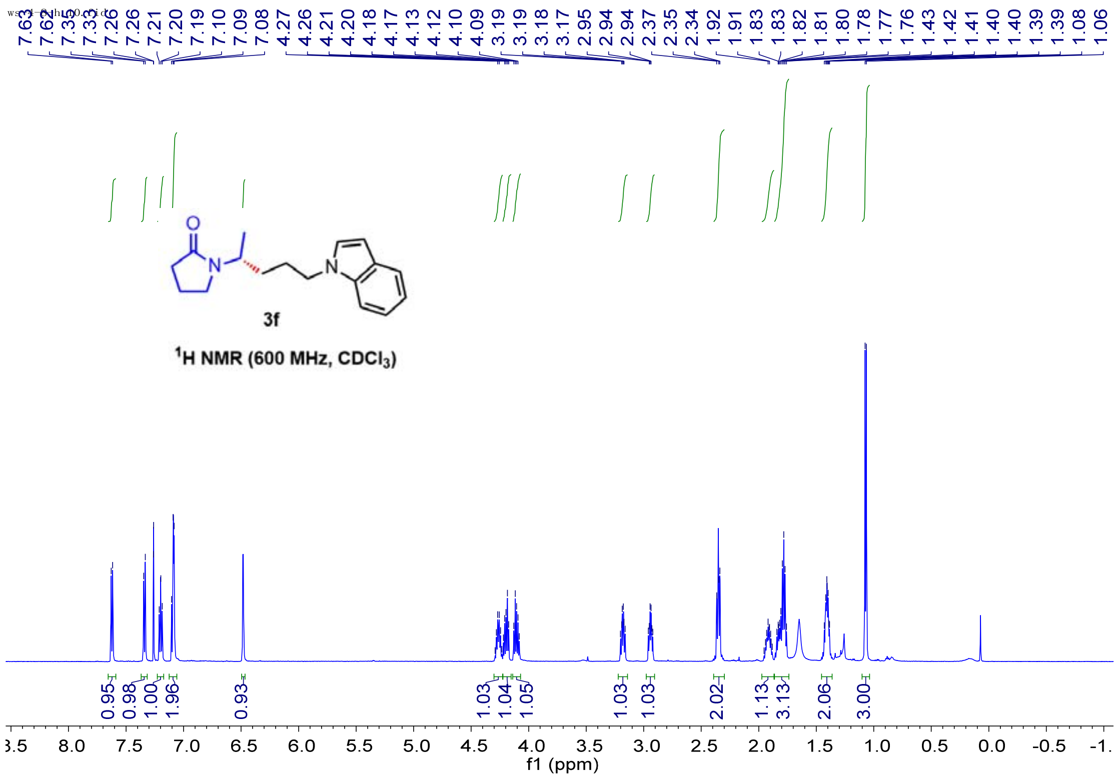

Supplementary Figure 22 ^{1}H NMR (600 MHz, CDCl$_3$) of **3f**

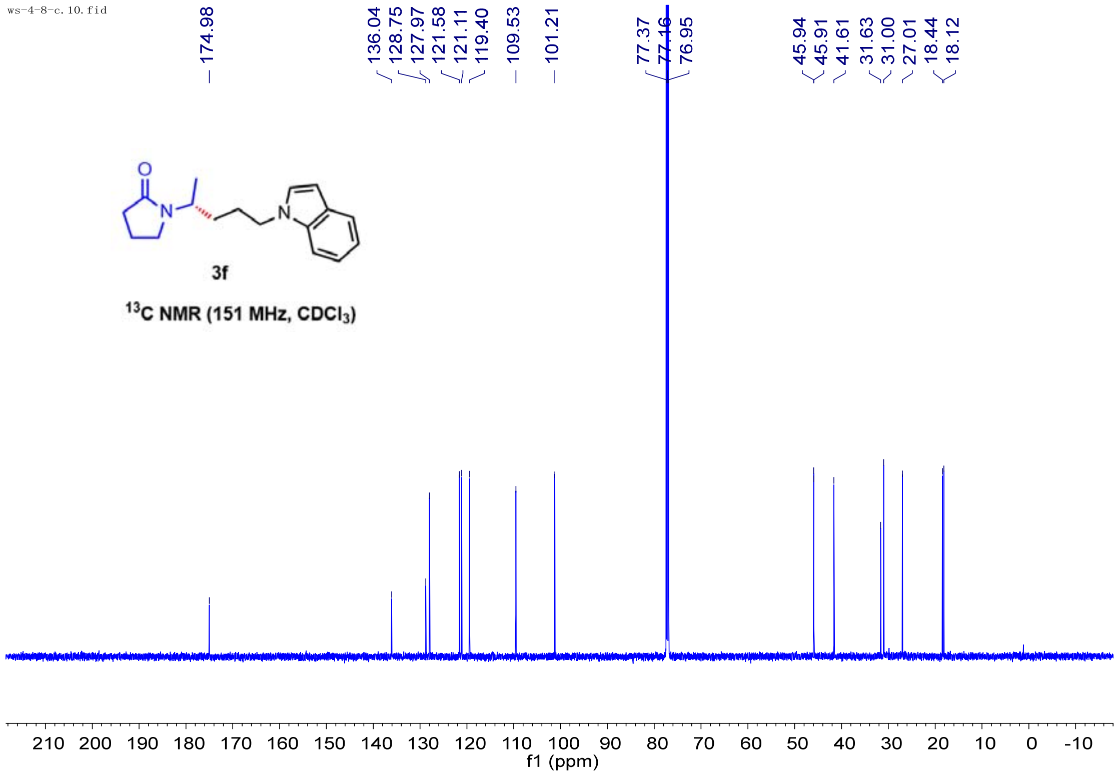

Supplementary Figure 23 ^{13}C NMR (151 MHz, CDCl$_3$) of **3f**

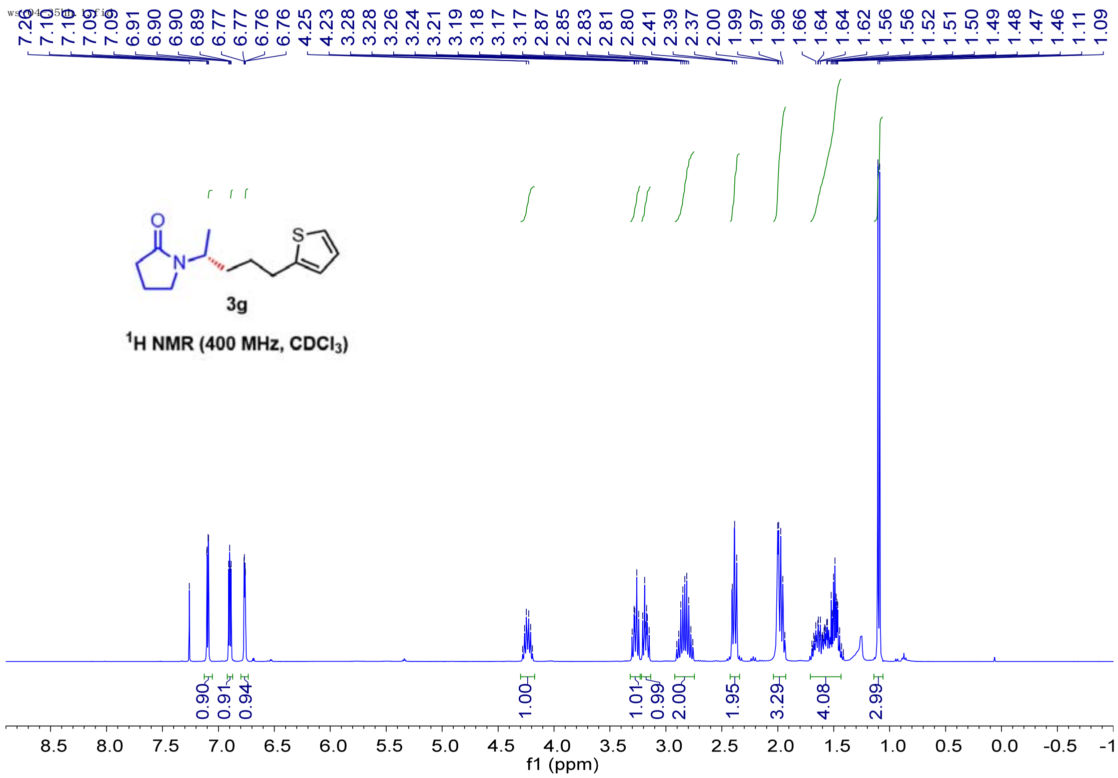

Supplementary Figure 24 ^{1}H NMR (400 MHz, $CDCl_3$) of **3g**

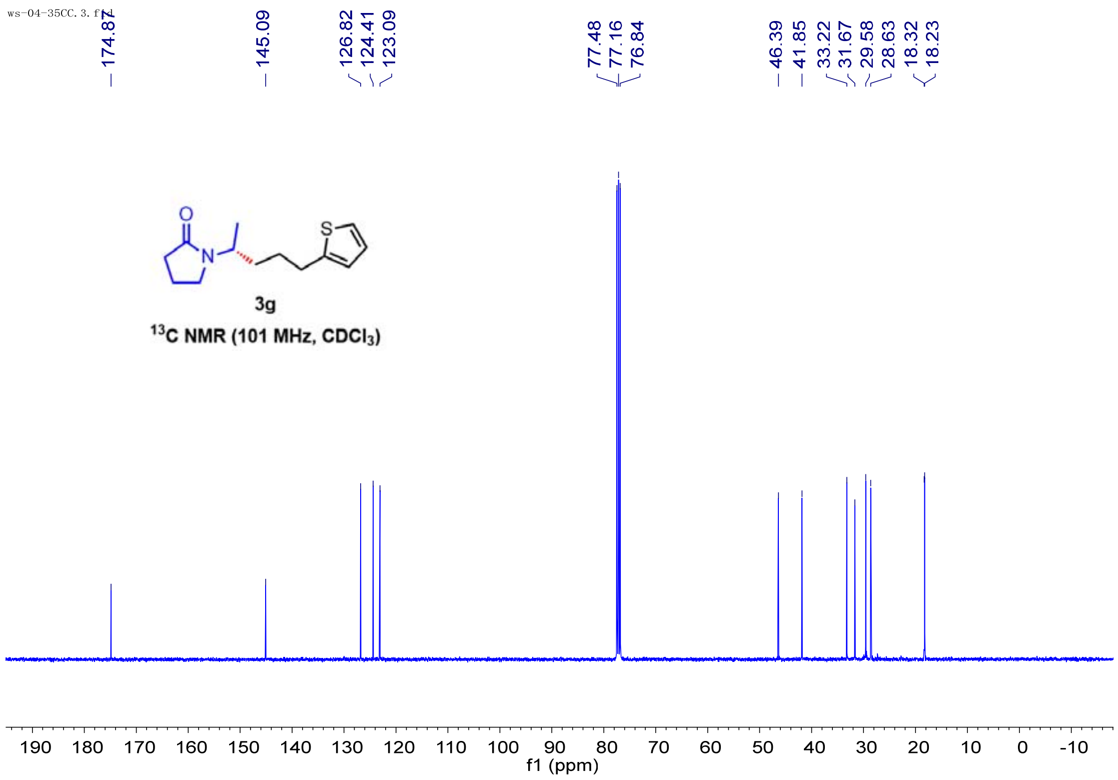

Supplementary Figure 25 ^{13}C NMR (101 MHz, $CDCl_3$) of **3g**

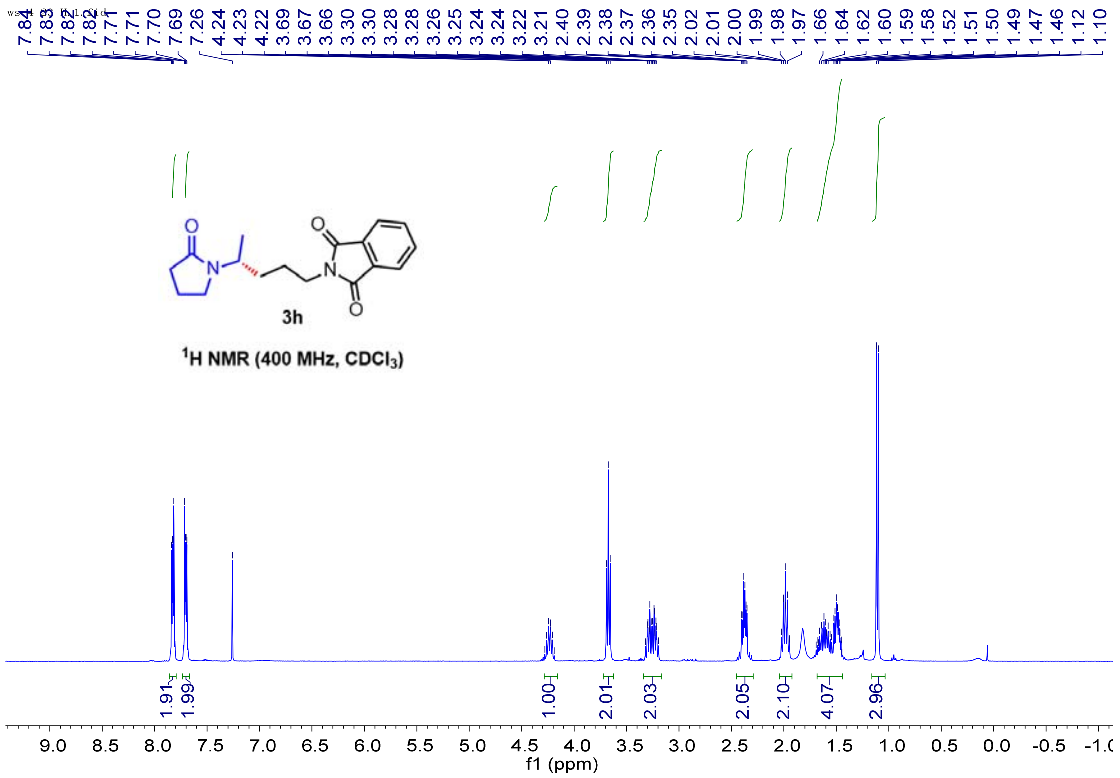

Supplementary Figure 26 ^{1}H NMR (400 MHz, $CDCl_3$) of **3h**

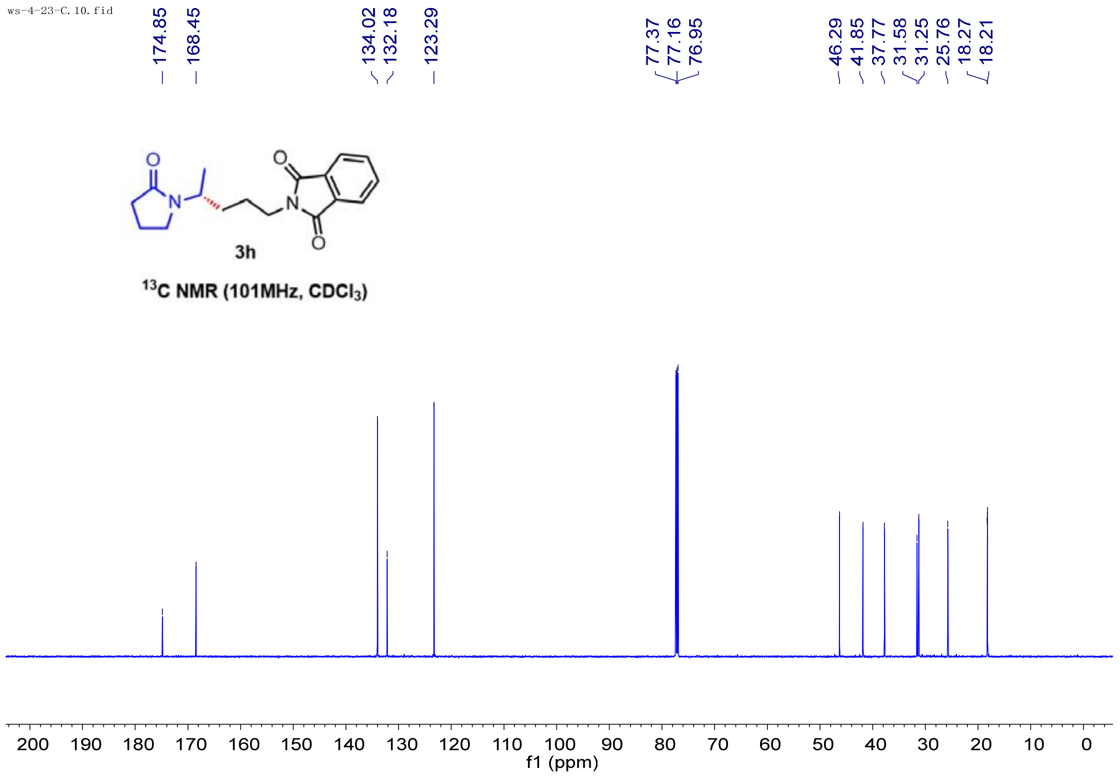

Supplementary Figure 27 ^{13}C NMR (101 MHz, $CDCl_3$) of **3h**

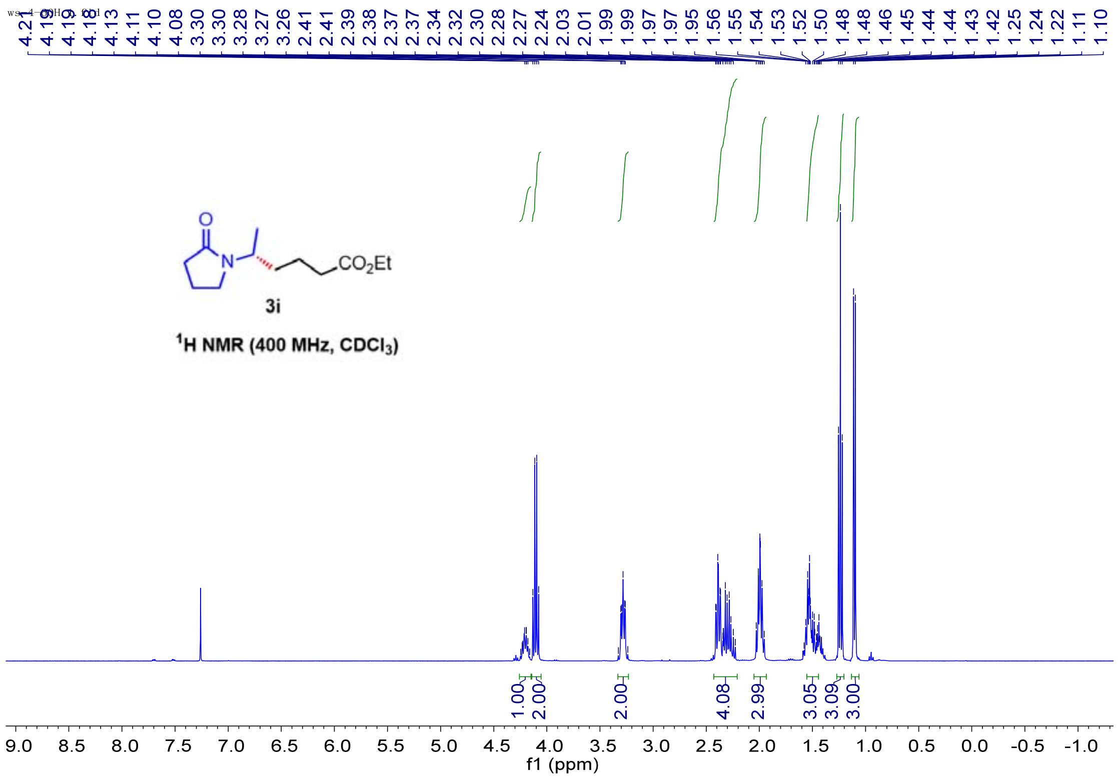

Supplementary Figure 28 ^{1}H NMR (400 MHz, $CDCl_3$) of **3i**

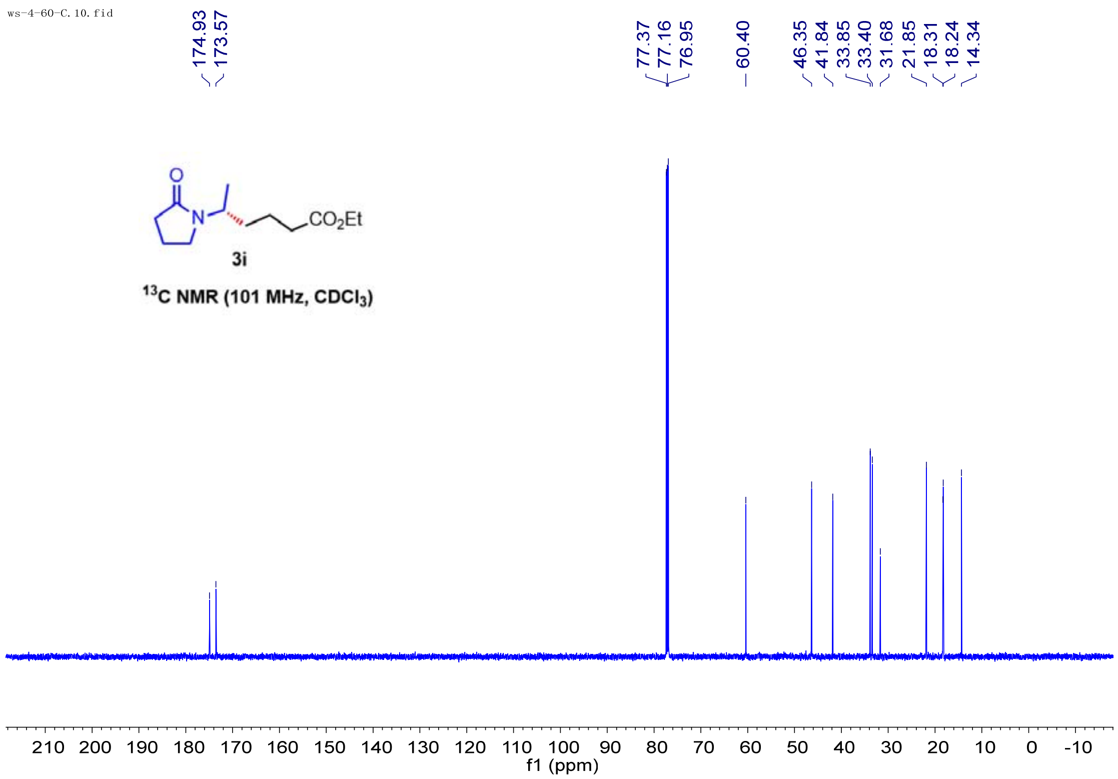

Supplementary Figure 29 ^{13}C NMR (101 MHz, $CDCl_3$) of **3i**

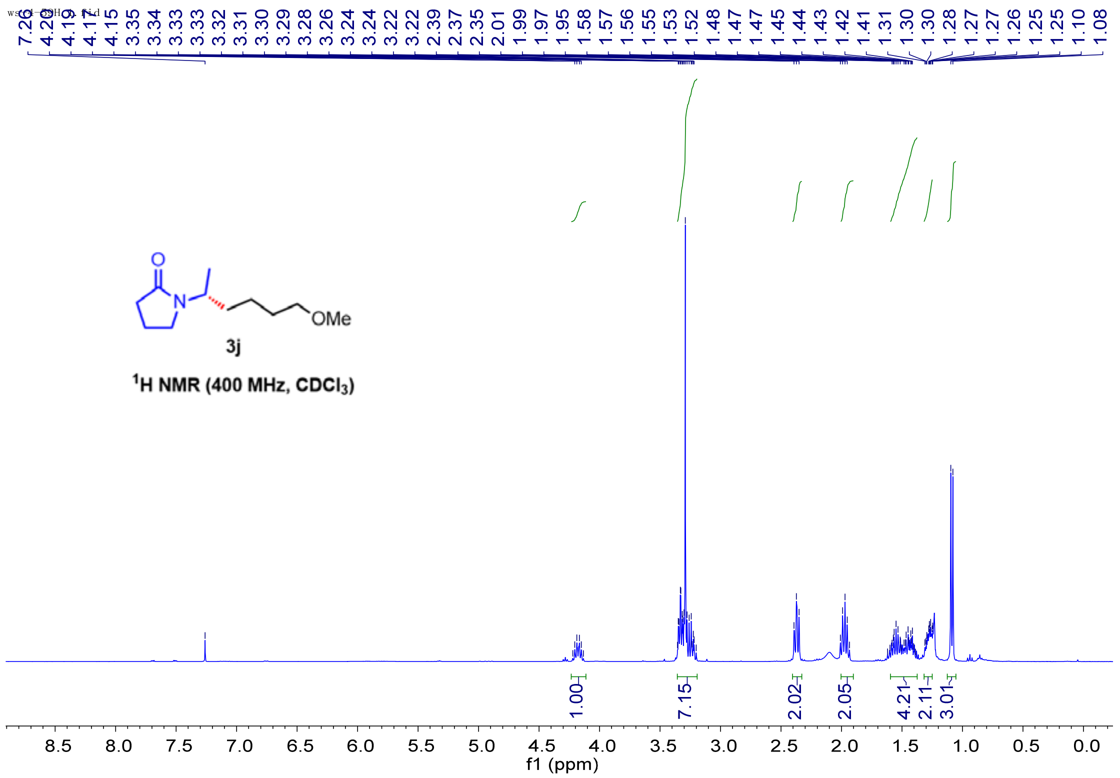

Supplementary Figure 30 ^{1}H NMR (400 MHz, $CDCl_3$) of **3j**

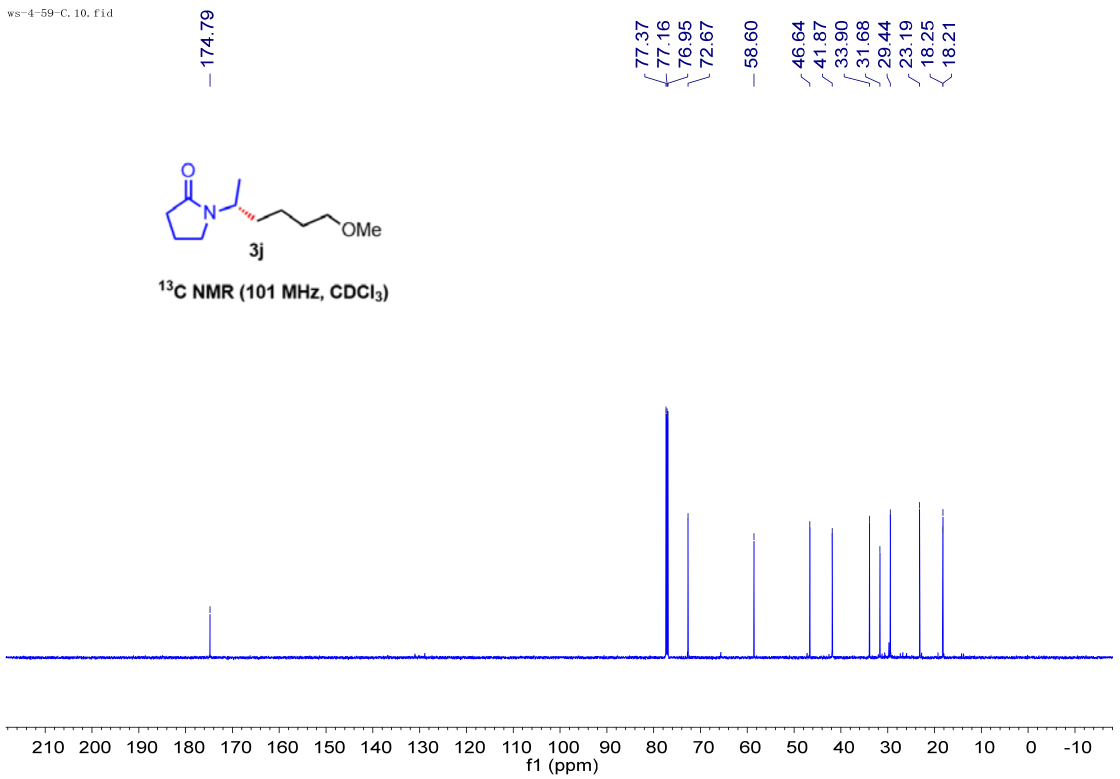

Supplementary Figure 31 ^{13}C NMR (101 MHz, $CDCl_3$) of **3j**

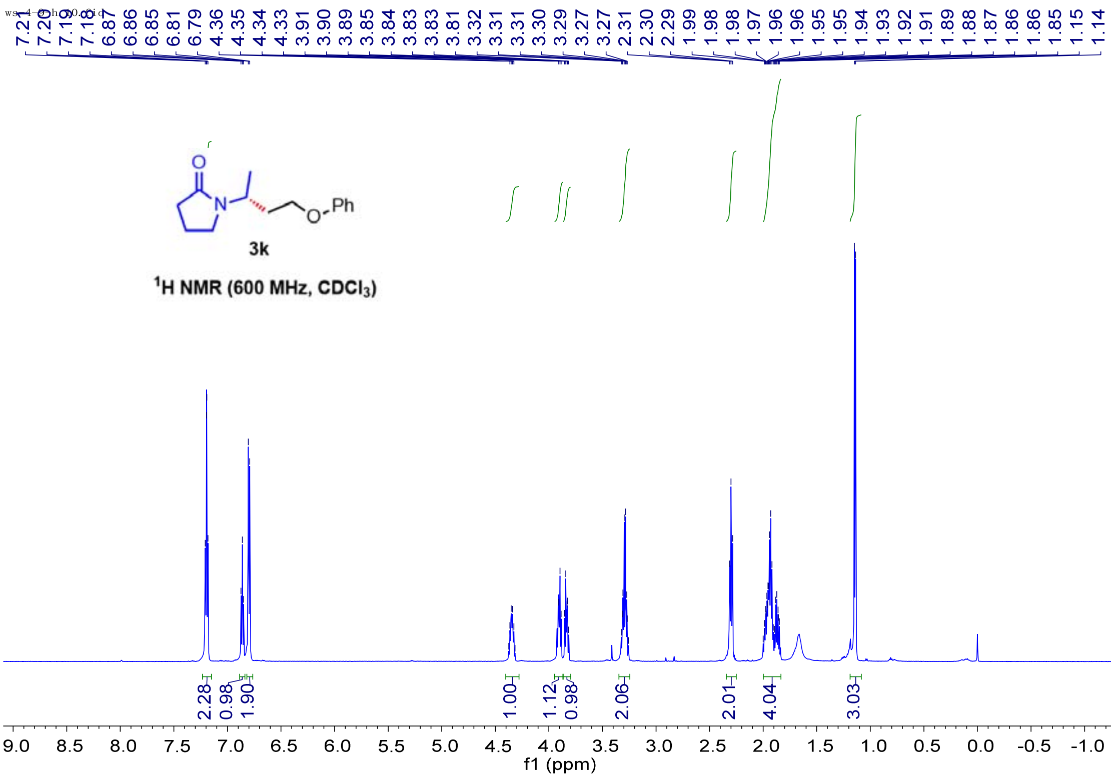

Supplementary Figure 32 ^{1}H NMR (600 MHz, $CDCl_3$) of **3k**

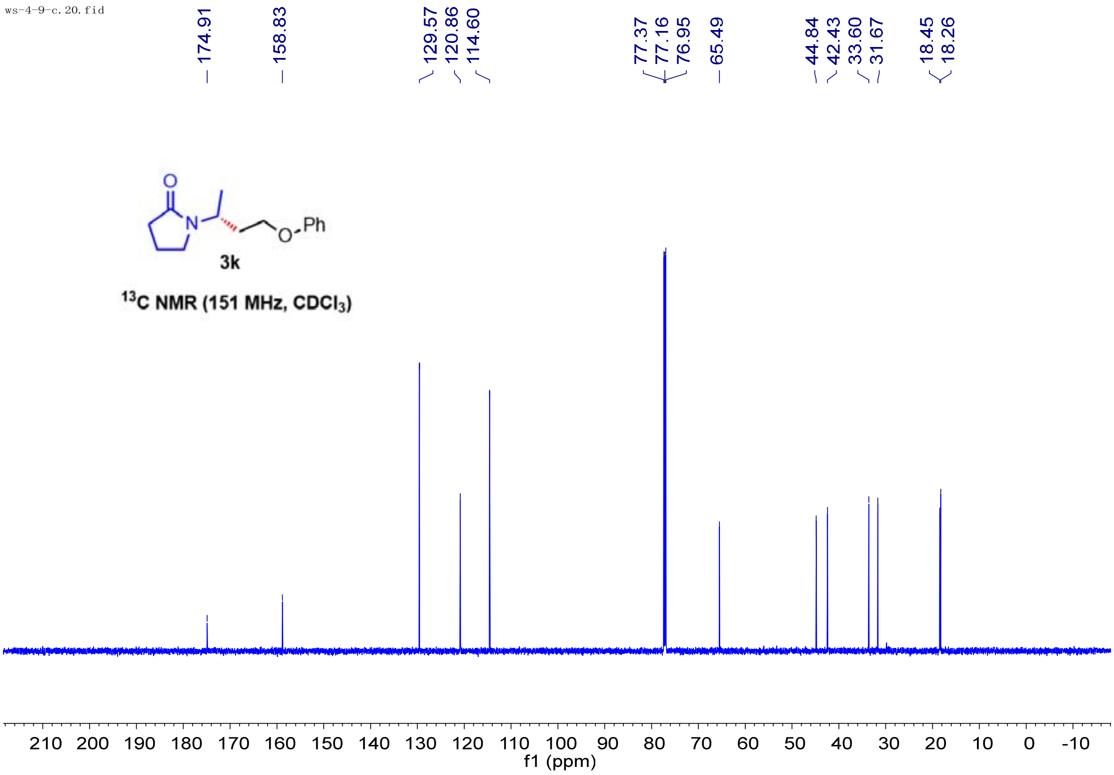

Supplementary Figure 33 ^{13}C NMR (151 MHz, $CDCl_3$) of **3k**

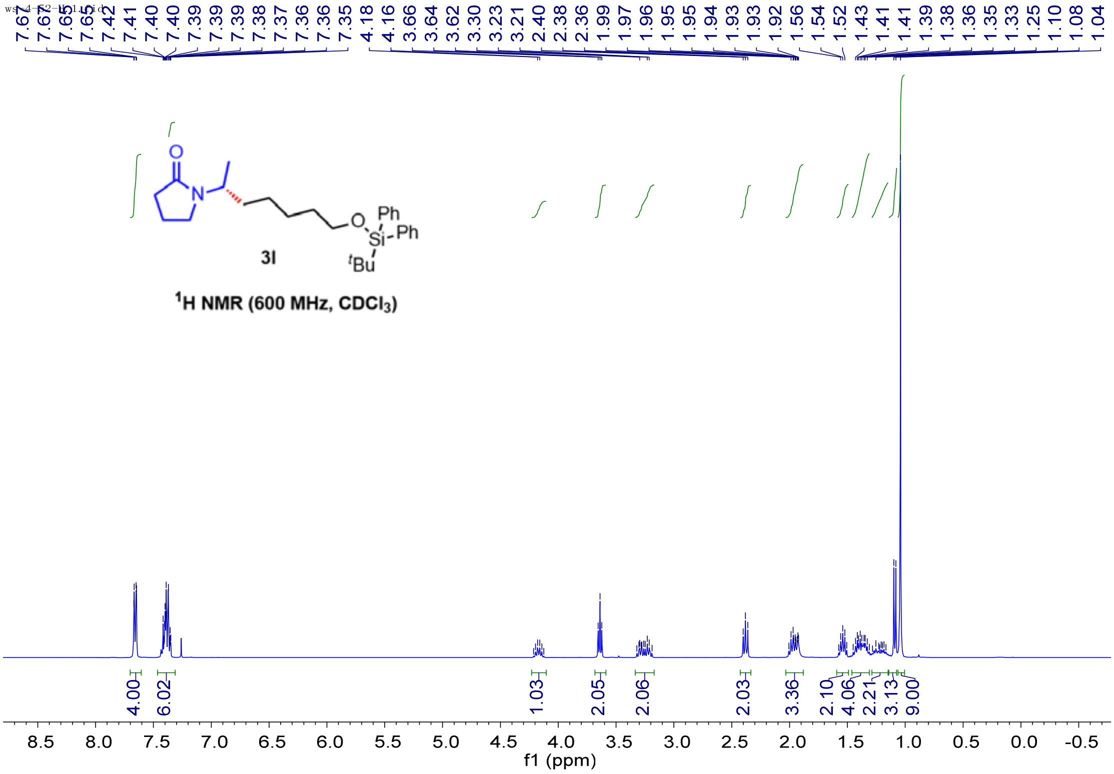

Supplementary Figure 34 ^{1}H NMR (600 MHz, $CDCl_3$) of **3l**

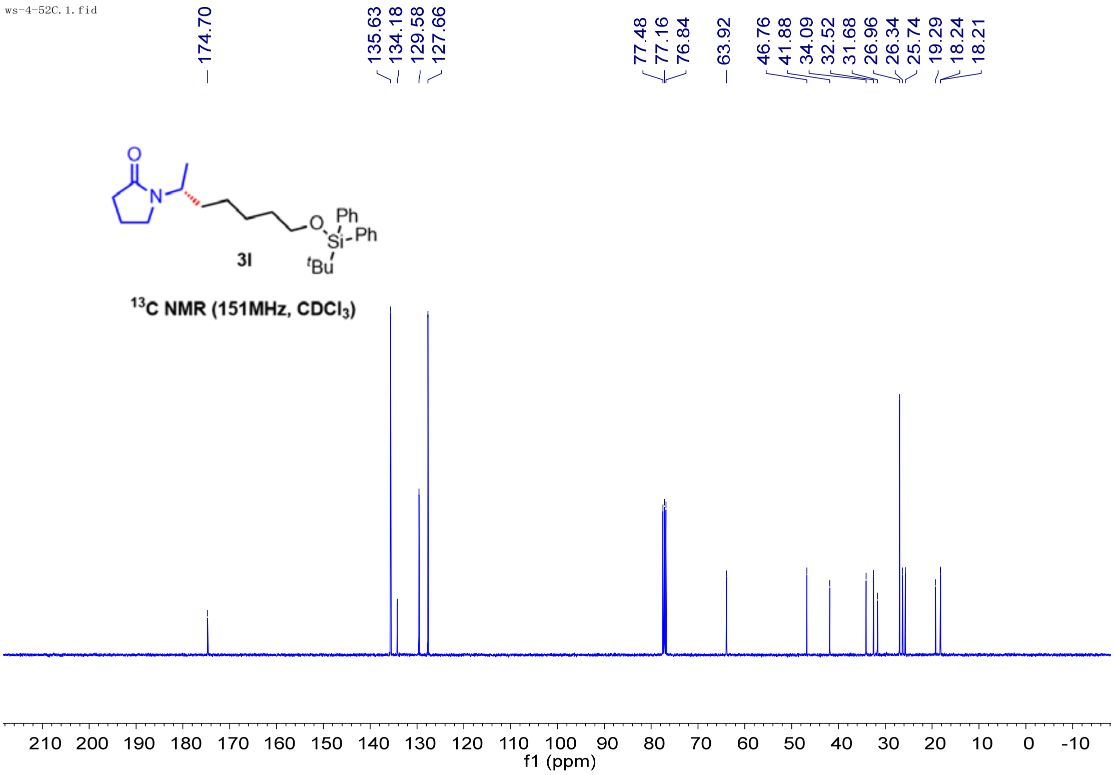

Supplementary Figure 35 ^{13}C NMR (151 MHz, $CDCl_3$) of **3l**

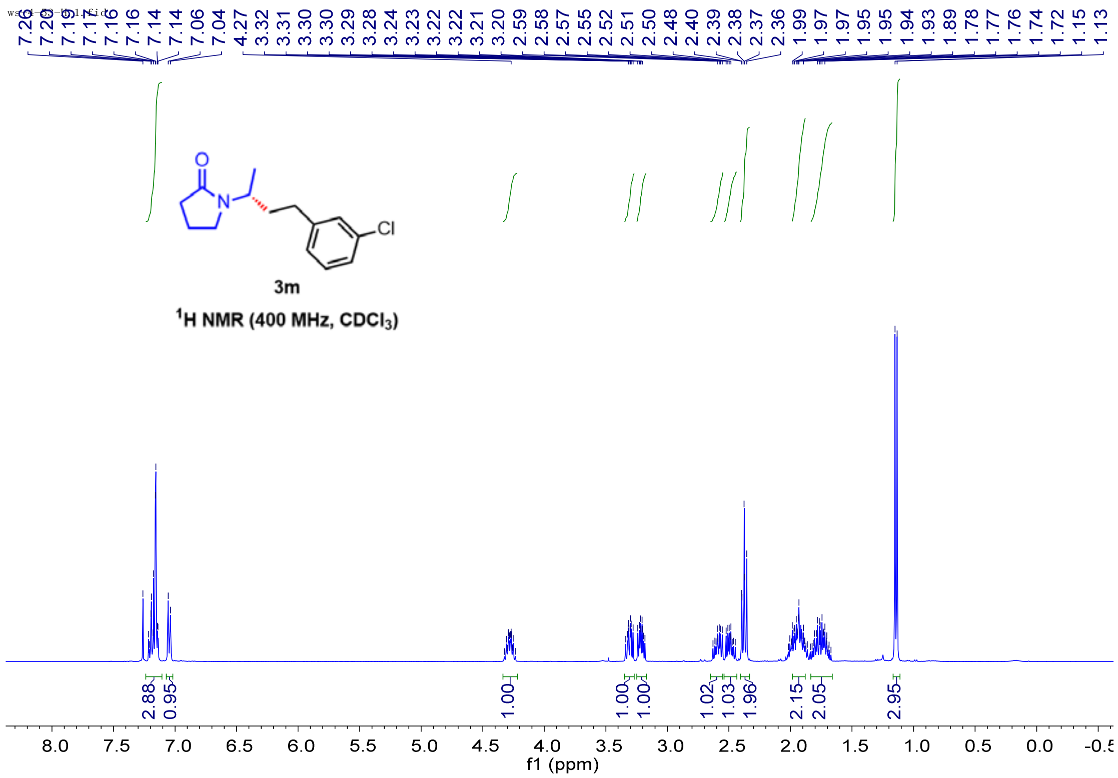

Supplementary Figure 36 ^{1}H NMR (400 MHz, CDCl$_3$) of **3m**

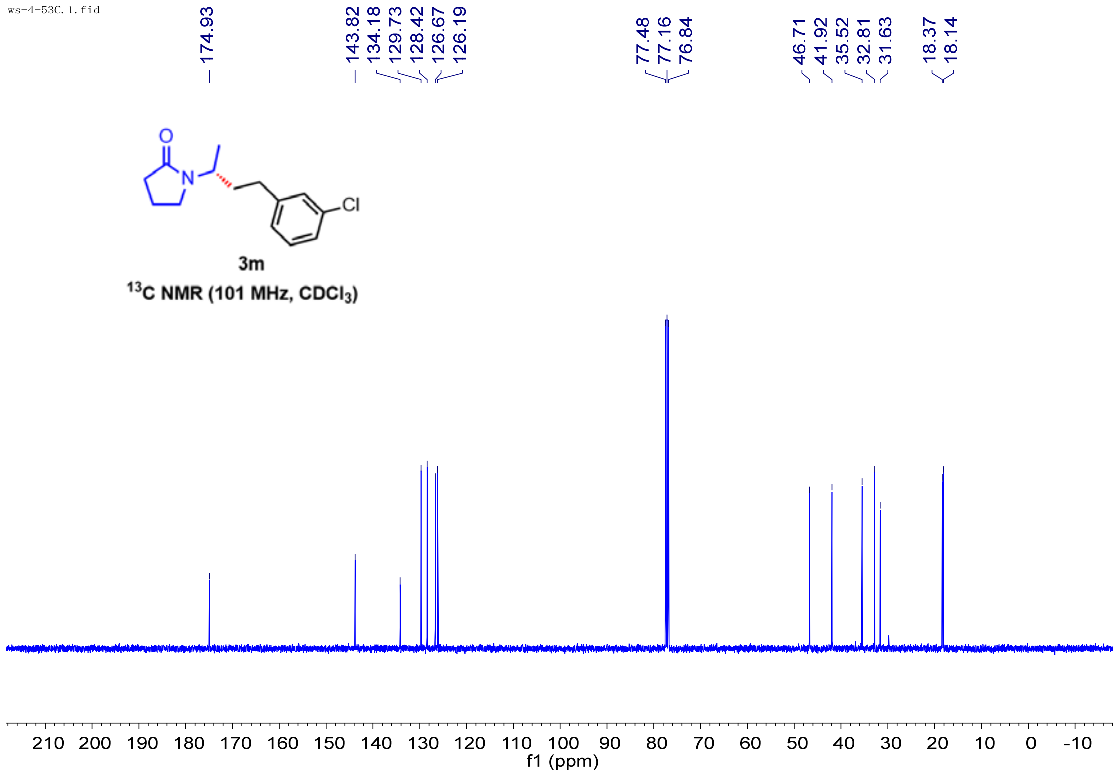

Supplementary Figure 37 ^{13}C NMR (101 MHz, CDCl$_3$) of **3m**

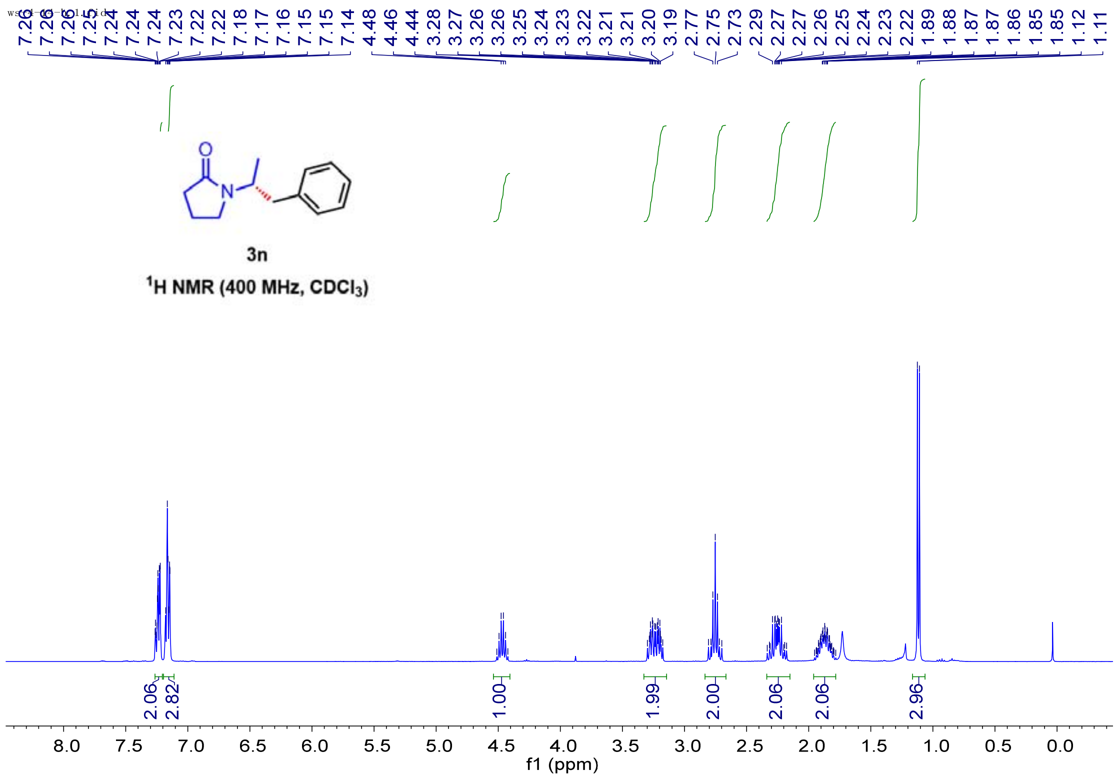

Supplementary Figure 38 ^{1}H NMR (400 MHz, $CDCl_3$) of **3n**

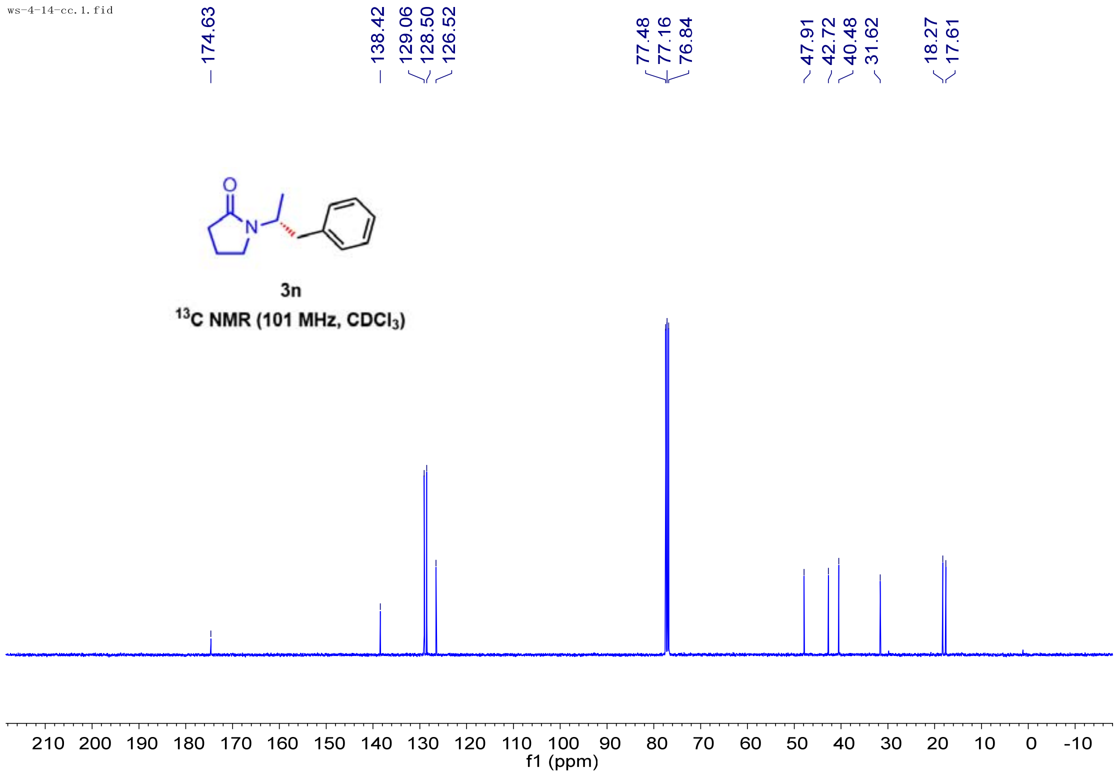

Supplementary Figure 39 ^{13}C NMR (101 MHz, $CDCl_3$) of **3n**

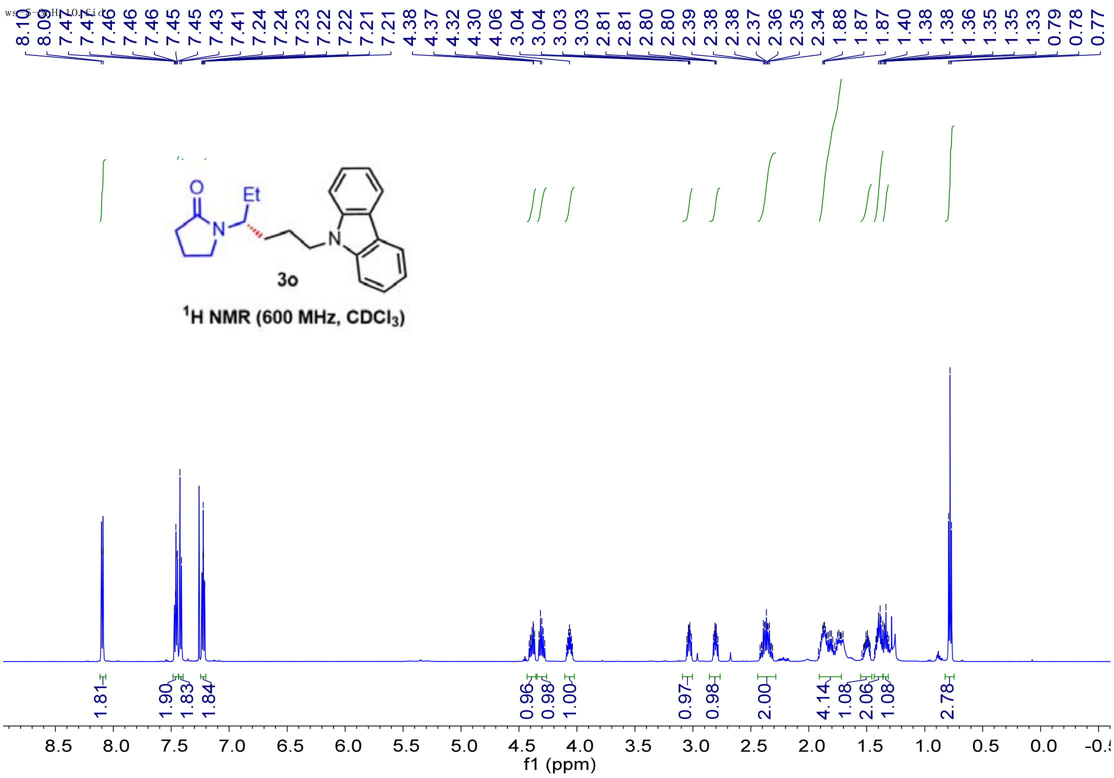

Supplementary Figure 40 ^{1}H NMR (600 MHz, $CDCl_3$) of **3o**

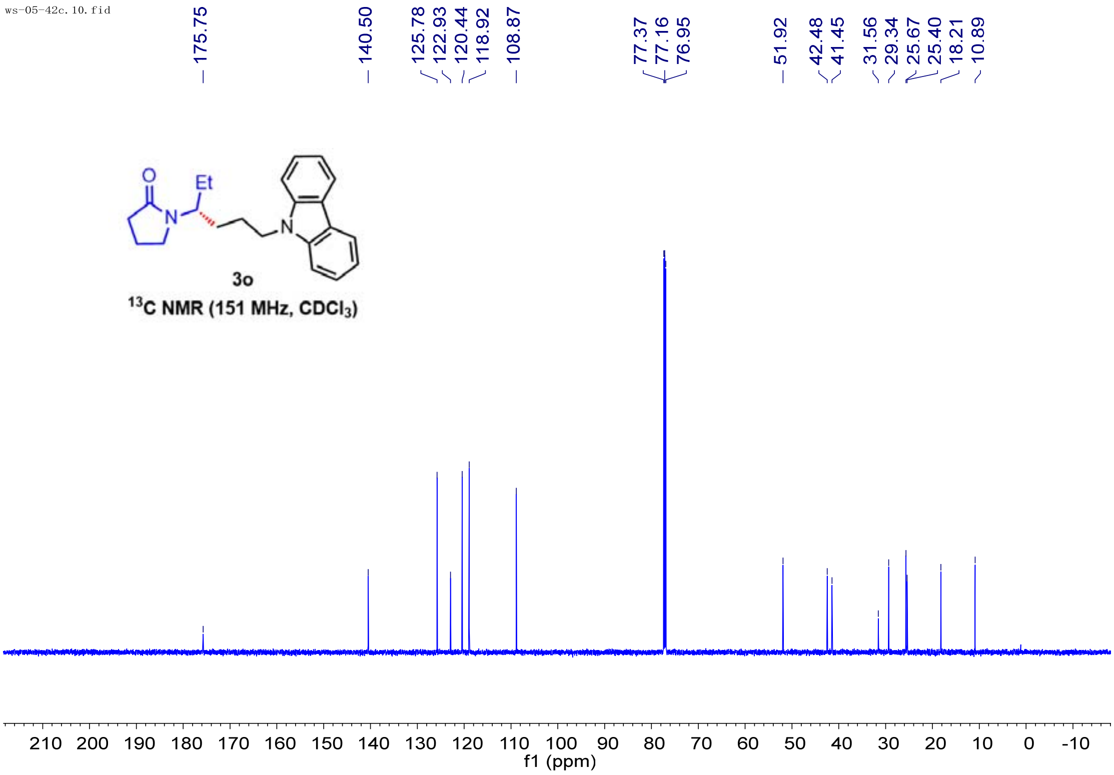

Supplementary Figure 41 ^{13}C NMR (151 MHz, $CDCl_3$) of **3o**

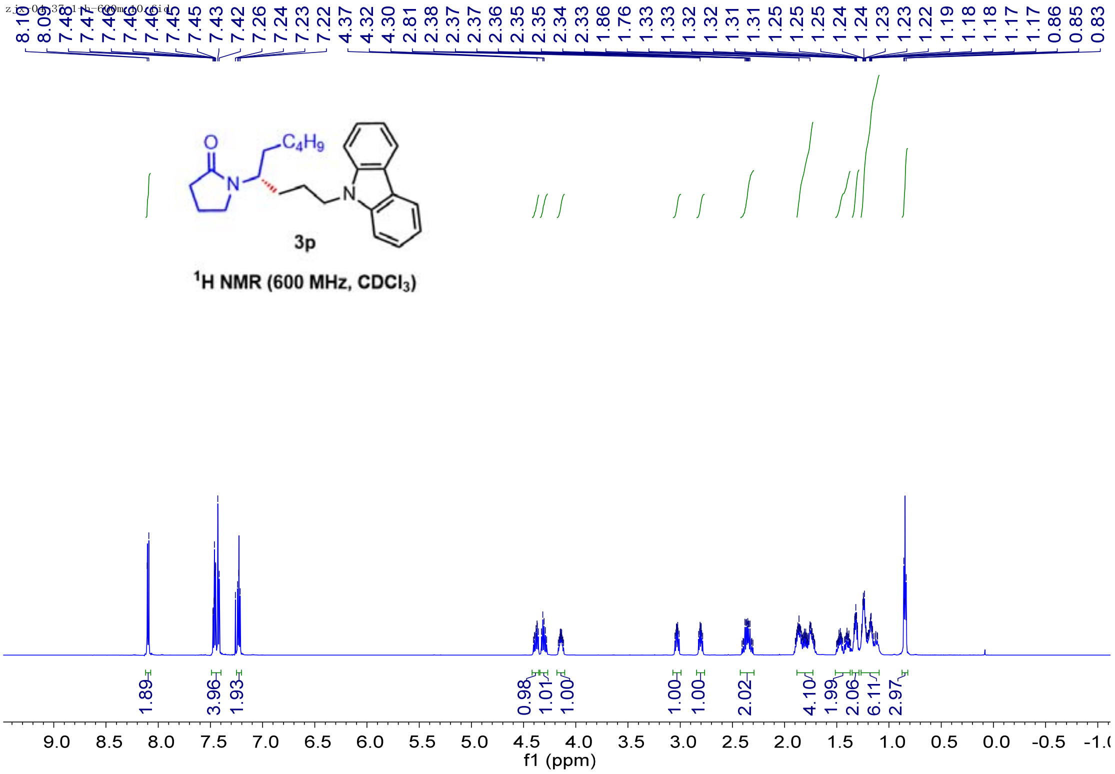

Supplementary Figure 42 ^{1}H NMR (600 MHz, $CDCl_3$) of **3p**

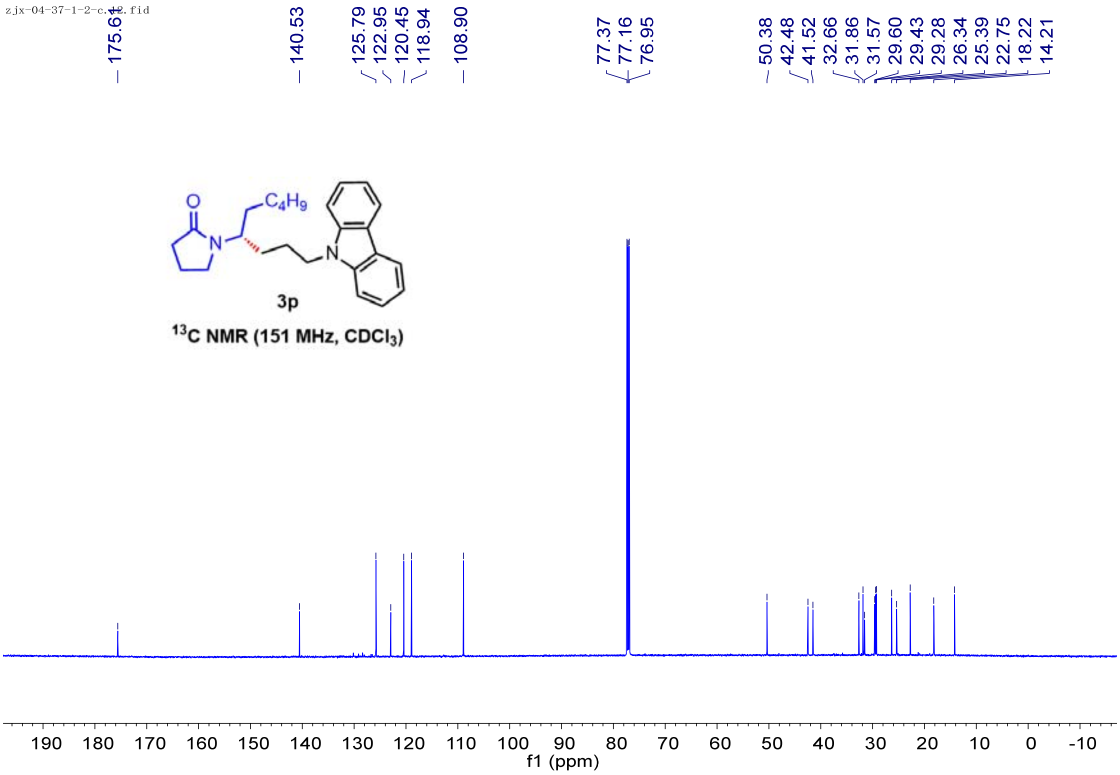

Supplementary Figure 43 ^{13}C NMR (151 MHz, $CDCl_3$) of **3p**

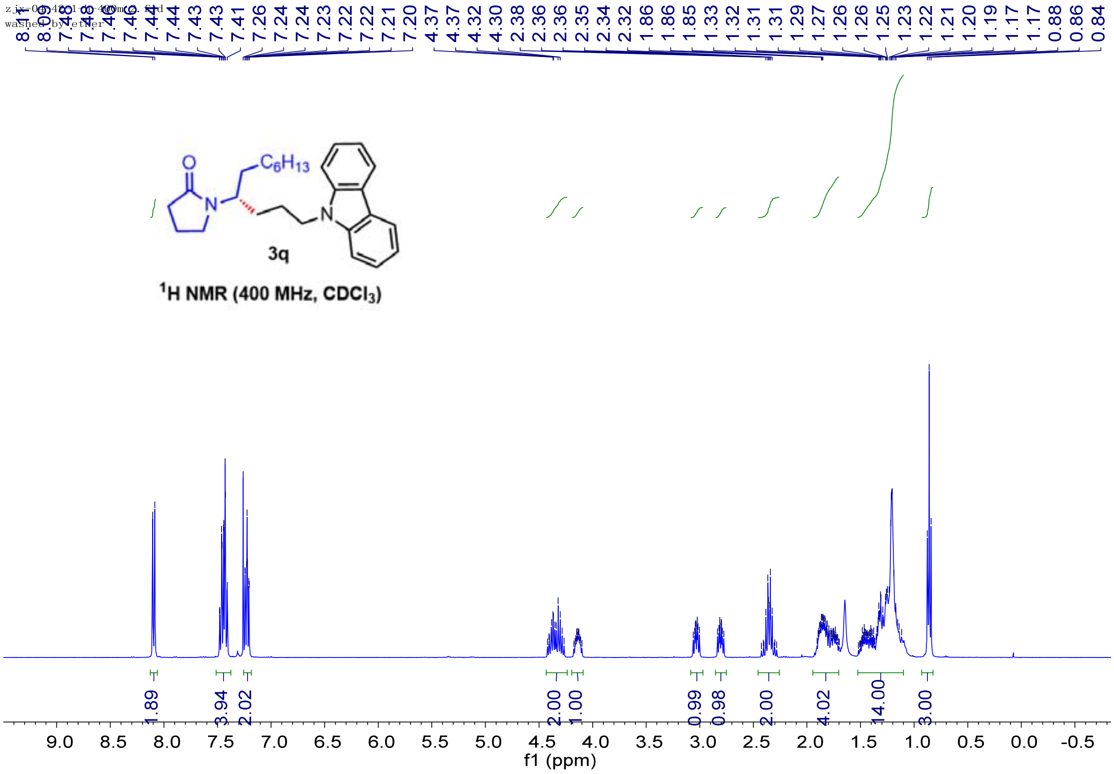

Supplementary Figure 44 ^{1}H NMR (400 MHz, $CDCl_3$) of **3q**

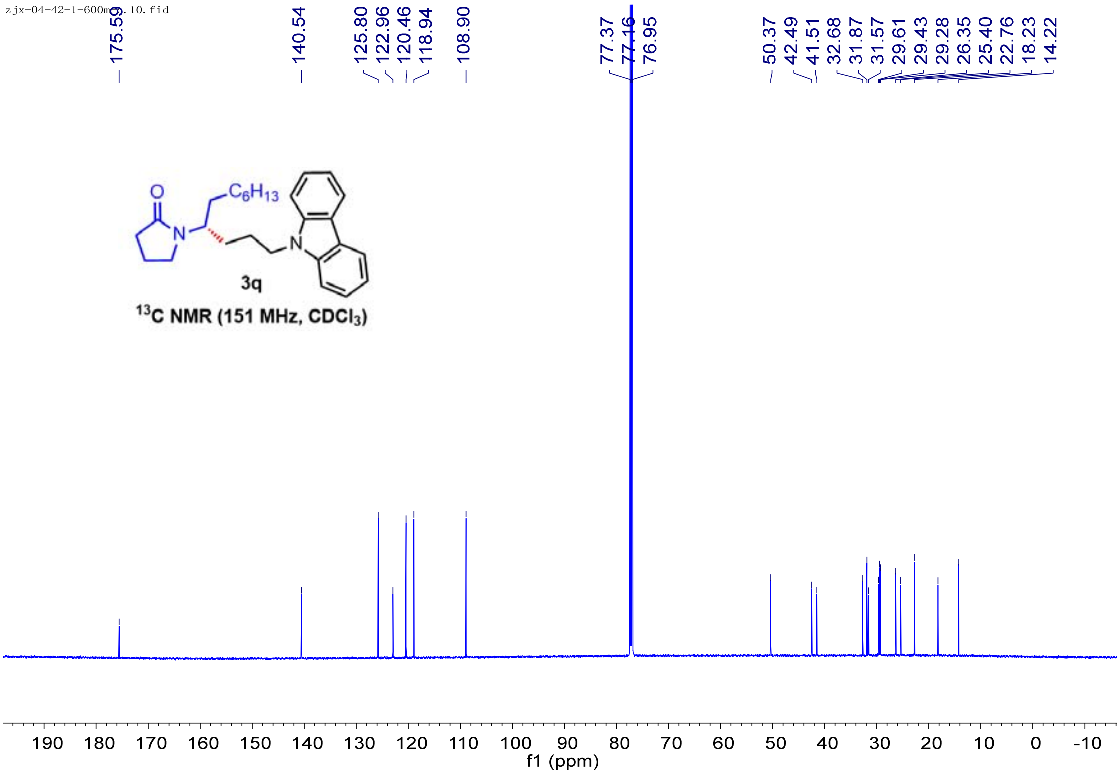

Supplementary Figure 45 ^{13}C NMR (151 MHz, $CDCl_3$) of **3q**

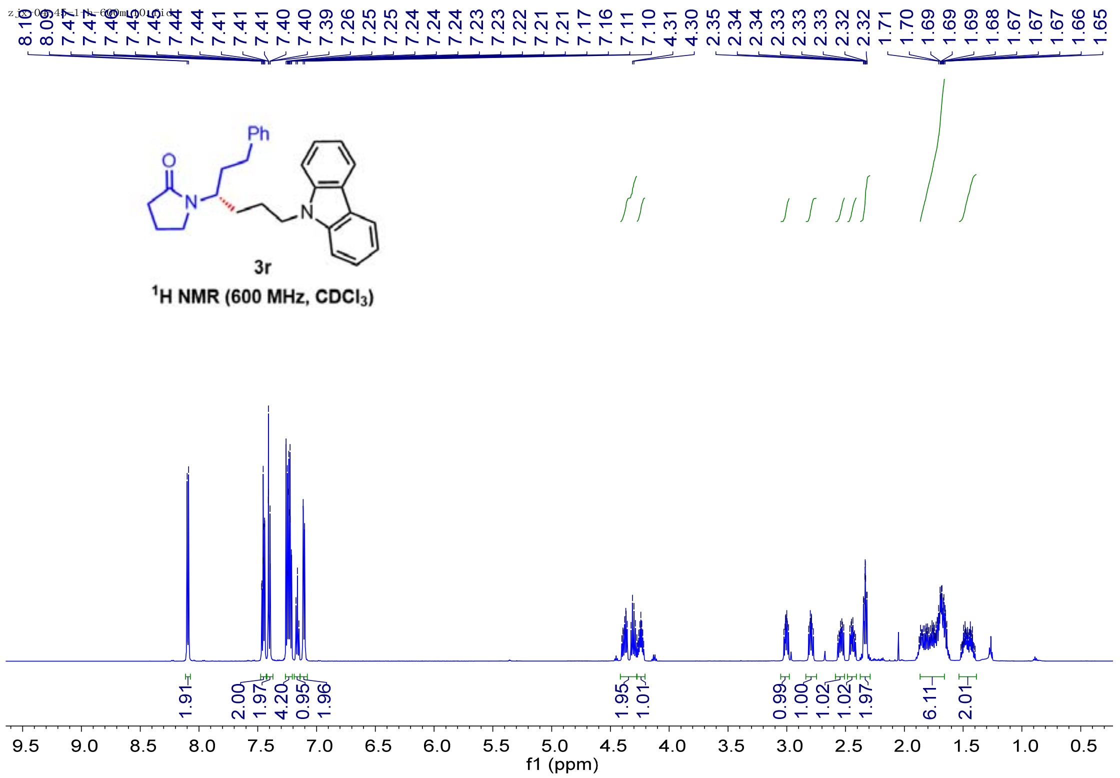

Supplementary Figure 46 ^{1}H NMR (600 MHz, $CDCl_3$) of **3r**

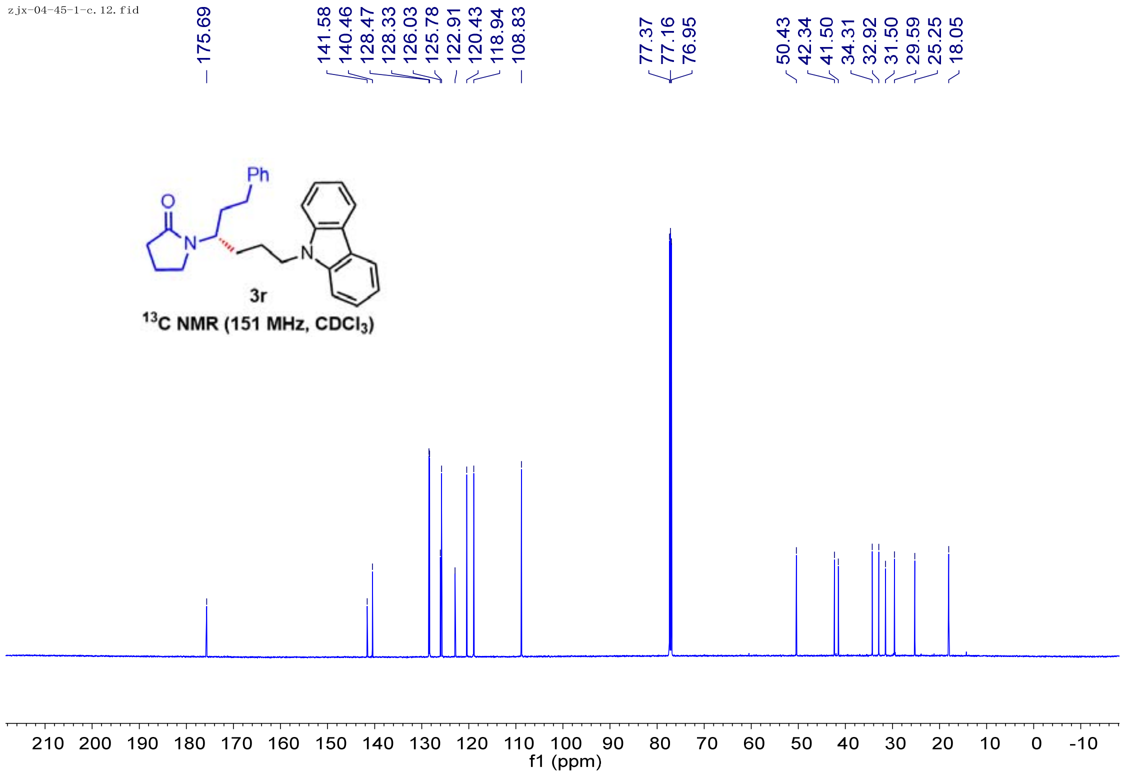

Supplementary Figure 47 ^{13}C NMR (151 MHz, $CDCl_3$) of **3r**

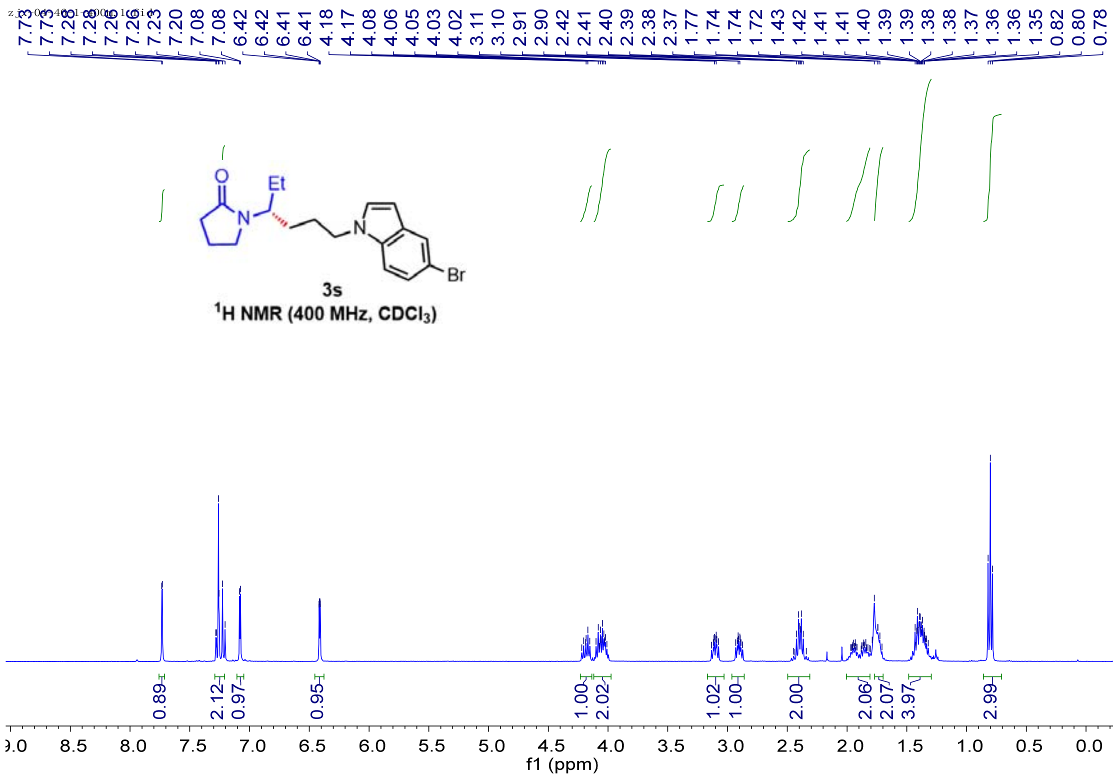

Supplementary Figure 48 ^{1}H NMR (400 MHz, $CDCl_3$) of **3s**

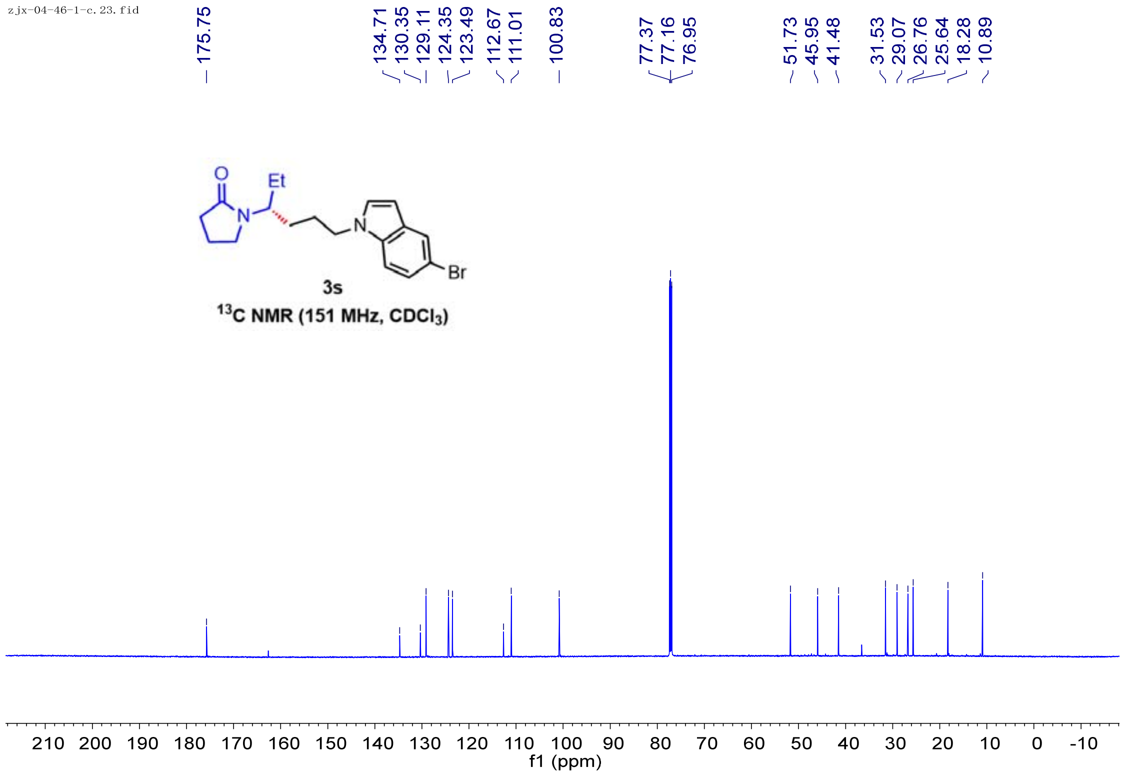

Supplementary Figure 49 ^{13}C NMR (151 MHz, $CDCl_3$) of **3s**

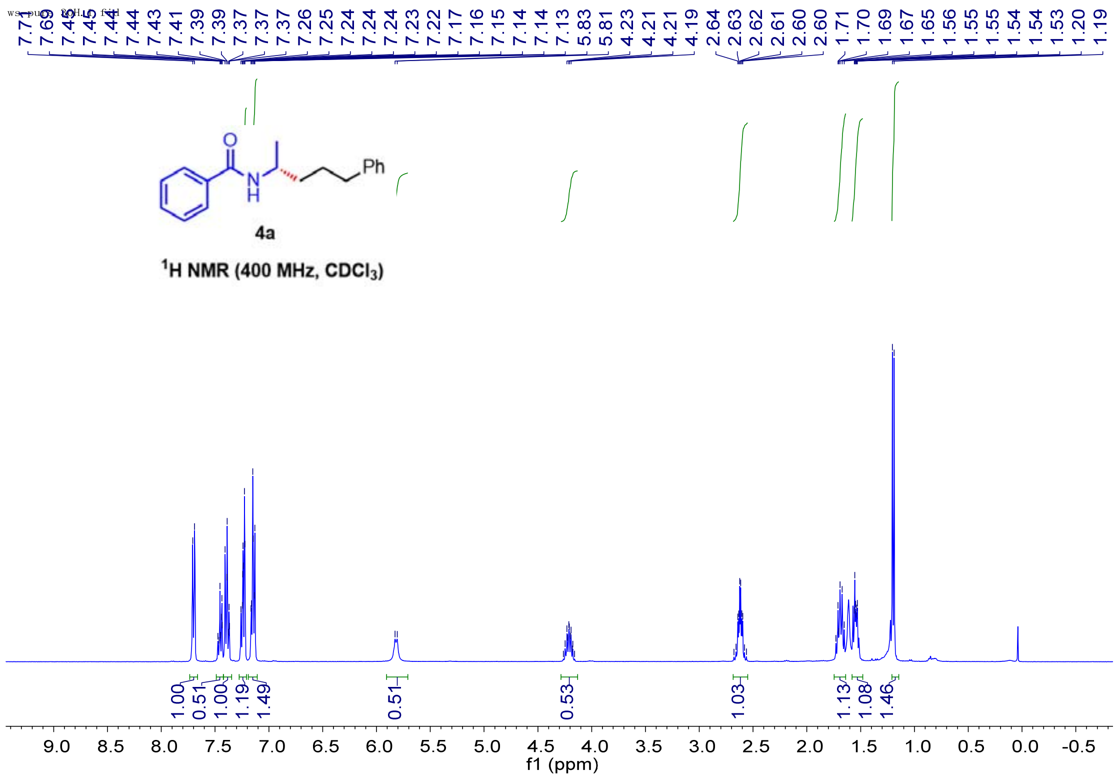

Supplementary Figure 50 ^{1}H NMR (400 MHz, $CDCl_3$) of **4a**

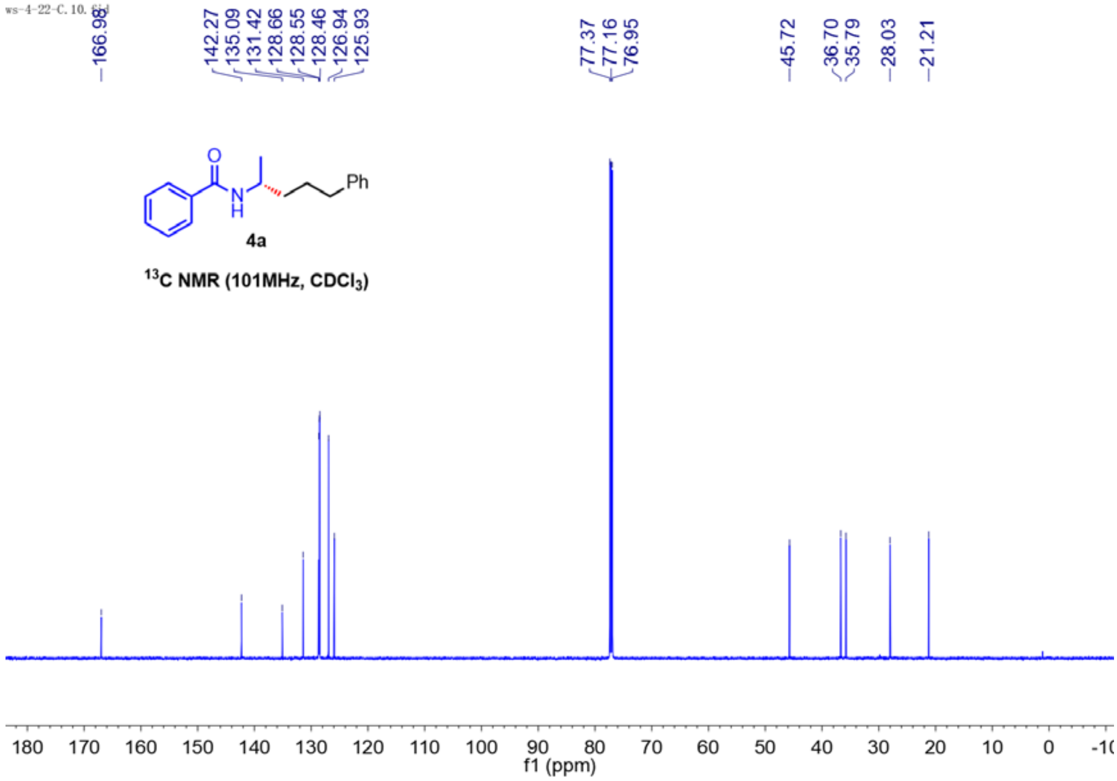

Supplementary Figure 51 ^{13}C NMR (101 MHz, $CDCl_3$) of **4a**

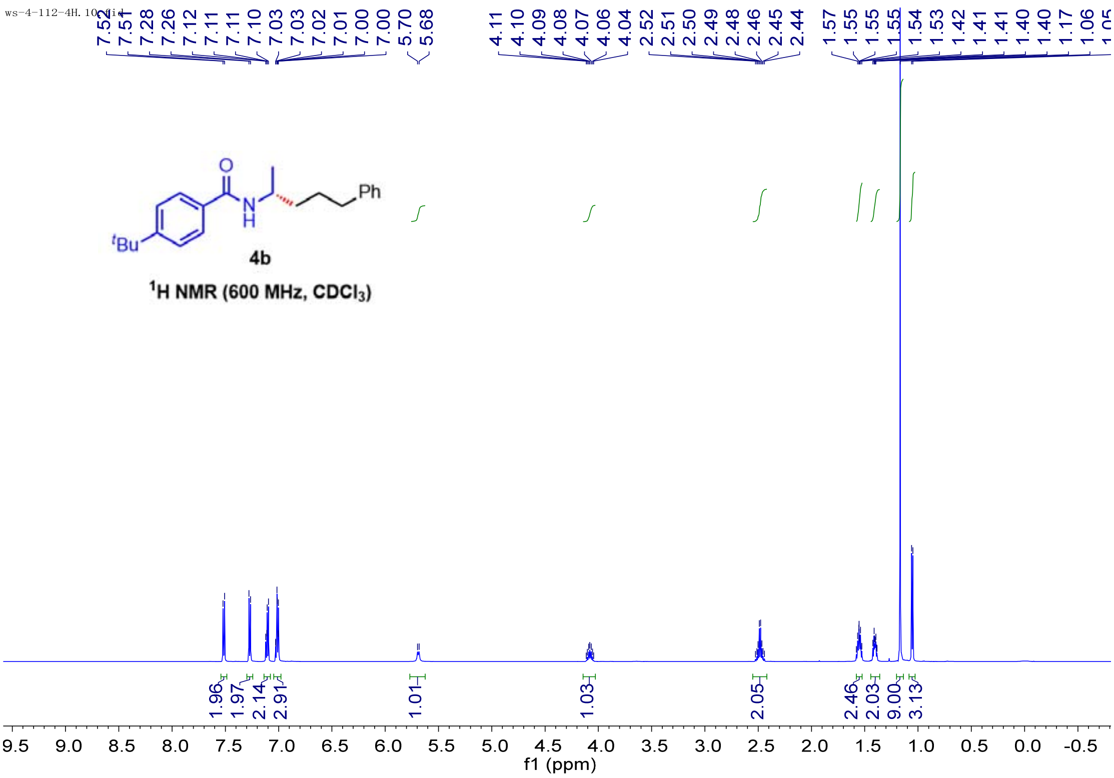

Supplementary Figure 52 ^{1}H NMR (600 MHz, $CDCl_3$) of **4b**

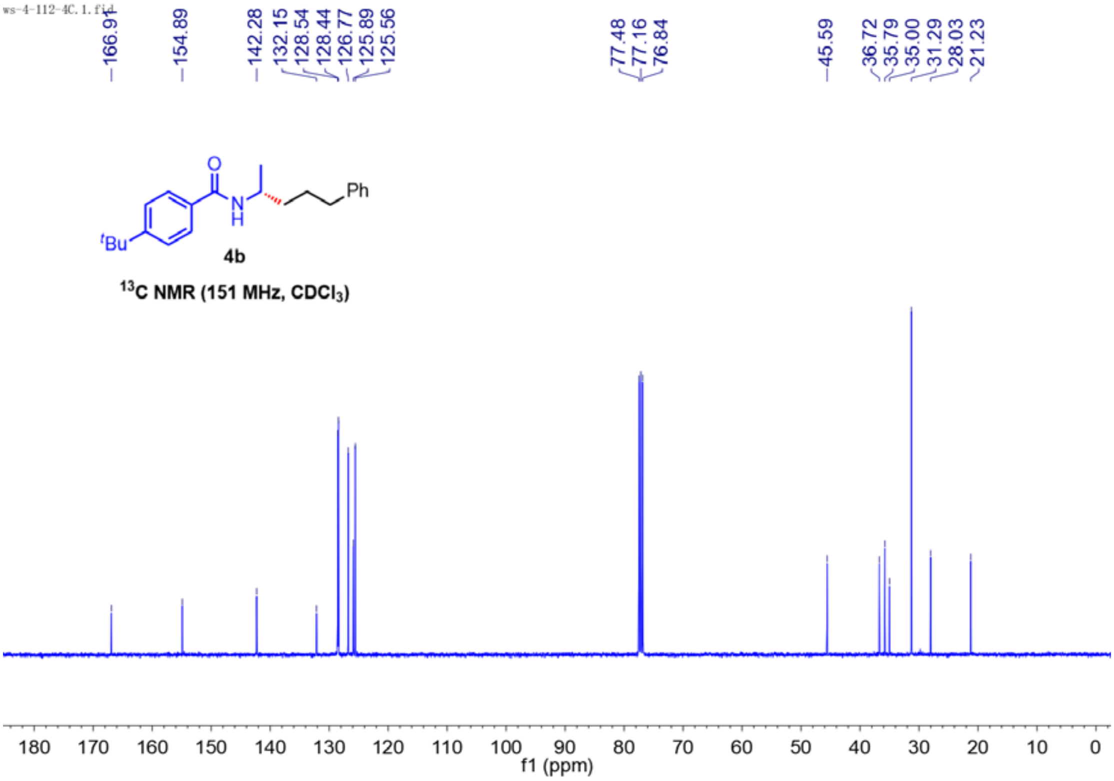

Supplementary Figure 53 ^{13}C NMR (151 MHz, $CDCl_3$) of **4b**

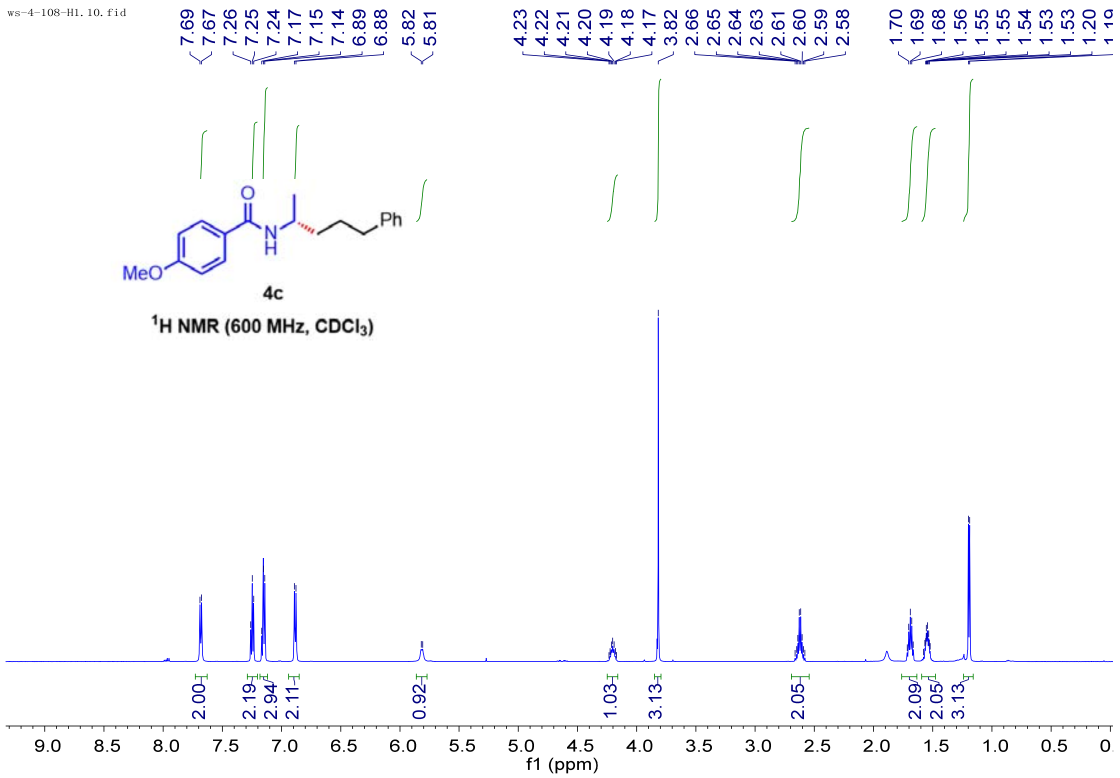

Supplementary Figure 54 ^{1}H NMR (600 MHz, $CDCl_3$) of **4c**

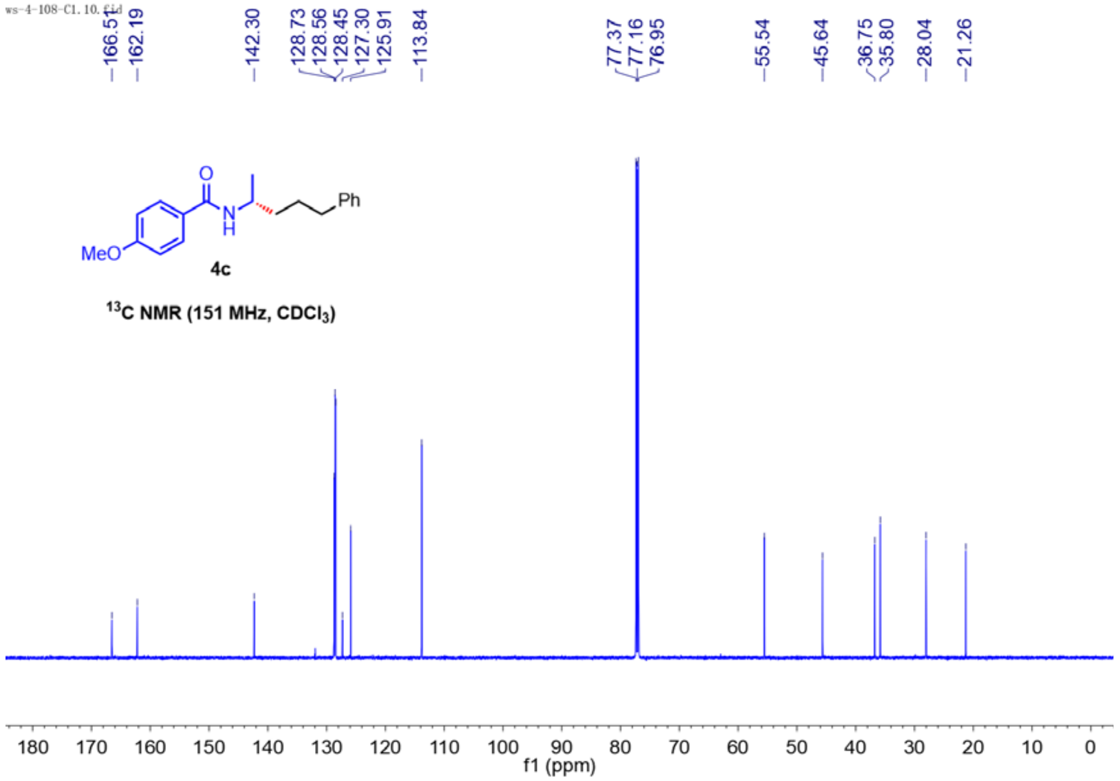

Supplementary Figure 55 ^{13}C NMR (151 MHz, $CDCl_3$) of **4c**

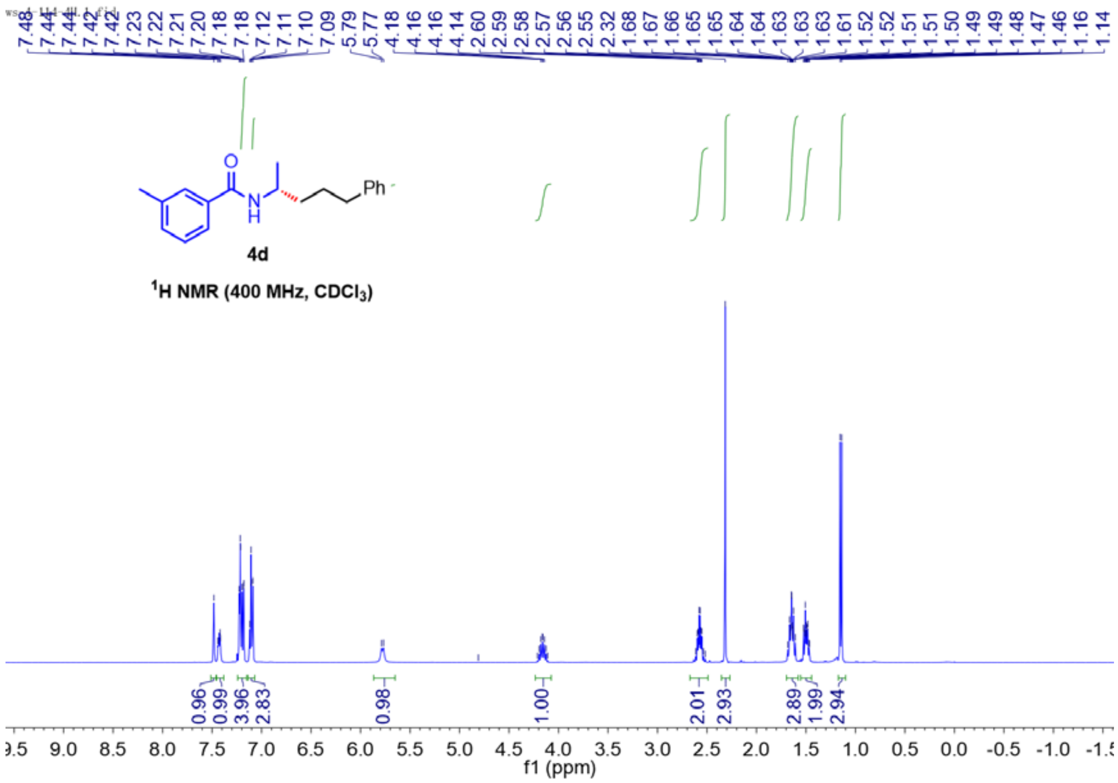

Supplementary Figure 56 ^{1}H NMR (400 MHz, $CDCl_3$) of **4d**

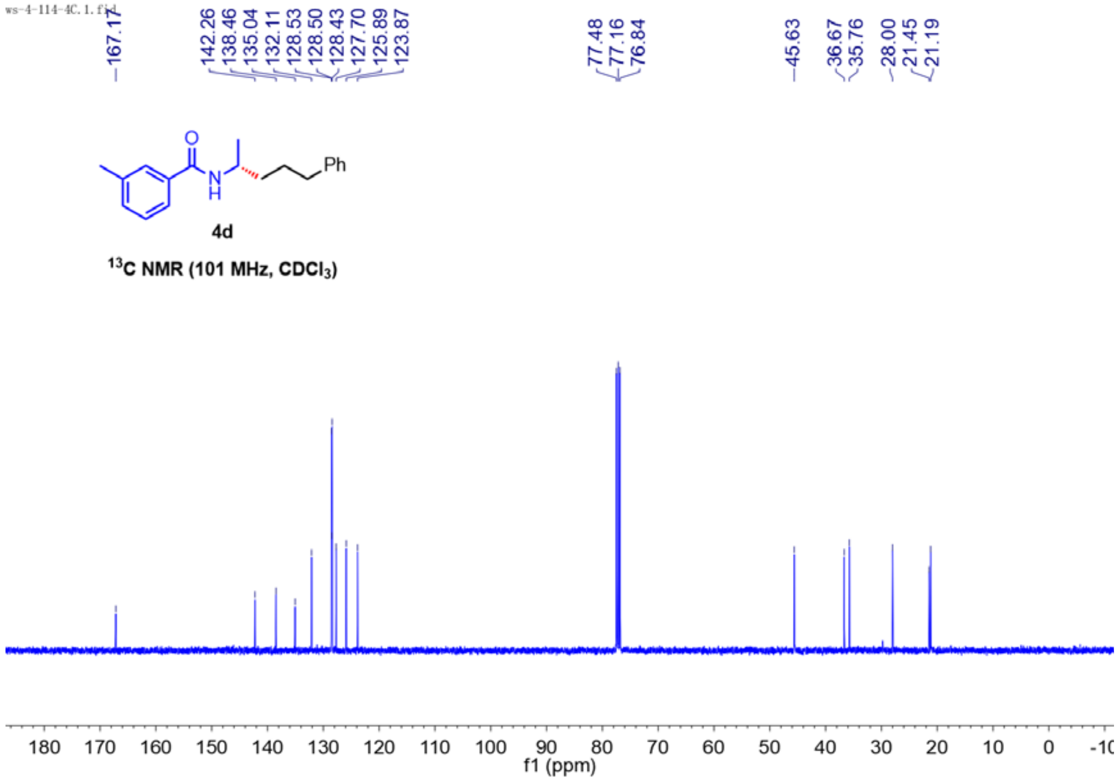

Supplementary Figure 57 ^{13}C NMR (101 MHz, $CDCl_3$) of **4d**

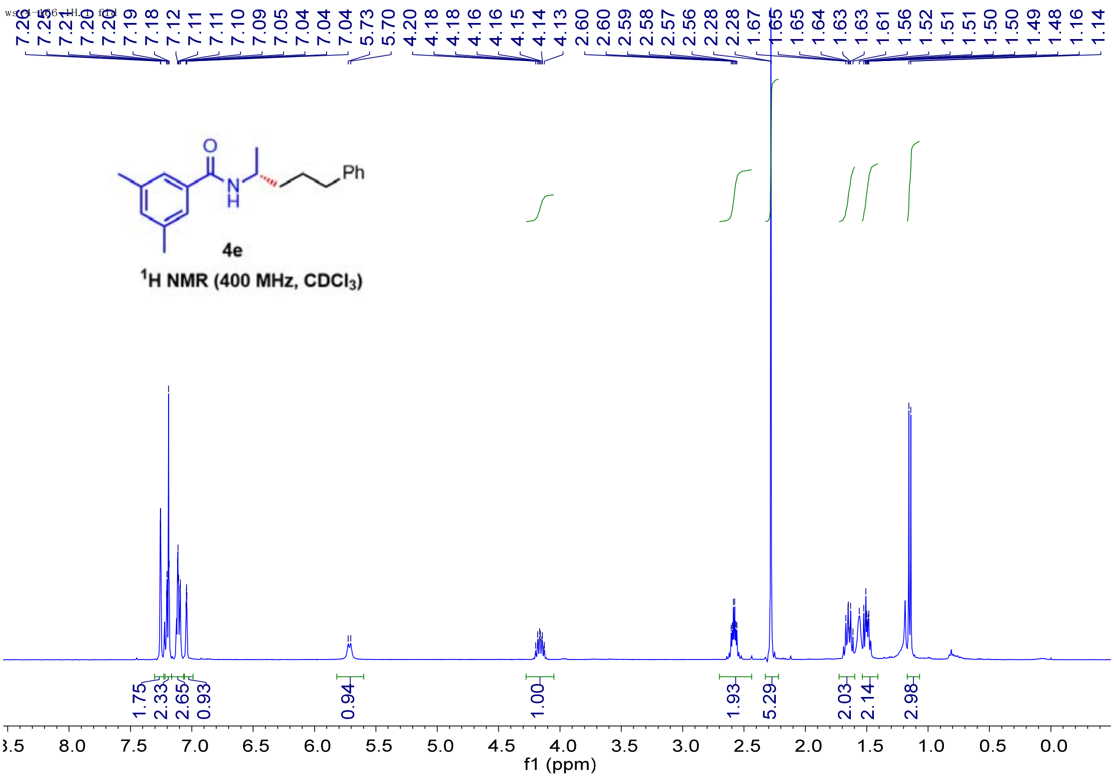

Supplementary Figure 58 ^{1}H NMR (400 MHz, $CDCl_3$) of **4e**

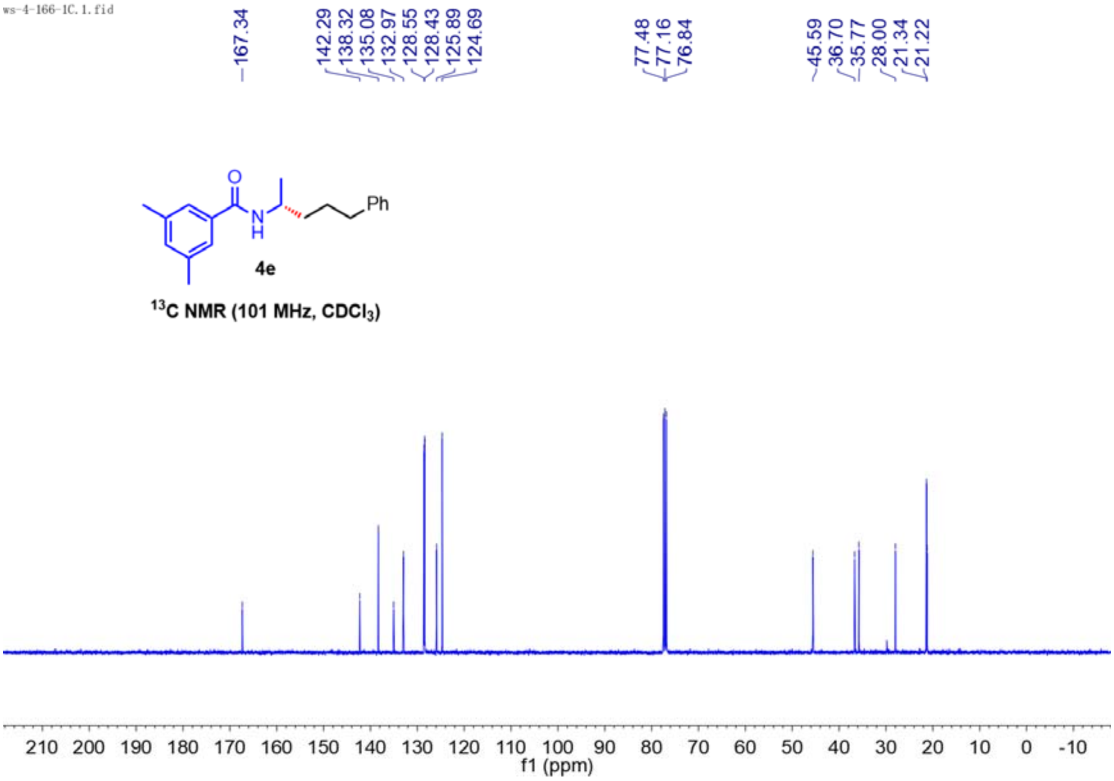

Supplementary Figure 59 ^{13}C NMR (101 MHz, $CDCl_3$) of **4e**

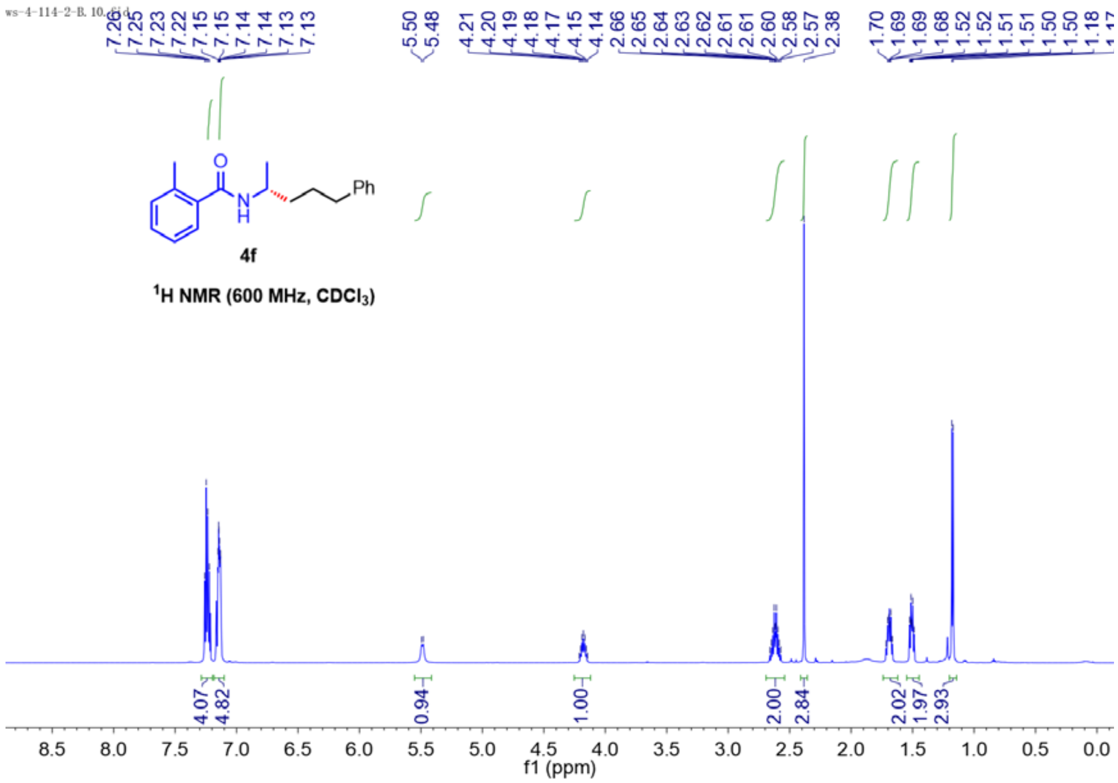

Supplementary Figure 60 ^{1}H NMR (600 MHz, $CDCl_3$) of **4f**

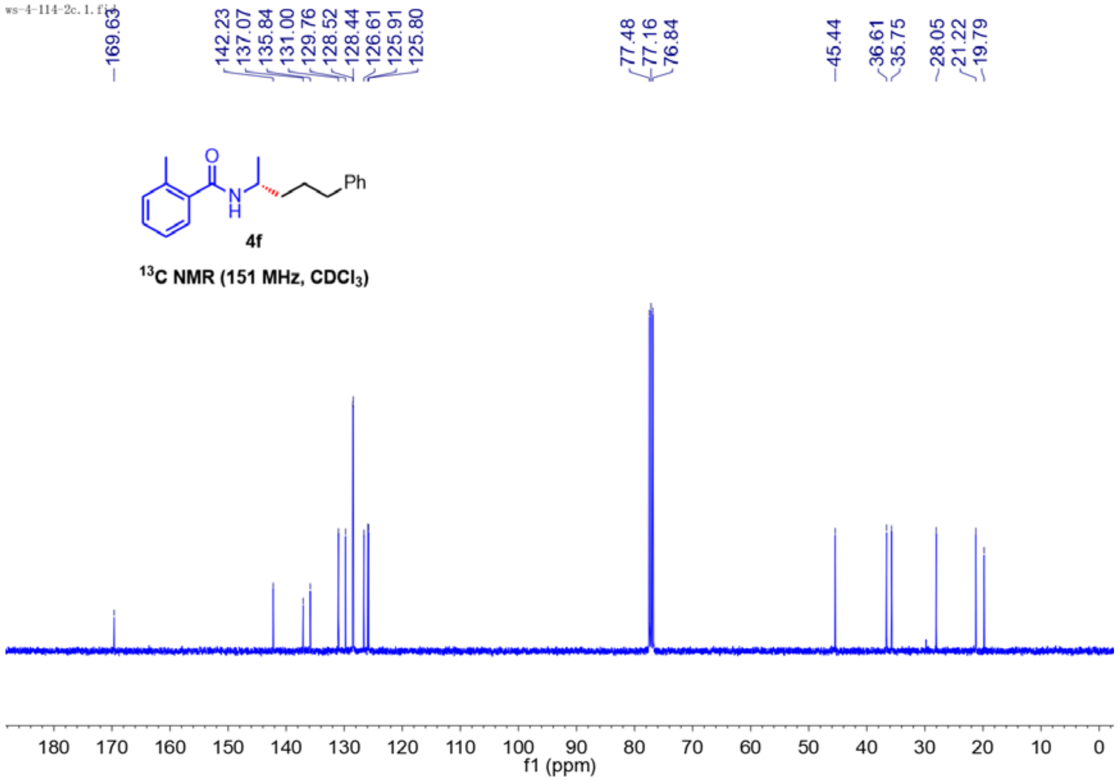

Supplementary Figure 61 ^{13}C NMR (151 MHz, $CDCl_3$) of **4f**

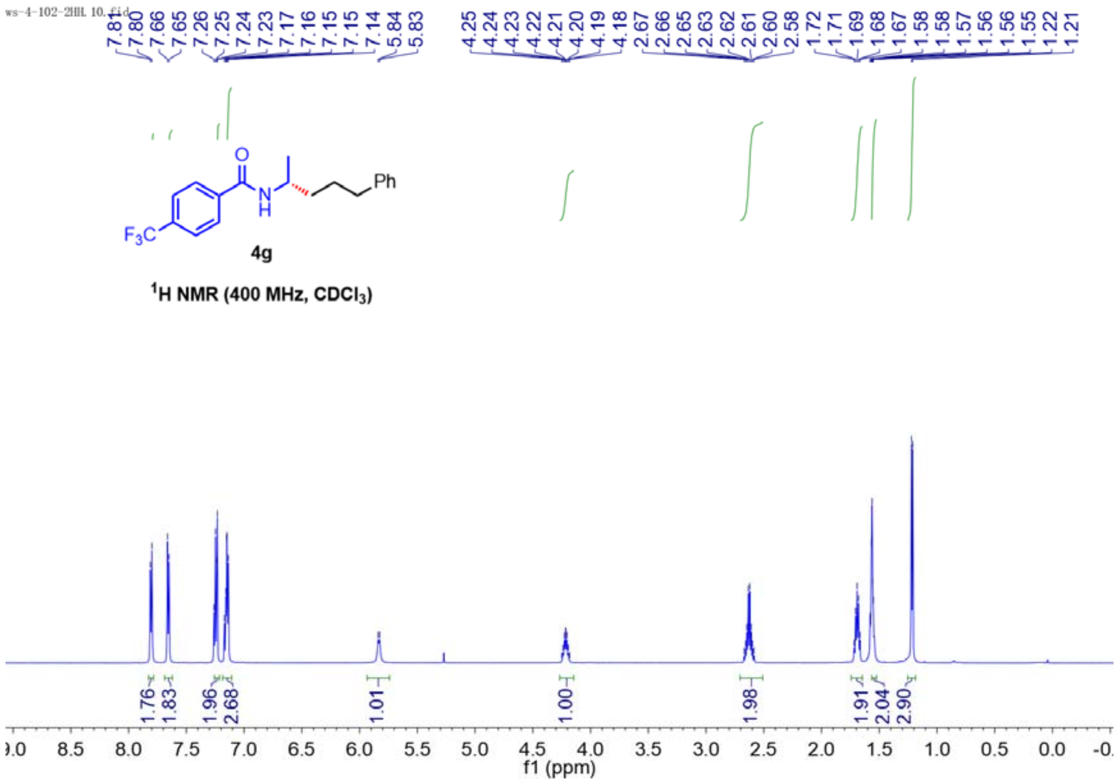

Supplementary Figure 62 ^{1}H NMR (400 MHz, $CDCl_3$) of **4g**

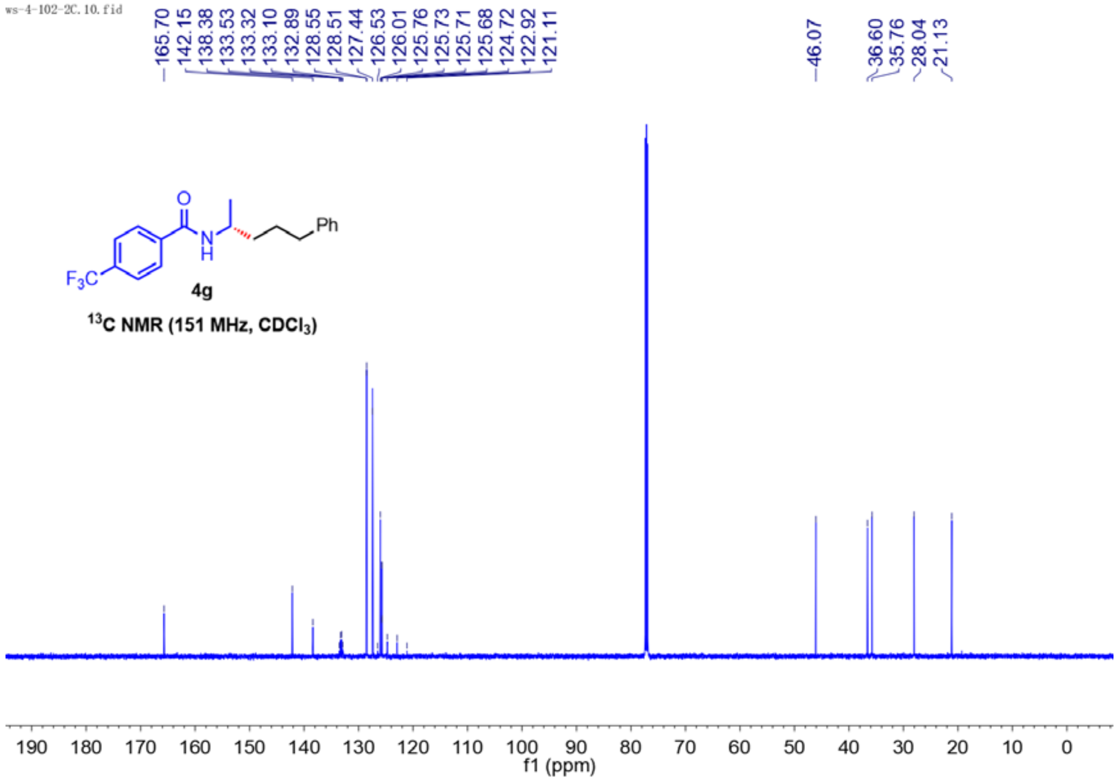

Supplementary Figure 63 ^{13}C NMR (151 MHz, $CDCl_3$) of **4g**

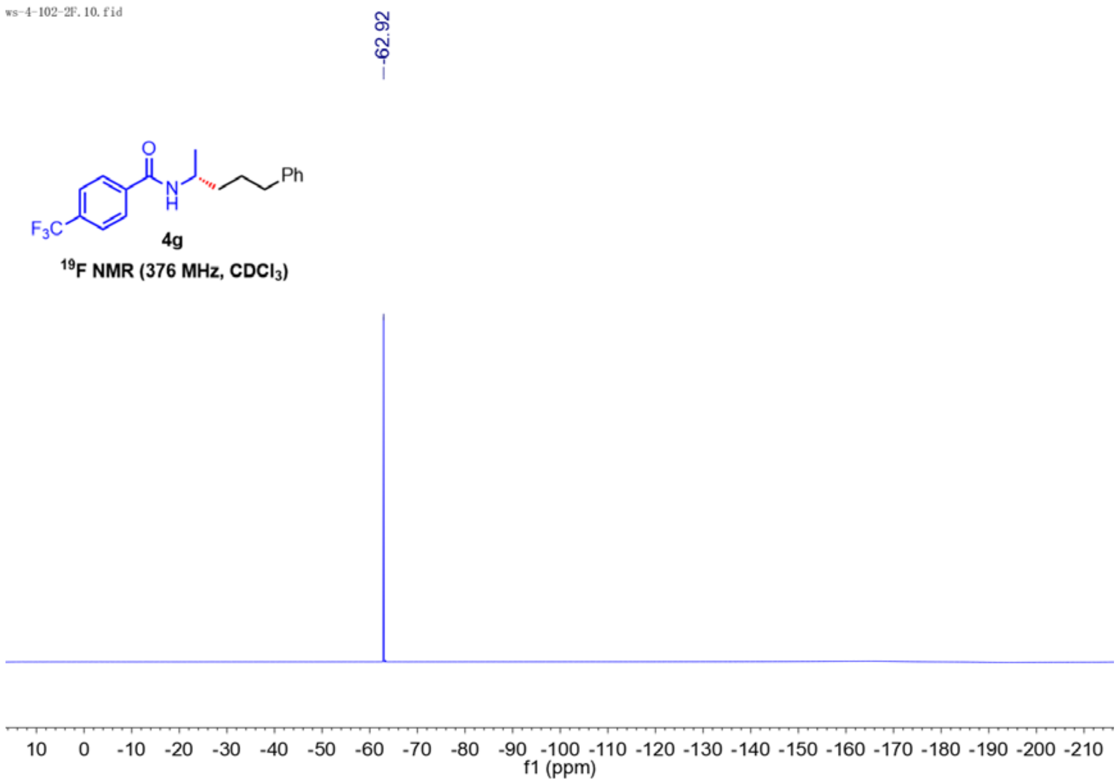

Supplementary Figure 64 ^{19}F NMR (376 MHz, $CDCl_3$) of **4g**

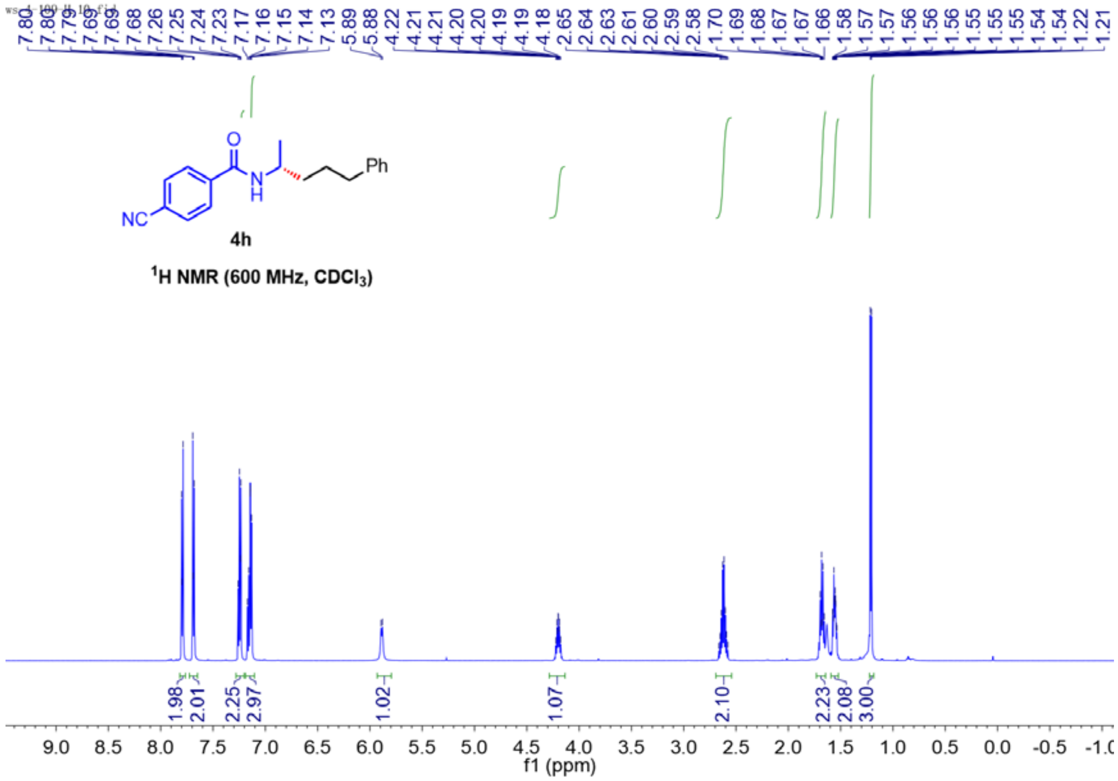

Supplementary Figure 65 ^{1}H NMR (600 MHz, $CDCl_3$) of **4h**

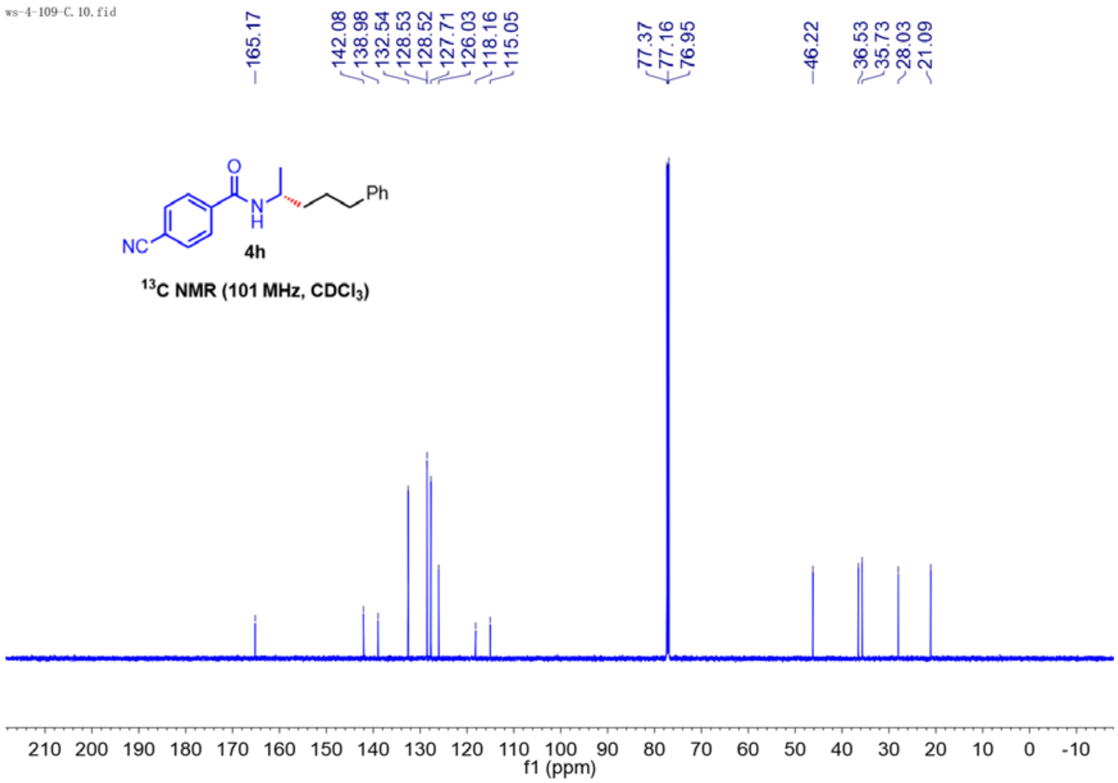

Supplementary Figure 66 ^{13}C NMR (101 MHz, $CDCl_3$) of **4h**

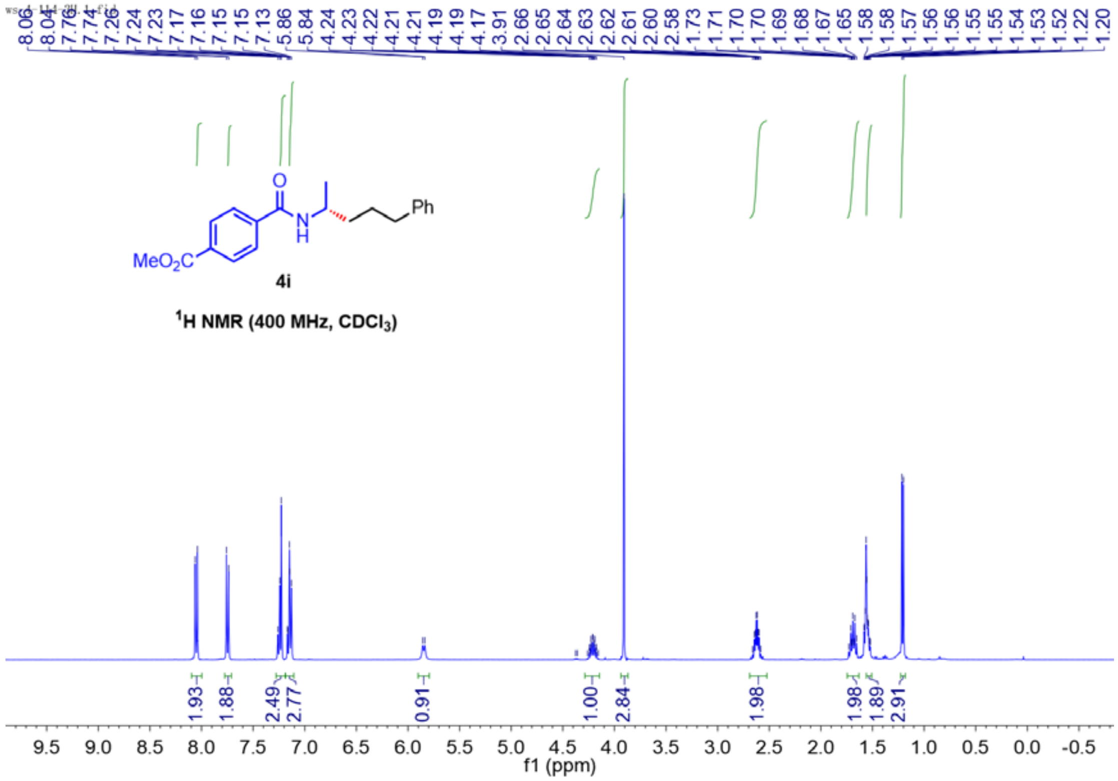

Supplementary Figure 67 ^{1}H NMR (400 MHz, $CDCl_3$) of **4i**

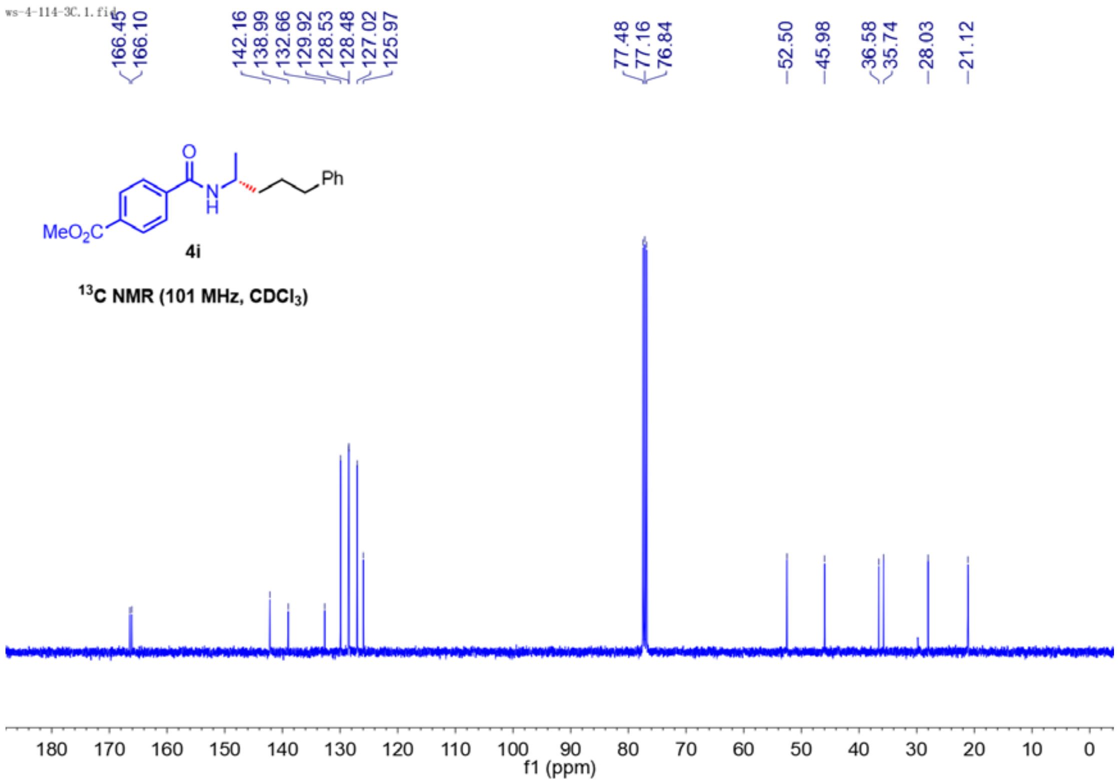

Supplementary Figure 68 ^{13}C NMR (101 MHz, $CDCl_3$) of **4i**

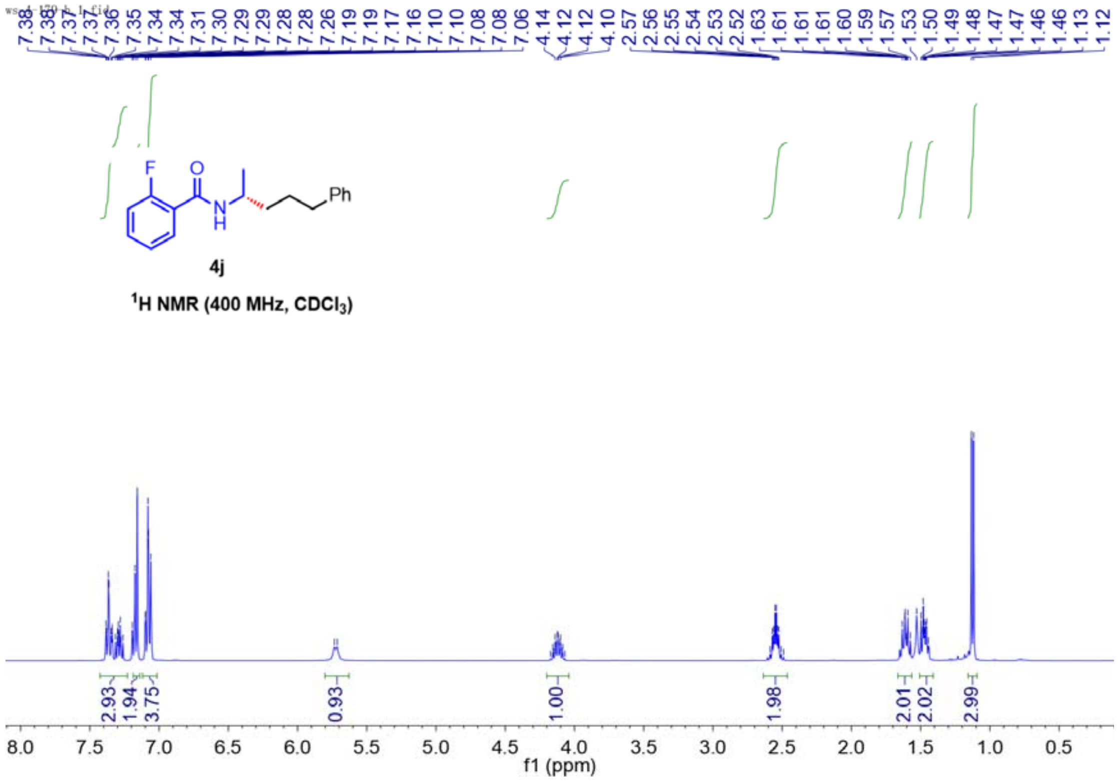

Supplementary Figure 69 ^{1}H NMR (400 MHz, $CDCl_3$) of **4j**

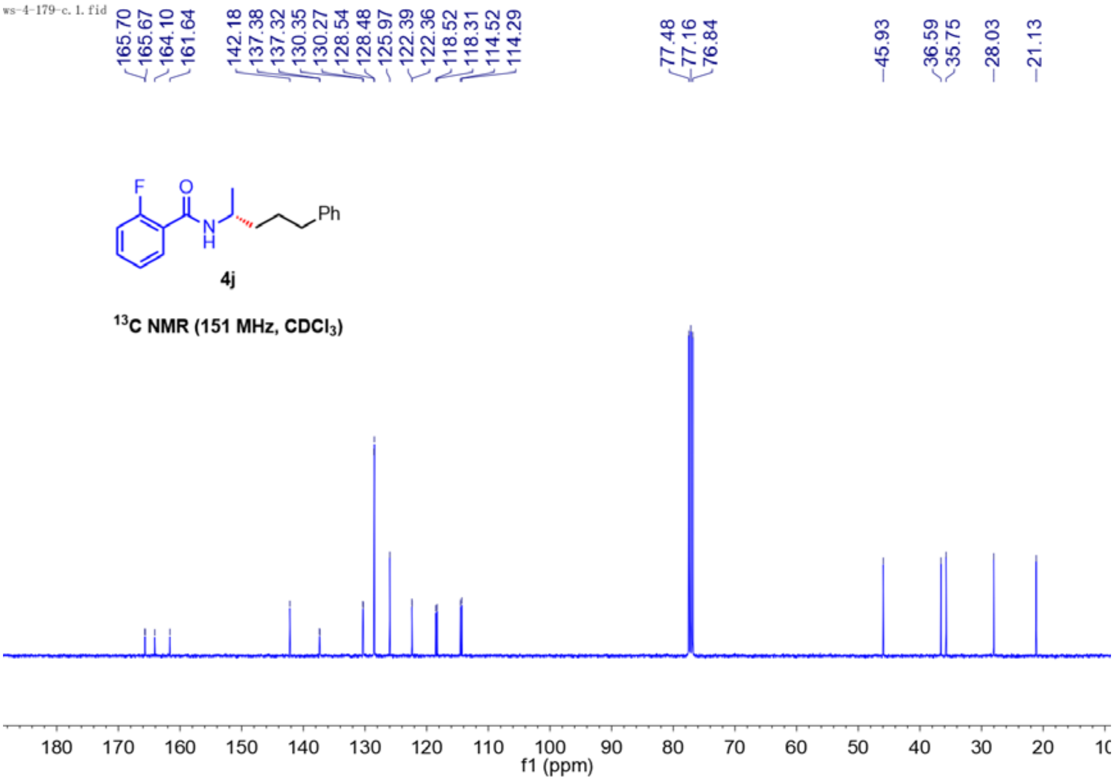

Supplementary Figure 70 ^{13}C NMR (101 MHz, $CDCl_3$) of **4j**

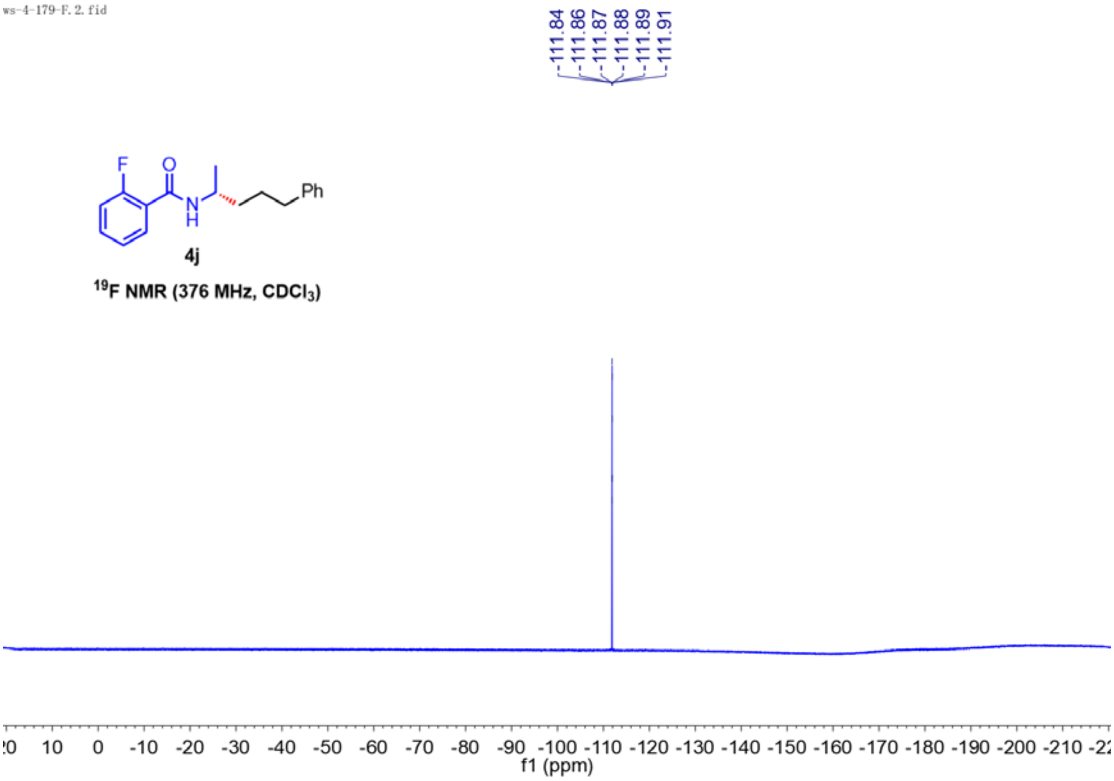

Supplementary Figure 71 ^{19}F NMR (376 MHz, $CDCl_3$) of **4j**

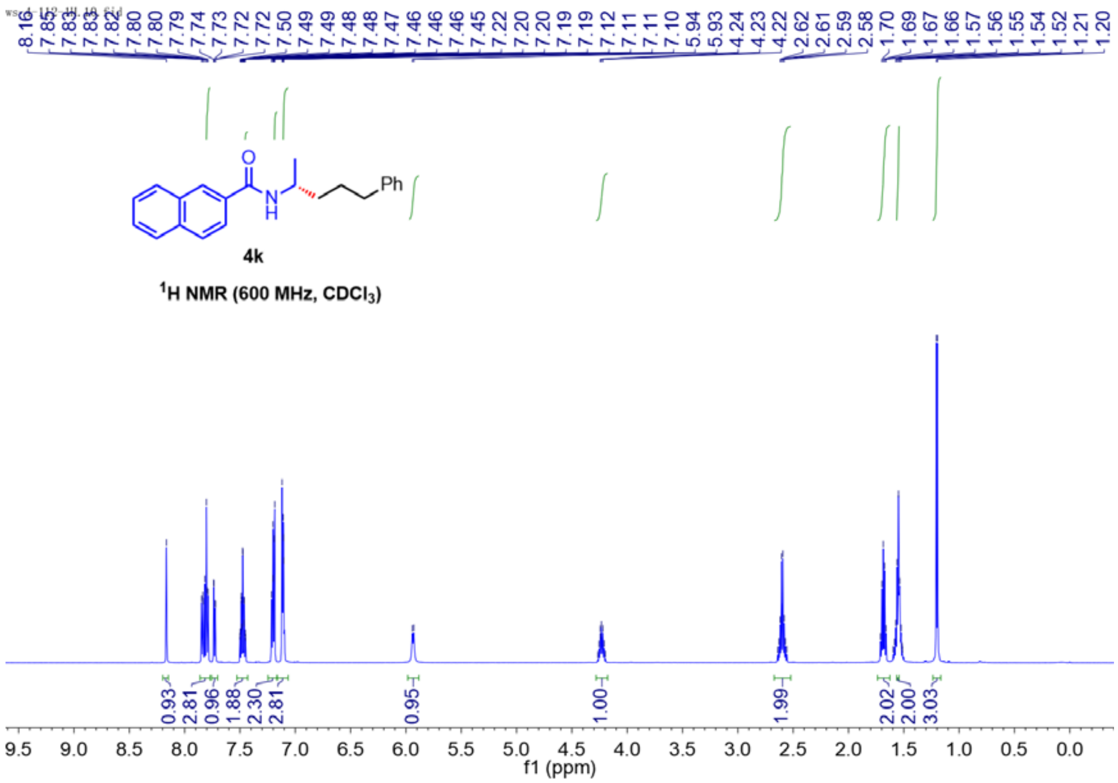

Supplementary Figure 72 ^{1}H NMR (600 MHz, CDCl$_3$) of **4k**

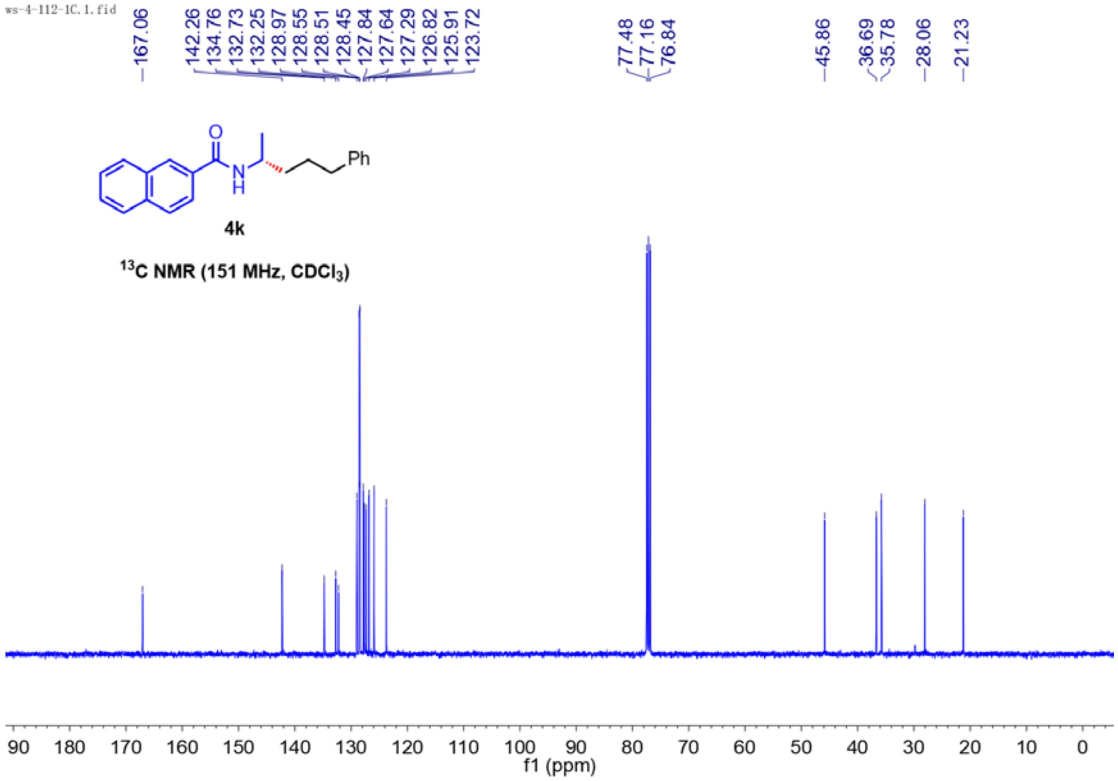

Supplementary Figure 73 ^{13}C NMR (151 MHz, CDCl$_3$) of **4k**

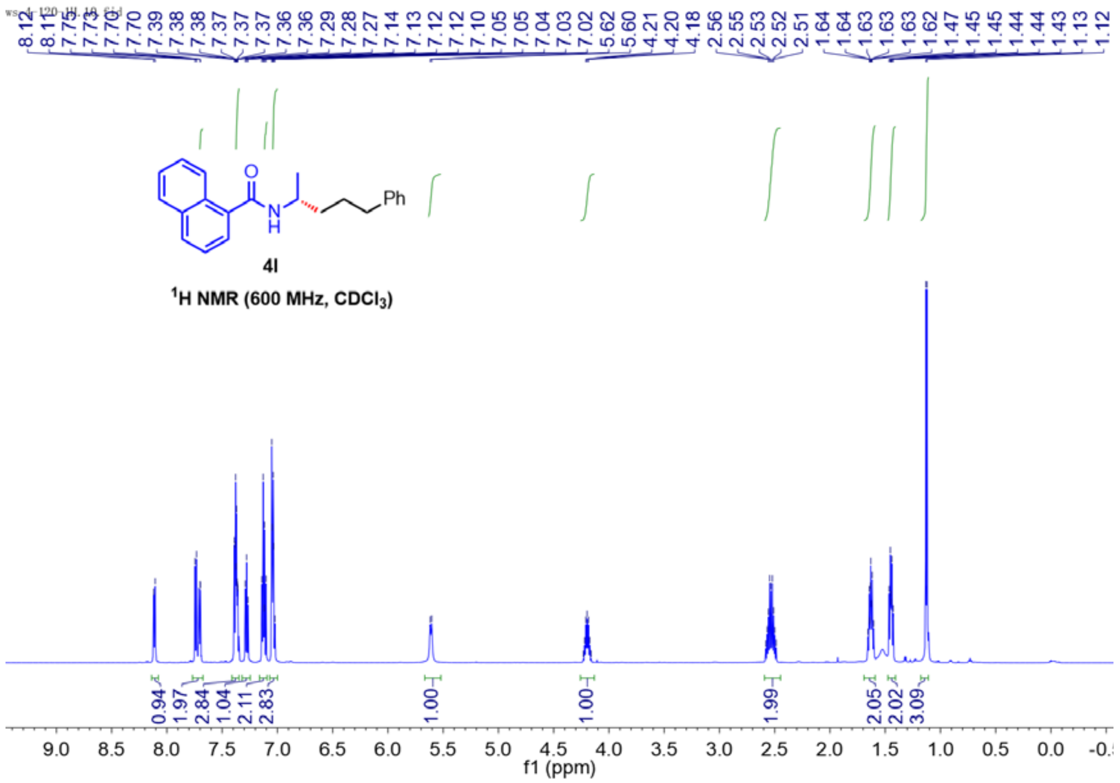

Supplementary Figure 74 ^{1}H NMR (600 MHz, $CDCl_3$) of **4l**

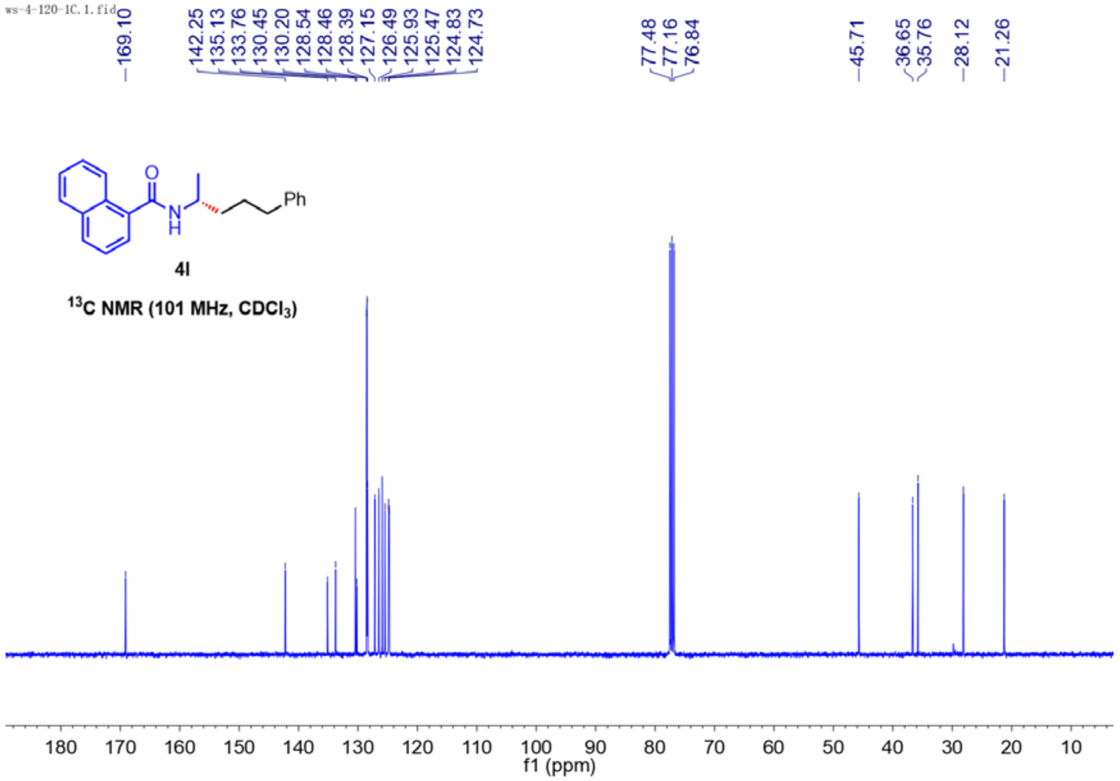

Supplementary Figure 75 ^{13}C NMR (101 MHz, $CDCl_3$) of **4l**

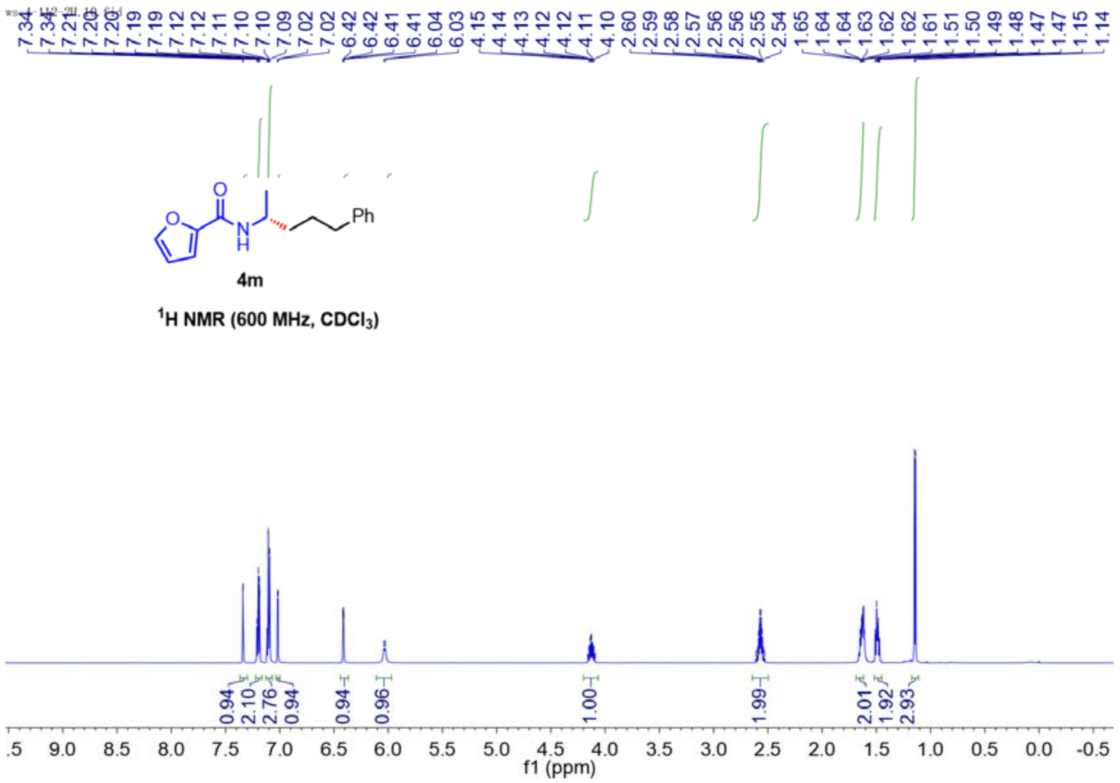

Supplementary Figure 76 ^{1}H NMR (600 MHz, $CDCl_3$) of **4m**

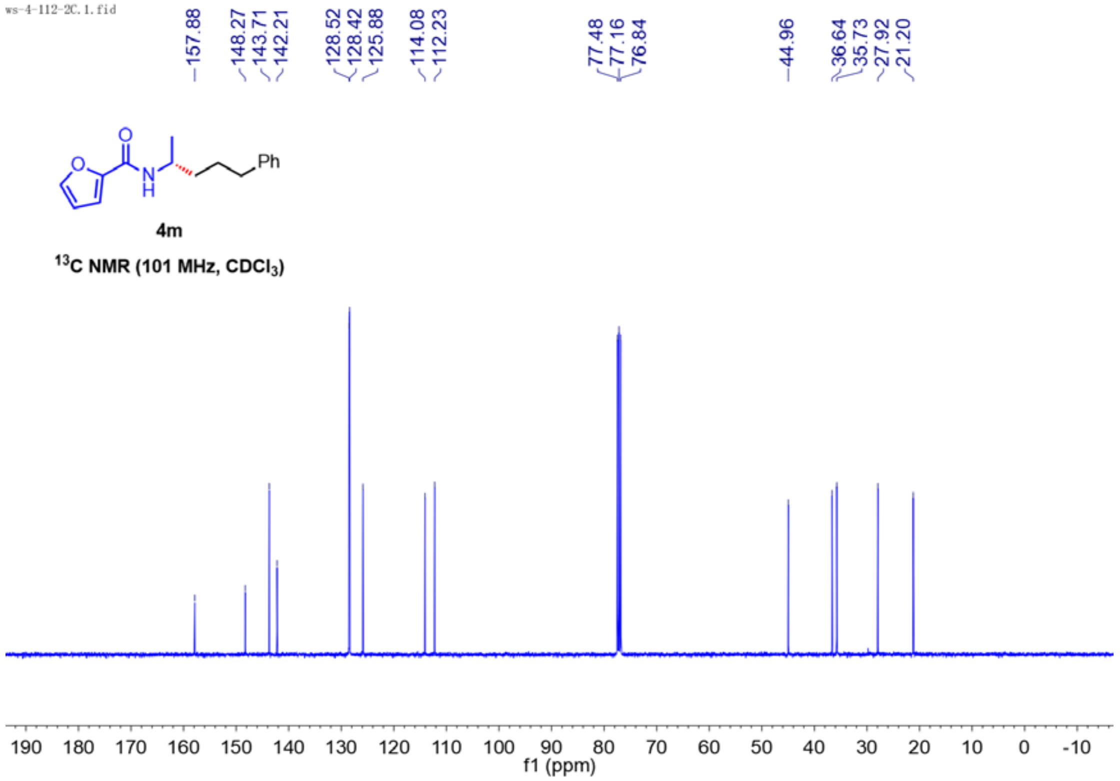

Supplementary Figure 77 ^{13}C NMR (101 MHz, $CDCl_3$) of **4m**

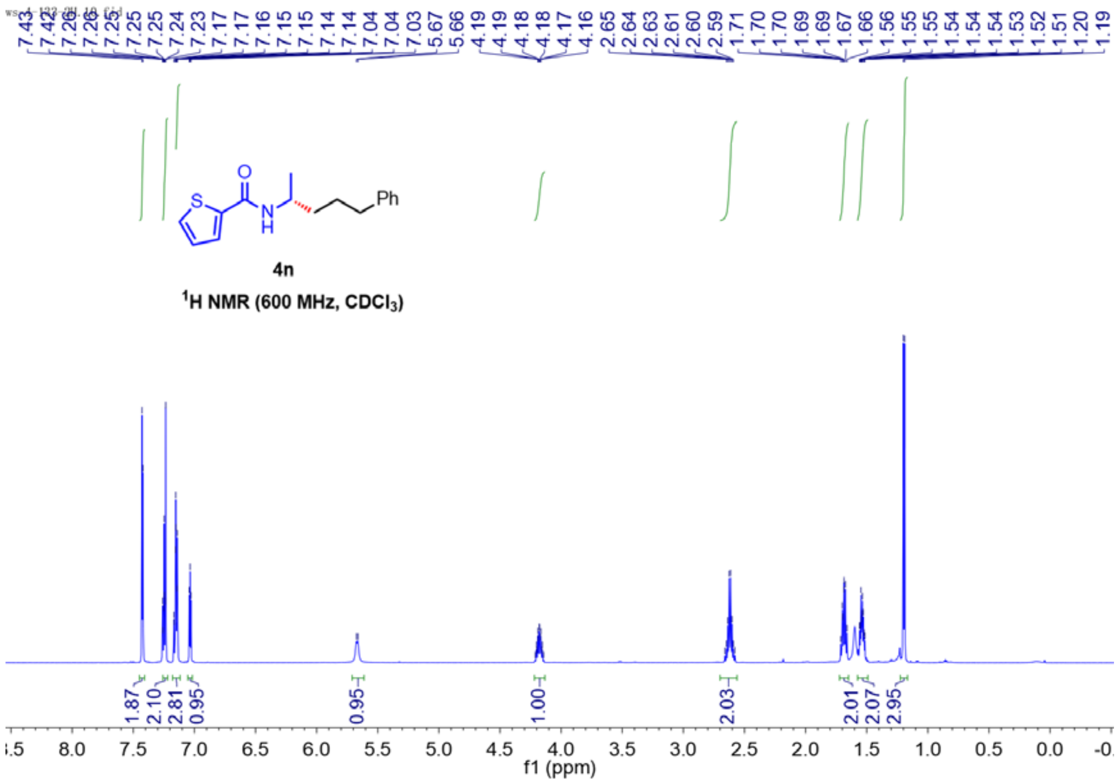

Supplementary Figure 78 ^{1}H NMR (600 MHz, $CDCl_3$) of **4n**

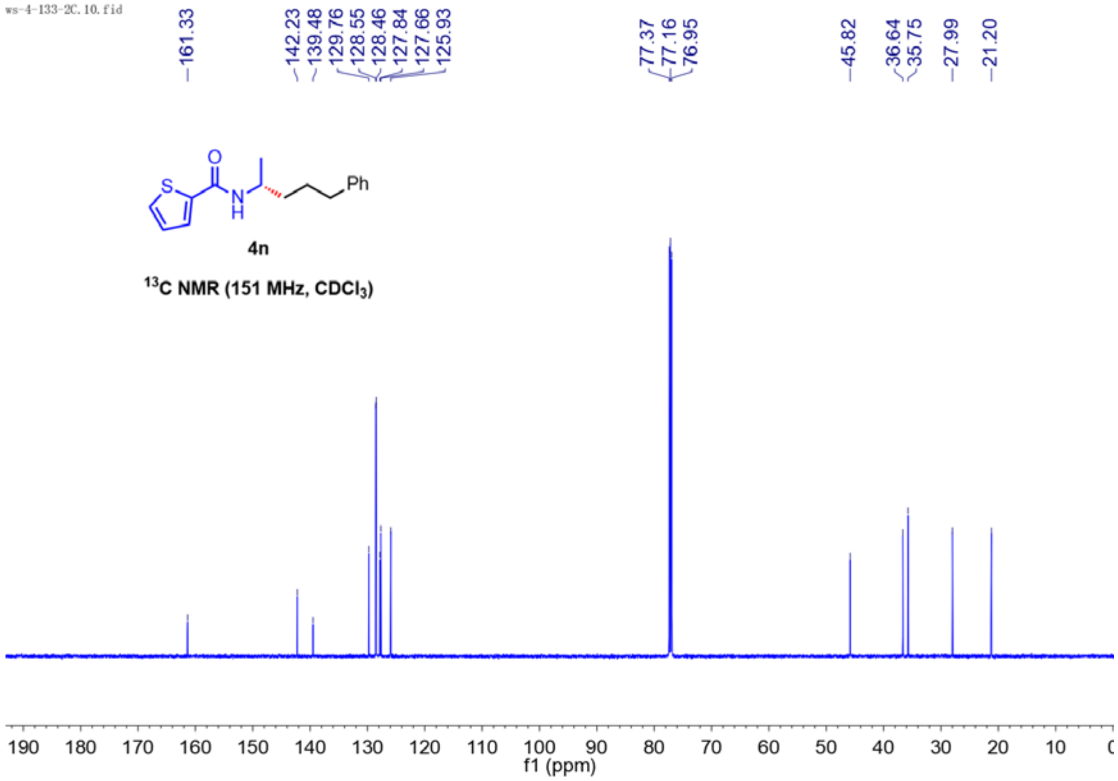

Supplementary Figure 79 ^{13}C NMR (151 MHz, $CDCl_3$) of **4n**

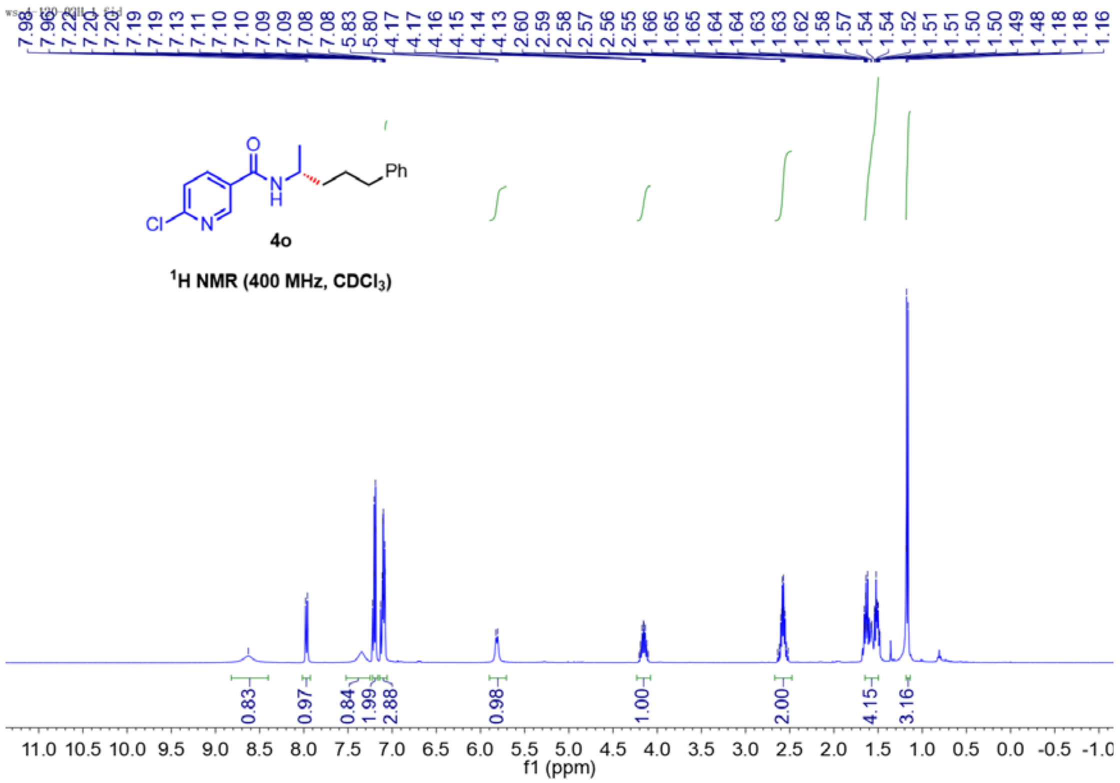

Supplementary Figure 80 ^{1}H NMR (400 MHz, $CDCl_3$) of **4o**

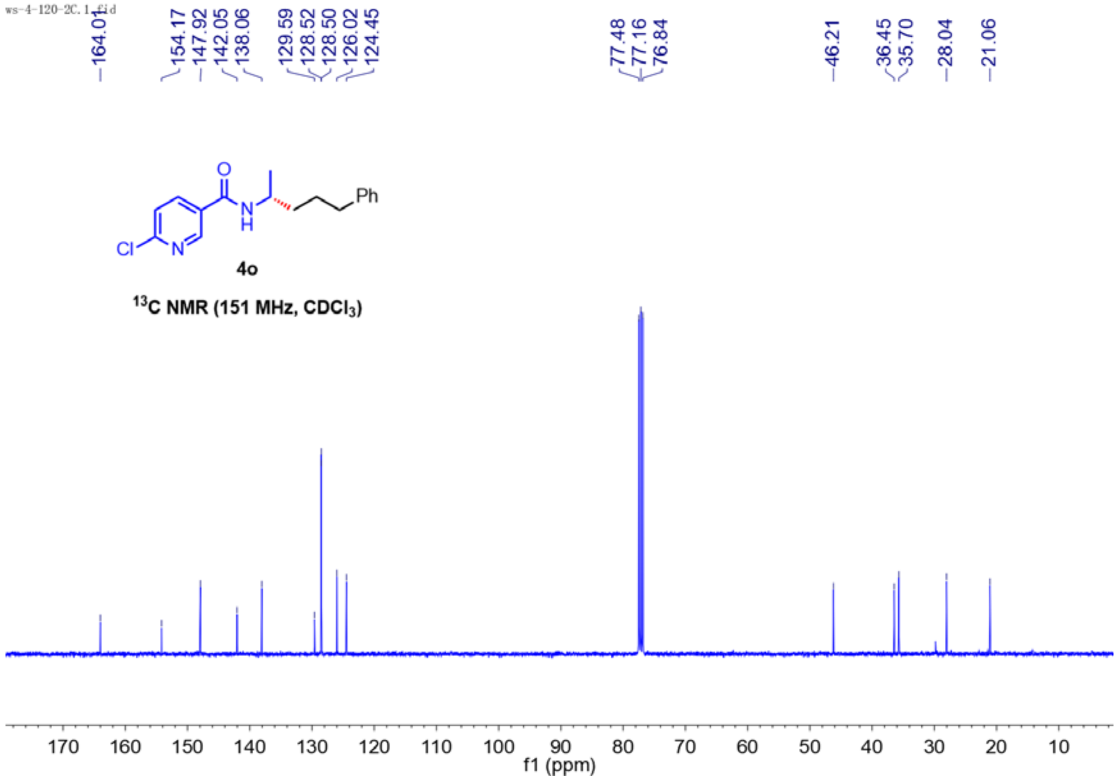

Supplementary Figure 81 ^{13}C NMR (151 MHz, $CDCl_3$) of **4o**

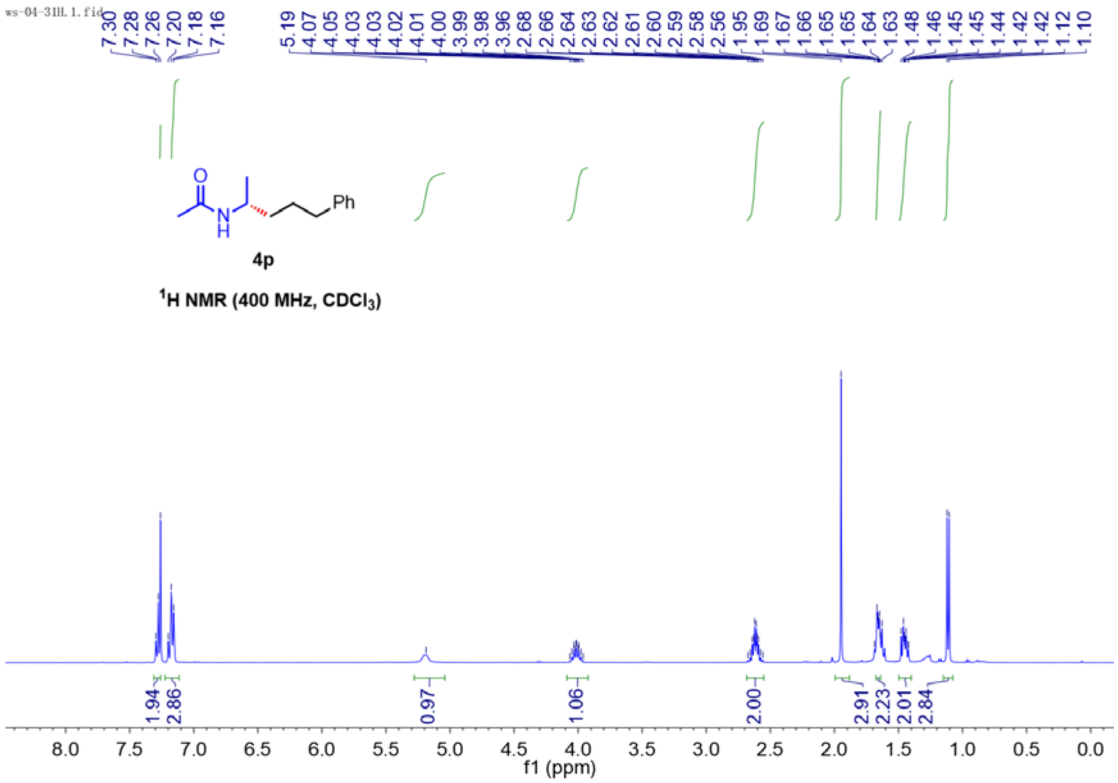

Supplementary Figure 82 ^{1}H NMR (400 MHz, $CDCl_3$) of **4p**

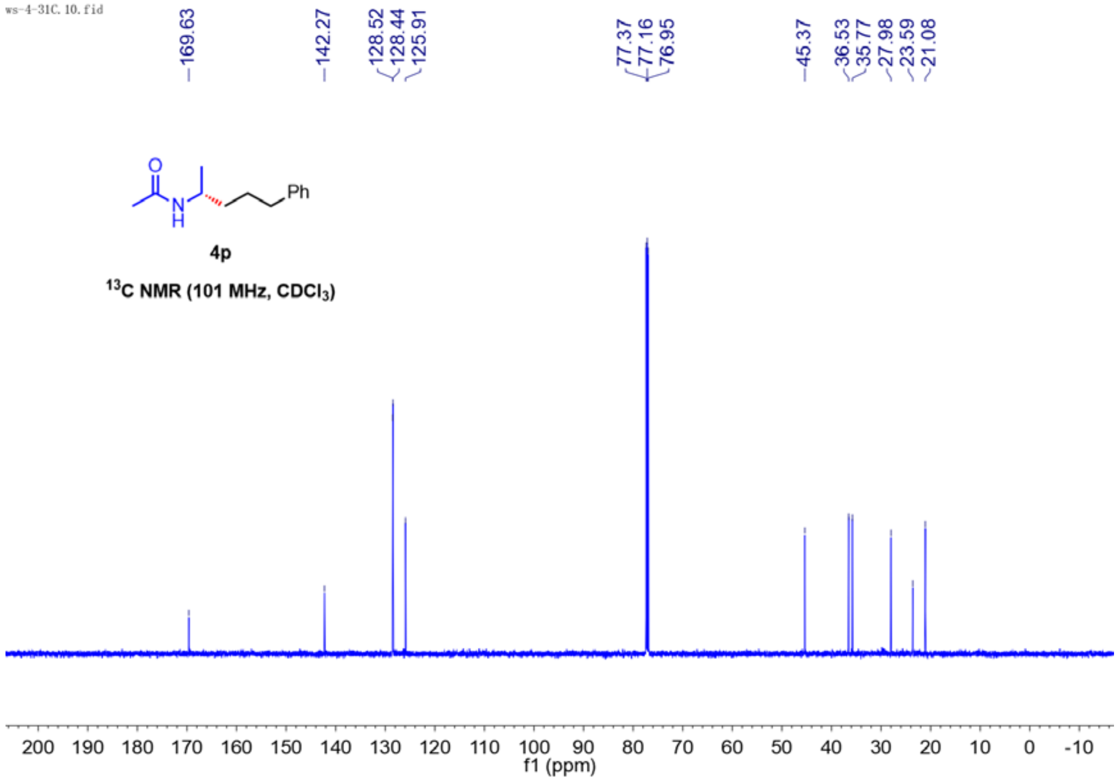

Supplementary Figure 83 ^{13}C NMR (101 MHz, $CDCl_3$) of **4p**

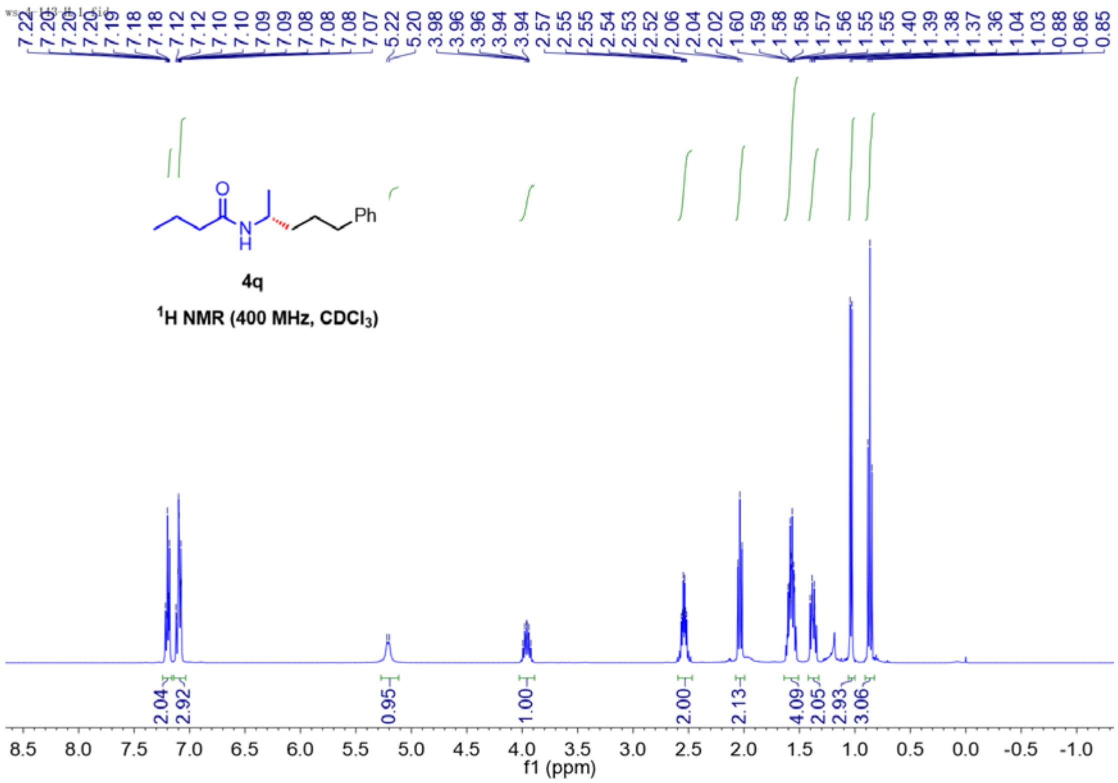

Supplementary Figure 84 ^{1}H NMR (400 MHz, $CDCl_3$) of **4q**

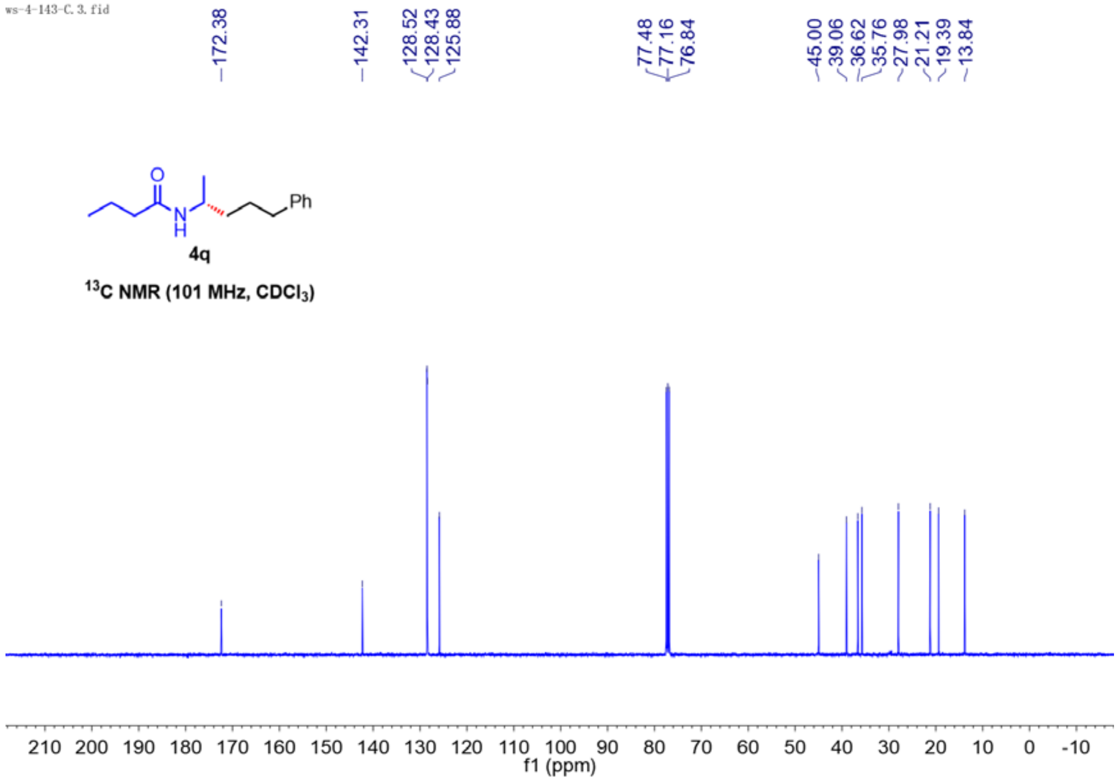

Supplementary Figure 85 ^{13}C NMR (101 MHz, $CDCl_3$) of **4q**

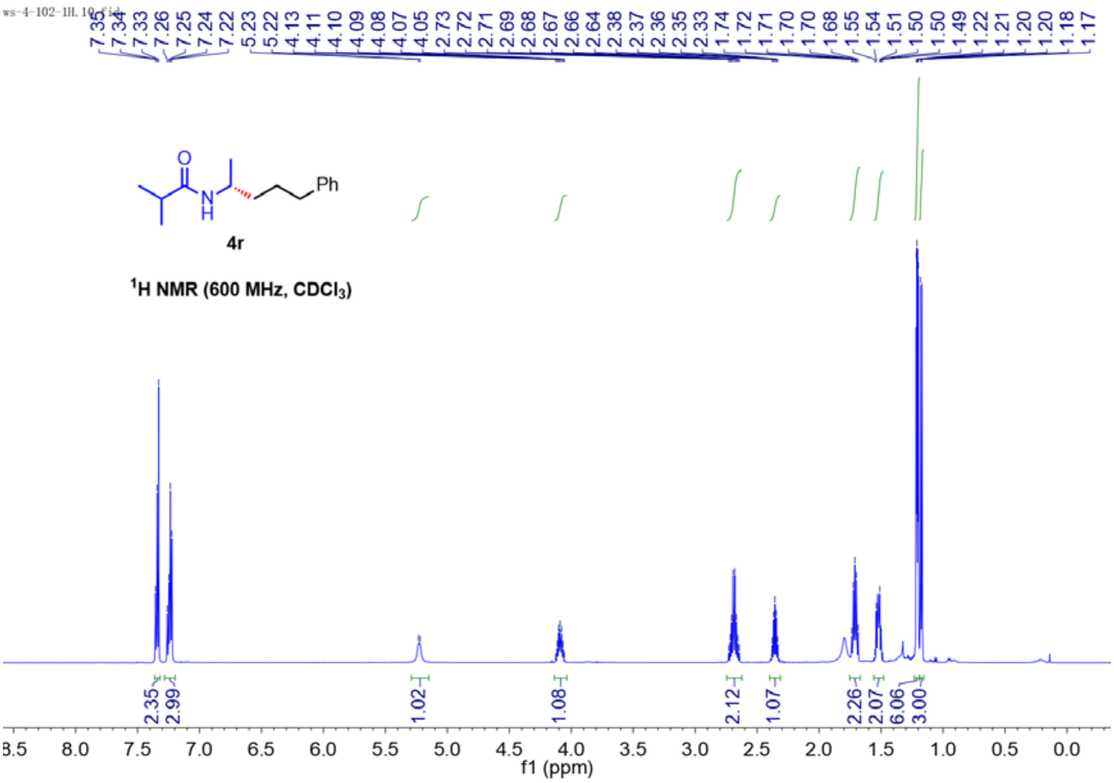

Supplementary Figure 86 ^{1}H NMR (600 MHz, CDCl$_3$) of **4r**

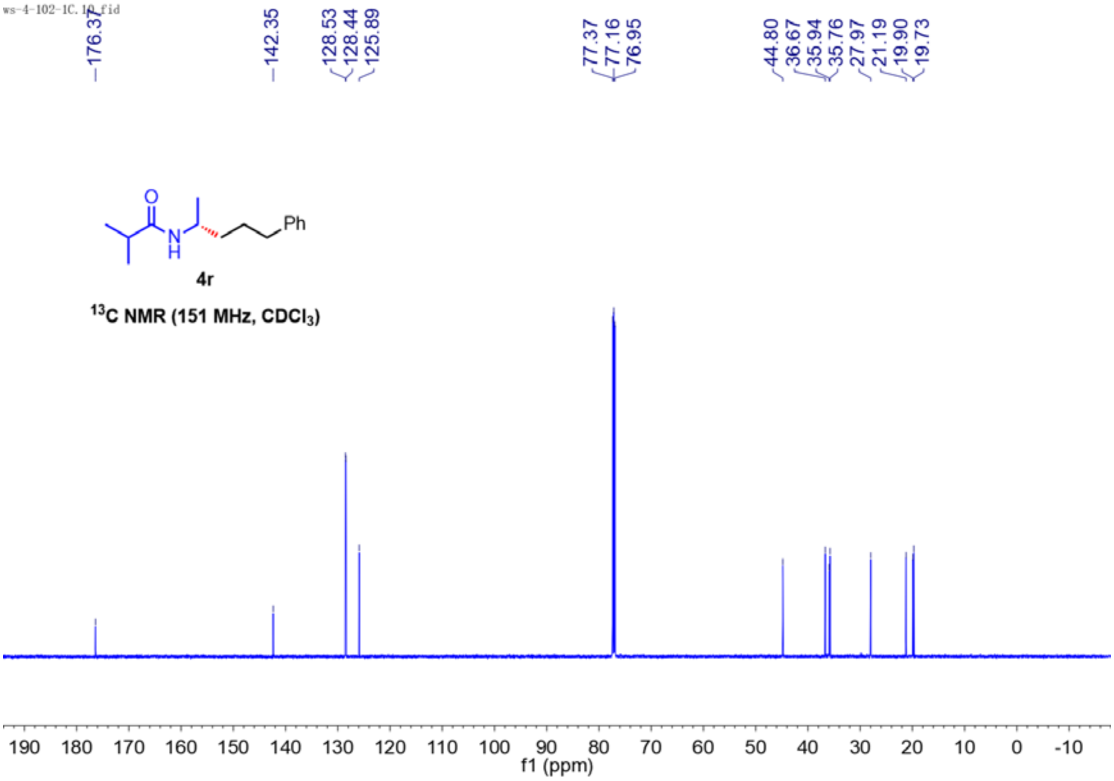

Supplementary Figure 87 ^{13}C NMR (151 MHz, CDCl$_3$) of **4r**

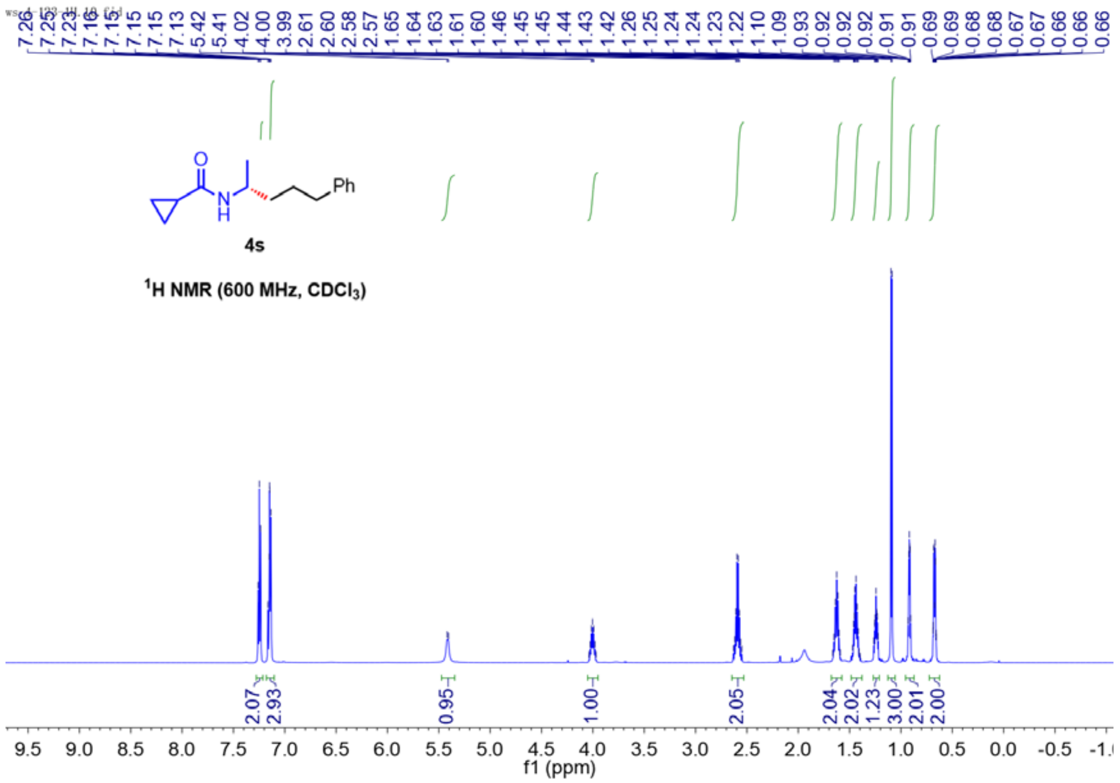

Supplementary Figure 88 ^{1}H NMR (600 MHz, $CDCl_3$) of **4s**

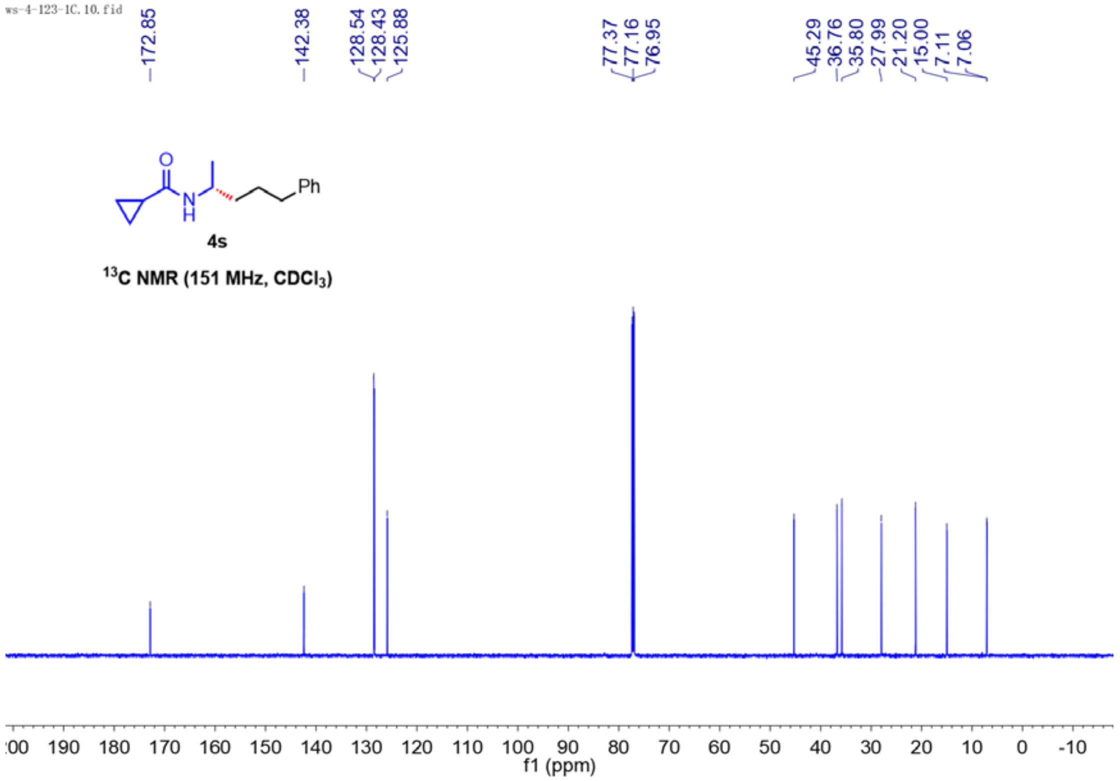

Supplementary Figure 89 ^{13}C NMR (151 MHz, $CDCl_3$) of **4s**

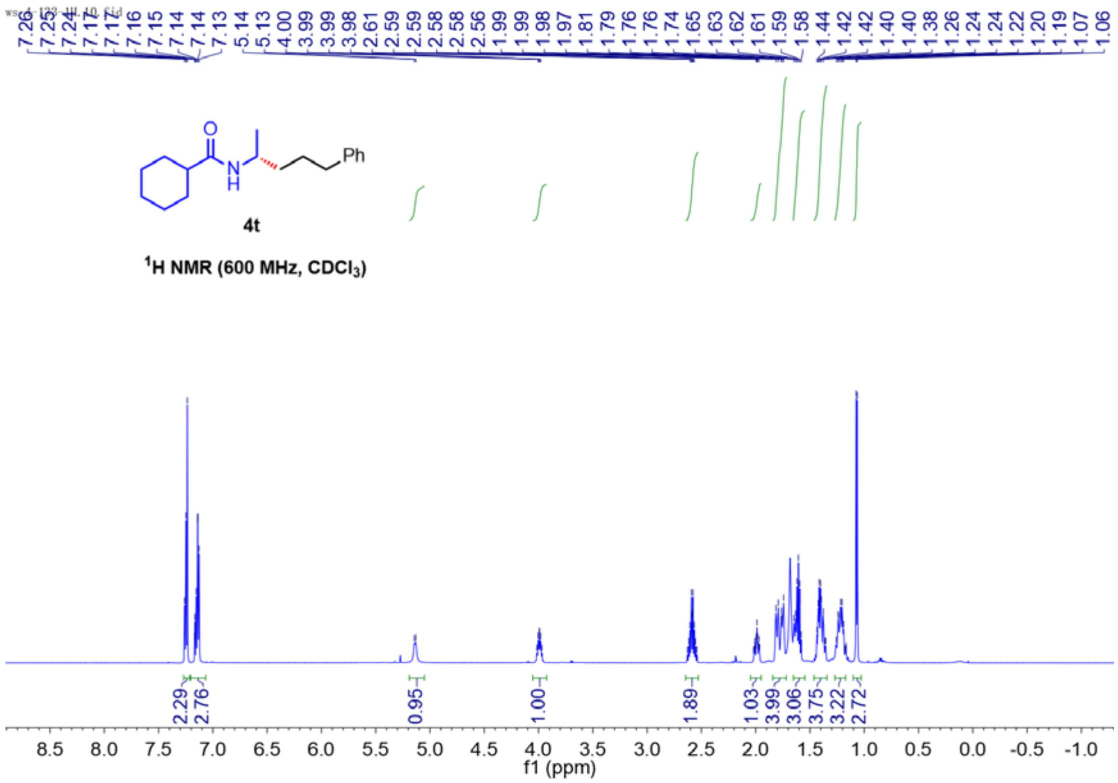

Supplementary Figure 90 ^{1}H NMR (600 MHz, CDCl$_3$) of **4t**

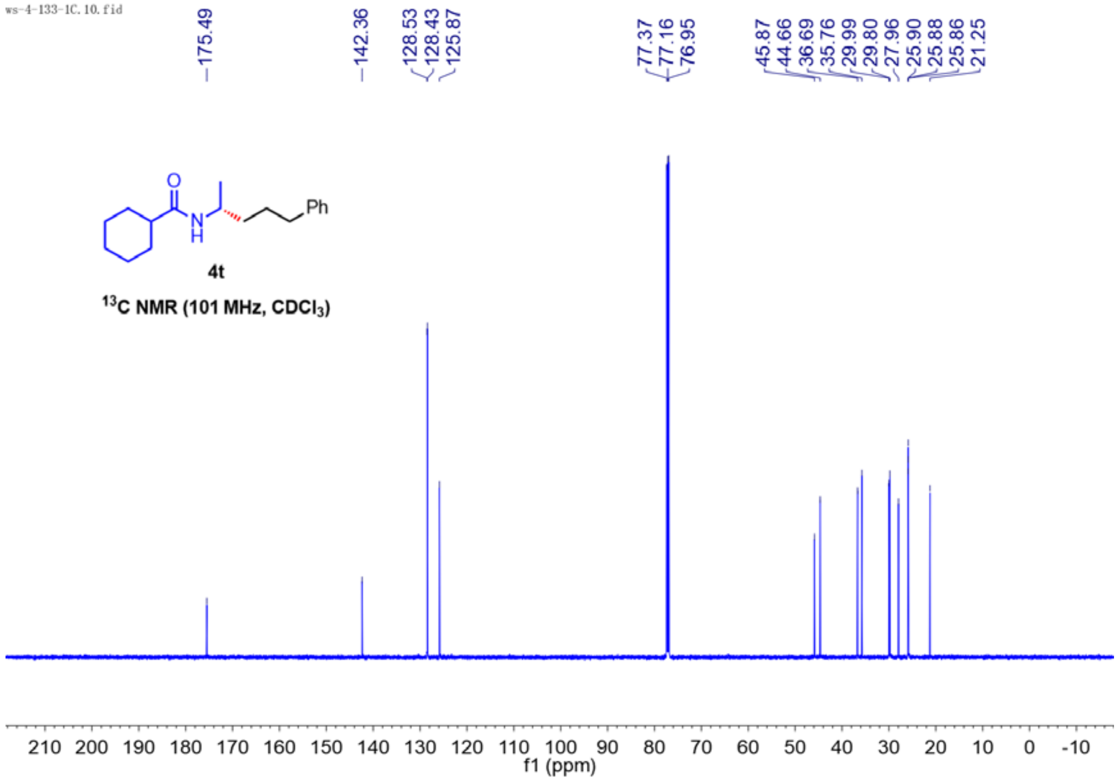

Supplementary Figure 91 ^{13}C NMR (101 MHz, CDCl$_3$) of **4t**

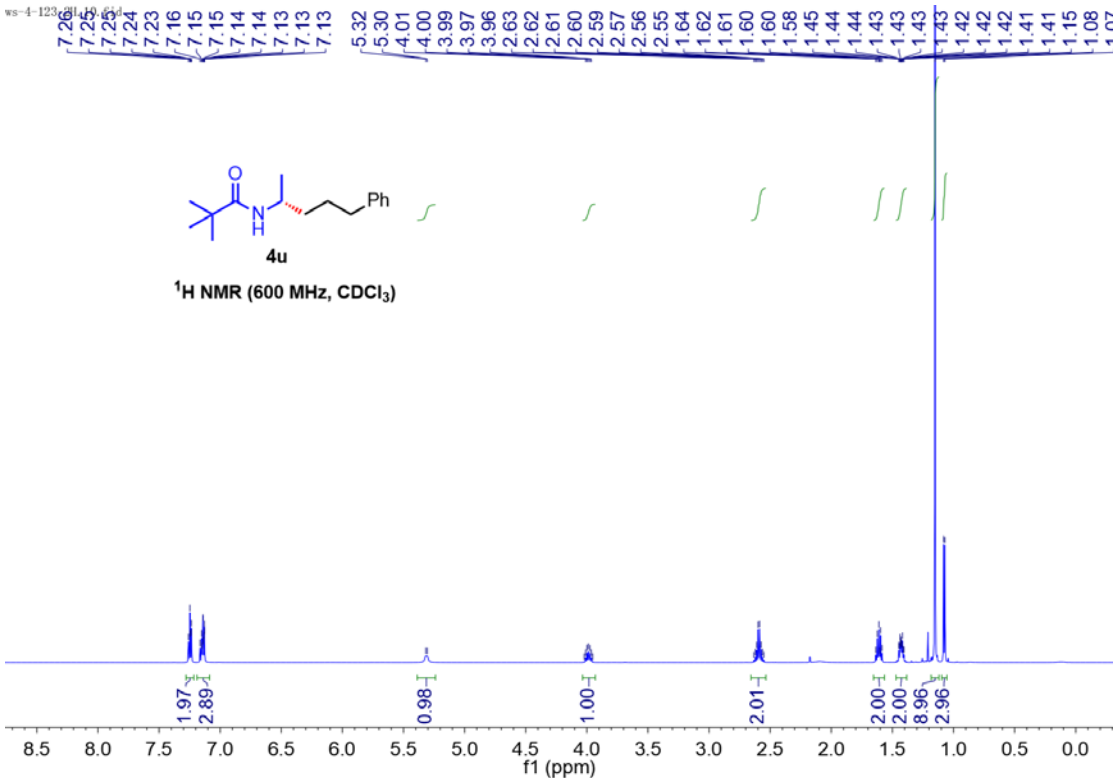

Supplementary Figure 92 ^{1}H NMR (600 MHz, $CDCl_3$) of **4u**

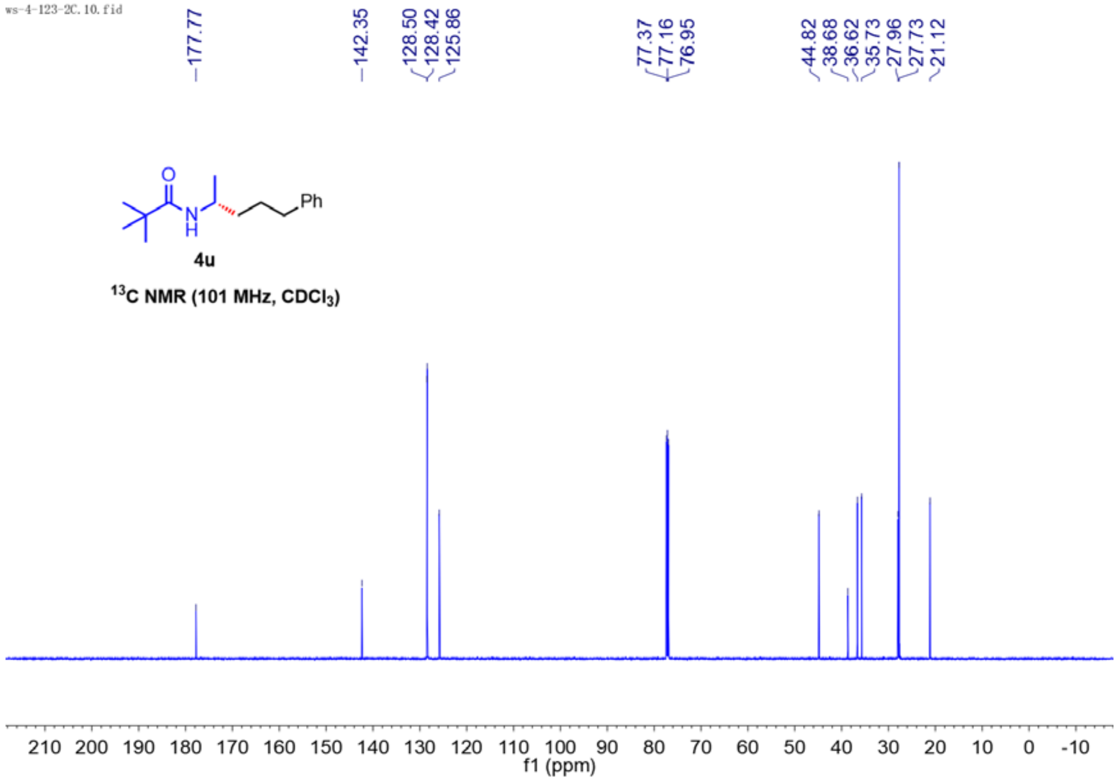

Supplementary Figure 93 ^{13}C NMR (101 MHz, $CDCl_3$) of **4u**

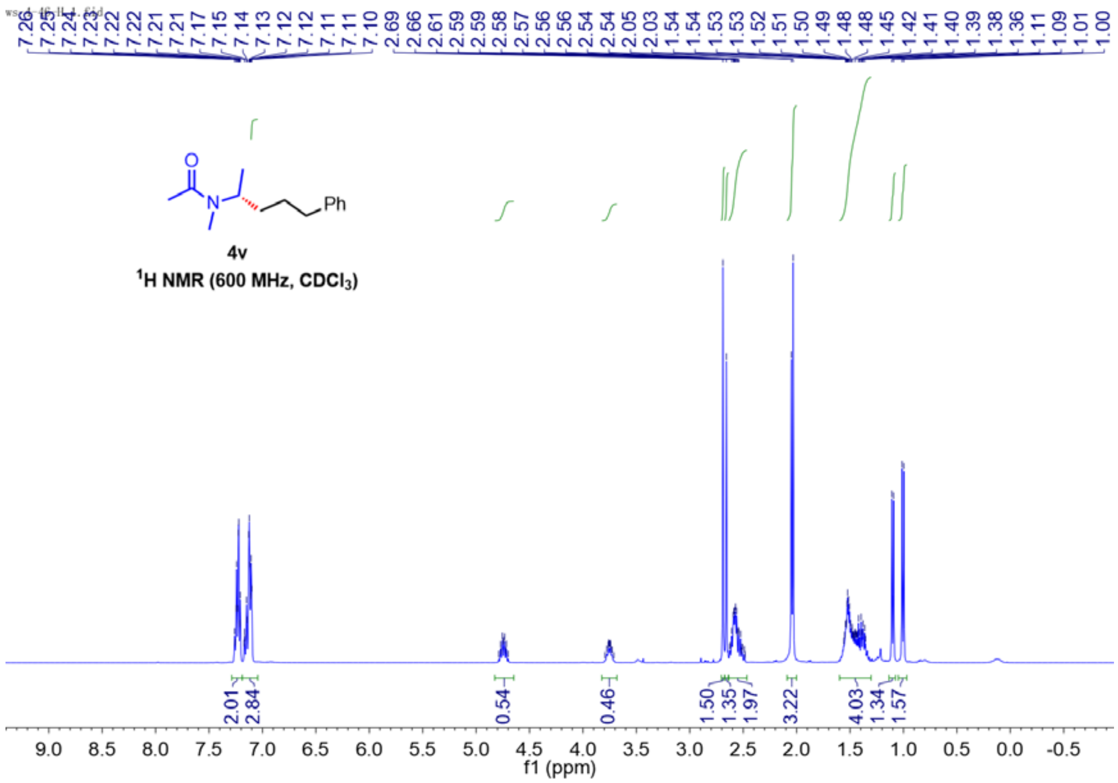

Supplementary Figure 94 ^{1}H NMR (600 MHz, $CDCl_3$) of **4v**

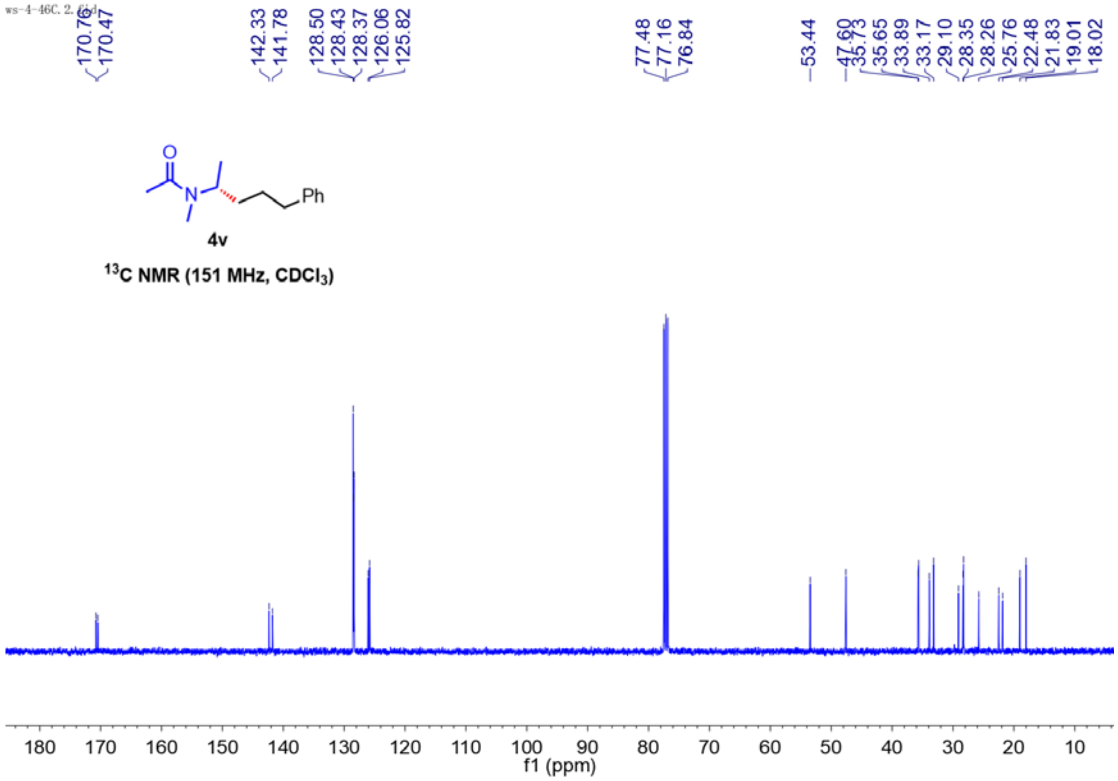

Supplementary Figure 95 ^{13}C NMR (151 MHz, $CDCl_3$) of **4v**

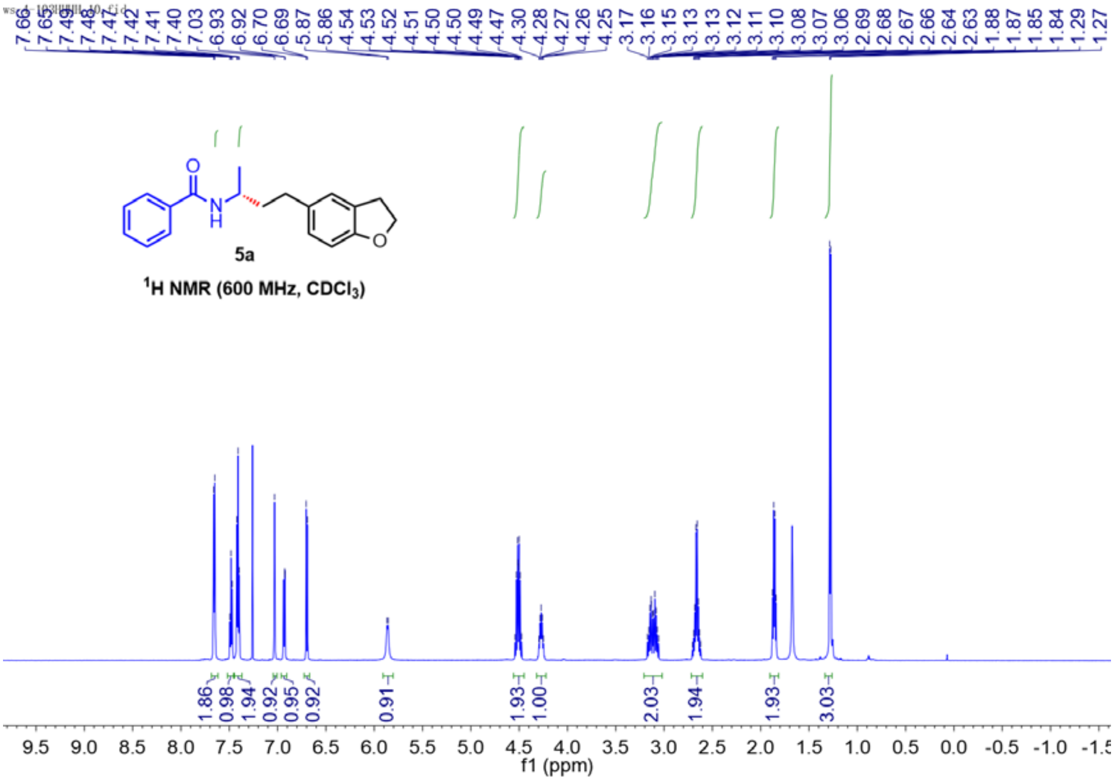

Supplementary Figure 96 ^{1}H NMR (600 MHz, $CDCl_3$) of **5a**

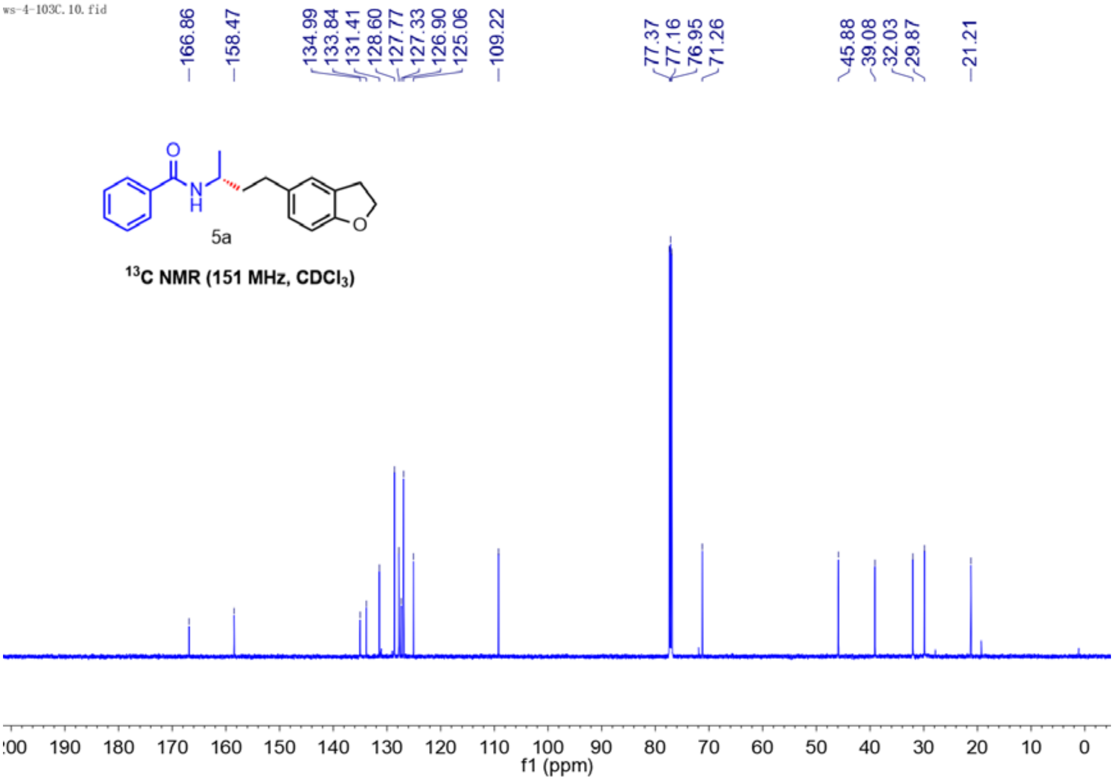

Supplementary Figure 97 ^{13}C NMR (151 MHz, $CDCl_3$) of **5a**

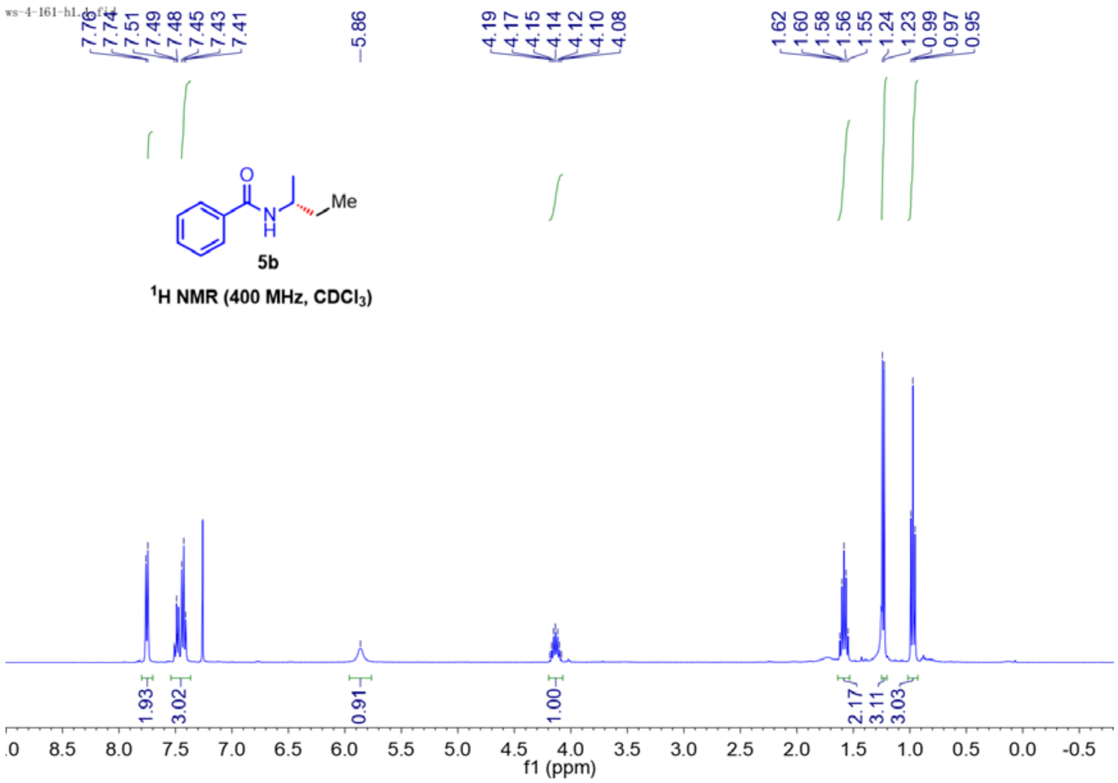

Supplementary Figure 98 ^{1}H NMR (400 MHz, $CDCl_3$) of **5b**

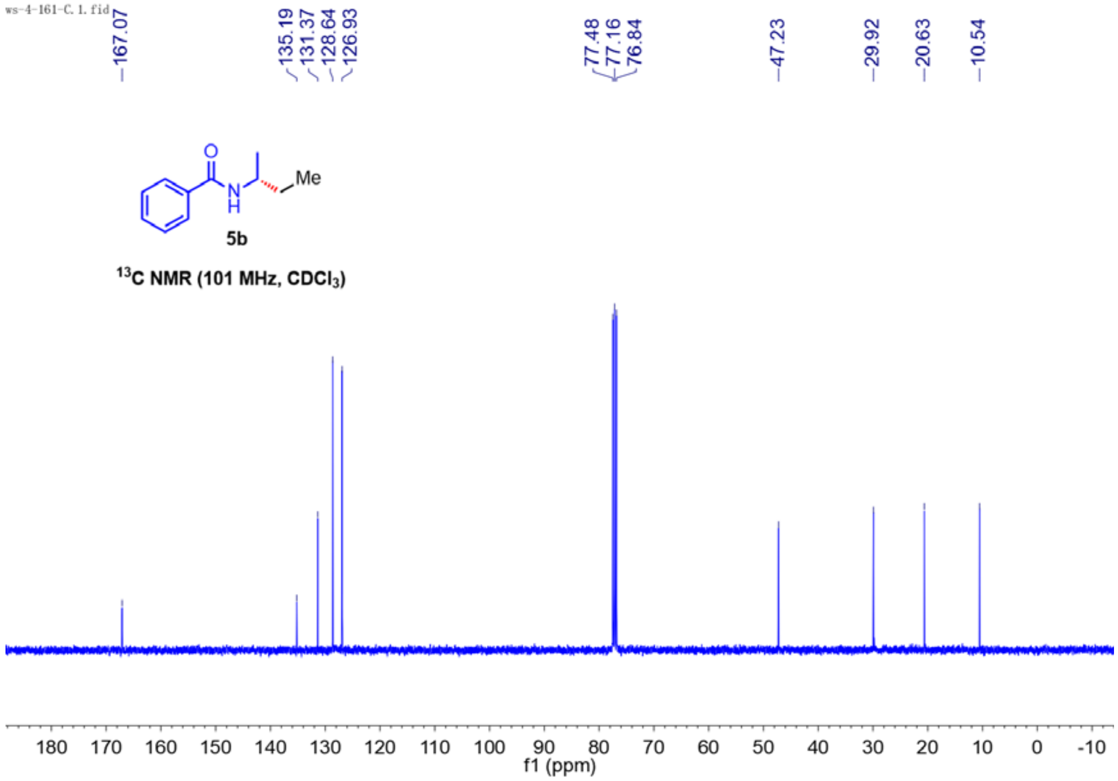

Supplementary Figure 99 ^{13}C NMR (101 MHz, $CDCl_3$) of **5b**

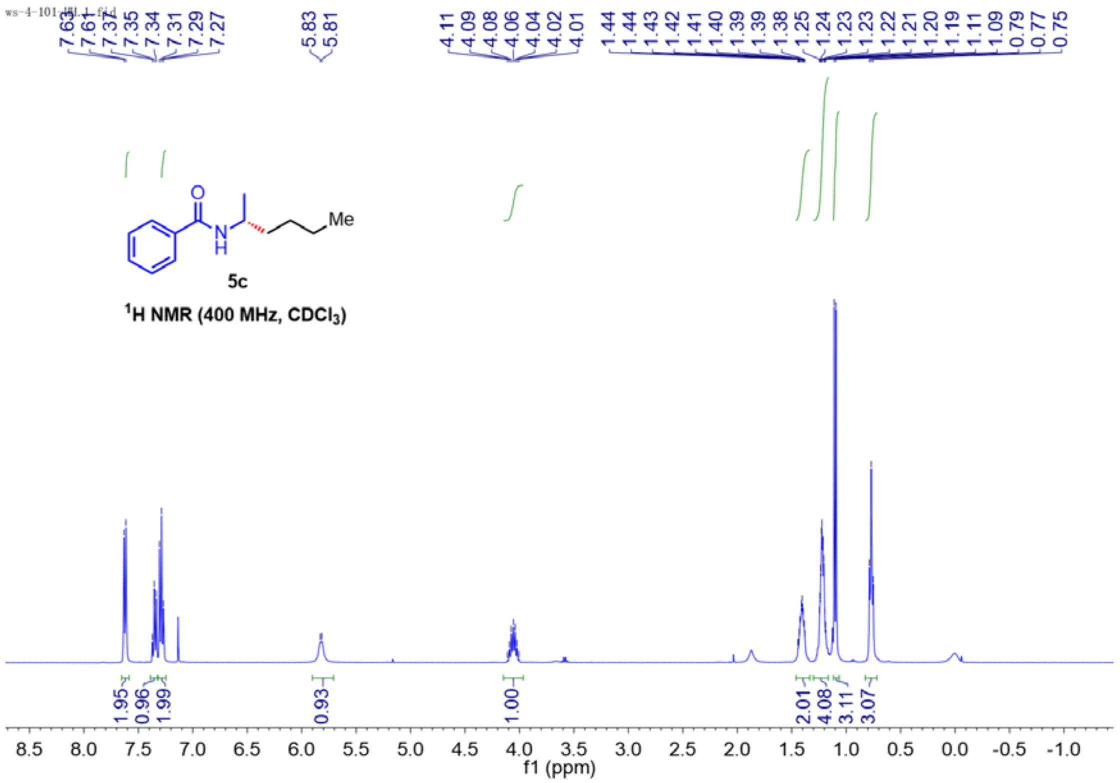

Supplementary Figure 100 ^{1}H NMR (400 MHz, $CDCl_3$) of **5c**

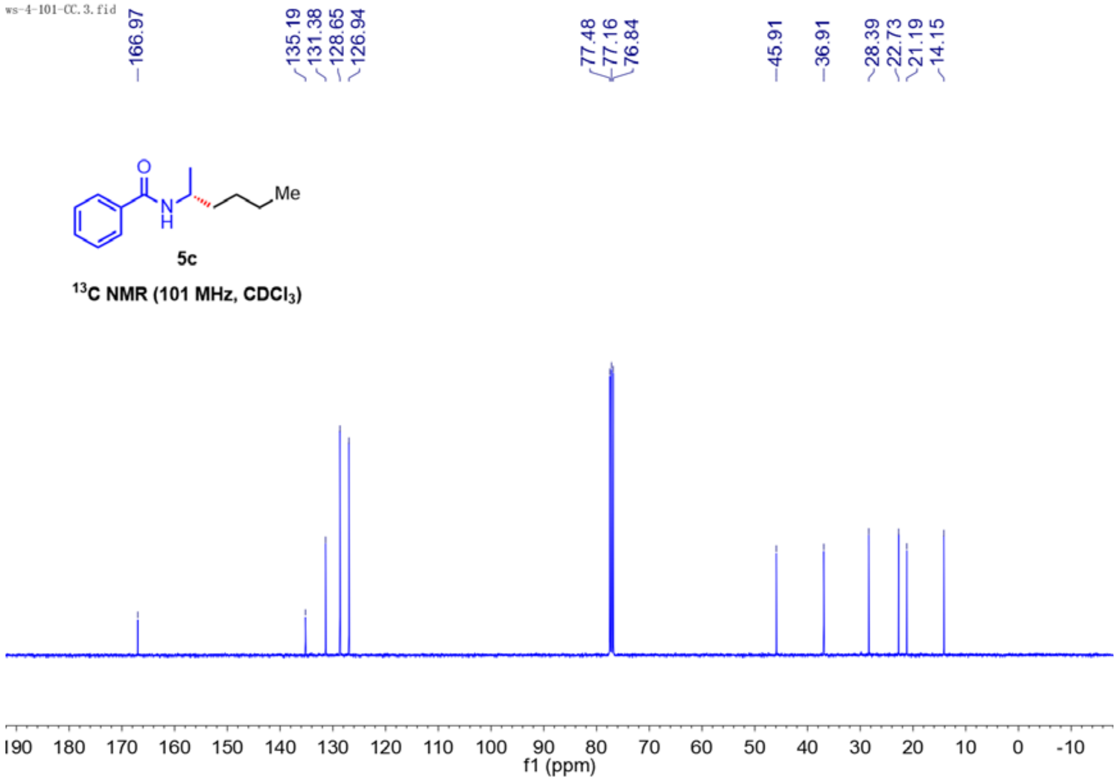

Supplementary Figure 101 ^{13}C NMR (101 MHz, $CDCl_3$) of **5c**

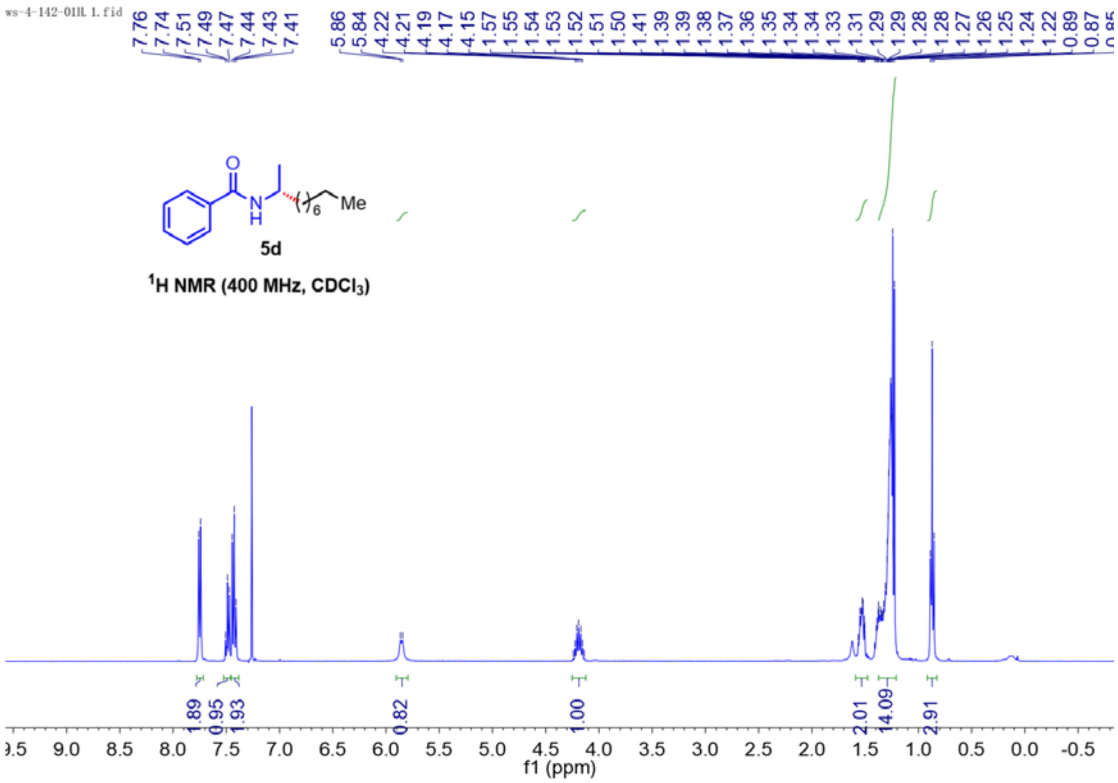

Supplementary Figure 102 ^{1}H NMR (400 MHz, $CDCl_3$) of **5d**

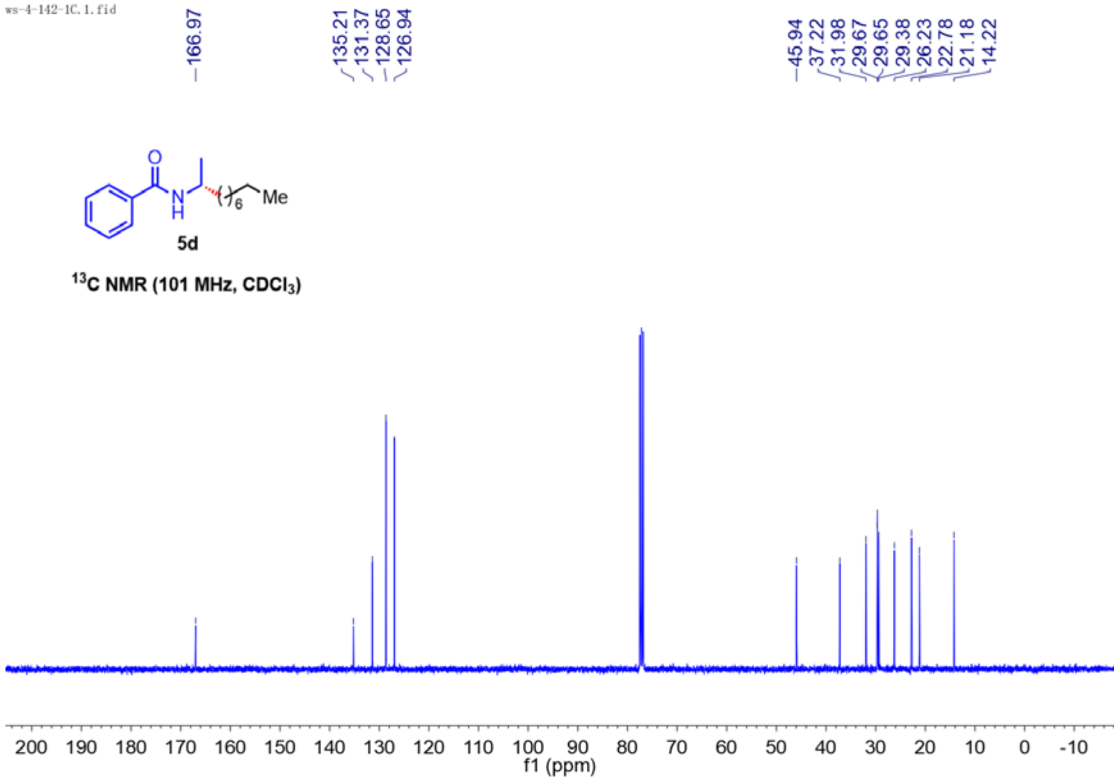

Supplementary Figure 103 ^{13}C NMR (101 MHz, $CDCl_3$) of **5d**

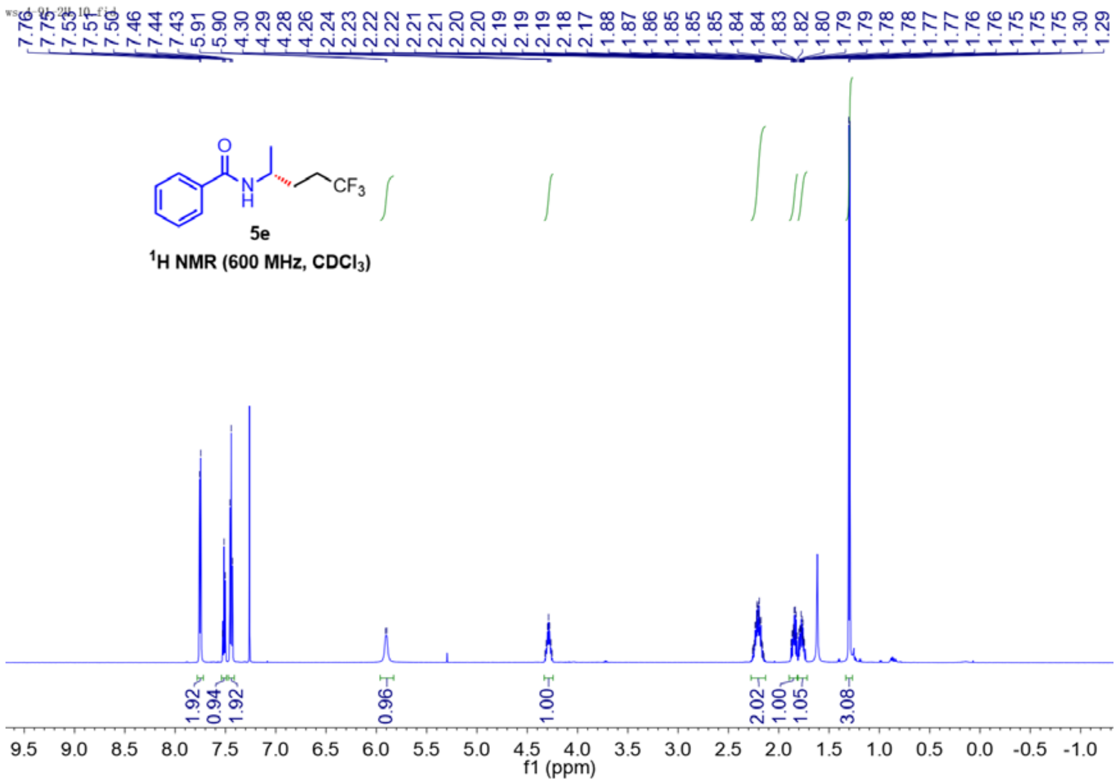

Supplementary Figure 104 ^{1}H NMR (600 MHz, $CDCl_3$) of **5e**

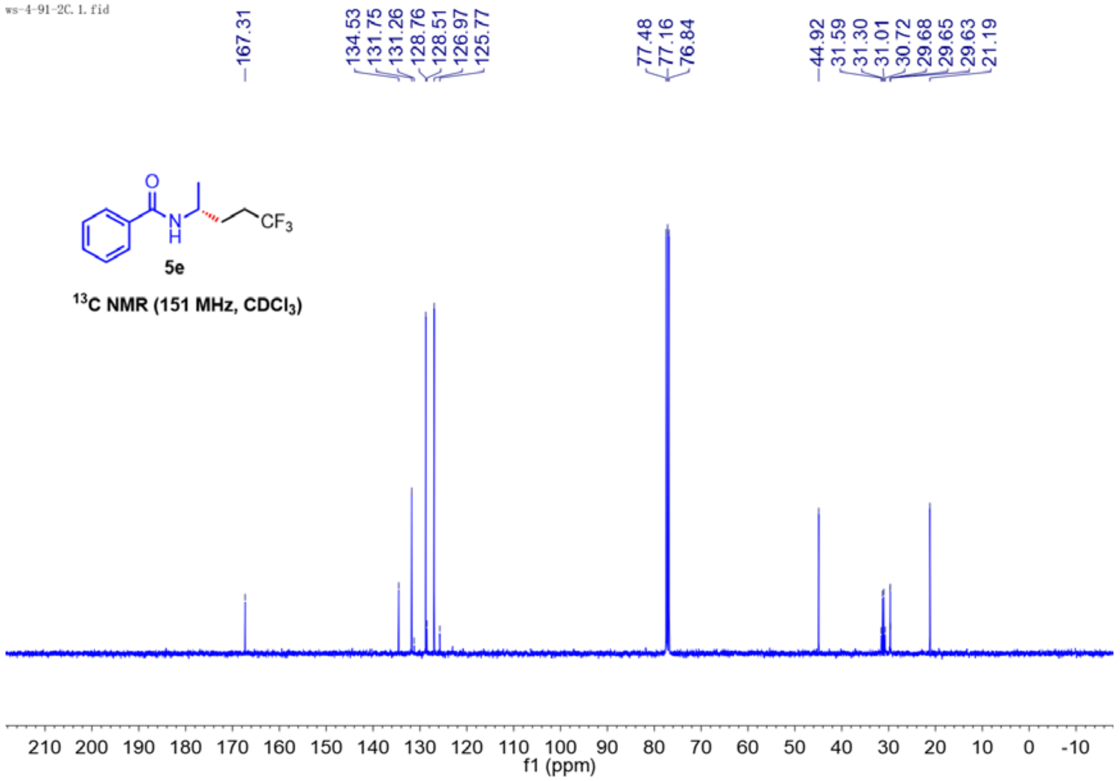

Supplementary Figure 105 ^{13}C NMR (151 MHz, $CDCl_3$) of **5e**

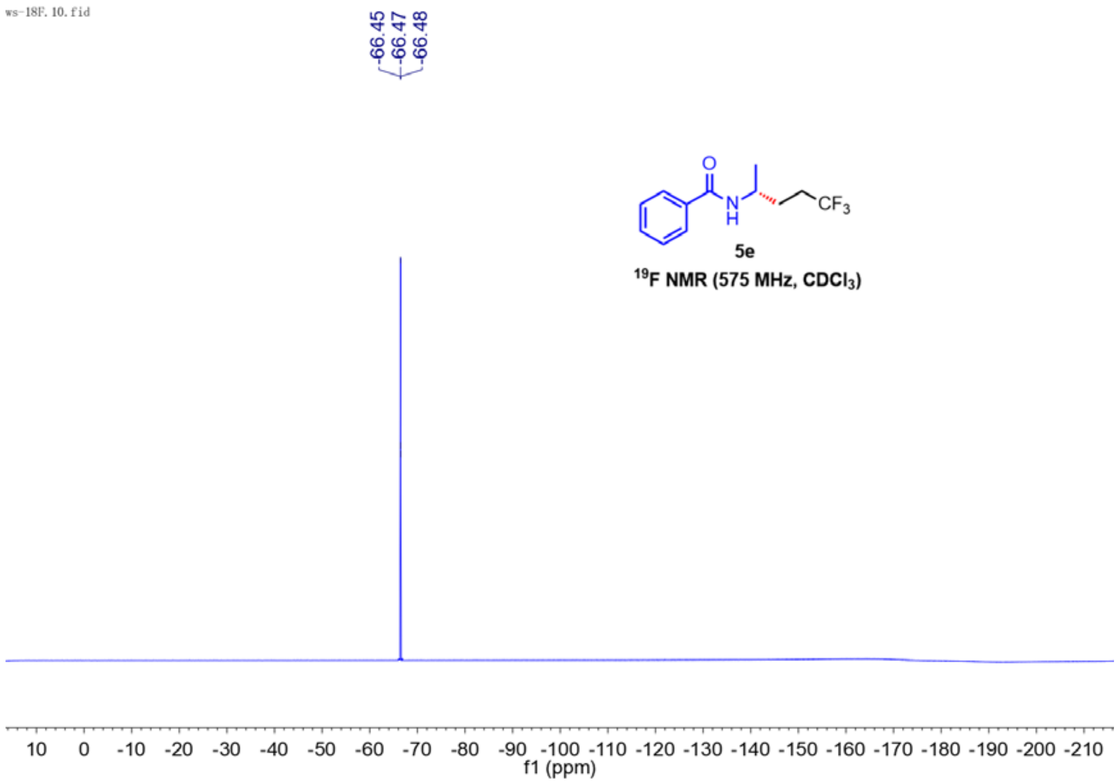

Supplementary Figure 106 ^{19}F NMR (575 MHz, $CDCl_3$) of **5e**

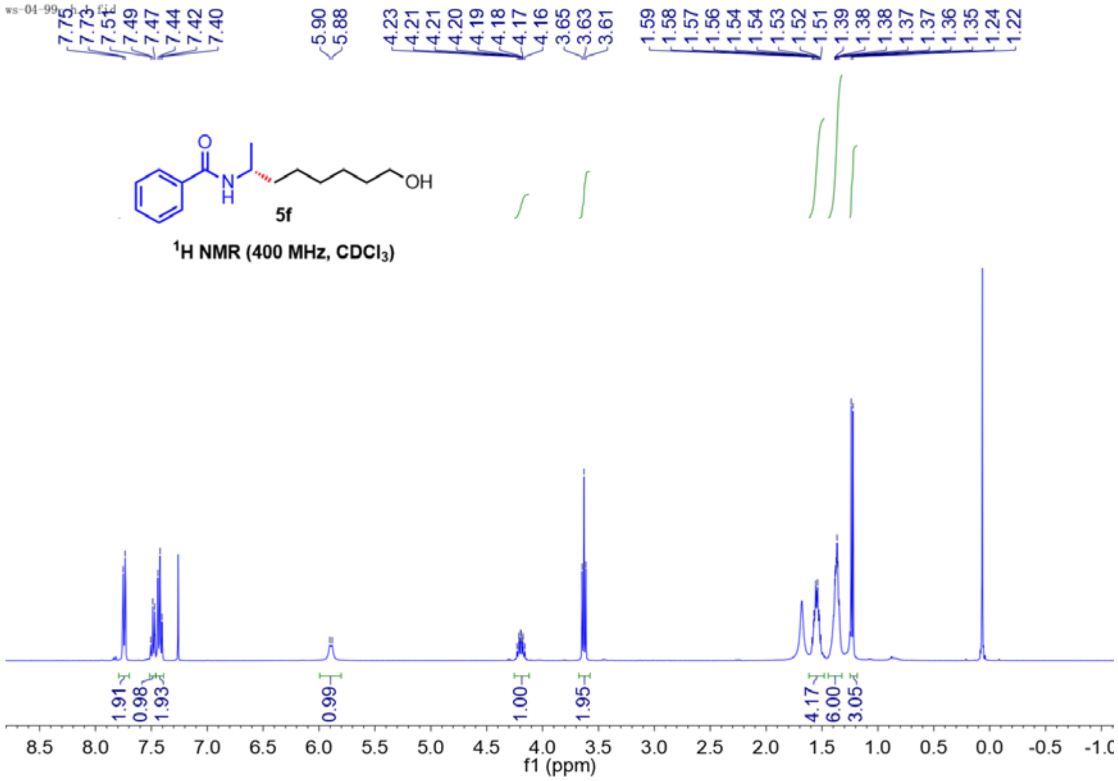

Supplementary Figure 107 ^{1}H NMR (400 MHz, $CDCl_3$) of **5f**

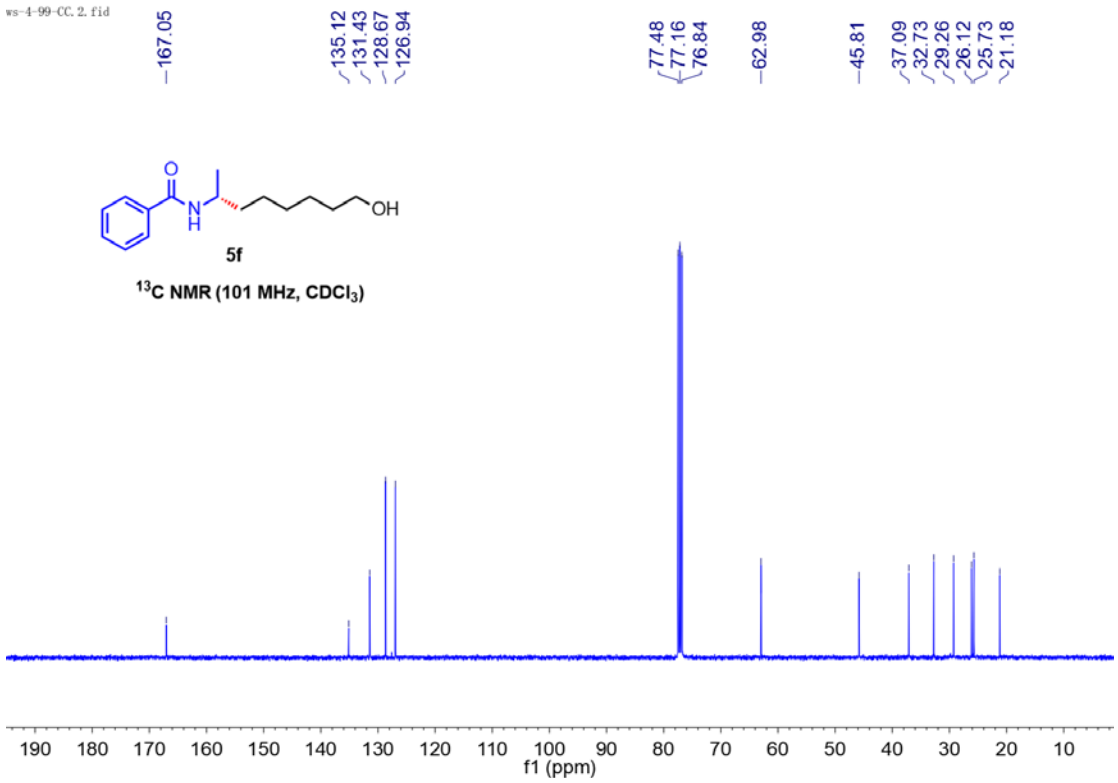

Supplementary Figure 108 ^{13}C NMR (101 MHz, $CDCl_3$) of **5f**

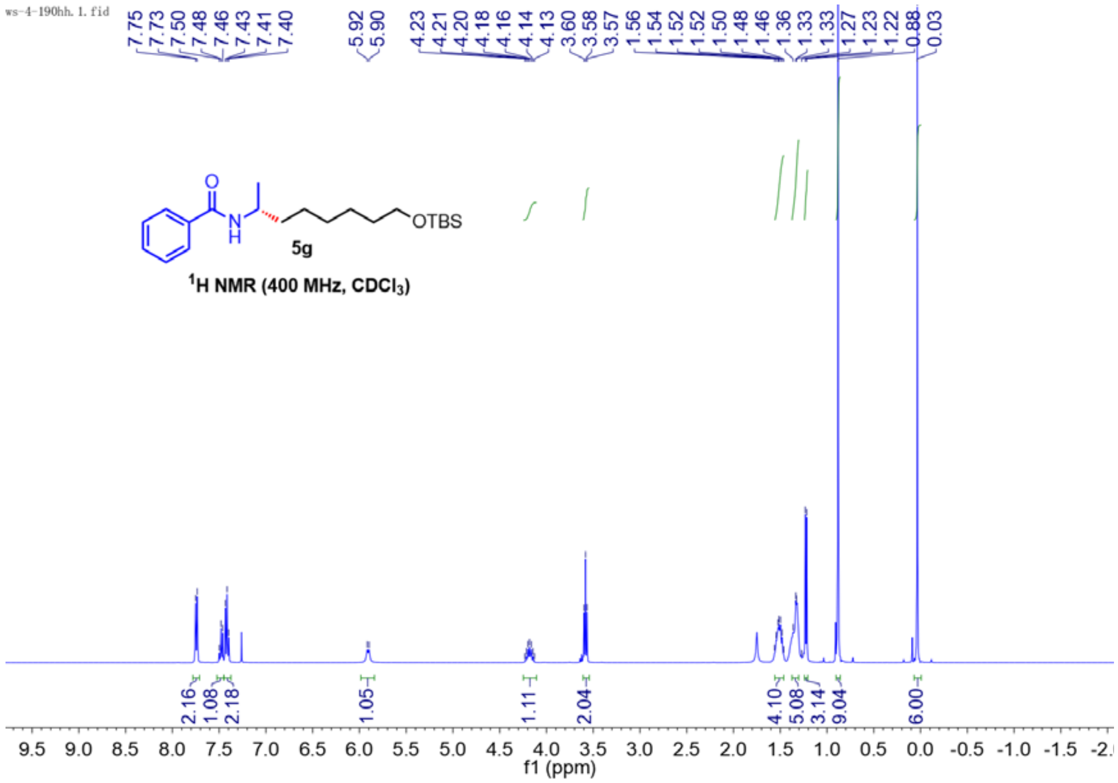

Supplementary Figure 109 ^{1}H NMR (400 MHz, $CDCl_3$) of **5g**

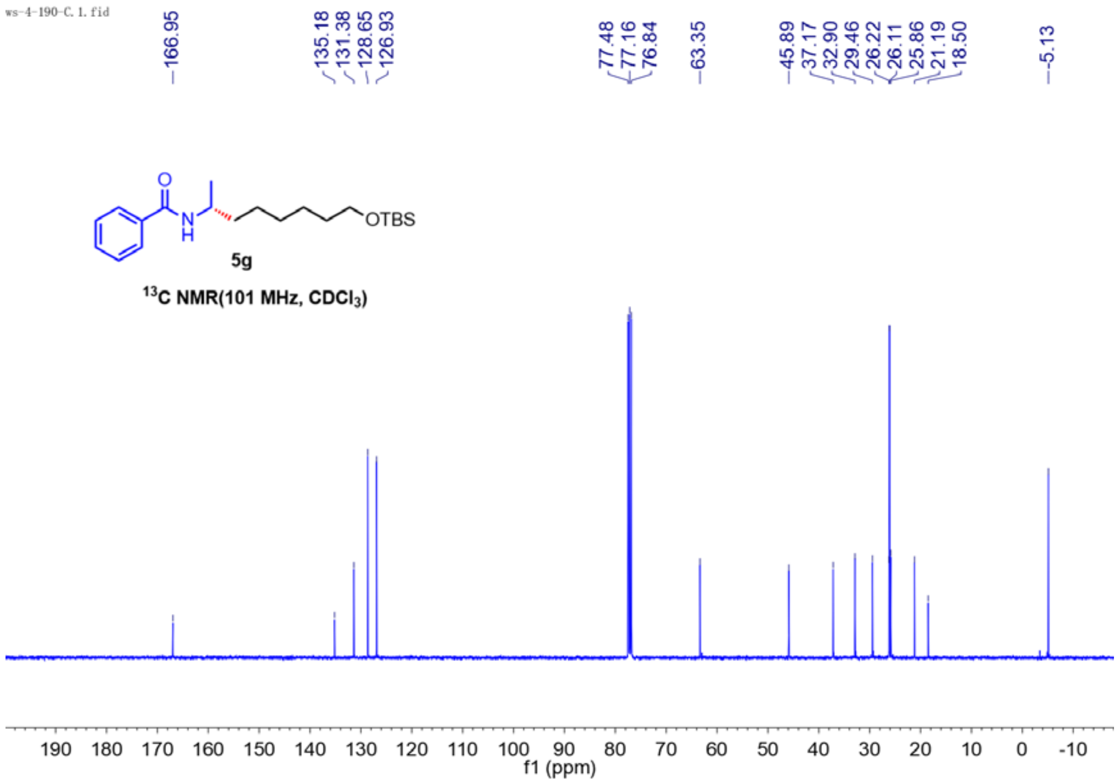

Supplementary Figure 110 ^{13}C NMR (101 MHz, $CDCl_3$) of **5g**

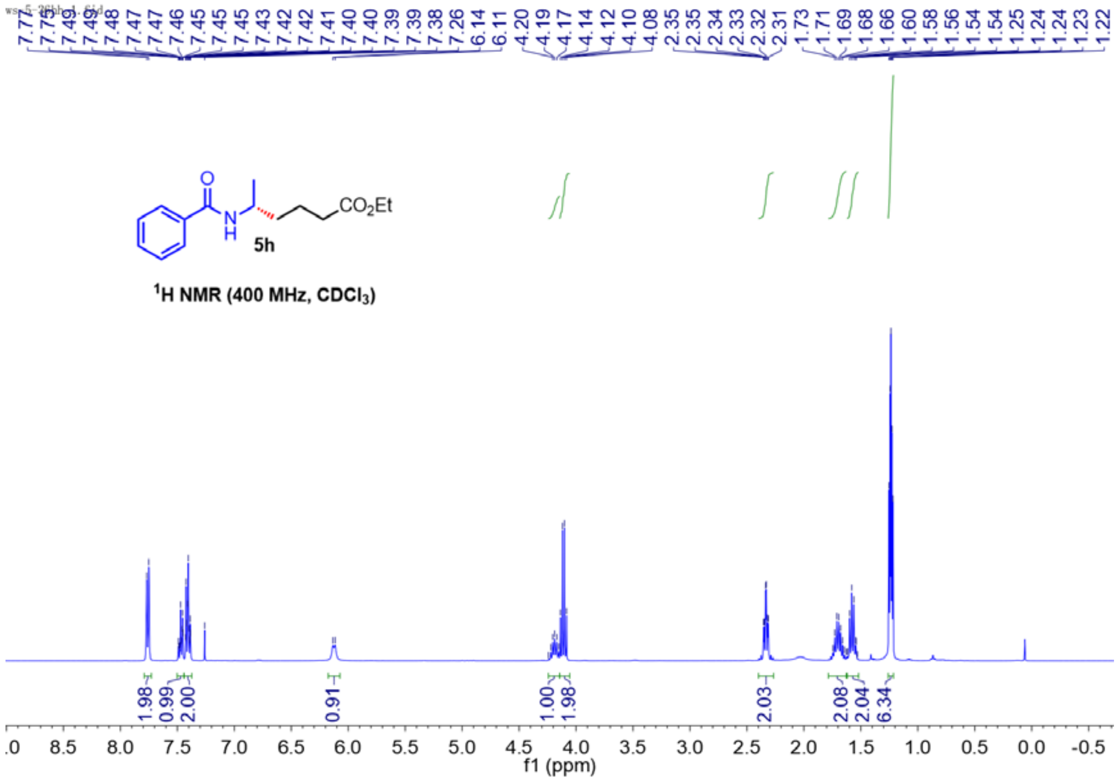

Supplementary Figure 111 ^{1}H NMR (400 MHz, $CDCl_3$) of **5h**

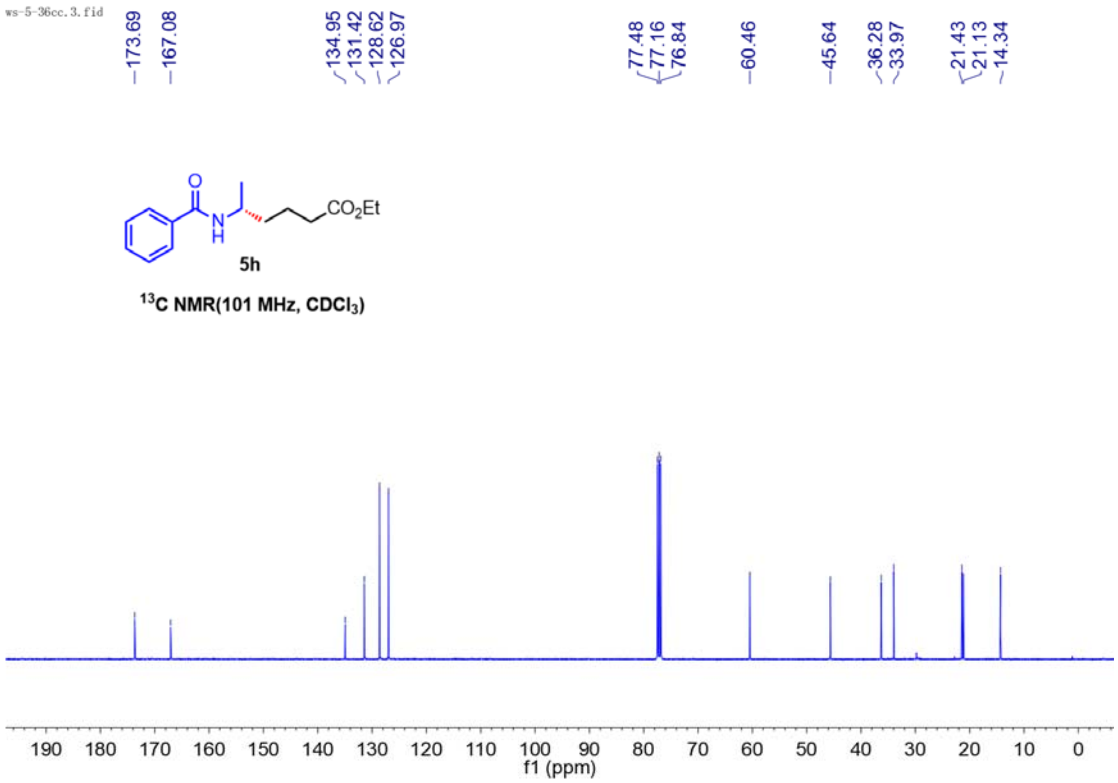

Supplementary Figure 112 ^{13}C NMR (101 MHz, $CDCl_3$) of **5h**

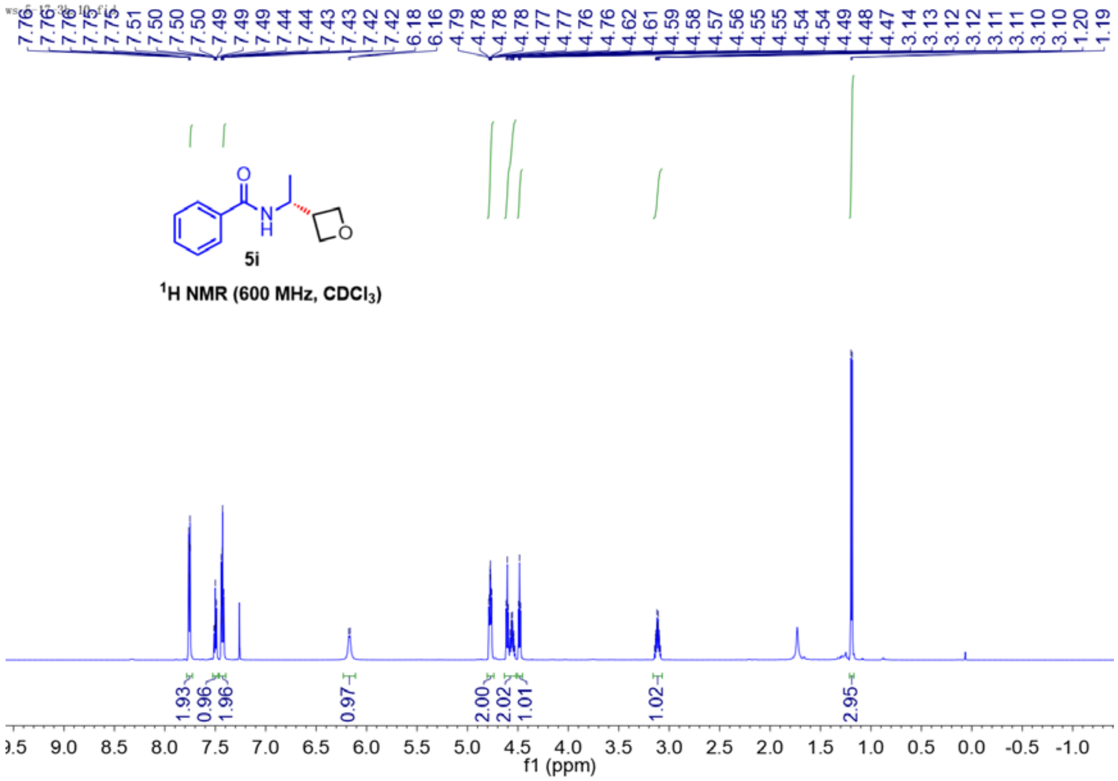

Supplementary Figure 113 ^{1}H NMR (600 MHz, $CDCl_3$) of **5i**

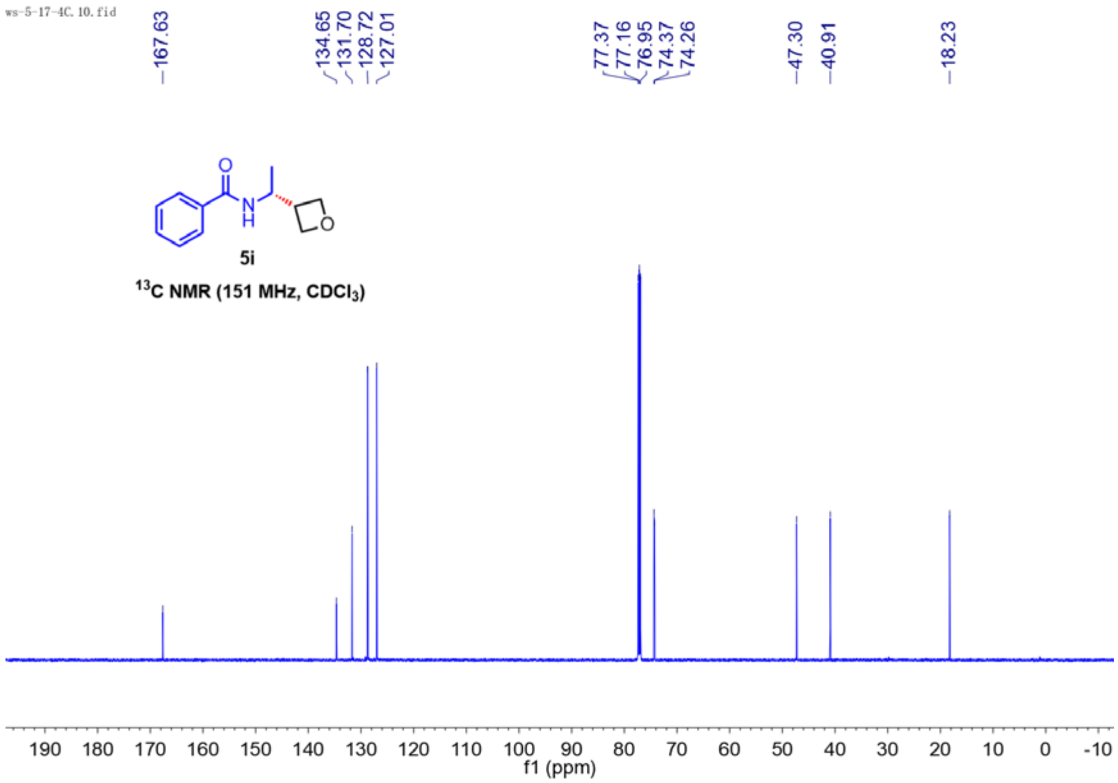

Supplementary Figure 114 ^{13}C NMR (151 MHz, $CDCl_3$) of **5i**

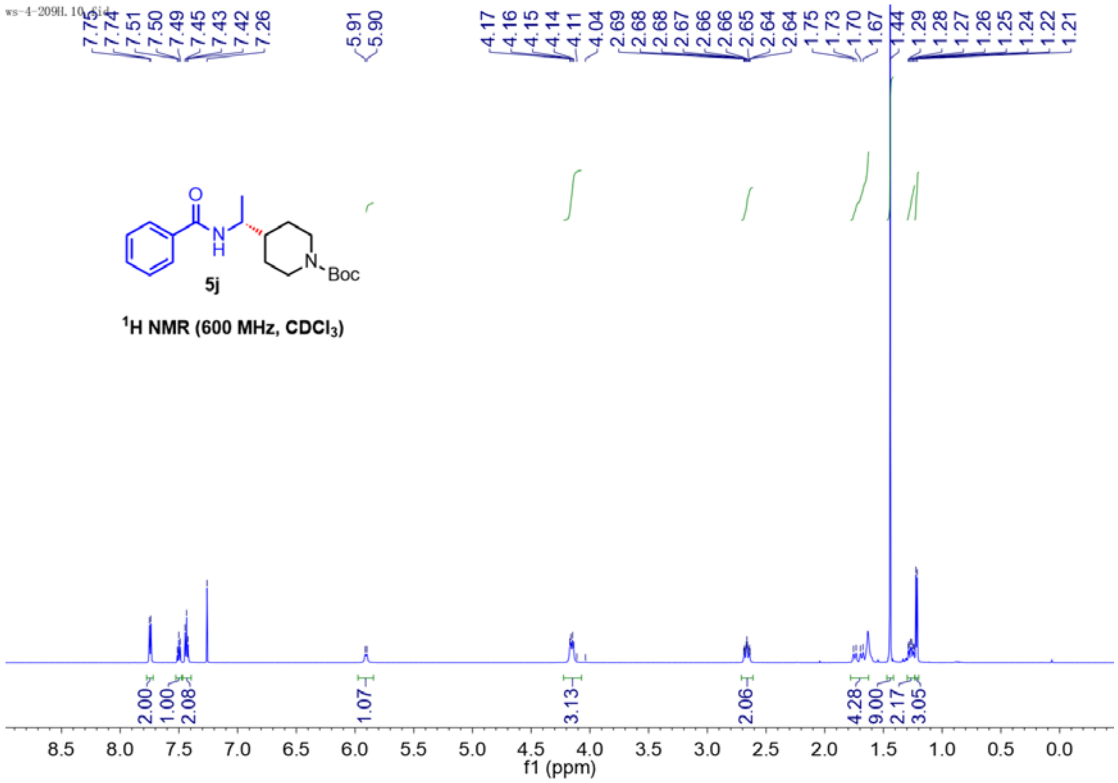

Supplementary Figure 115 ^{1}H NMR (600 MHz, $CDCl_3$) of **5j**

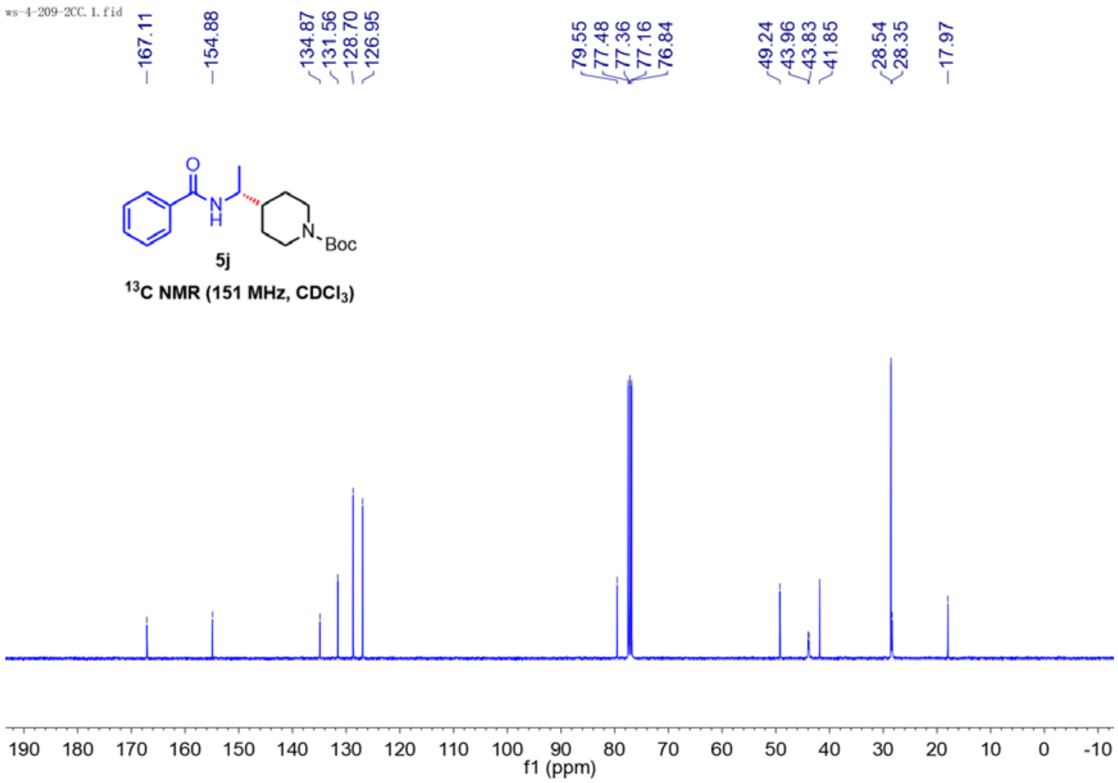

Supplementary Figure 116 ^{13}C NMR (151 MHz, $CDCl_3$) of **5j**

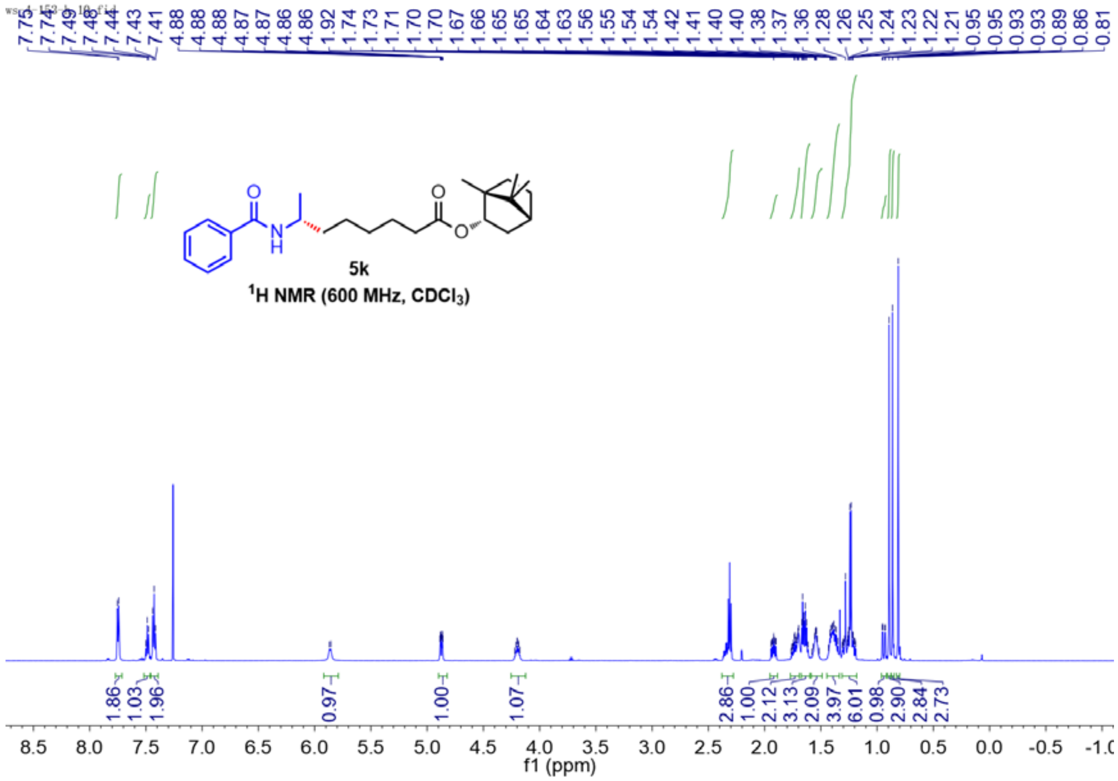

Supplementary Figure 117 ^{1}H NMR (600 MHz, $CDCl_3$) of **5k**

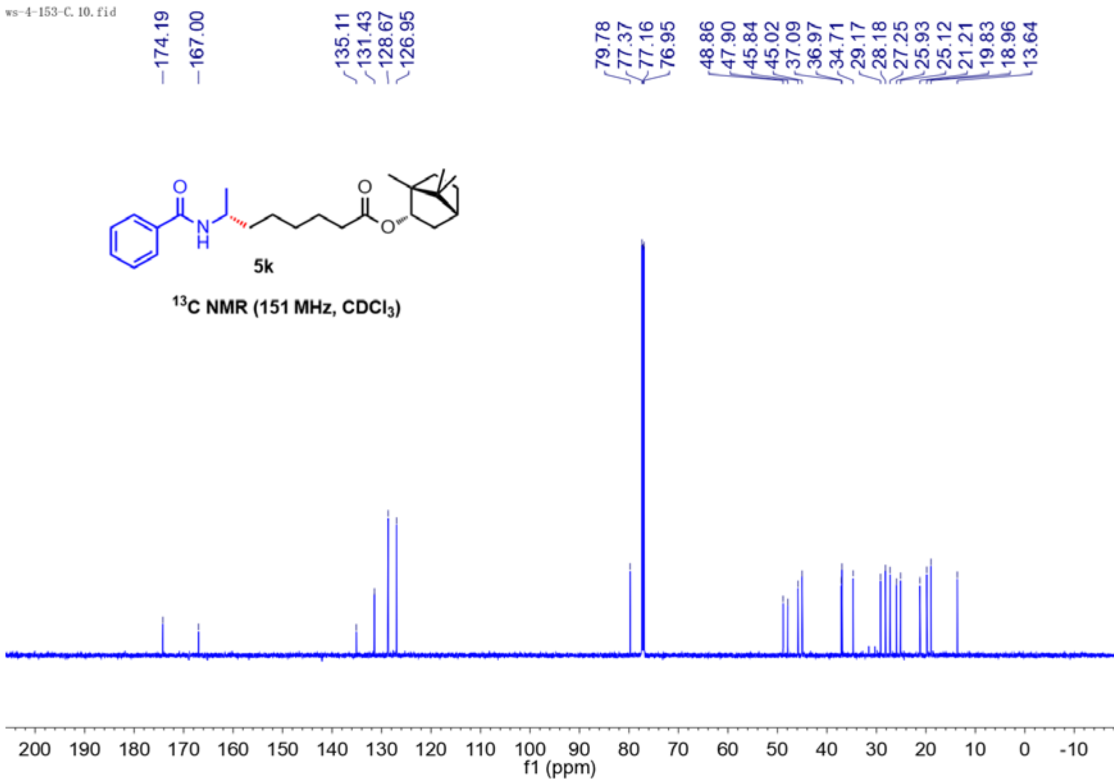

Supplementary Figure 118 ^{13}C NMR (151 MHz, $CDCl_3$) of **5k**

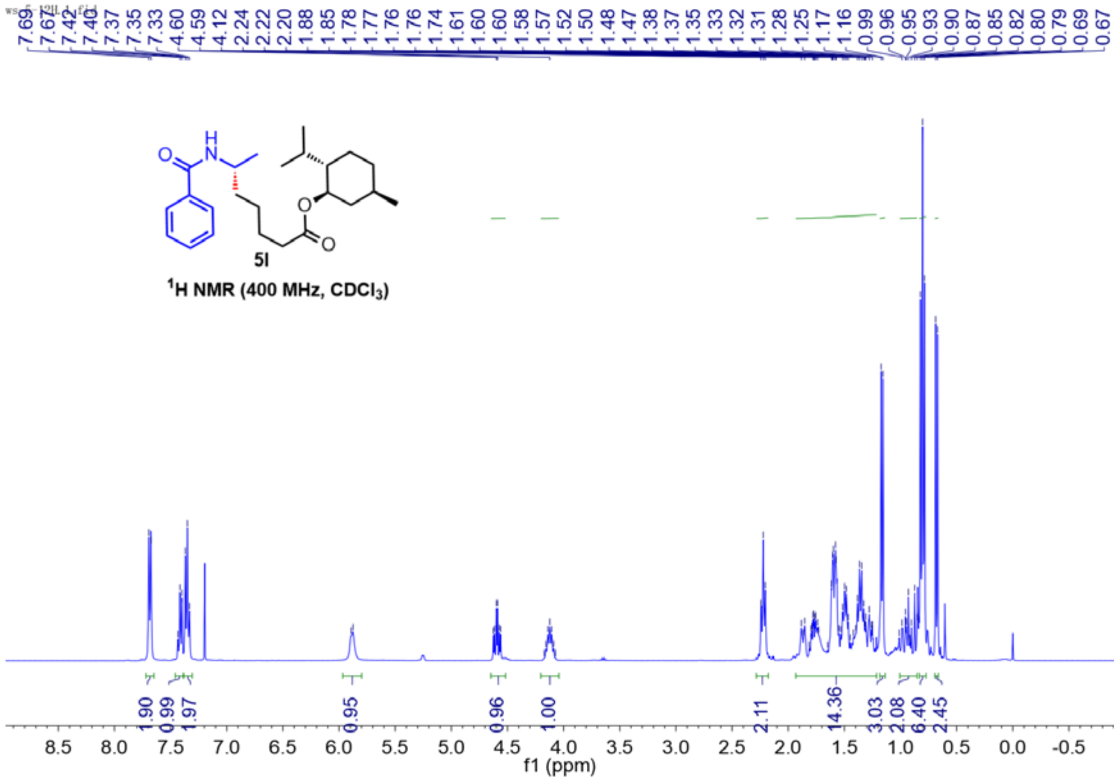

Supplementary Figure 119 ^{1}H NMR (400 MHz, $CDCl_3$) of **5l**

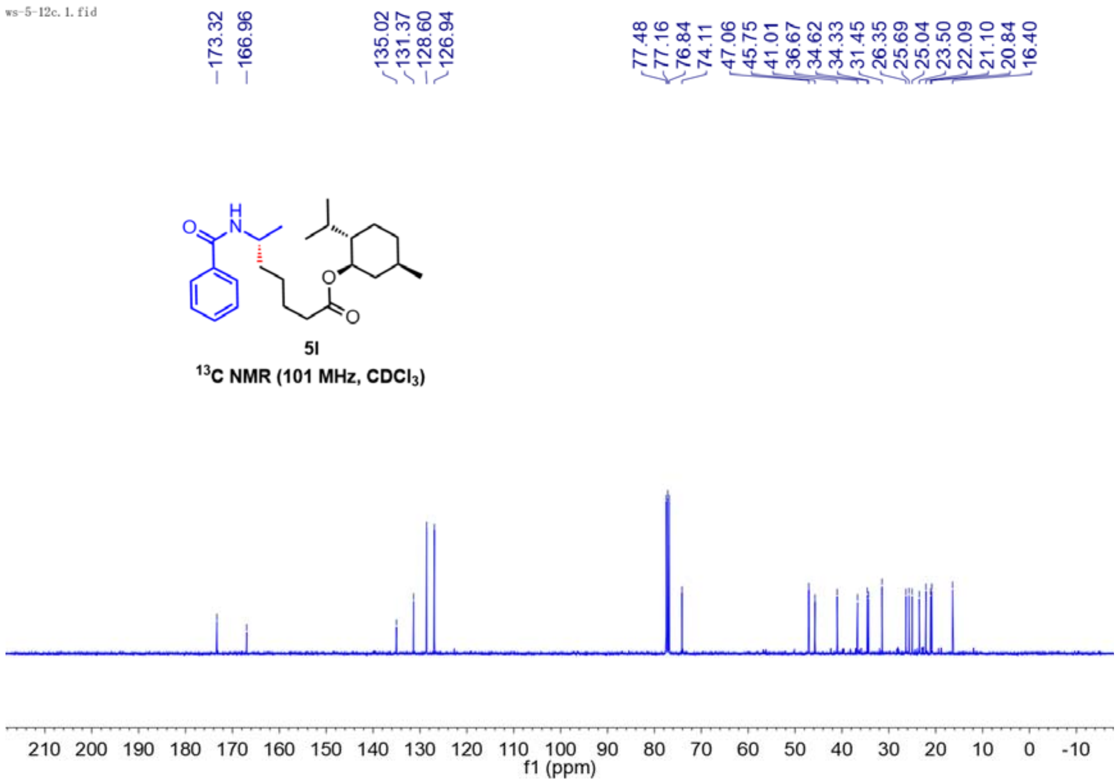

Supplementary Figure 120 ^{13}C NMR (101 MHz, $CDCl_3$) of **5l**

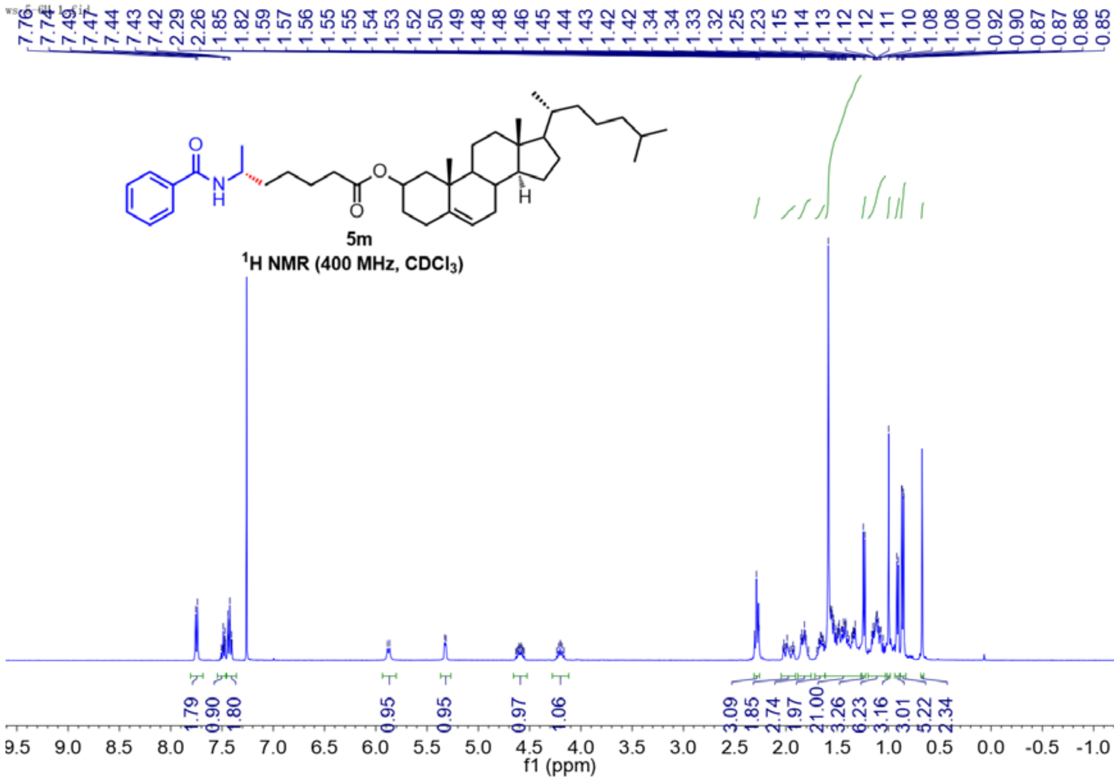

Supplementary Figure 121 ^{1}H NMR (400 MHz, $CDCl_3$) of **5m**

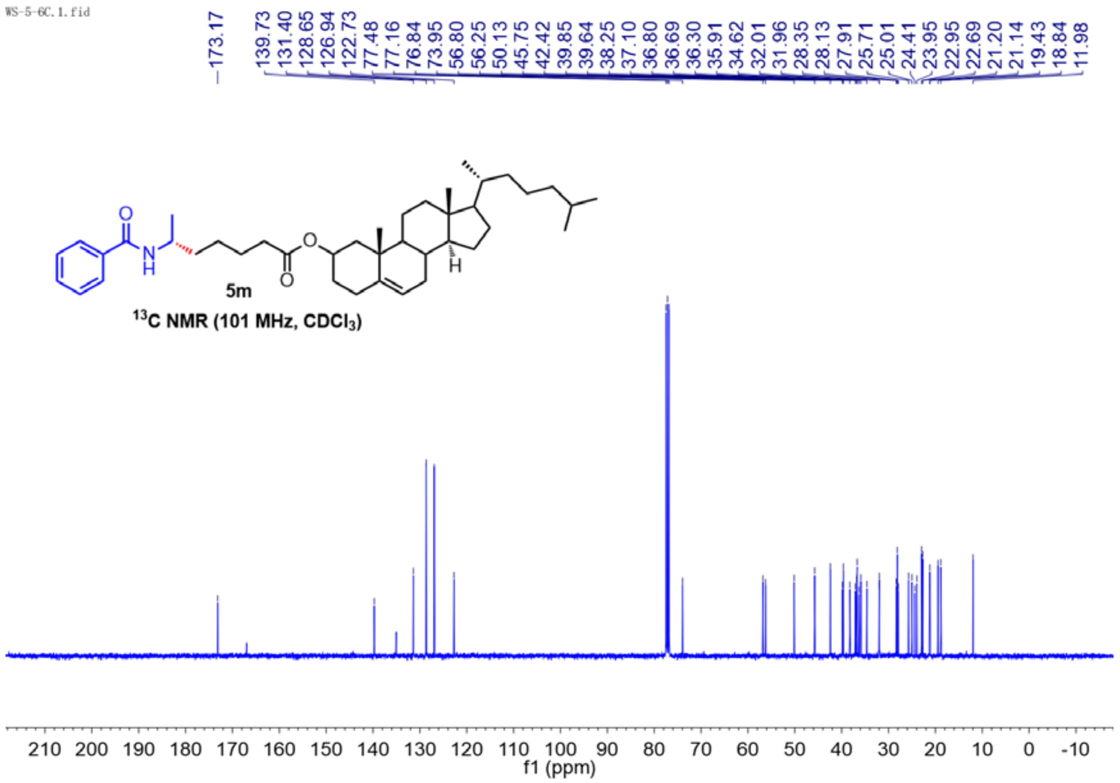

Supplementary Figure 122 ^{13}C NMR (101 MHz, CDCl$_3$) of **5m**

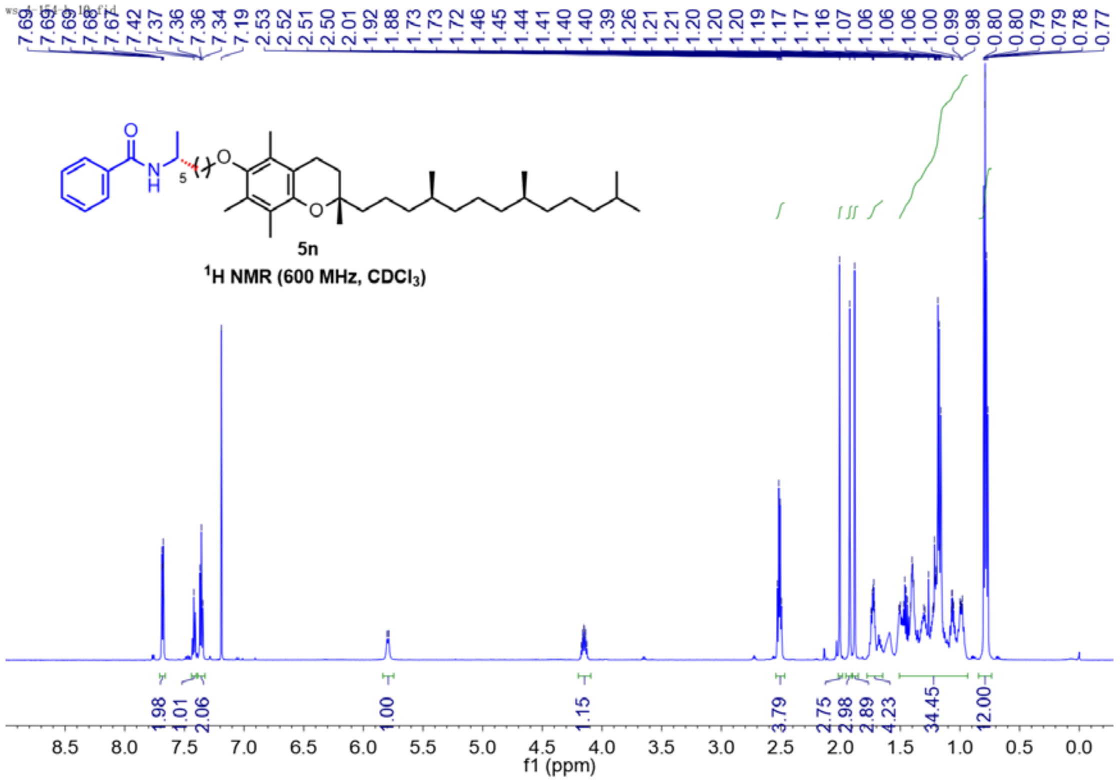

Supplementary Figure 123 ^{1}H NMR (600 MHz, CDCl$_3$) of **5n**

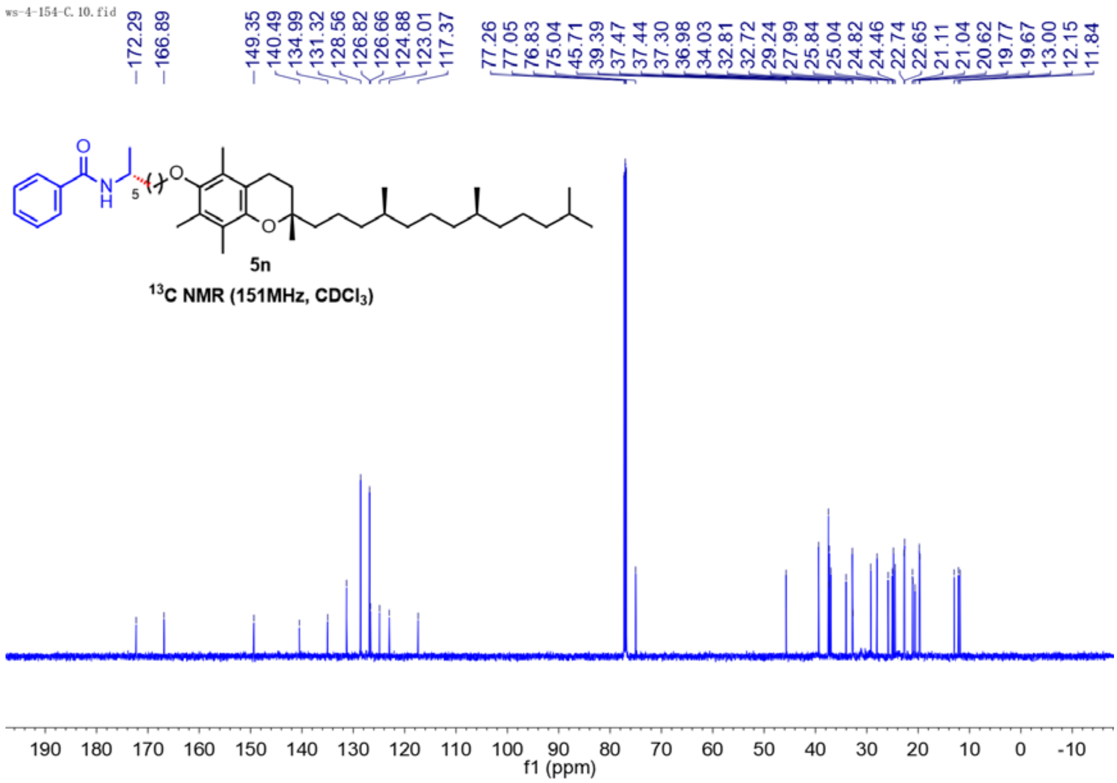

Supplementary Figure 124 ^{13}C NMR (151 MHz, $CDCl_3$) of **5n**

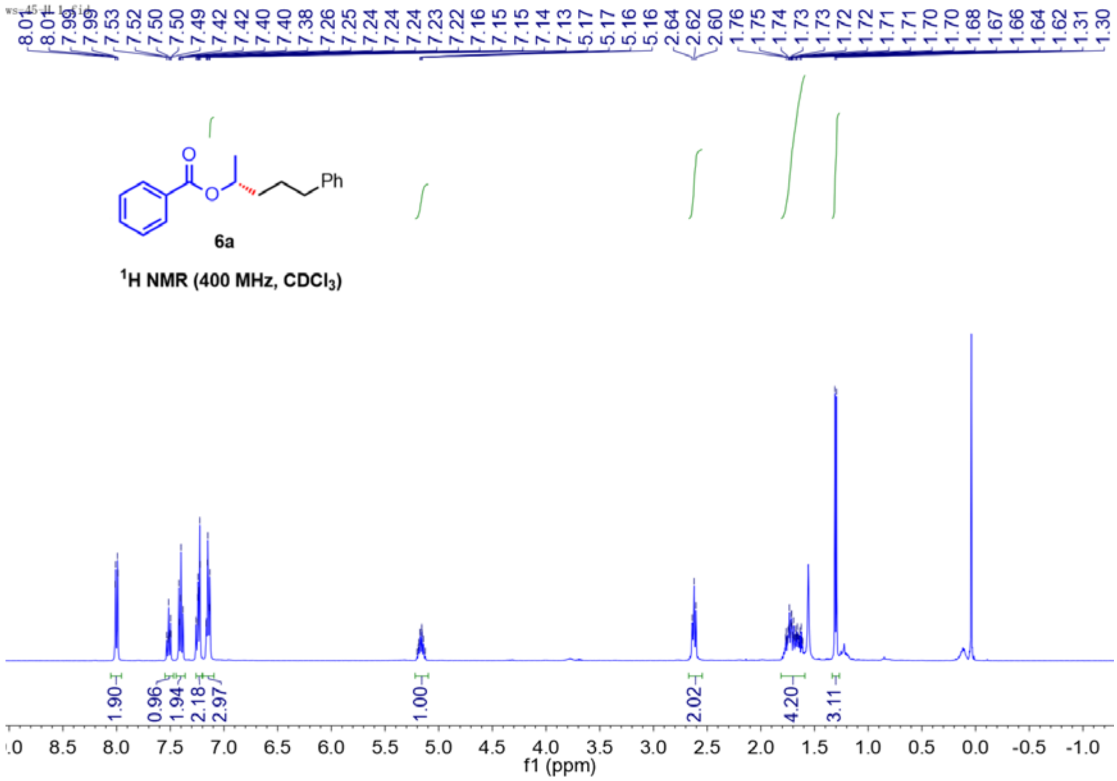

Supplementary Figure 125 ^{1}H NMR (400 MHz, $CDCl_3$) of **6a**

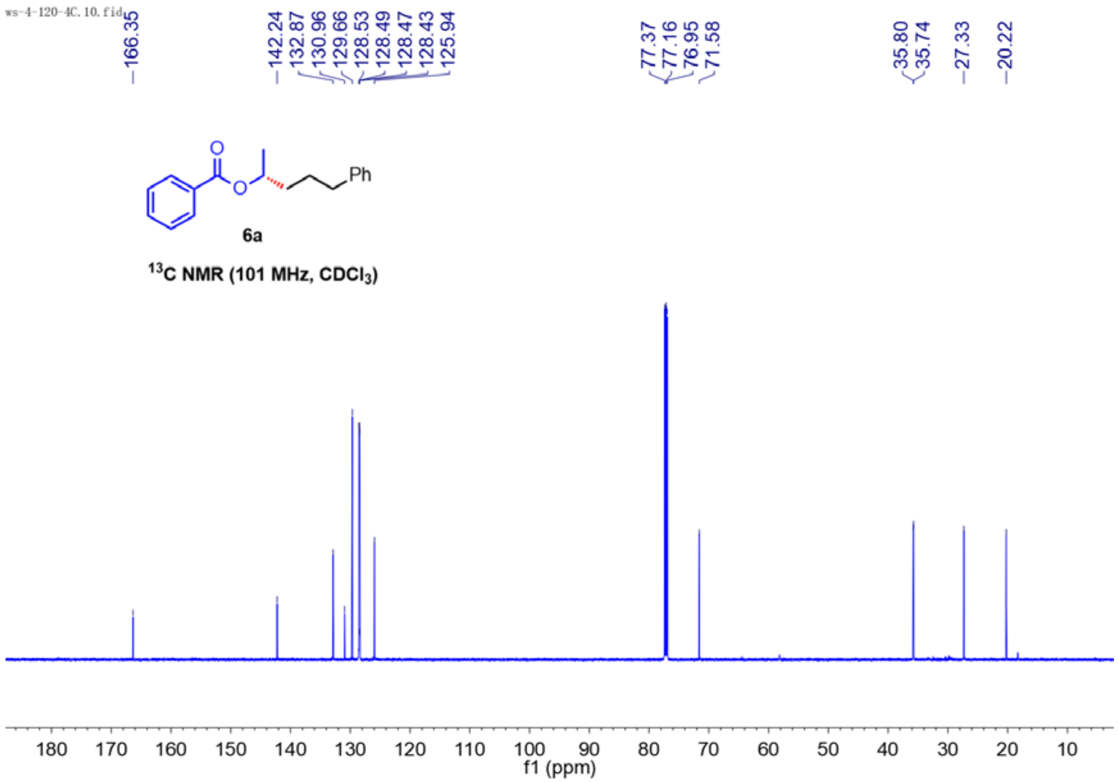

Supplementary Figure 126 ^{13}C NMR (101 MHz, $CDCl_3$) of **6a**

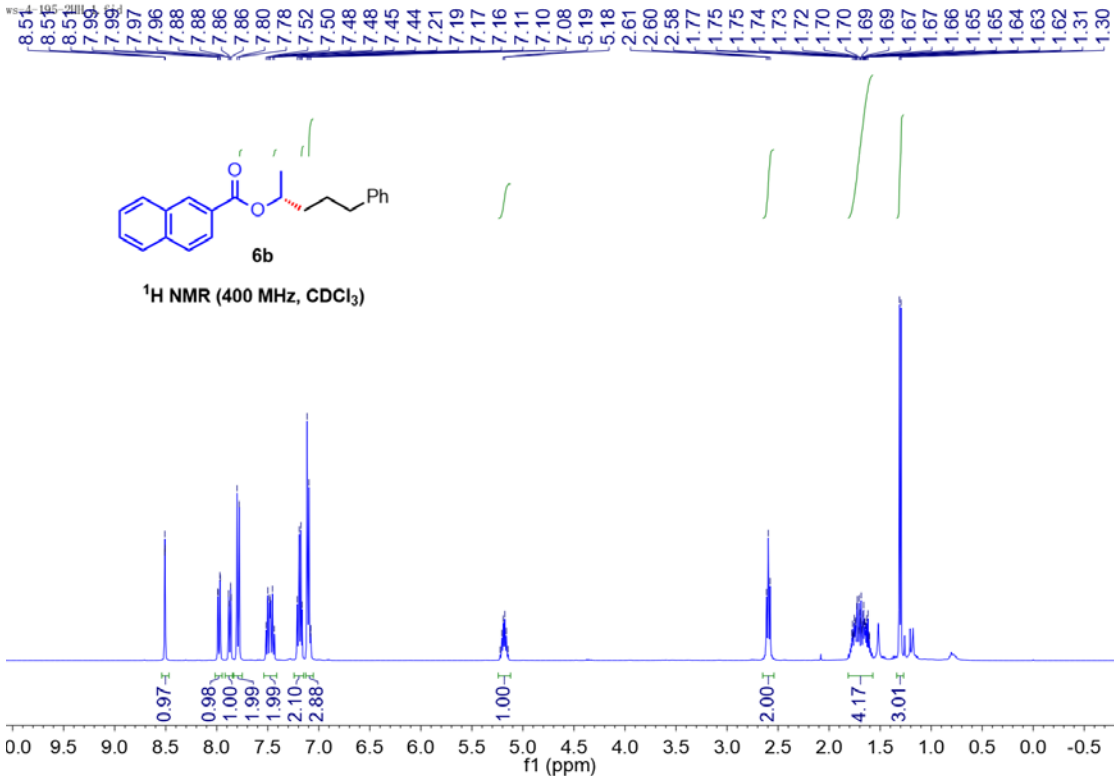

Supplementary Figure 127 ^{1}H NMR (400 MHz, $CDCl_3$) of **6b**

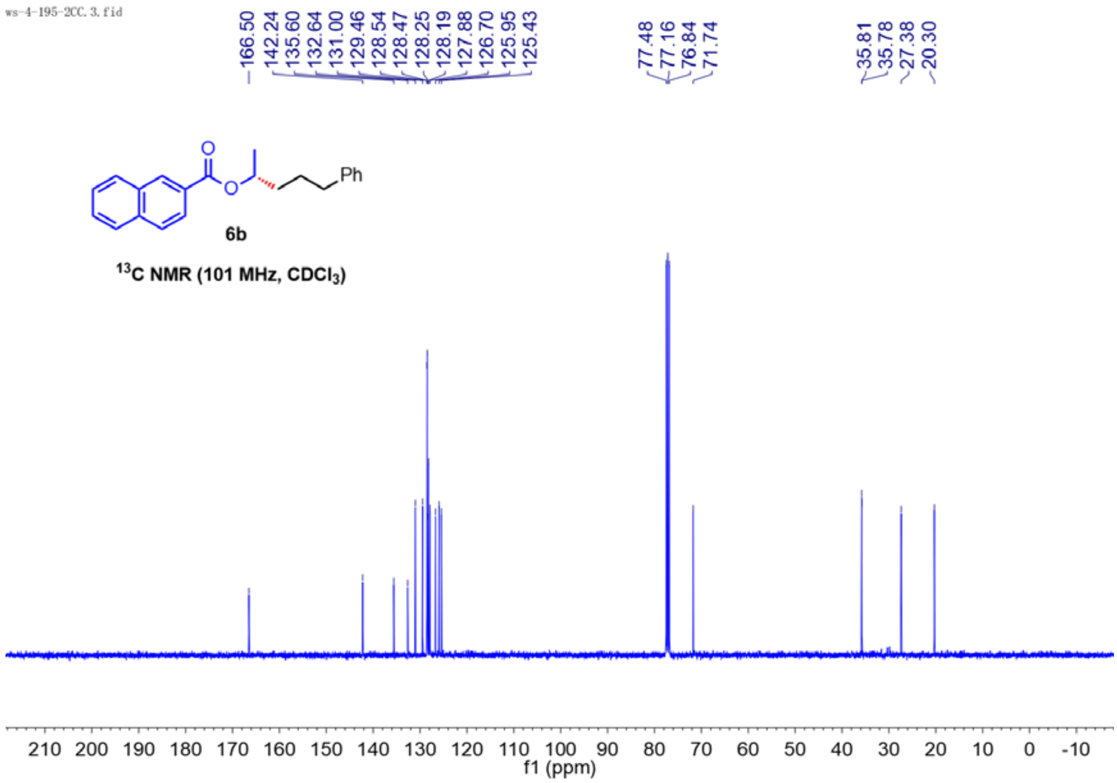

Supplementary Figure 128 ^{13}C NMR (101 MHz, $CDCl_3$) of **6b**

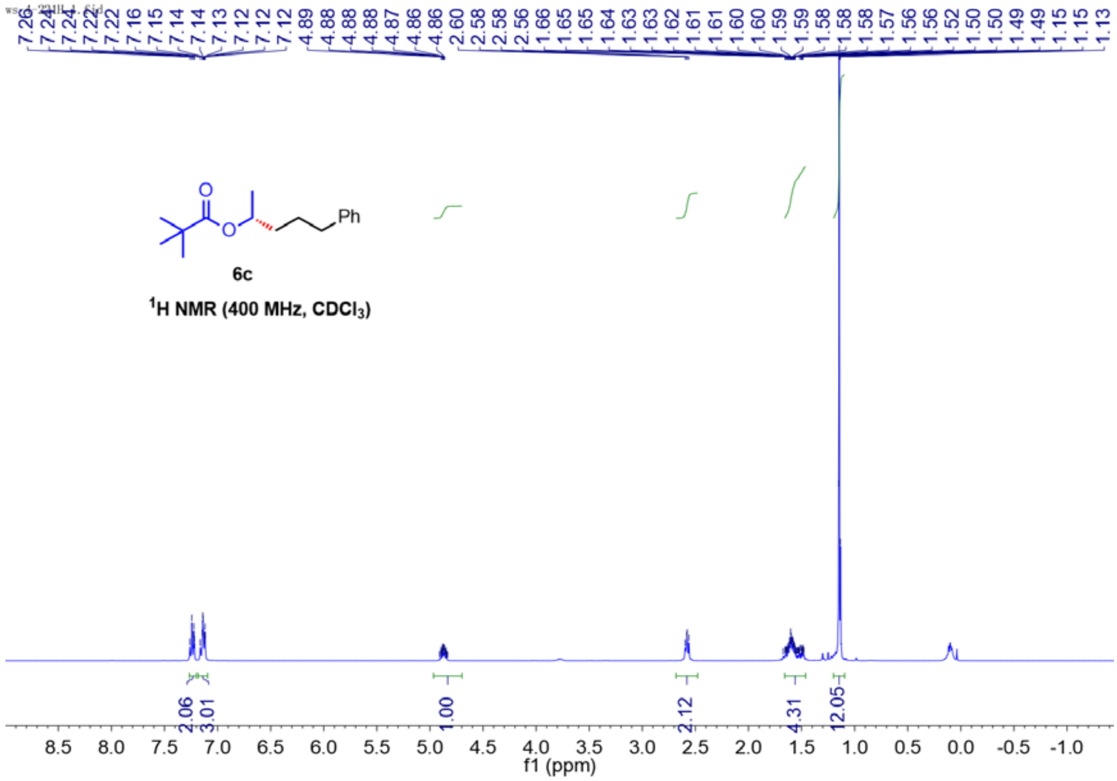

Supplementary Figure 129 ^{1}H NMR (400 MHz, $CDCl_3$) of **6c**

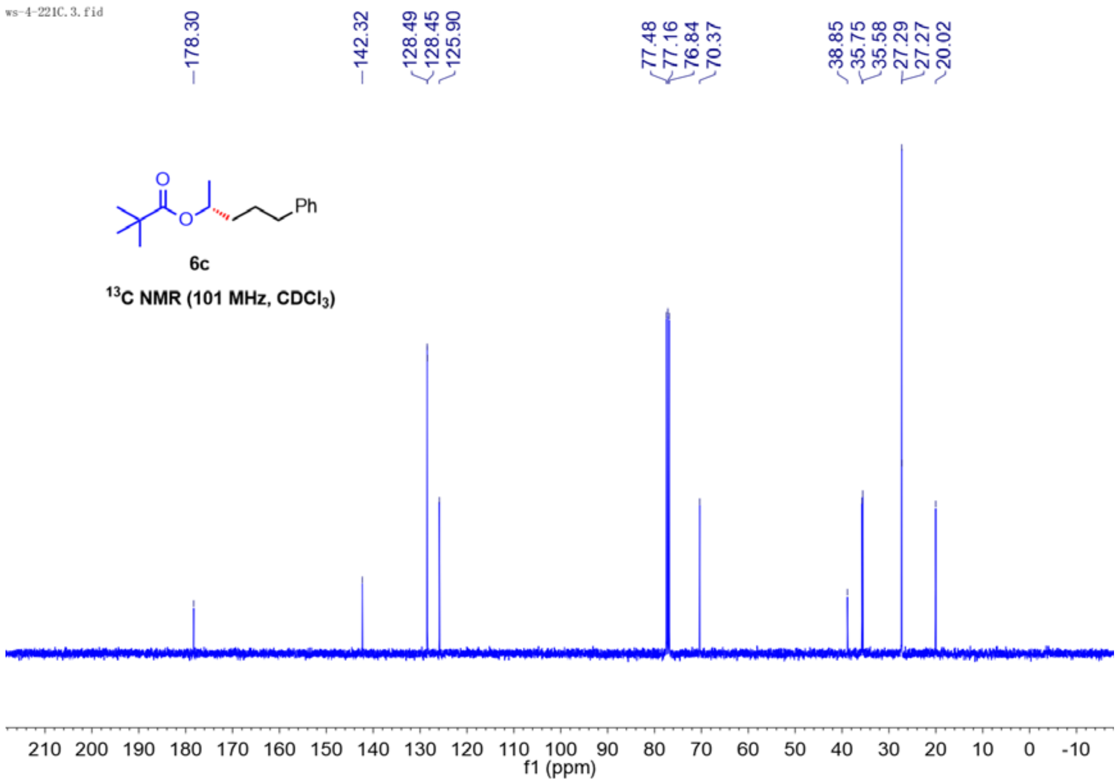

Supplementary Figure 130 ^{13}C NMR (101 MHz, $CDCl_3$) of **6c**

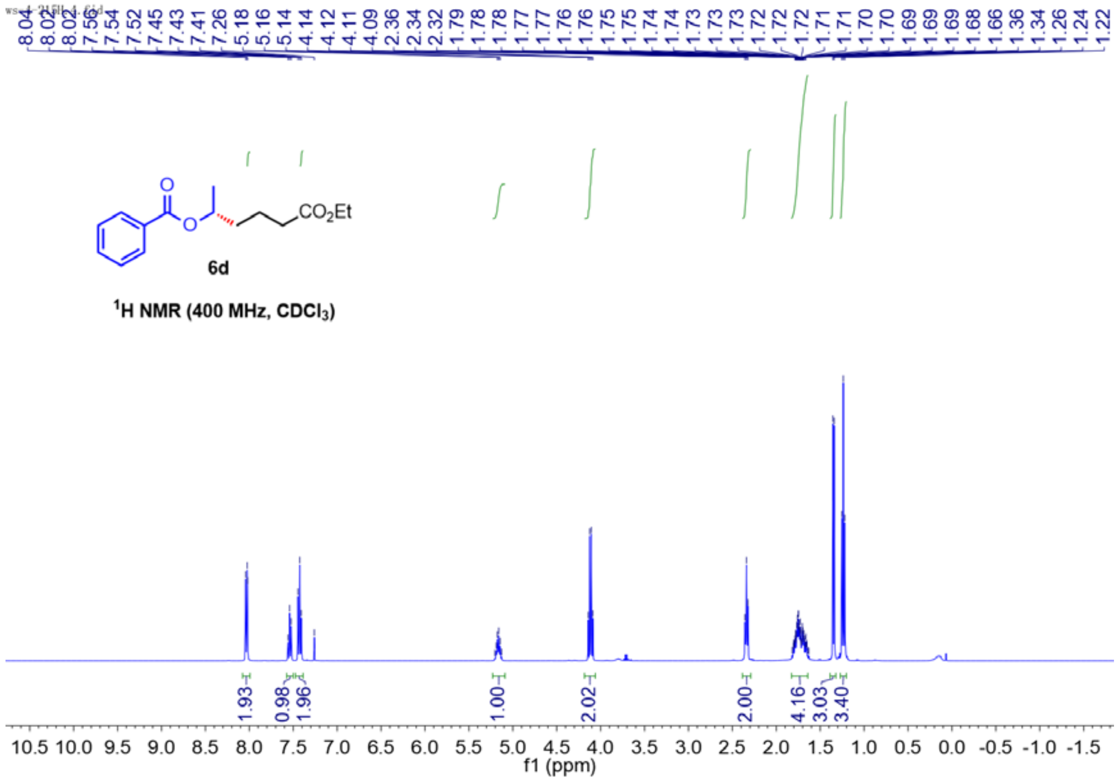

Supplementary Figure 131 ^{1}H NMR (400 MHz, $CDCl_3$) of **6d**

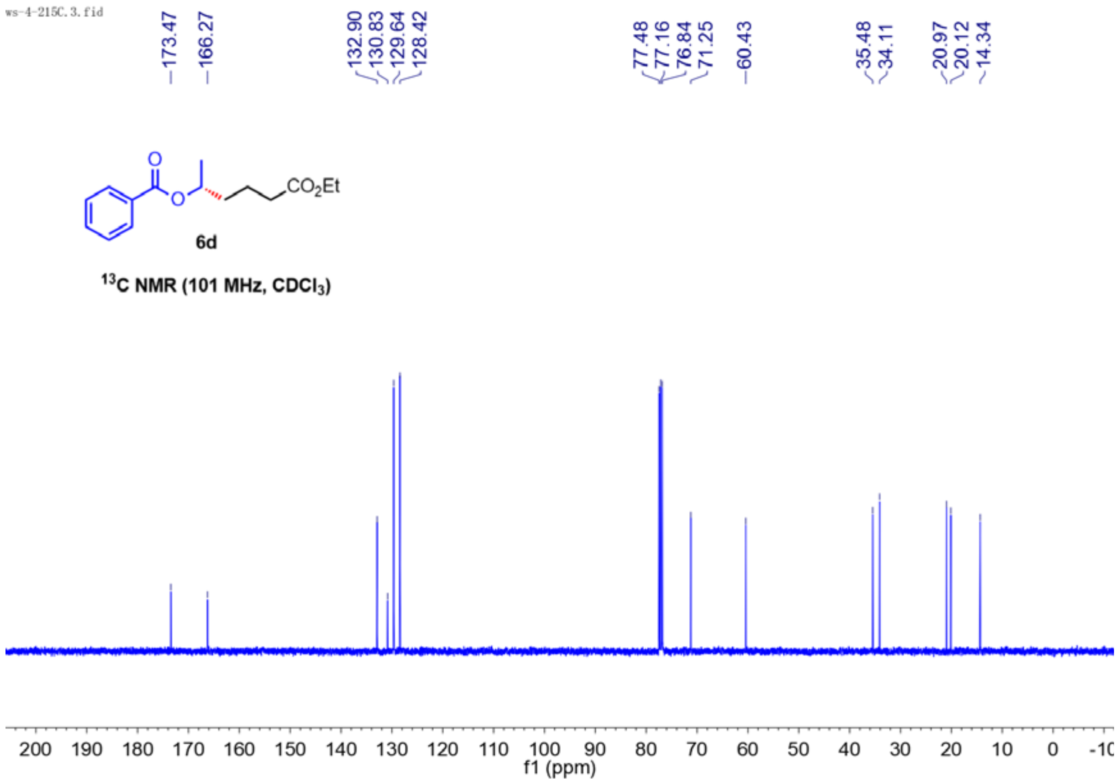

Supplementary Figure 132 ^{13}C NMR (101 MHz, $CDCl_3$) of **6d**

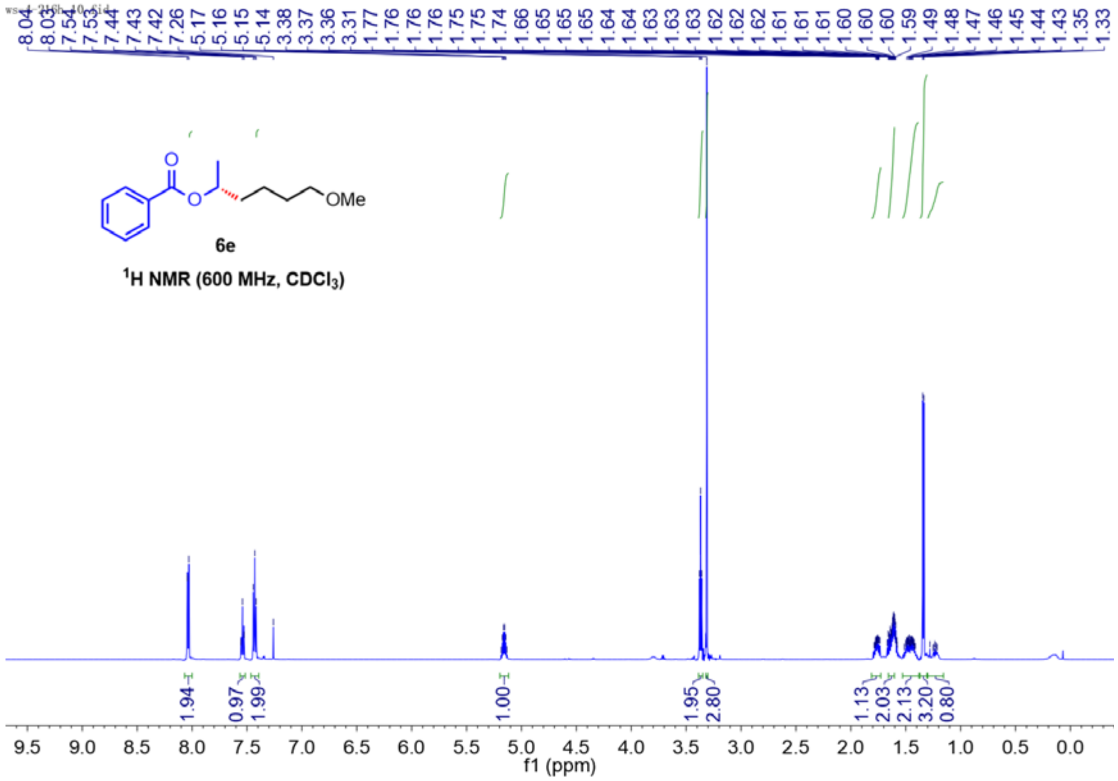

Supplementary Figure 133 ^{1}H NMR (600 MHz, $CDCl_3$) of **6e**

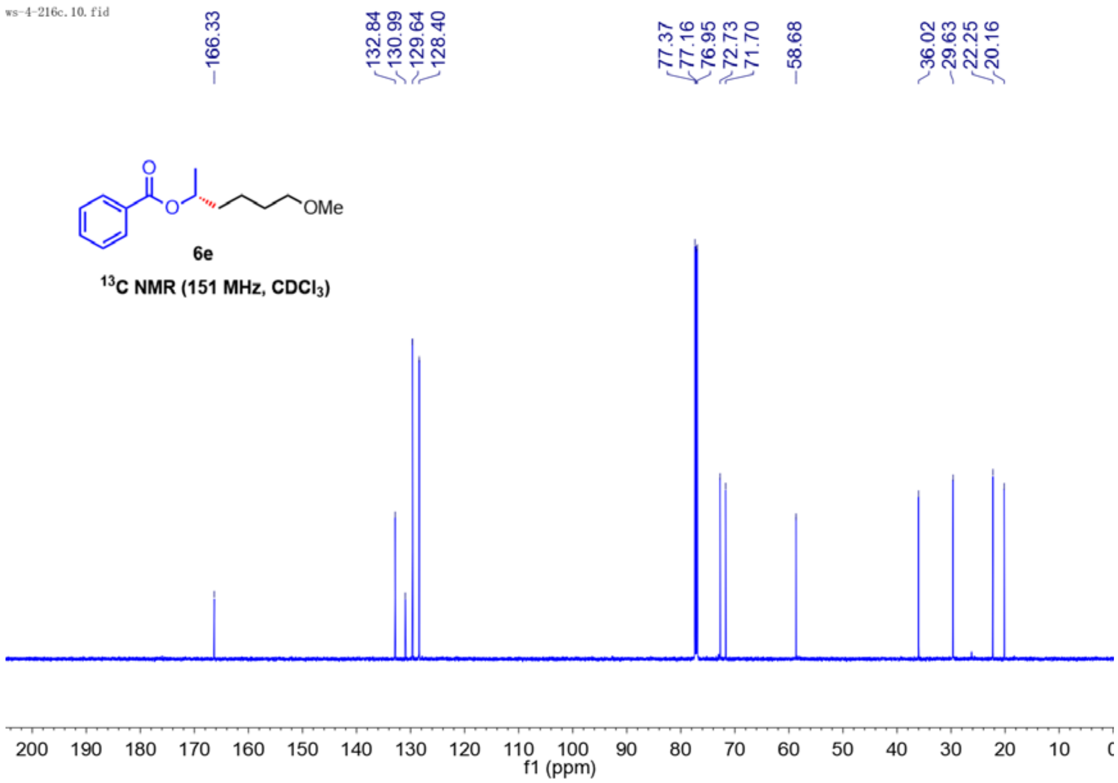

Supplementary Figure 134 ^{13}C NMR (151 MHz, $CDCl_3$) of **6e**

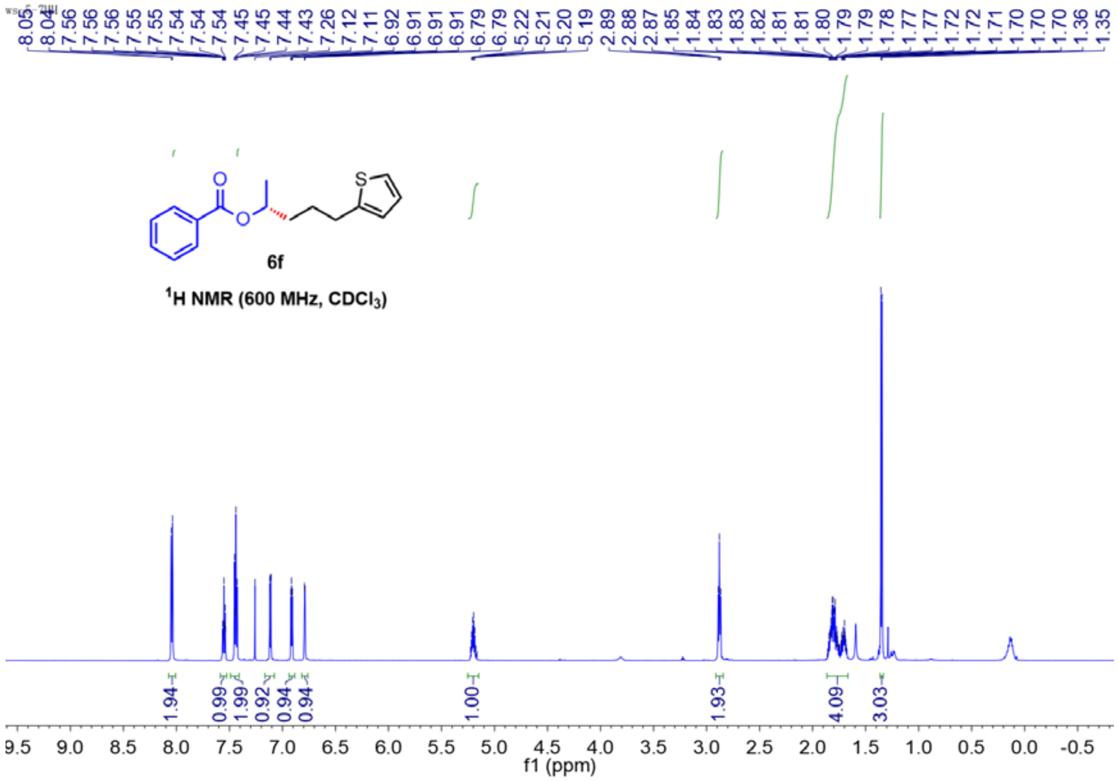

Supplementary Figure 135 ^{1}H NMR (600 MHz, $CDCl_3$) of **6f**

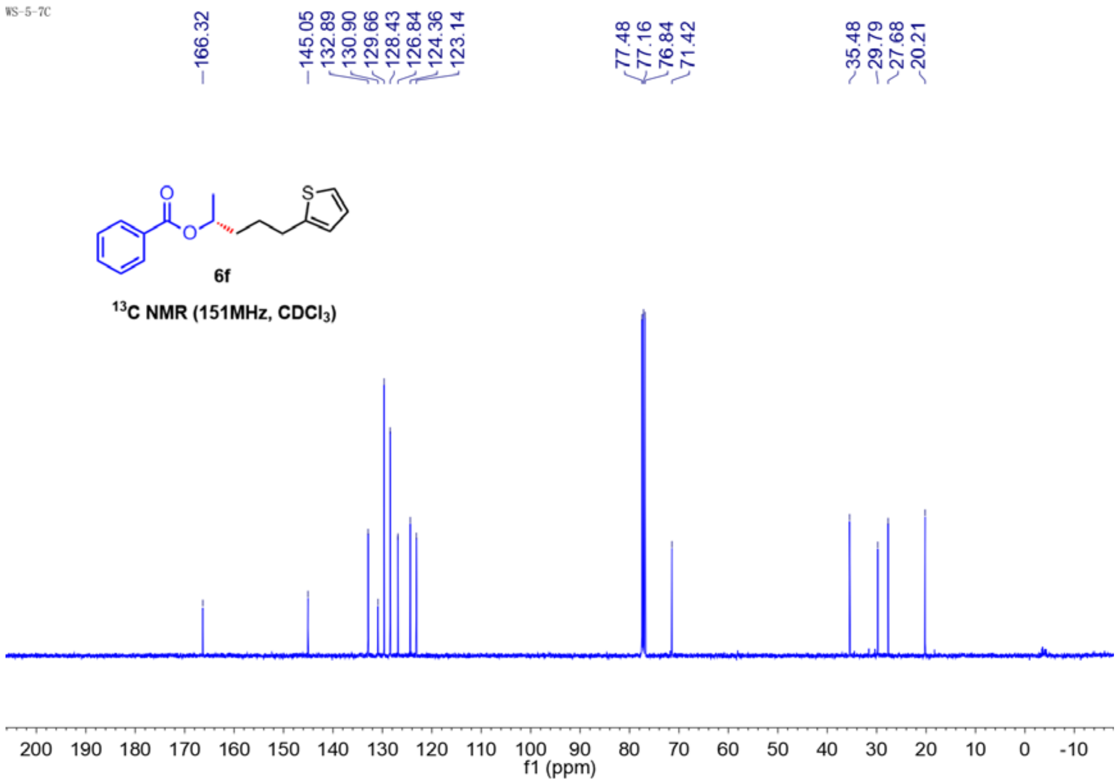

Supplementary Figure 136 ^{13}C NMR (151 MHz, CDCl$_3$) of **6f**

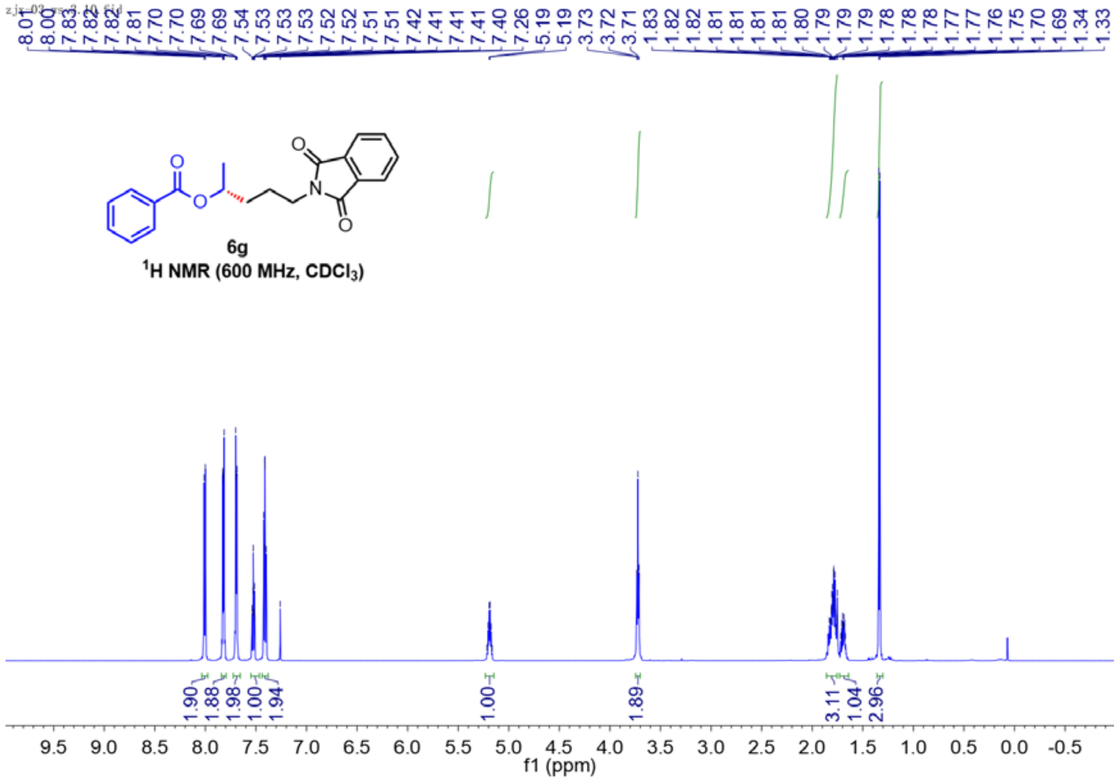

Supplementary Figure 137 ^{1}H NMR (600 MHz, CDCl$_3$) of **6g**

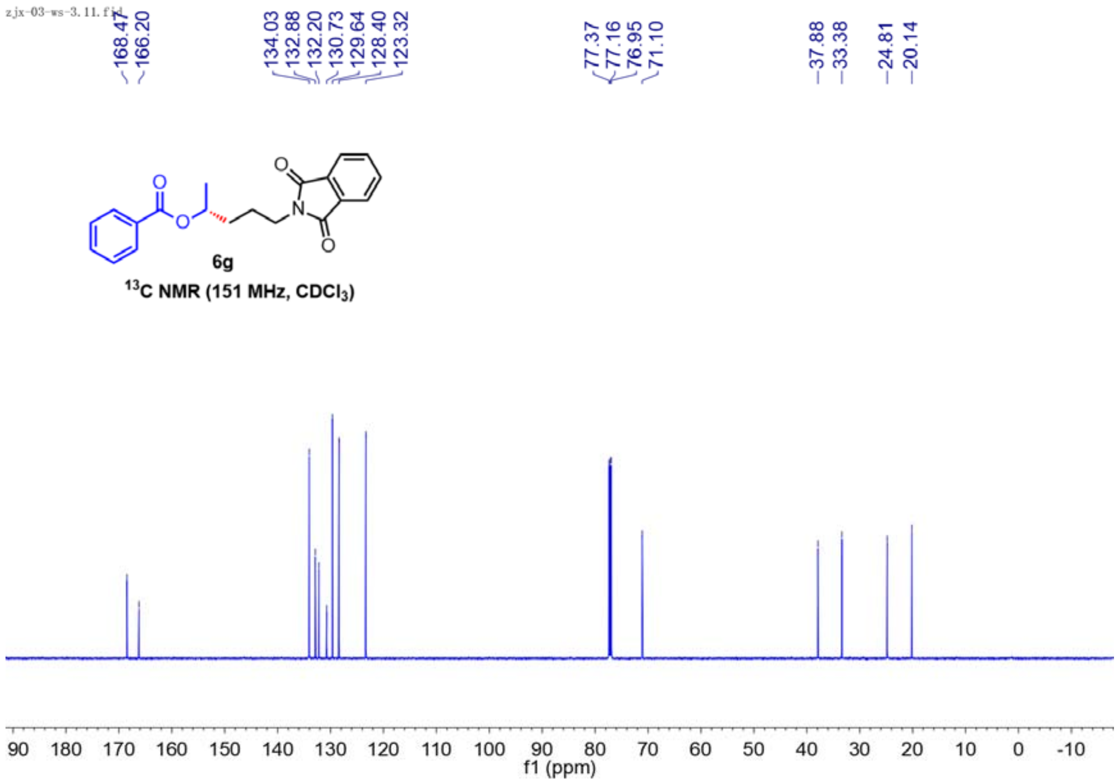

Supplementary Figure 138 ^{13}C NMR (151 MHz, $CDCl_3$) of **6g**

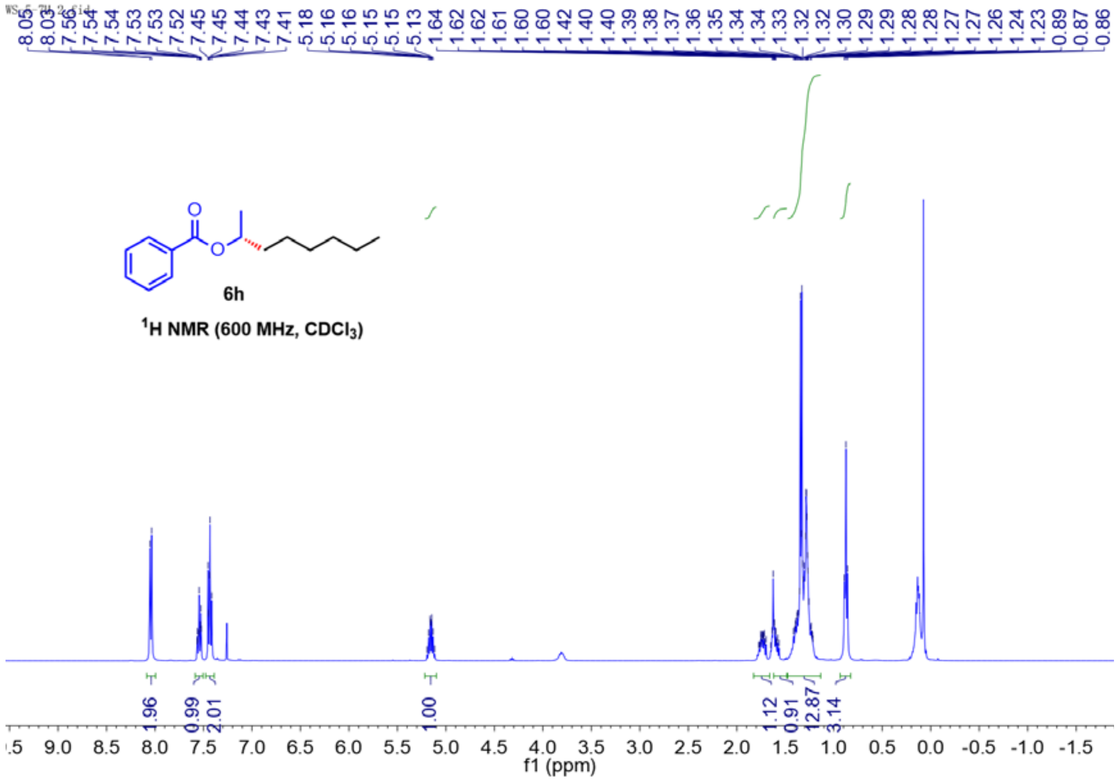

Supplementary Figure 139 ^{1}H NMR (600 MHz, $CDCl_3$) of **6h**

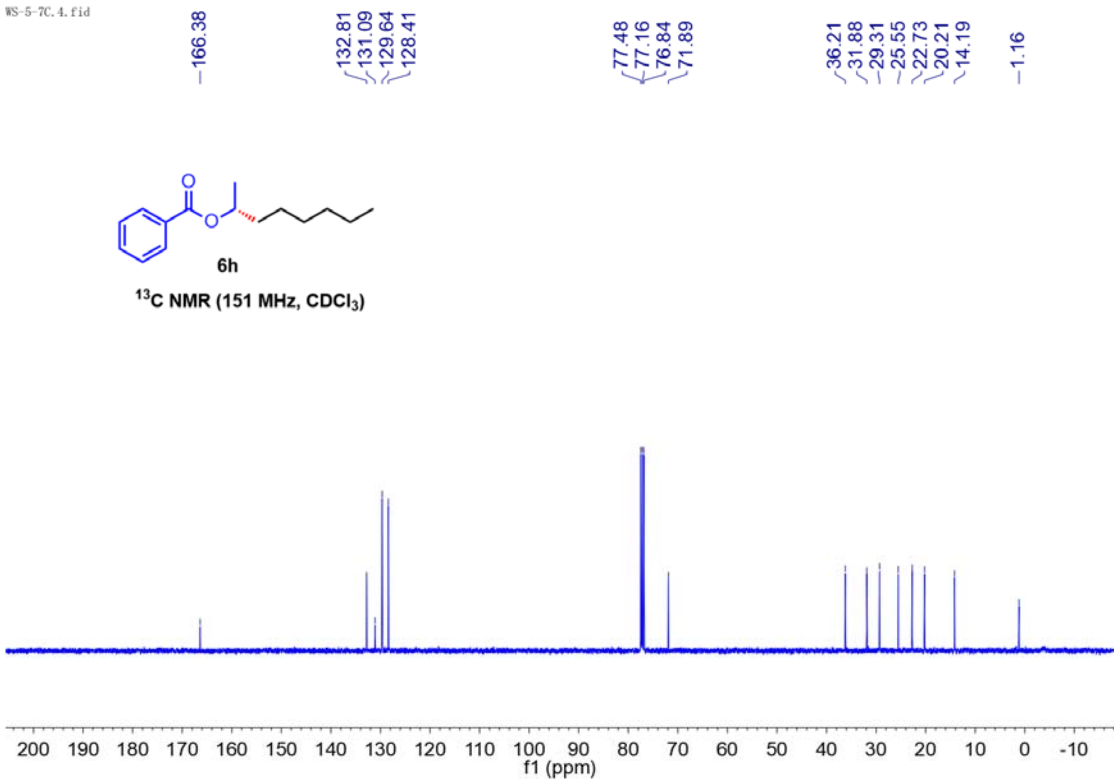

Supplementary Figure 140 ^{13}C NMR (151 MHz, $CDCl_3$) of **6h**

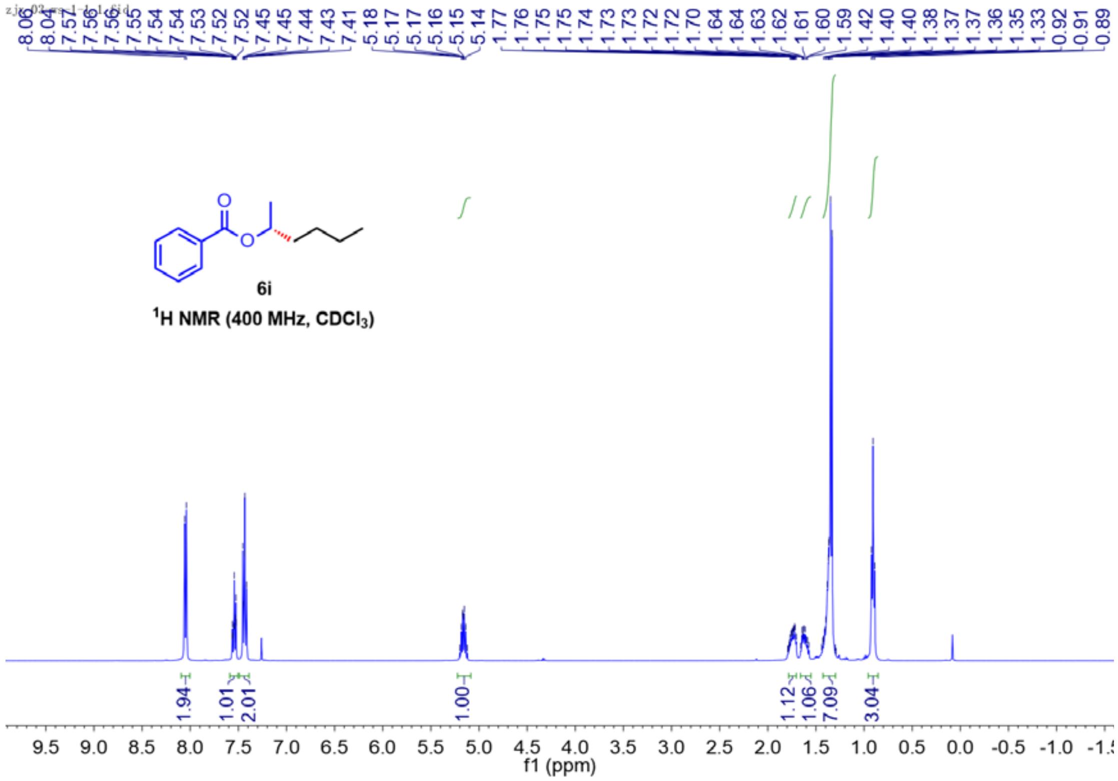

Supplementary Figure 141 ^{1}H NMR (400 MHz, $CDCl_3$) of **6i**

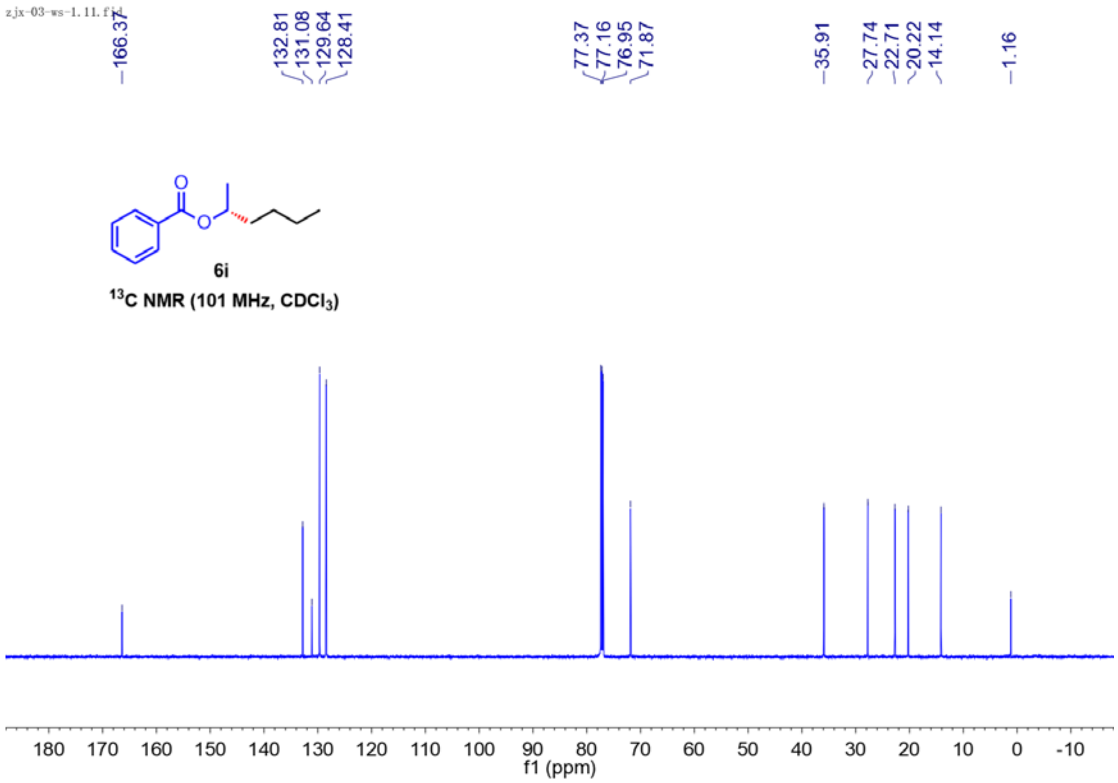

Supplementary Figure 142 ^{13}C NMR (101 MHz, $CDCl_3$) of **6i**

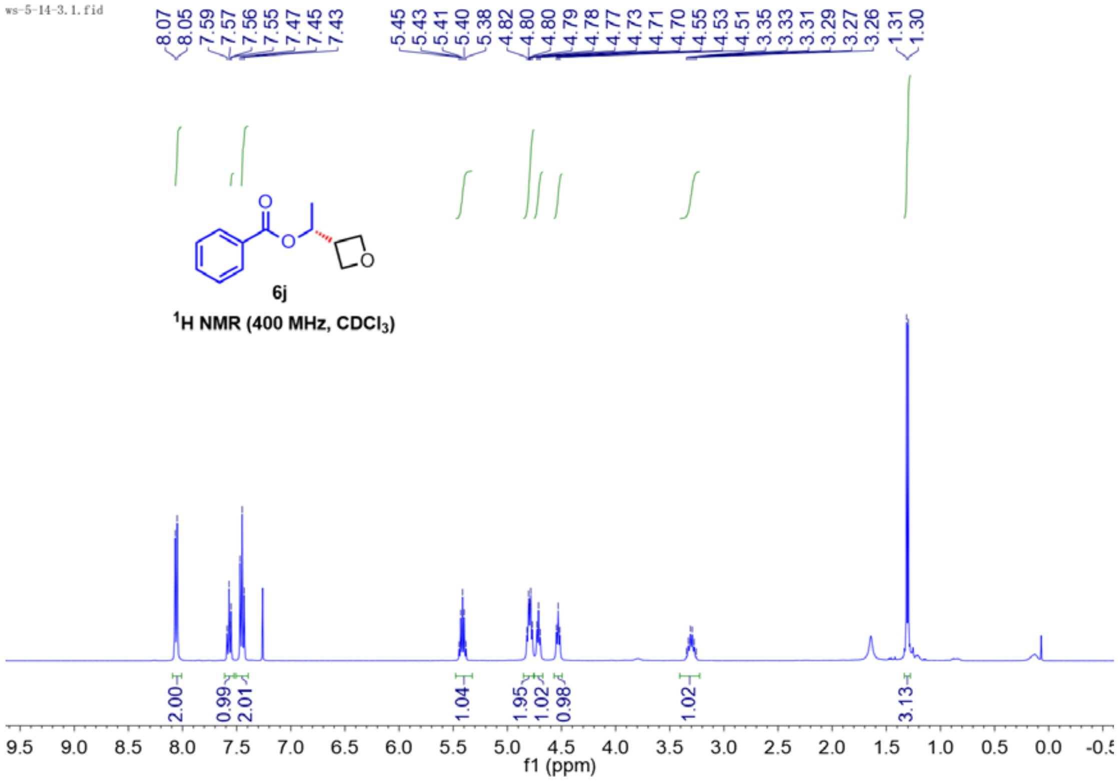

Supplementary Figure 143 ^{1}H NMR (400 MHz, $CDCl_3$) of **6j**

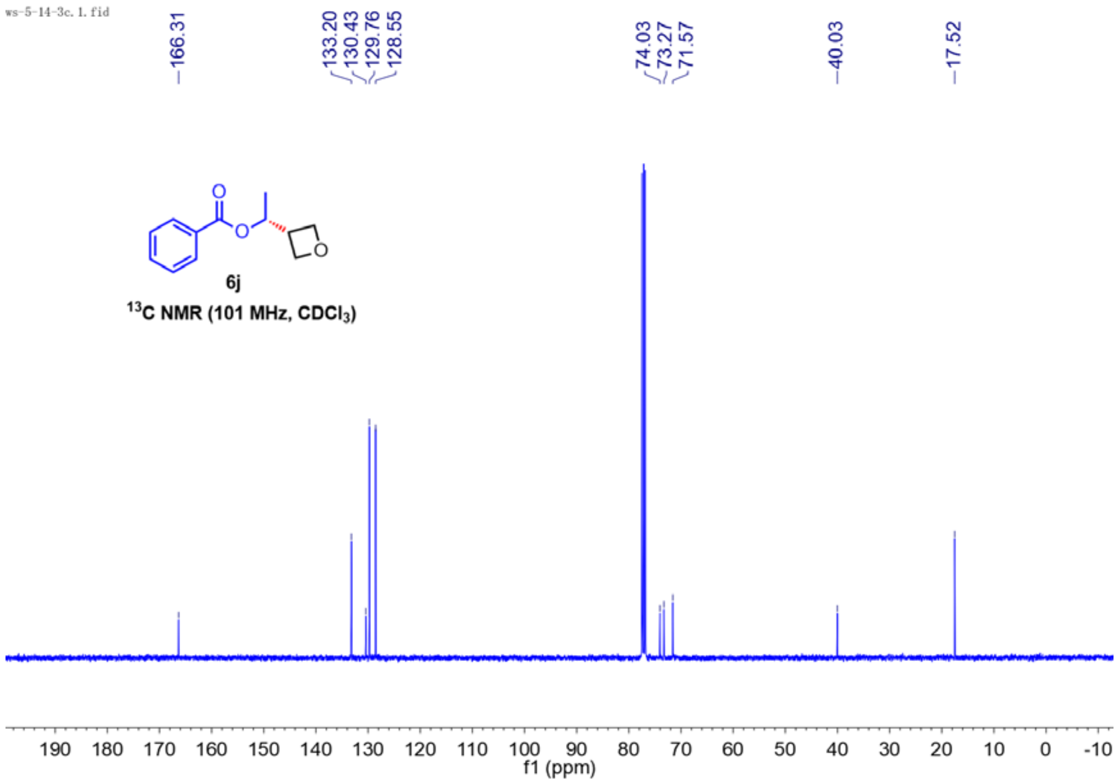

Supplementary Figure 144 ^{13}C NMR (101 MHz, CDCl$_3$) of **6j**

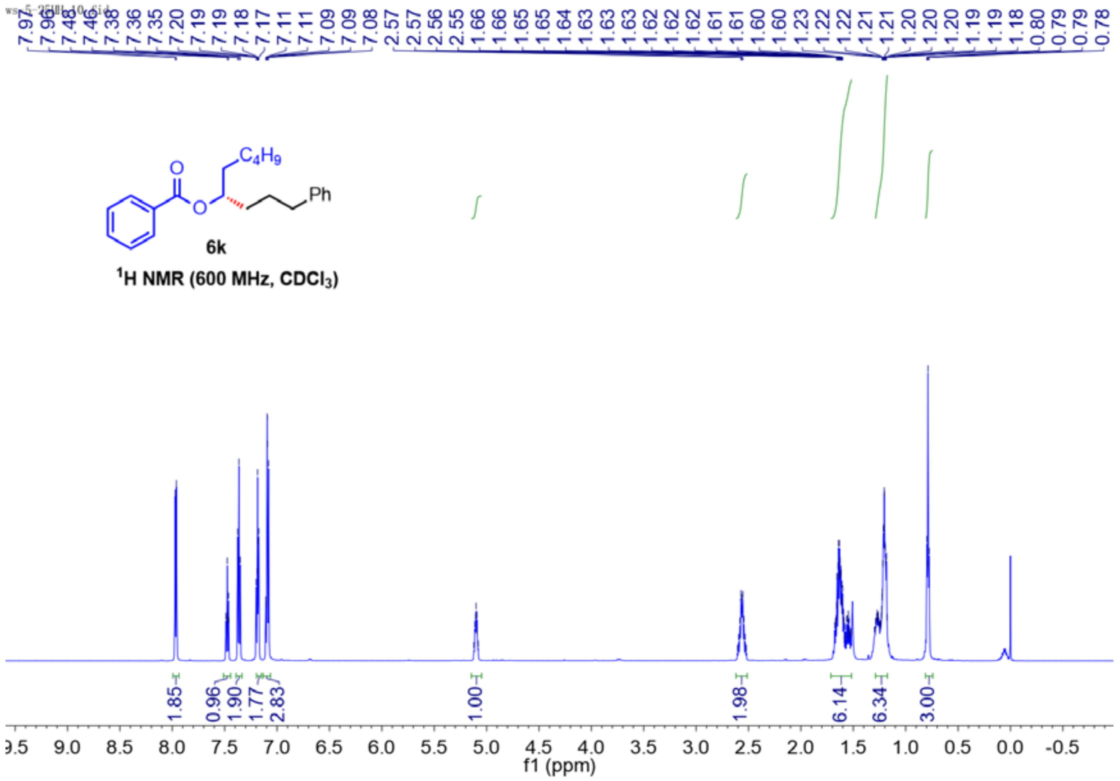

Supplementary Figure 145 ^{1}H NMR (600 MHz, CDCl$_3$) of **6k**

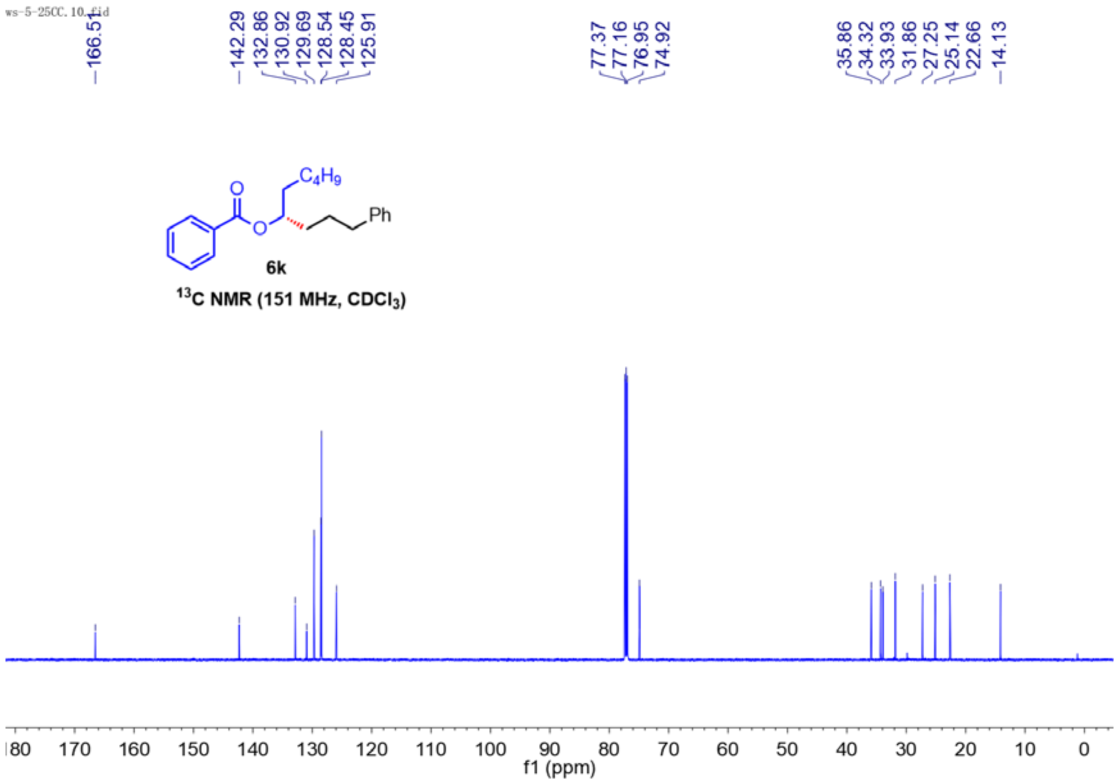

Supplementary Figure 146 ^{13}C NMR (151 MHz, $CDCl_3$) of **6k**

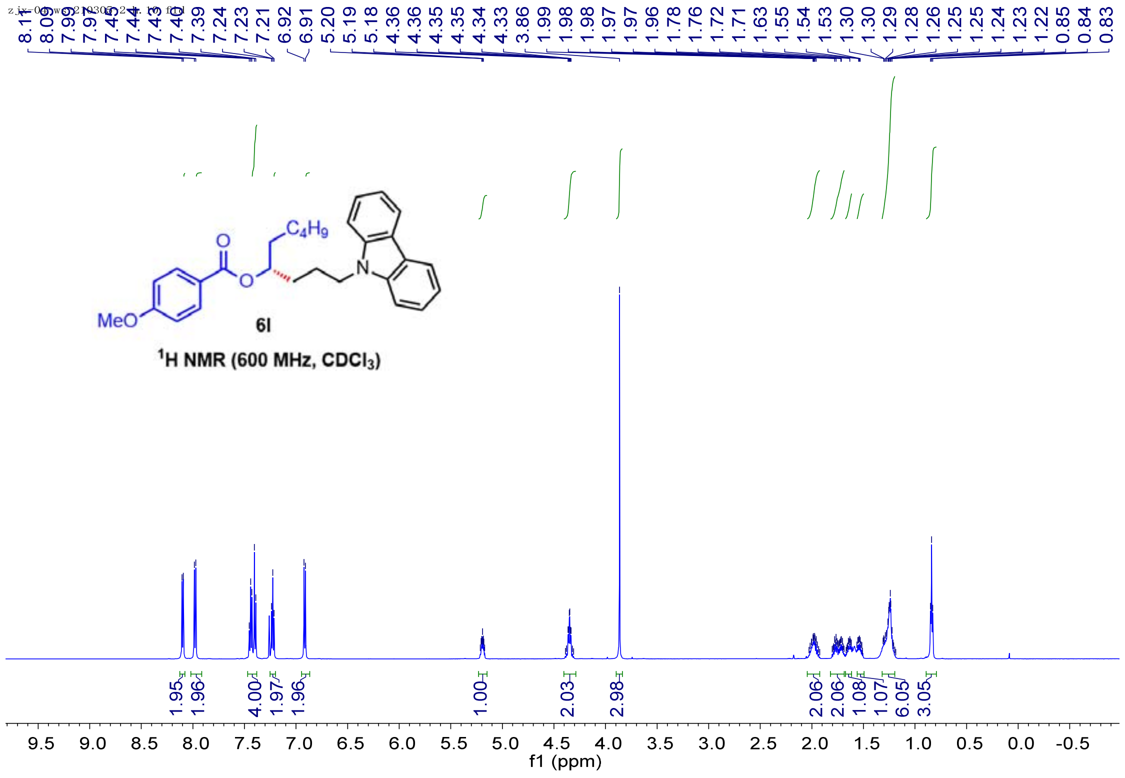

Supplementary Figure 147 ^{1}H NMR (600 MHz, $CDCl_3$) of **6l**

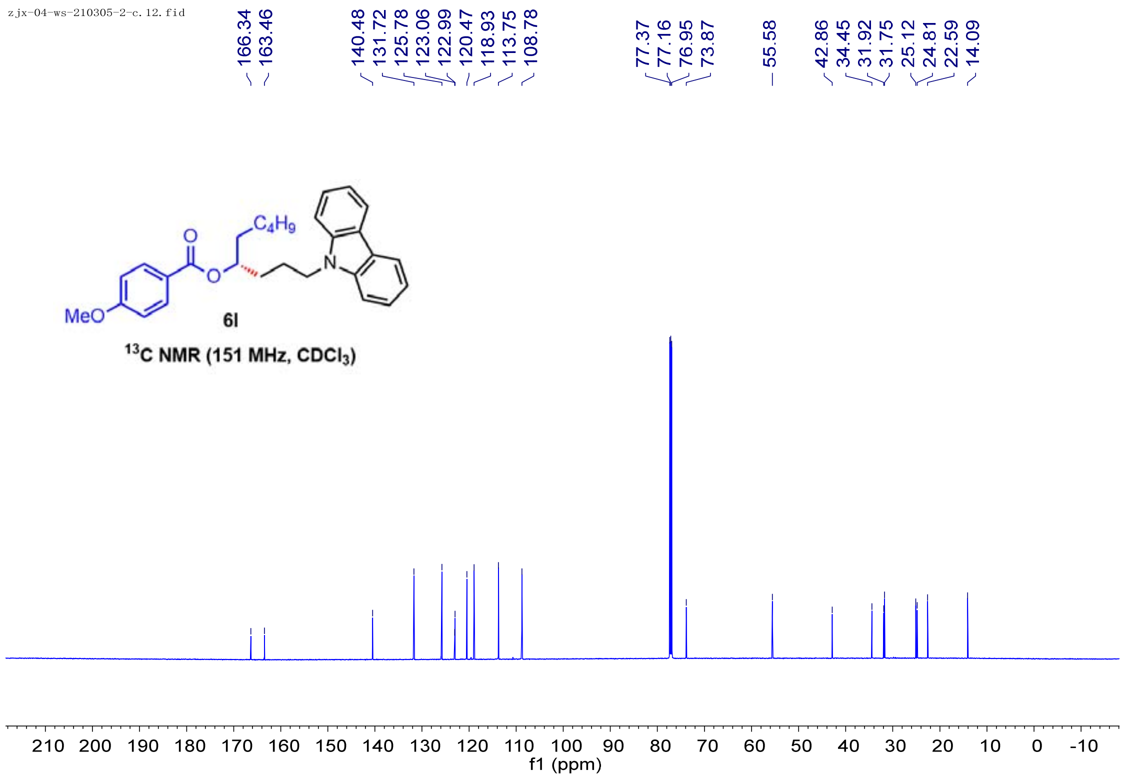

Supplementary Figure 148 ^{13}C NMR (151 MHz, $CDCl_3$) of **6l**

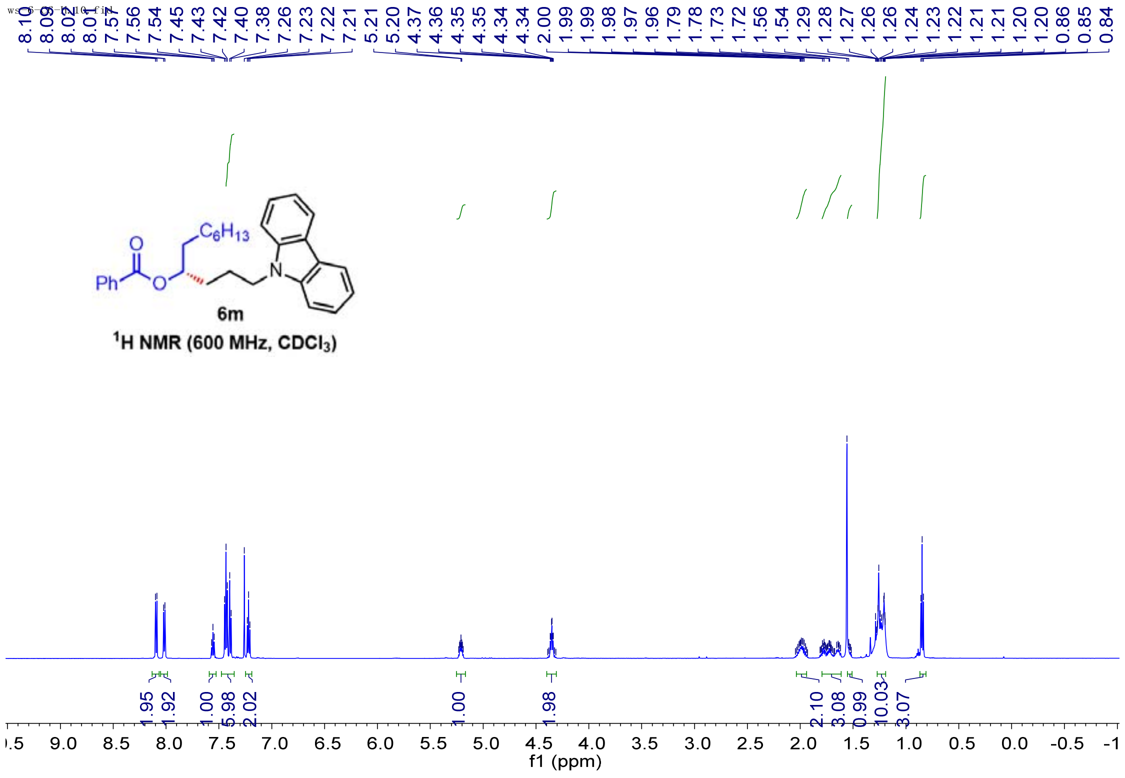

Supplementary Figure 149 ^{1}H NMR (600 MHz, $CDCl_3$) of **6m**

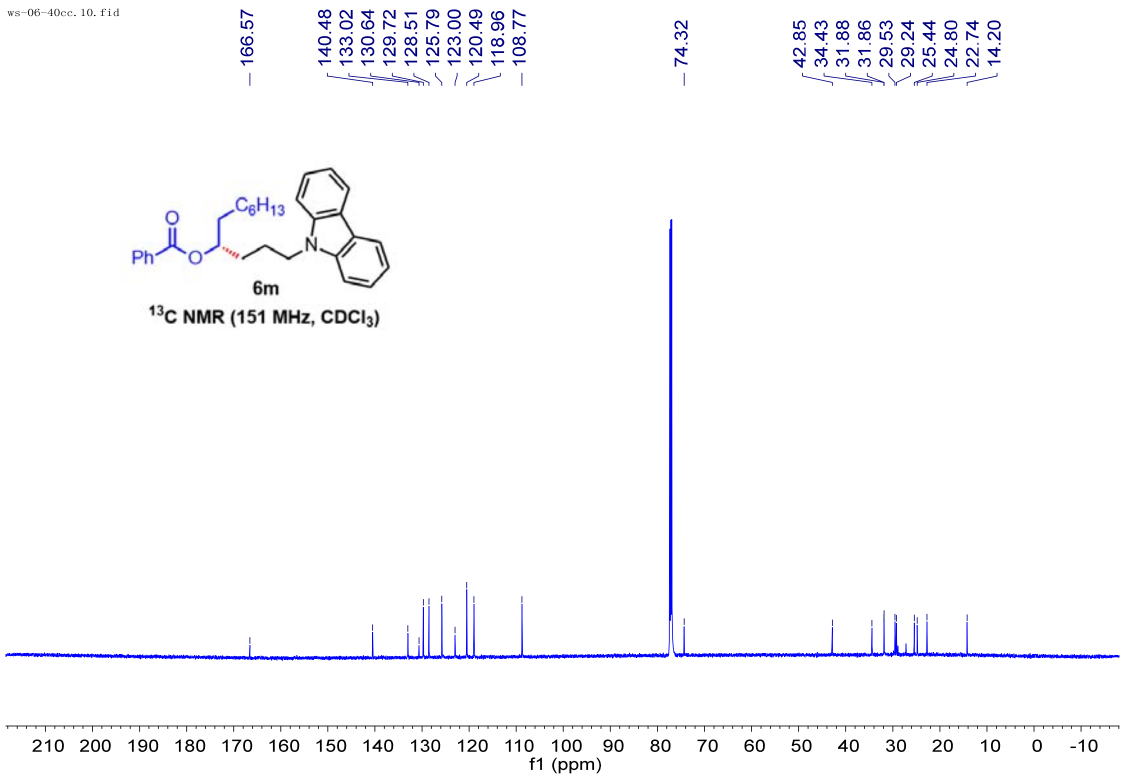

Supplementary Figure 150 ^{13}C NMR (151 MHz, $CDCl_3$) of **6m**

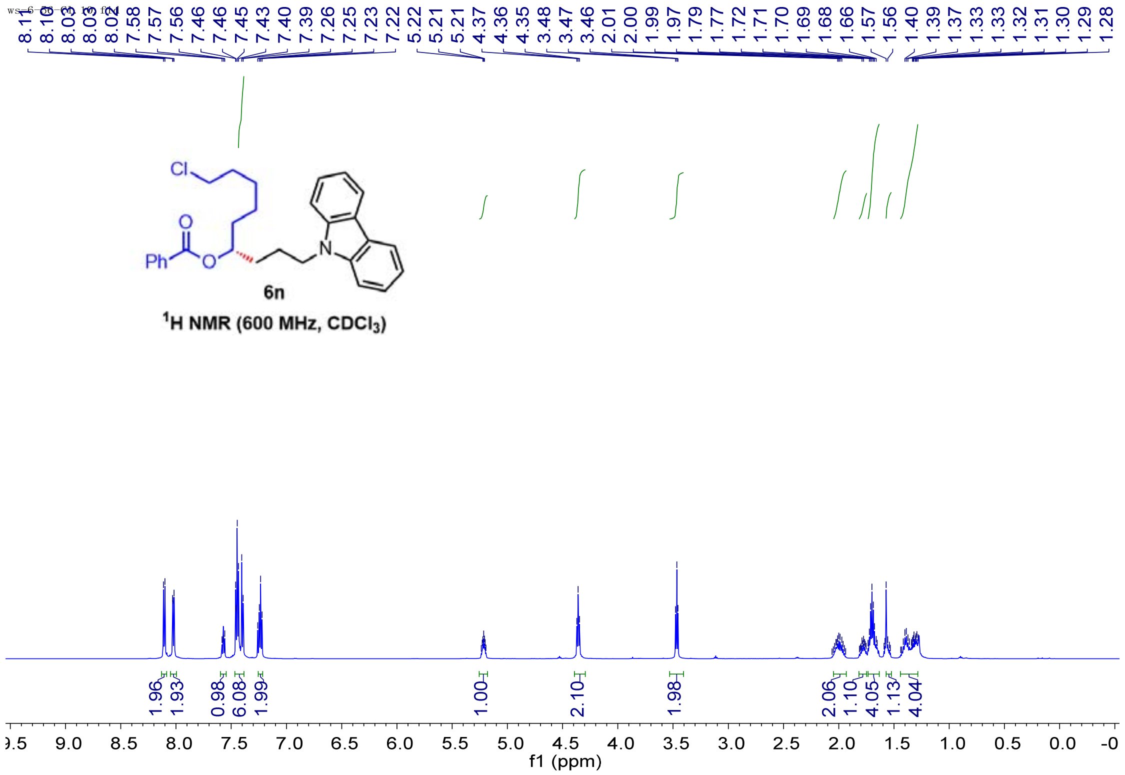

Supplementary Figure 151 ^{1}H NMR (600 MHz, $CDCl_3$) of **6n**

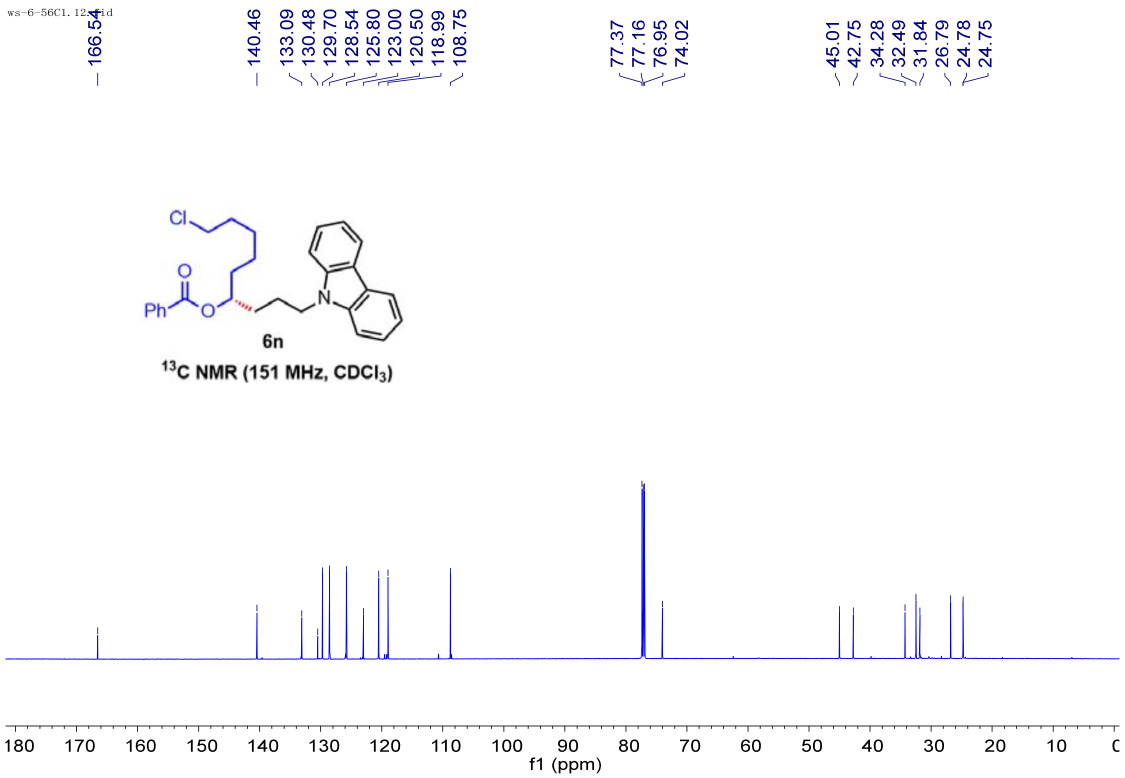

Supplementary Figure 152 ^{13}C NMR (151 MHz, $CDCl_3$) of **6n**

HPLC Traces of Products

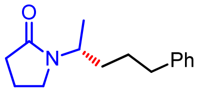

3a

HPLC data using *rac*-**L4**

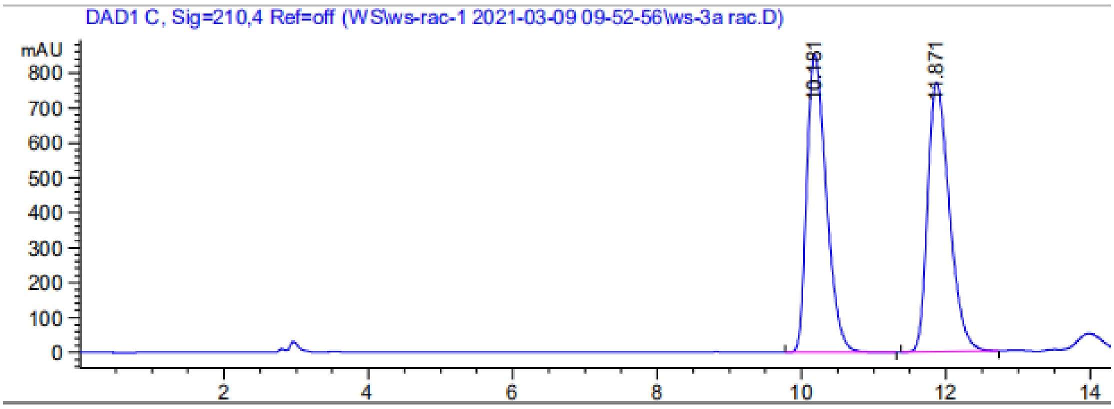

Signal 2: DAD1 C, Sig=210,4 Ref=off

Peak #	RetTime [min]	Type	Width [min]	Area [mAU*s]	Height [mAU]	Area %
1	10.181	BB	0.2915	1.60515e4	853.13147	49.9148
2	11.871	BB	0.3217	1.61063e4	770.97754	50.0852

HPLC data using (1*S*, 2*R*)-**L9**

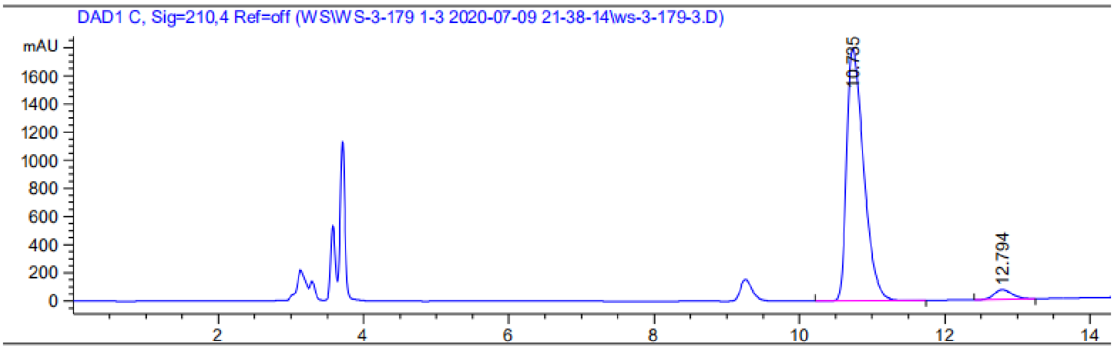

Signal 2: DAD1 C, Sig=210,4 Ref=off

Peak #	RetTime [min]	Type	Width [min]	Area [mAU*s]	Height [mAU]	Area %
1	10.735	BV R	0.2522	2.92310e4	1791.93030	96.0068
2	12.794	BV	0.2652	1215.81750	69.79015	3.9932

Supplementary Figure 153 HPLC spectra for **3a**

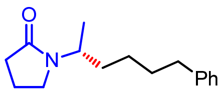

3b

HPLC data using *rac*-**L4**

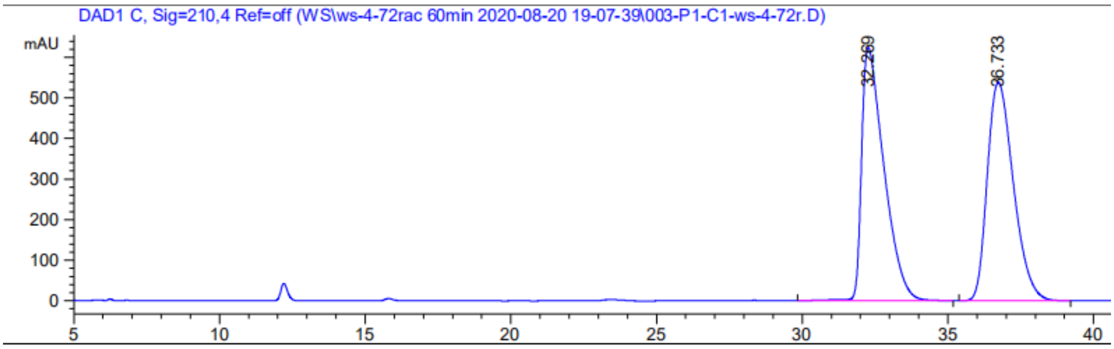

Signal 2: DAD1 C, Sig=210,4 Ref=off

Peak #	RetTime [min]	Type	Width [min]	Area [mAU*s]	Height [mAU]	Area %
1	32.269	BB	0.7864	3.33052e4	624.32355	49.8113
2	36.733	BB	0.9661	3.35576e4	537.71338	50.1887

HPLC data using (1*S*, 2*R*)-**L9**

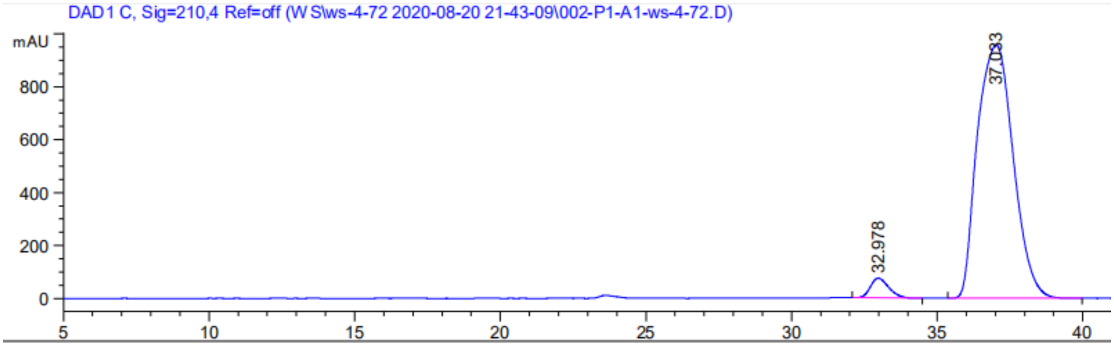

Signal 1: DAD1 B, Sig=254,4 Ref=off

Peak #	RetTime [min]	Type	Width [min]	Area [mAU*s]	Height [mAU]	Area %
1	3.435	BB	0.0627	7.51476	1.87888	2.3658
2	37.553	BB	0.7170	310.13284	5.29791	97.6342

Supplementary Figure 154 HPLC spectra for **3b**

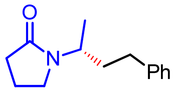

3c

HPLC data using *rac*-**L4**

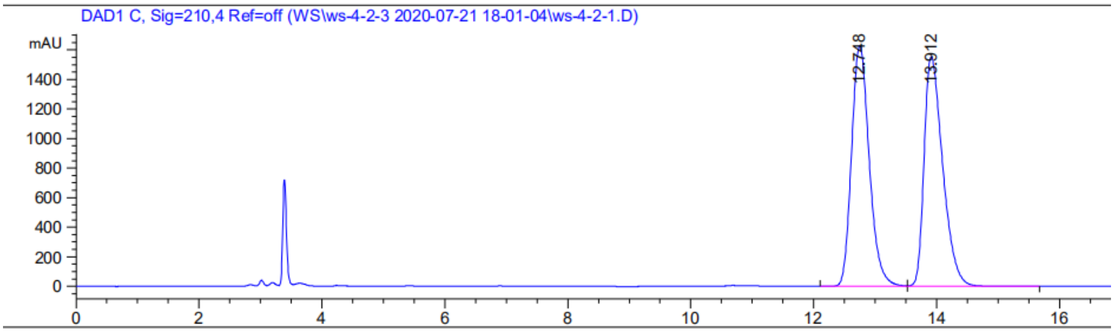

Signal 1: DAD1 B, Sig=254,4 Ref=off

Peak #	RetTime [min]	Type	Width [min]	Area [mAU*s]	Height [mAU]	Area %
1	12.749	BB	0.2986	507.56091	26.13306	49.9328
2	13.912	BB	0.3120	508.92667	24.73615	50.0672

HPLC data using (1*S*, 2*R*)-**L9**

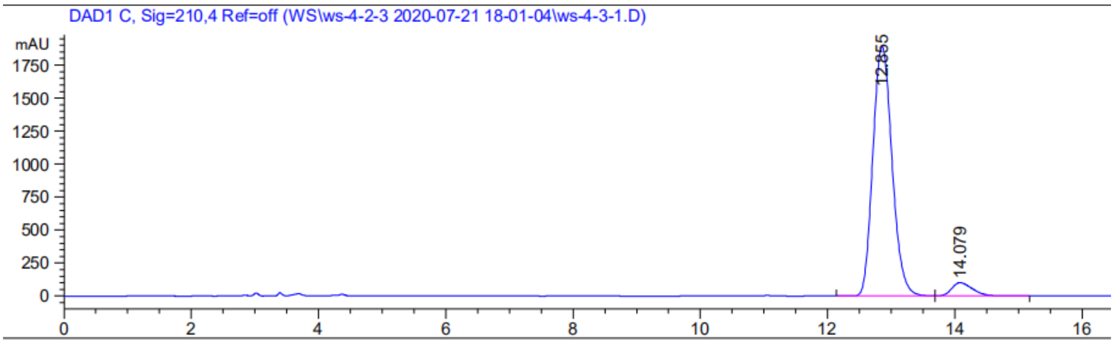

Peak #	RetTime [min]	Type	Width [min]	Area [mAU*s]	Height [mAU]	Area %
1	11.957	BB	0.3550	1246.30408	57.01432	92.6691
2	13.091	BB	0.4019	98.59352	3.91394	7.3309

Supplementary Figure 155 HPLC spectra for **3c**

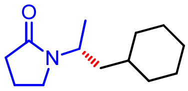

3d

HPLC data using *rac*-**L4**

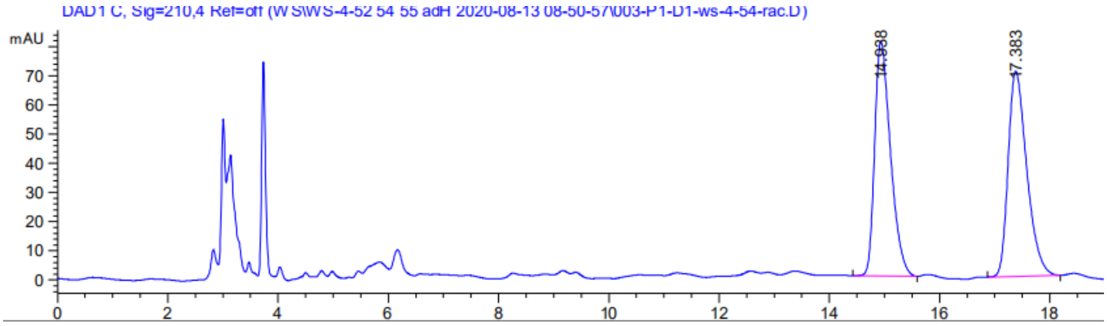

Signal 2: DAD1 C, Sig=210,4 Ref=off

Peak #	RetTime [min]	Type	Width [min]	Area [mAU*s]	Height [mAU]	Area %
1	17.401	BB	0.3701	5299.53467	217.63385	50.1804
2	18.928	BB	0.4373	5261.42676	179.85439	49.8196

HPLC data using (1*S*, 2*R*)-**L9**

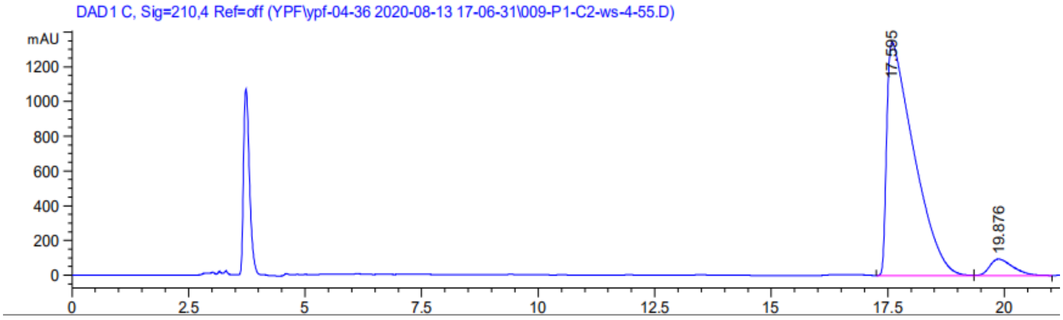

Signal 2: DAD1 C, Sig=210,4 Ref=off

Peak #	RetTime [min]	Type	Width [min]	Area [mAU*s]	Height [mAU]	Area %
1	17.595	BV	0.5749	5.42730e4	1344.53064	94.3780
2	19.876	VB	0.5237	3232.95947	94.36605	5.6220

Supplementary Figure 156 HPLC spectra for **3d**

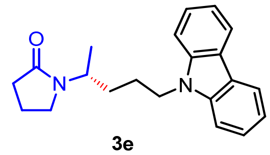

3e

HPLC data using *rac*-**L4**

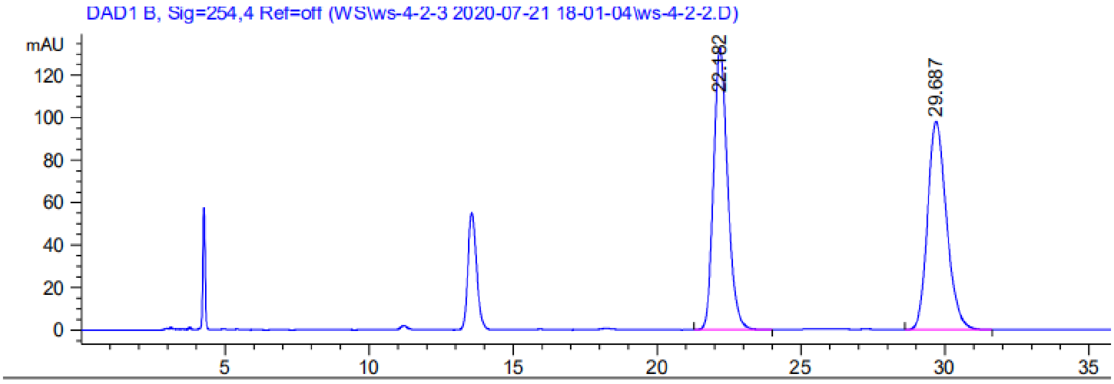

Signal 2: DAD1 C, Sig=210,4 Ref=off

Peak #	RetTime [min]	Type	Width [min]	Area [mAU*s]	Height [mAU]	Area %
1	22.182	BB	0.5188	8136.84814	240.46016	50.0164
2	29.687	BB	0.7026	8131.51758	177.44228	49.9836

HPLC data using (1*S*, 2*R*)-**L9**

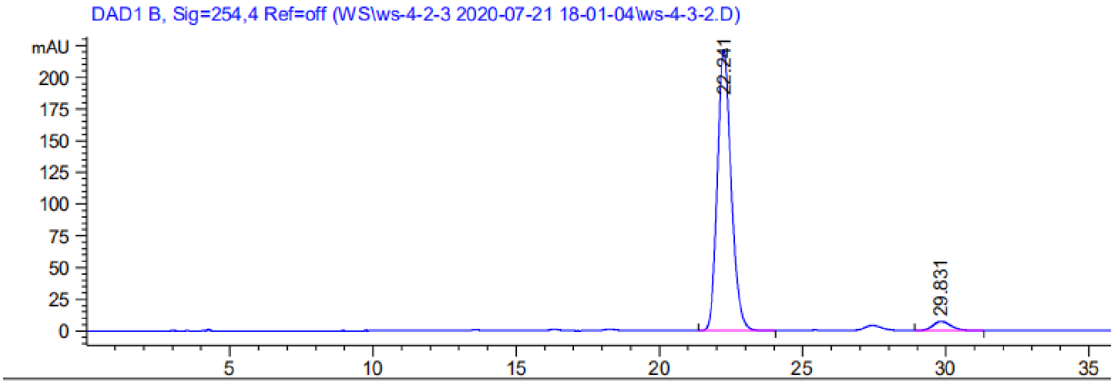

Signal 2: DAD1 C, Sig=210,4 Ref=off

Peak #	RetTime [min]	Type	Width [min]	Area [mAU*s]	Height [mAU]	Area %
1	20.903	BB	0.4848	2.21093e4	699.34106	95.3820
2	28.638	BB	0.6712	1070.43750	24.33394	4.6180

Supplementary Figure 157 HPLC spectra for **3e**

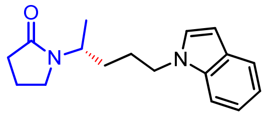

3f

HPLC data using *rac*-**L4**

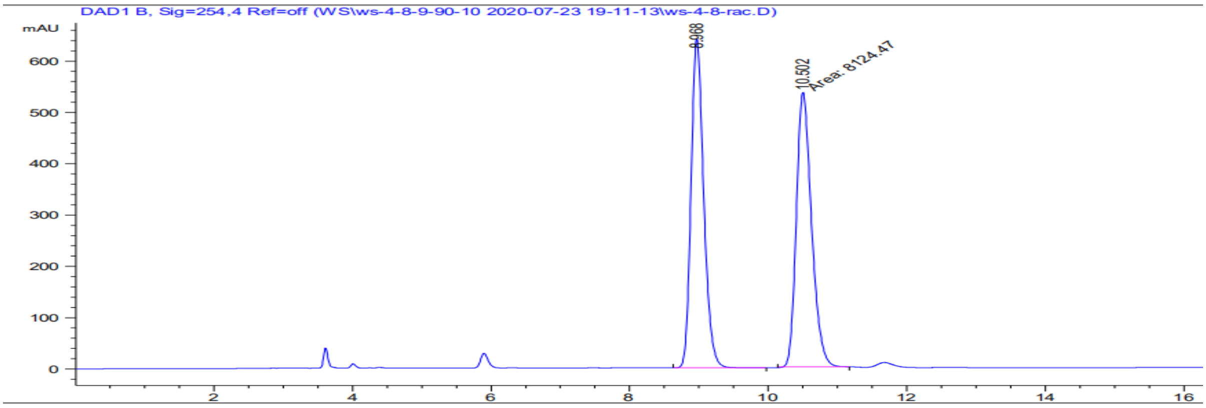

Signal 1: DAD1 B, Sig=254,4 Ref=off

Peak #	RetTime [min]	Type	Width [min]	Area [mAU*s]	Height [mAU]	Area %
1	8.968	BB	0.1961	8132.77637	640.73199	50.0256
2	10.502	MM	0.2532	8124.46875	534.82269	49.9744

HPLC data using (1*S*, 2*R*)-**L9**

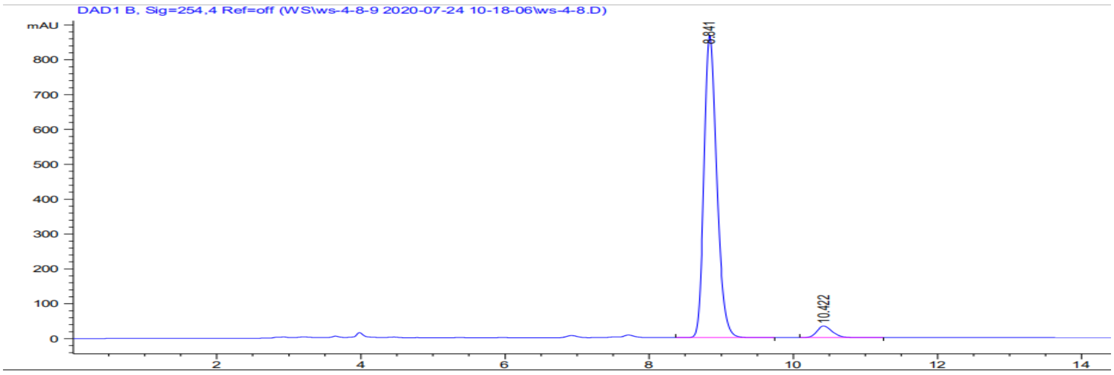

Signal 1: DAD1 B, Sig=254,4 Ref=off

Peak #	RetTime [min]	Type	Width [min]	Area [mAU*s]	Height [mAU]	Area %
1	8.841	BB	0.1892	1.06348e4	866.22150	95.6073
2	10.422	BB	0.2261	488.61963	33.11900	4.3927

Supplementary Figure 158 HPLC spectra for **3f**

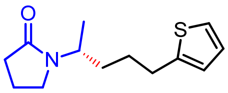

3g

HPLC data using *rac*-**L4**

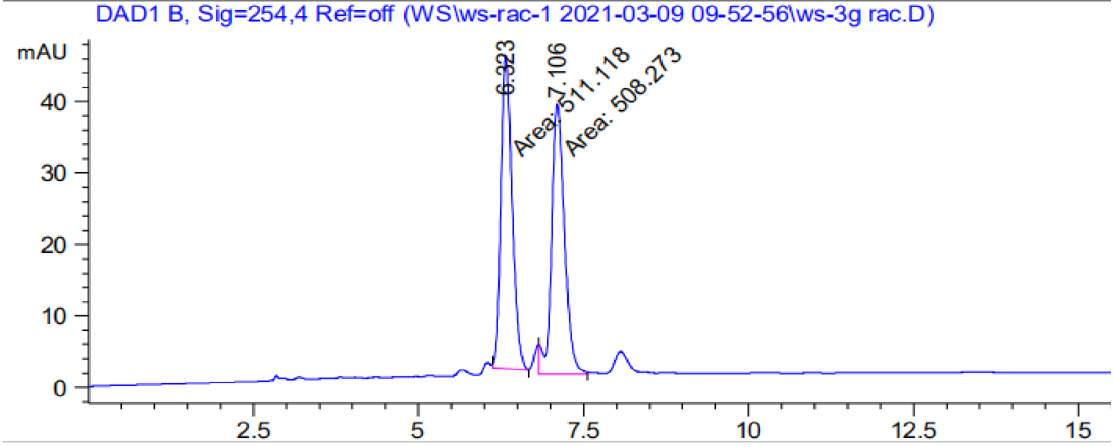

Signal 2: DAD1 C, Sig=210,4 Ref=off

Peak #	RetTime [min]	Type	Width [min]	Area [mAU*s]	Height [mAU]	Area %
1	6.594	MM	0.1440	3793.81787	439.05496	48.0982
2	7.603	MM	0.1829	4093.83423	373.13739	51.9018

HPLC data using (1*S*, 2*R*)-**L9**

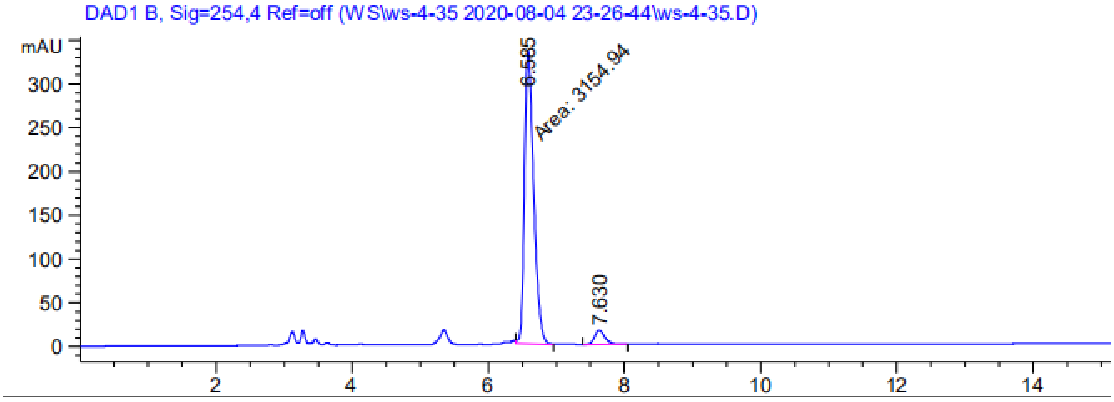

Signal 2: DAD1 C, Sig=210,4 Ref=off

Peak #	RetTime [min]	Type	Width [min]	Area [mAU*s]	Height [mAU]	Area %
1	6.585	VB R	0.1616	2.64378e4	2548.04736	95.0029
2	7.630	BB	0.1591	1390.60254	133.93008	4.9971

Supplementary Figure 159 HPLC spectra for **3g**

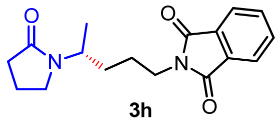

3h

HPLC data using *rac*-**L4**

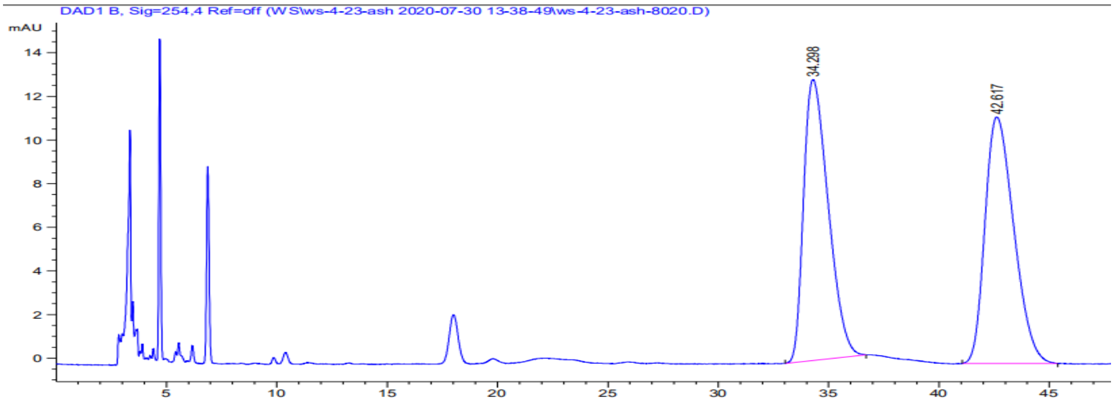

Signal 1: DAD1 B, Sig=254,4 Ref=off

Peak #	RetTime [min]	Type	Width [min]	Area [mAU*s]	Height [mAU]	Area %
1	34.298	BB	1.0424	997.88007	12.87244	49.3004
2	42.617	BB	1.1177	1026.20166	11.29763	50.6996

HPLC data using (1*S*, 2*R*)-**L9**

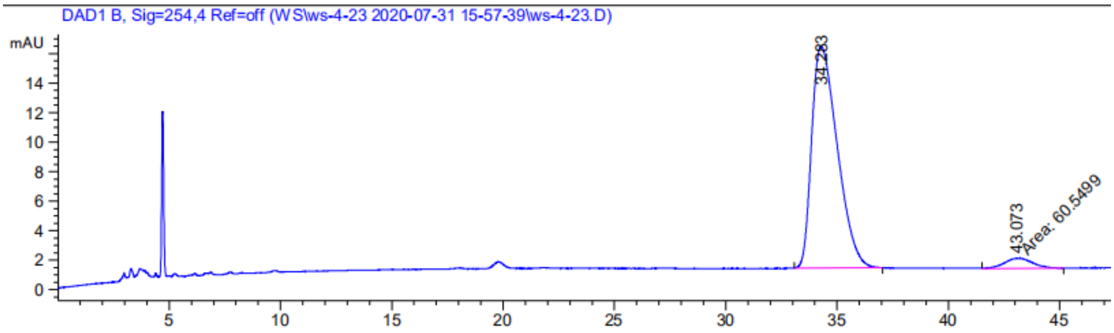

Signal 1: DAD1 B, Sig=254,4 Ref=off

Peak #	RetTime [min]	Type	Width [min]	Area [mAU*s]	Height [mAU]	Area %
1	34.283	BB	1.0939	1203.47705	15.03506	95.2098
2	43.073	MM	1.4590	60.54986	6.91697e-1	4.7902

Supplementary Figure 160 HPLC spectra for **3h**

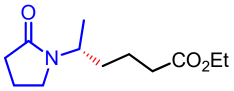

3i

HPLC data using *rac*-**L4**

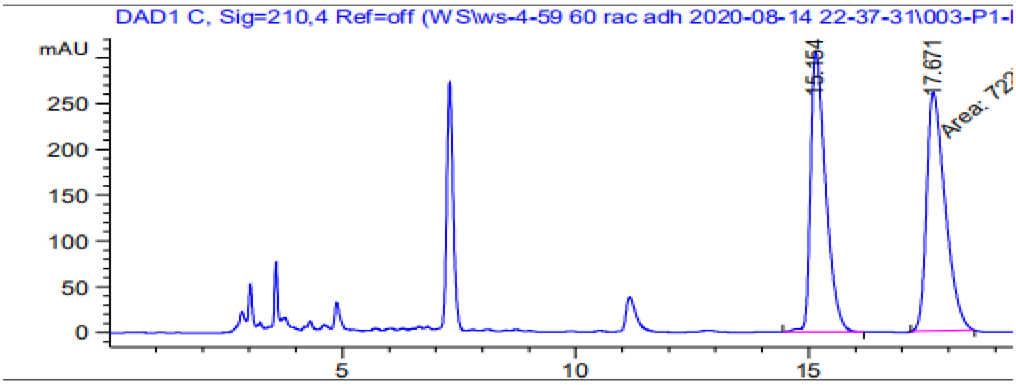

Signal 2: DAD1 C, Sig=210,4 Ref=off

Peak #	RetTime [min]	Type	Width [min]	Area [mAU*s]	Height [mAU]	Area %
1	15.154	BB	0.3391	6893.46240	305.62512	48.8157
2	17.671	MM	0.4627	7227.93359	260.33777	51.1843

HPLC data using (1*S*, 2*R*)-**L9**

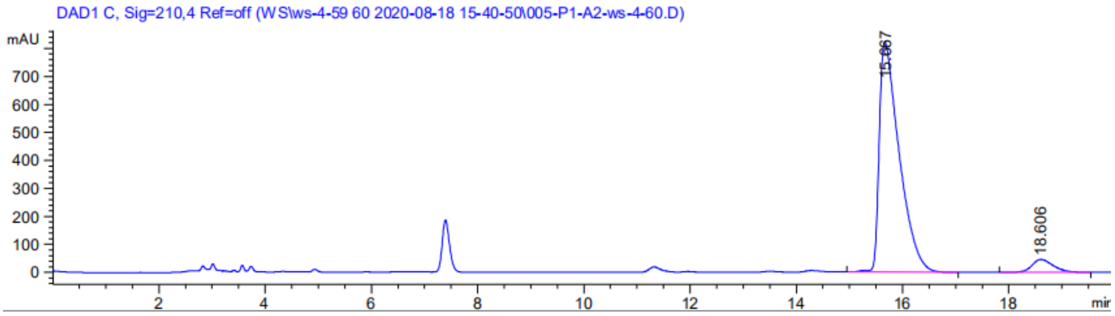

Signal 2: DAD1 C, Sig=210,4 Ref=off

Peak #	RetTime [min]	Type	Width [min]	Area [mAU*s]	Height [mAU]	Area %
1	15.667	VB R	0.3827	2.14226e4	822.37012	94.3185
2	18.606	BB	0.4217	1290.44385	46.22613	5.6815

Supplementary Figure 161 HPLC spectra for **3i**

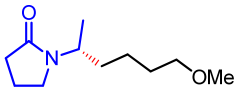

3j

HPLC data using *rac*-**L4**

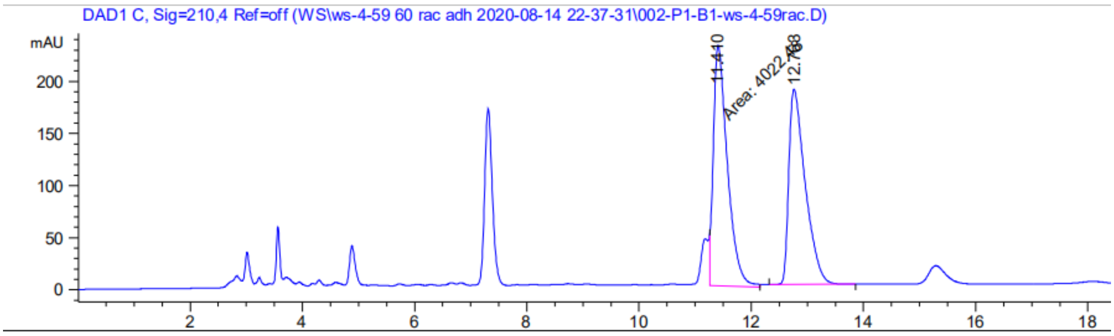

Signal 2: DAD1 C, Sig=210,4 Ref=off

Peak #	RetTime [min]	Type	Width [min]	Area [mAU*s]	Height [mAU]	Area %
1	11.410	MM	0.2903	4022.47656	230.91672	51.4002
2	12.763	BB	0.3028	3803.31714	187.38770	48.5998

H

PLC data using (1*S*, 2*R*)-**L9**

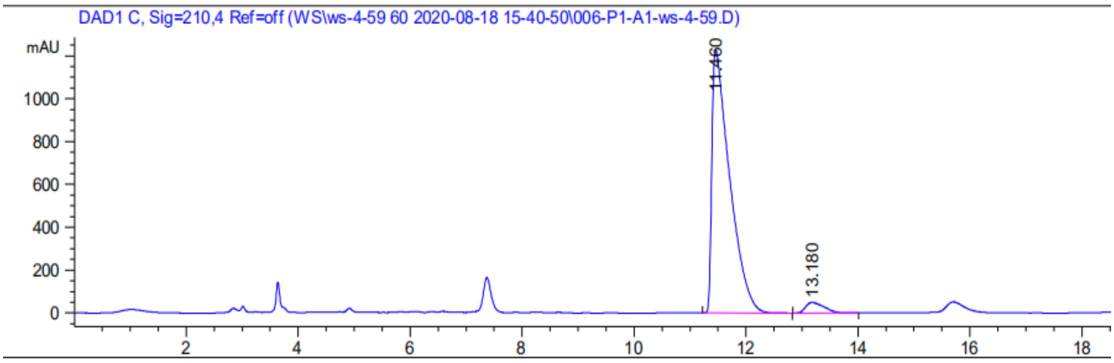

Signal 2: DAD1 C, Sig=210,4 Ref=off

Peak #	RetTime [min]	Type	Width [min]	Area [mAU*s]	Height [mAU]	Area %
1	11.460	BB	0.3150	2.70142e4	1226.13037	95.9654
2	13.180	BB	0.3447	1135.72363	49.67984	4.0346

Supplementary Figure 162 HPLC spectra for **3j**

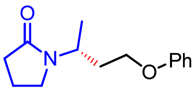

3k

HPLC data using *rac*-**L4**

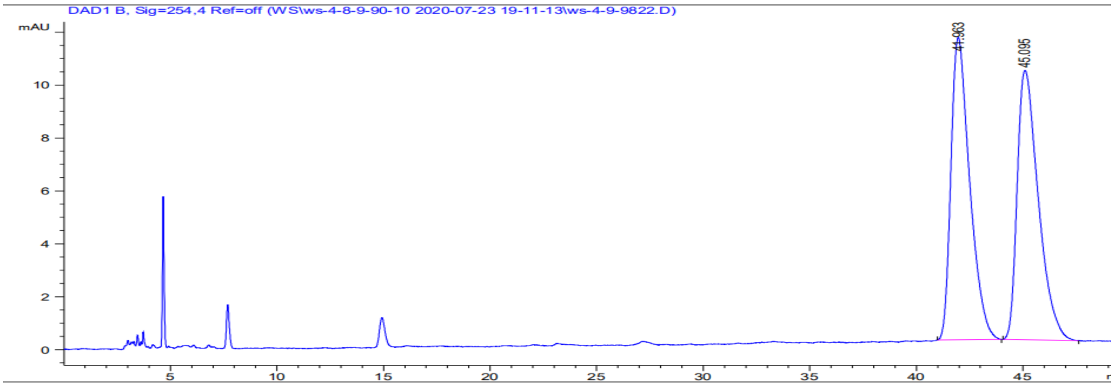

Signal 1: DAD1 B, Sig=254,4 Ref=off

Peak #	RetTime [min]	Type	Width [min]	Area [mAU*s]	Height [mAU]	Area %
1	41.963	BB	0.8573	699.07367	11.43473	49.9441
2	45.095	BB	0.9267	700.63898	10.18375	50.0559

HPLC data using (1*S*, 2*R*)-**L9**

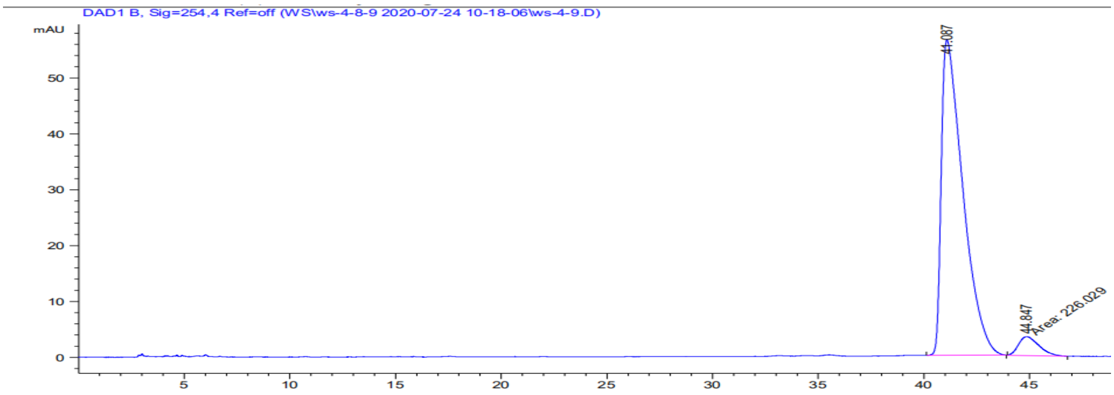

Signal 1: DAD1 B, Sig=254,4 Ref=off

Peak #	RetTime [min]	Type	Width [min]	Area [mAU*s]	Height [mAU]	Area %
1	41.087	BB	0.9940	4004.58081	56.42204	94.6573
2	44.847	MM	1.1127	226.02878	3.38562	5.3427

Supplementary Figure 163 HPLC spectra for **3k**

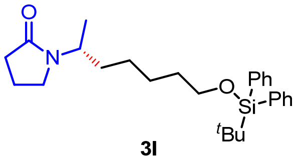

3l

HPLC data using *rac*-**L4**

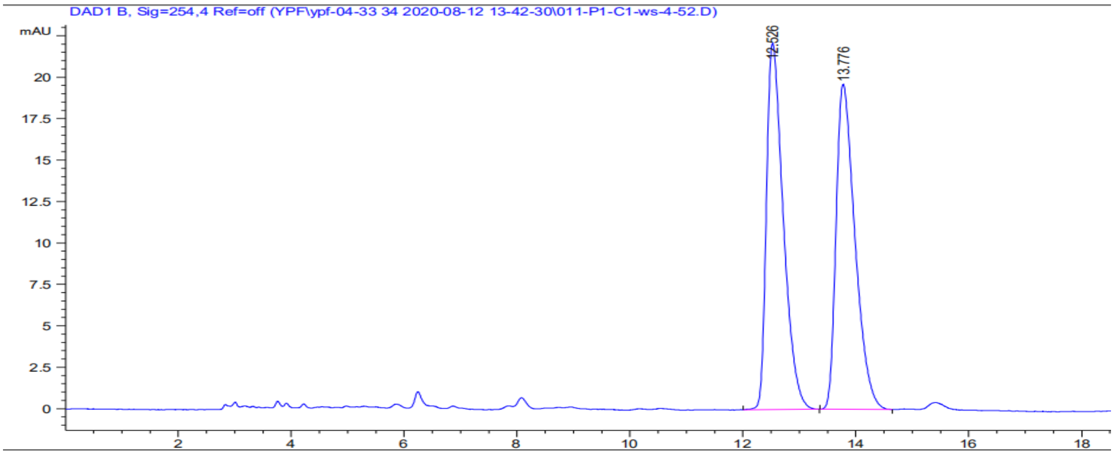

Signal 1: DAD1 B, Sig=254,4 Ref=off

Peak #	RetTime [min]	Type	Width [min]	Area [mAU*s]	Height [mAU]	Area %
1	12.526	BB	0.3154	457.48480	22.10621	50.1026
2	13.776	BB	0.3490	455.61026	19.61252	49.8974

HPLC data using (1*S*, 2*R*)-**L9**

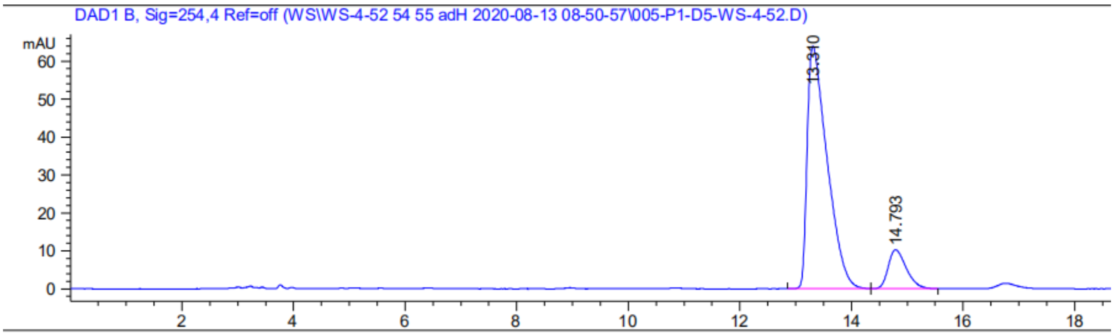

Signal 1: DAD1 B, Sig=254,4 Ref=off

Peak #	RetTime [min]	Type	Width [min]	Area [mAU*s]	Height [mAU]	Area %
1	13.310	BB	0.3669	1582.27454	63.89964	87.2944
2	14.793	BB	0.3420	230.29785	10.25558	12.7056

Supplementary Figure 164 HPLC spectra for **3l**

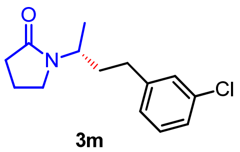

3m

HPLC data using *rac*-**L4**

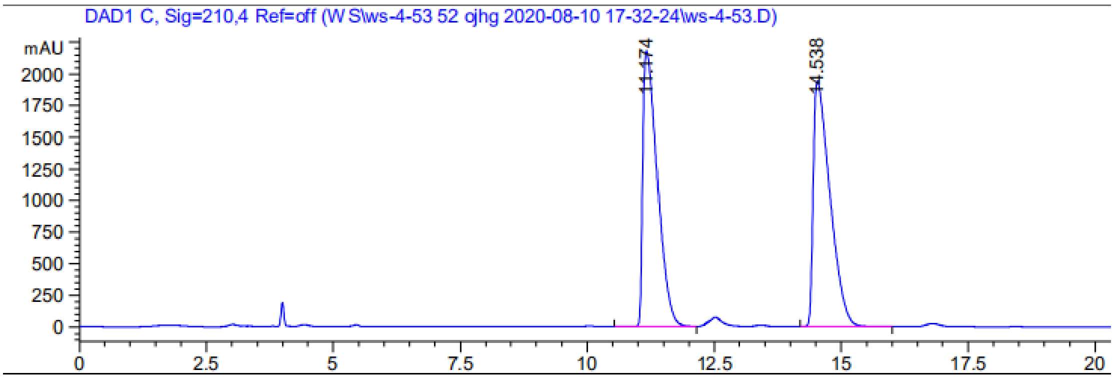

Signal 1: DAD1 B, Sig=254,4 Ref=off

Peak #	RetTime [min]	Type	Width [min]	Area [mAU*s]	Height [mAU]	Area %
1	11.174	BB	0.2736	512.31744	27.72804	49.5114
2	14.538	BB	0.3261	522.42963	23.43353	50.4886

HPLC data using (1*S*, 2*R*)-**L9**

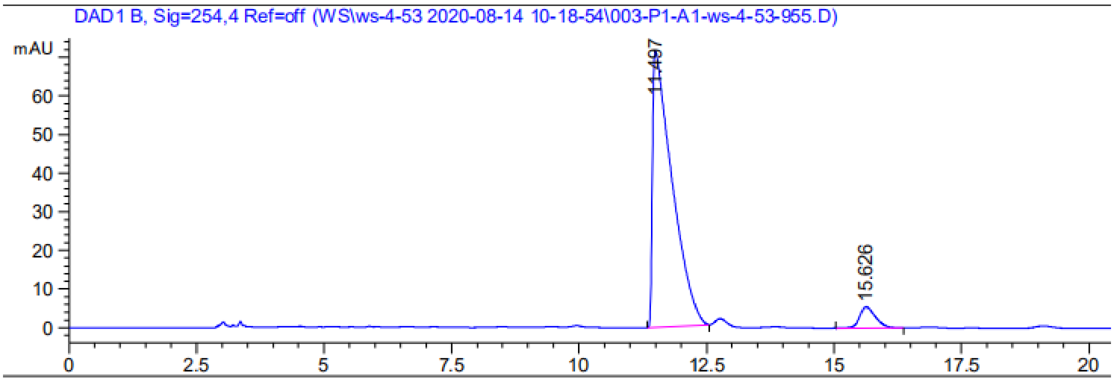

Signal 1: DAD1 B, Sig=254,4 Ref=off

Peak #	RetTime [min]	Type	Width [min]	Area [mAU*s]	Height [mAU]	Area %
1	11.497	BB	0.3733	1928.30896	71.36021	94.2463
2	15.626	BB	0.3267	117.72189	5.43494	5.7537

Supplementary Figure 165 HPLC spectra for **3m**

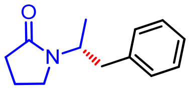

3n

HPLC data using *rac*-**L4**

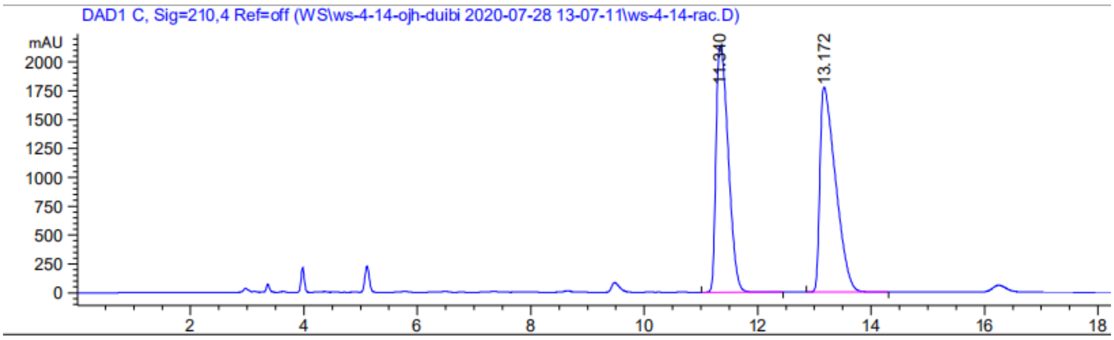


```
Signal 1: DAD1 B, Sig=254,4 Ref=off

Peak RetTime Type  Width     Area       Height      Area
  #   [min]        [min]   [mAU*s]      [mAU]        %
----|-------|----|-------|----------|----------|--------|
   1  11.341 BB    0.2030  482.74164   35.86716  50.1923
   2  13.172 BB    0.2681  479.04224   26.10583  49.8077
```

HPLC data using (1*S*, 2*R*)-**L9**

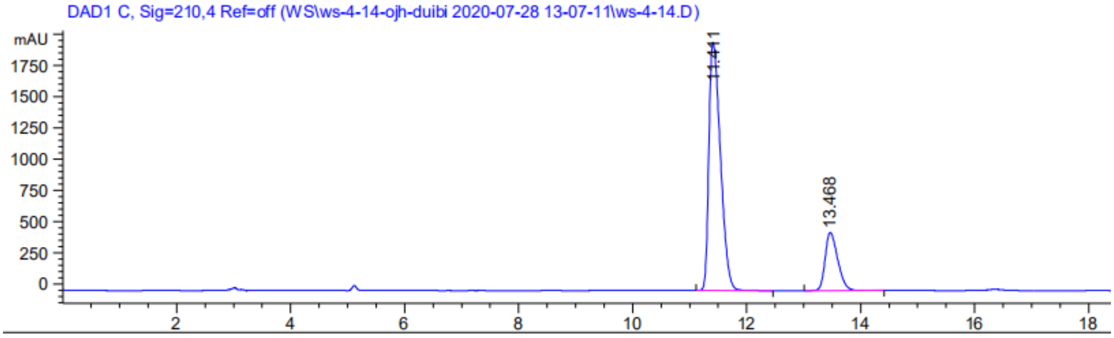


```
Signal 1: DAD1 B, Sig=254,4 Ref=off

Peak RetTime Type  Width     Area       Height      Area
  #   [min]        [min]   [mAU*s]      [mAU]        %
----|-------|----|-------|----------|----------|--------|
   1  11.411 BB    0.2026  396.47821   29.92487  80.6525
   2  13.468 BB    0.2327   95.10999    6.28029  19.3475
```

Supplementary Figure 166 HPLC spectra for **3n**

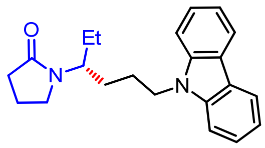

3o

HPLC data using *rac*-**L4**

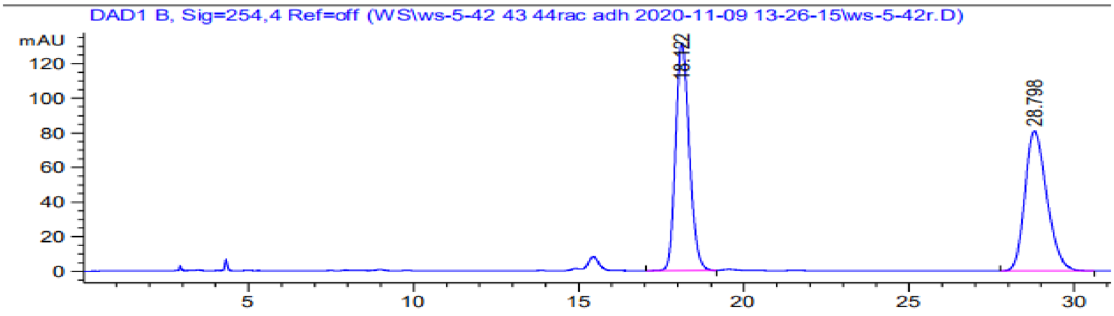

Signal 1: DAD1 B, Sig=254,4 Ref=off

Peak #	RetTime [min]	Type	Width [min]	Area [mAU*s]	Height [mAU]	Area %
1	18.122	BB	0.4444	3780.10547	131.09605	50.1377
2	28.798	BB	0.7135	3759.33765	80.99487	49.8623

HPLC data using (1*S*, 2*R*)-**L9**

From (*E*)-acyl enamine

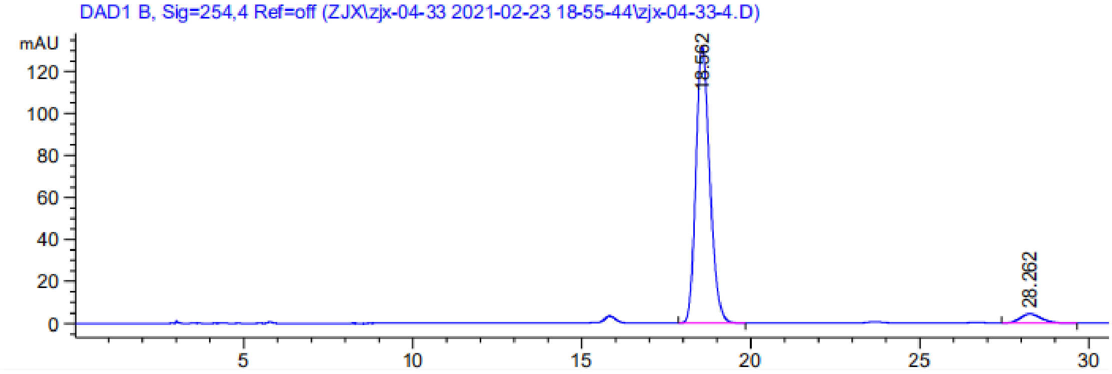

Signal 1: DAD1 B, Sig=254,4 Ref=off

Peak #	RetTime [min]	Type	Width [min]	Area [mAU*s]	Height [mAU]	Area %
1	18.562	BB	0.4398	3765.59058	131.62482	95.0474
2	28.262	BB	0.6156	196.21391	4.49198	4.9526

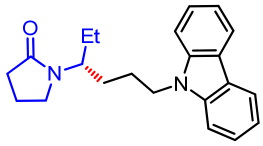

3o

From (*Z*)-acyl enamine

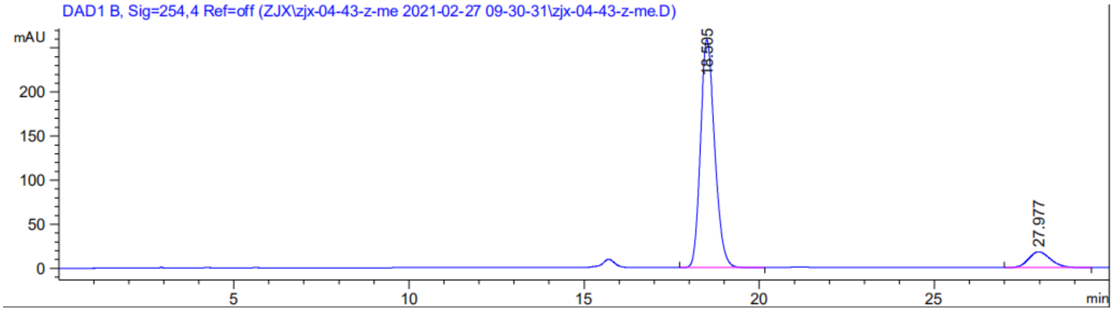

Signal 1: DAD1 B, Sig=254,4 Ref=off

Peak #	RetTime [min]	Type	Width [min]	Area [mAU*s]	Height [mAU]	Area %
1	18.505	BB	0.4347	7280.11328	258.42679	90.3824
2	27.977	BB	0.6723	774.68188	17.85092	9.6176

Supplementary Figure 167 HPLC spectra for **3o**

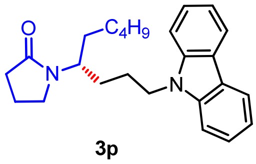

3p

HPLC data using *rac*-**L4**

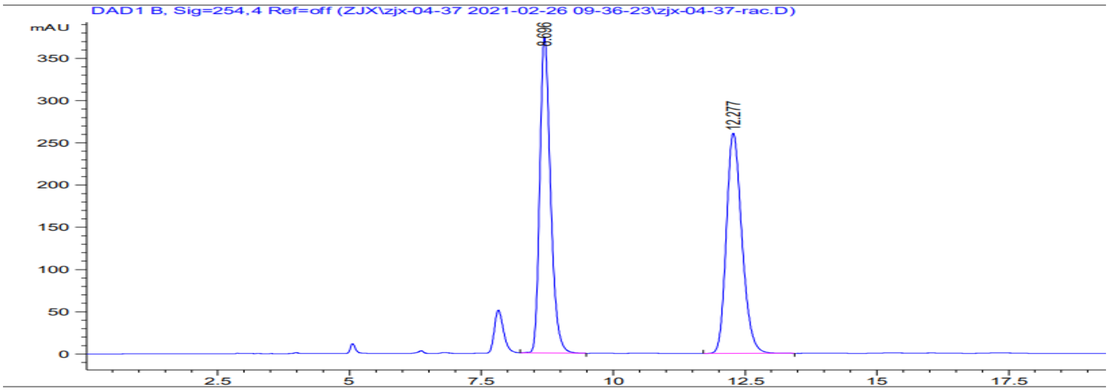

Signal 1: DAD1 B, Sig=254,4 Ref=off

Peak #	RetTime [min]	Type	Width [min]	Area [mAU*s]	Height [mAU]	Area %
1	8.696	BB	0.2179	5309.87402	373.31342	49.7987
2	12.277	BB	0.3155	5352.80713	260.72894	50.2013

HPLC data using (1*S*, 2*R*)-**L9**

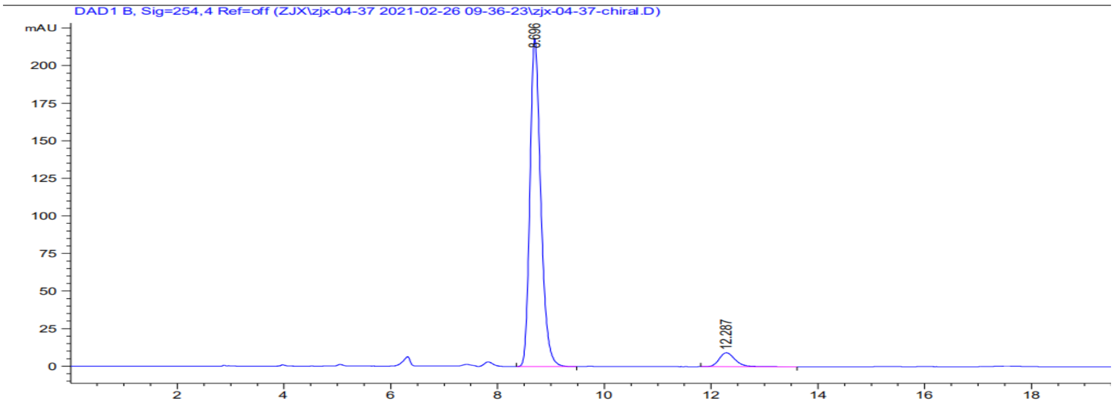

Signal 1: DAD1 B, Sig=254,4 Ref=off

Peak #	RetTime [min]	Type	Width [min]	Area [mAU*s]	Height [mAU]	Area %
1	8.696	BB	0.2170	3081.21606	217.88864	94.0861
2	12.287	BB	0.3187	193.67371	9.31107	5.9139

Supplementary Figure 168 HPLC spectra for **3p**

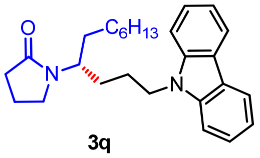

3q

HPLC data using *rac*-**L4**

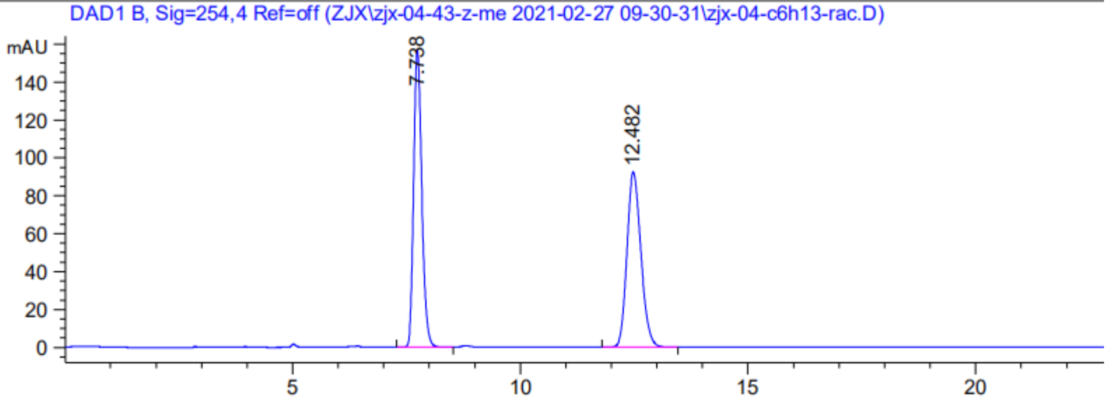

Signal 1: DAD1 B, Sig=254,4 Ref=off

Peak #	RetTime [min]	Type	Width [min]	Area [mAU*s]	Height [mAU]	Area %
1	7.738	BB	0.1911	1946.25952	156.49745	49.7525
2	12.482	BB	0.3305	1965.62134	92.29543	50.2475

HPLC data using (1*S*, 2*R*)-**L9**

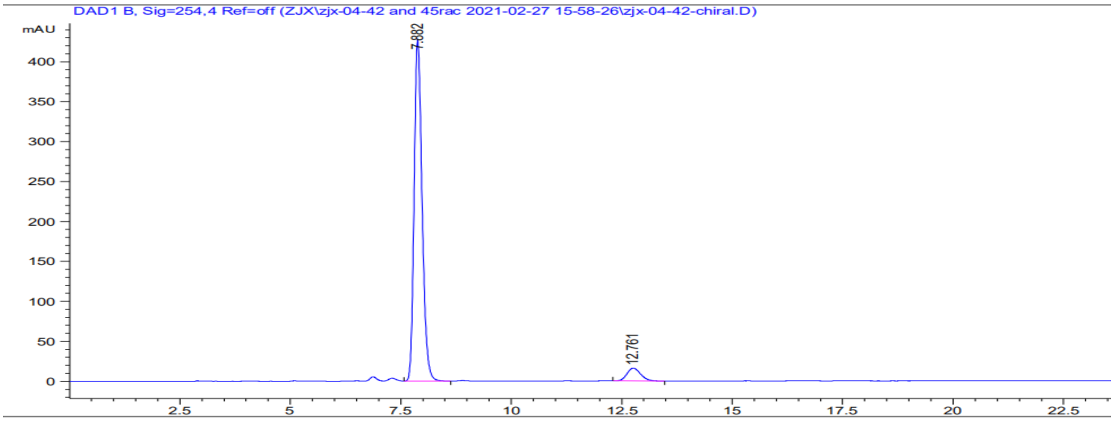

Signal 1: DAD1 B, Sig=254,4 Ref=off

Peak #	RetTime [min]	Type	Width [min]	Area [mAU*s]	Height [mAU]	Area %
1	7.882	VB	0.1922	5344.50830	426.52005	93.9911
2	12.761	BB	0.3275	341.67661	16.10768	6.0089

Supplementary Figure 169 HPLC spectra for **3q**

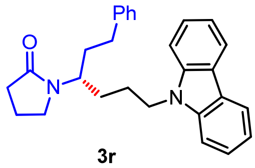

3r

HPLC data using *rac*-**L4**

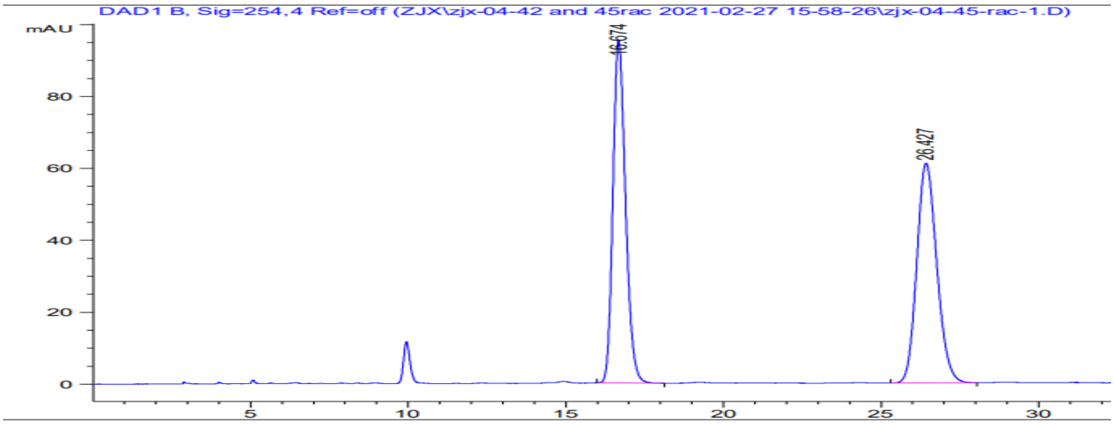

Signal 1: DAD1 B, Sig=254,4 Ref=off

Peak #	RetTime [min]	Type	Width [min]	Area [mAU*s]	Height [mAU]	Area %
1	16.674	BB	0.4284	2645.67285	95.16203	49.5450
2	26.427	BB	0.6805	2694.27075	61.09250	50.4550

HPLC data using (1*S*, 2*R*)-**L9**

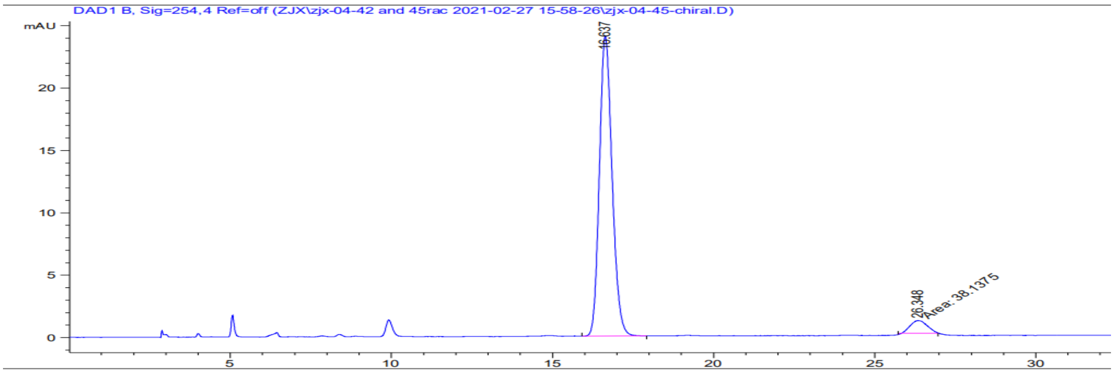

Signal 1: DAD1 B, Sig=254,4 Ref=off

Peak #	RetTime [min]	Type	Width [min]	Area [mAU*s]	Height [mAU]	Area %
1	16.637	BB	0.4223	660.06274	24.04069	94.5377
2	26.348	MM	0.6180	38.13750	1.02849	5.4623

Supplementary Figure 170 HPLC spectra for **3r**

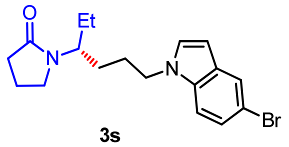

3s

HPLC data using *rac*-**L4**

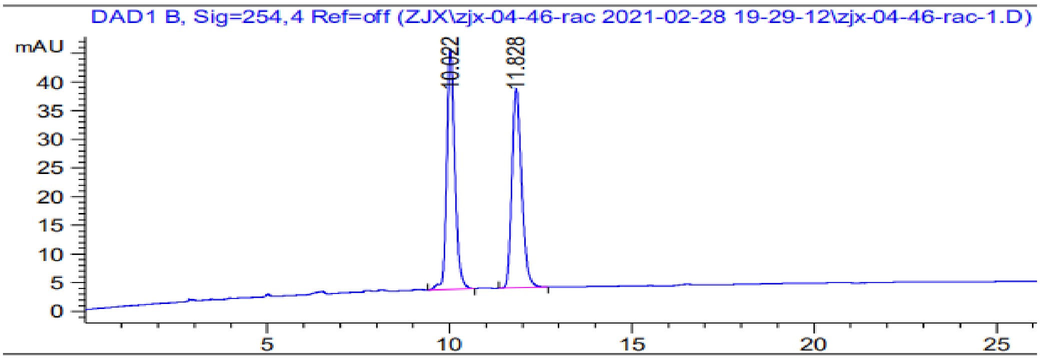

Signal 1: DAD1 B, Sig=254,4 Ref=off

Peak #	RetTime [min]	Type	Width [min]	Area [mAU*s]	Height [mAU]	Area %
1	10.022	BB	0.2350	648.20911	41.78112	50.2413
2	11.828	BB	0.2839	641.98151	34.69312	49.7587

HPLC data using (1*S*, 2*R*)-**L9**

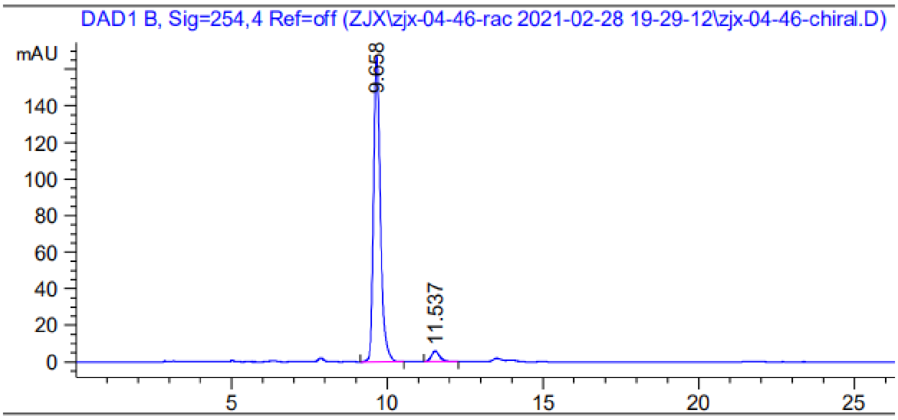

Signal 1: DAD1 B, Sig=254,4 Ref=off

Peak #	RetTime [min]	Type	Width [min]	Area [mAU*s]	Height [mAU]	Area %
1	9.658	BB	0.2298	2511.08911	166.60023	95.9251
2	11.537	BB	0.2757	106.67098	5.87980	4.0749

Supplementary Figure 171 HPLC spectra for **3s**

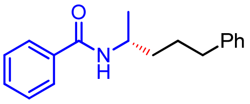

4a

HPLC data using *rac*-**L41**

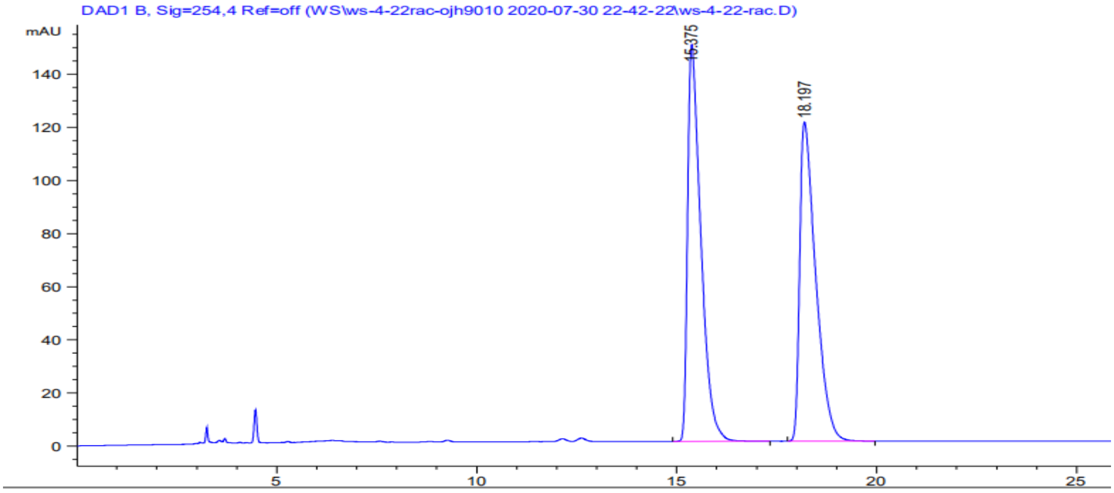

Signal 1: DAD1 B, Sig=254,4 Ref=off

Peak #	RetTime [min]	Type	Width [min]	Area [mAU*s]	Height [mAU]	Area %
1	15.375	BB	0.3424	3433.47827	149.23711	50.6538
2	18.197	BB	0.4132	3344.84473	120.00629	49.3462

HPLC data using (1*S*, 2*R*)-**L41**

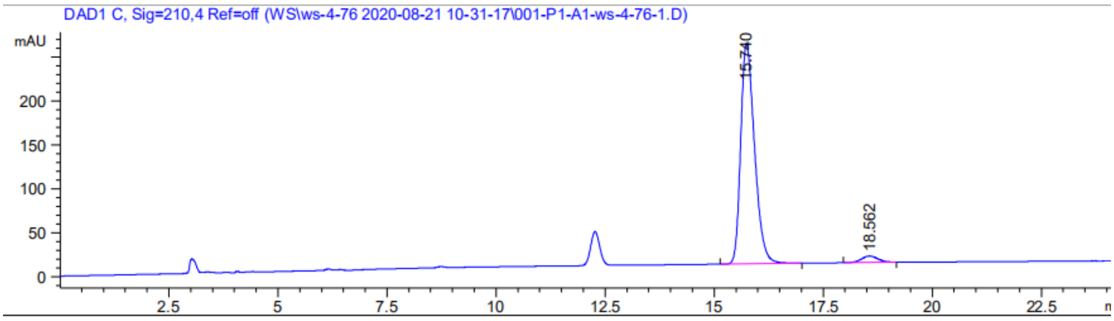

Signal 1: DAD1 B, Sig=254,4 Ref=off

Peak #	RetTime [min]	Type	Width [min]	Area [mAU*s]	Height [mAU]	Area %
1	15.740	BB	0.3380	769.80188	35.07842	96.4995
2	18.561	MM	0.4523	27.92457	1.02897	3.5005

Supplementary Figure 172 HPLC spectra for **4a**

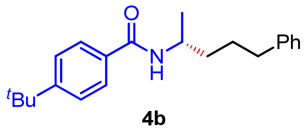

4b

HPLC data using *rac*-**L41**

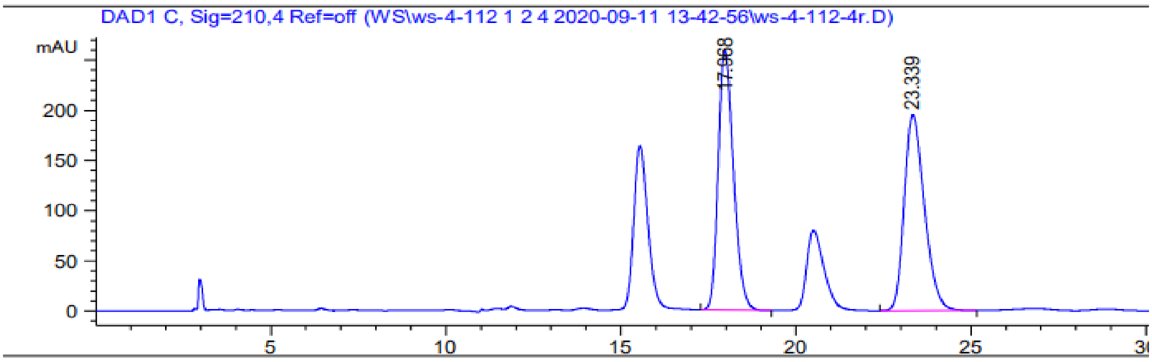

Signal 2: DAD1 C, Sig=210,4 Ref=off

Peak #	RetTime [min]	Type	Width [min]	Area [mAU*s]	Height [mAU]	Area %
1	18.272	BB	0.4748	7861.26758	255.64638	50.0784
2	23.730	BB	0.6248	7836.65625	192.55989	49.9216

HPLC data using (1*S*, 2*R*)-**L41**

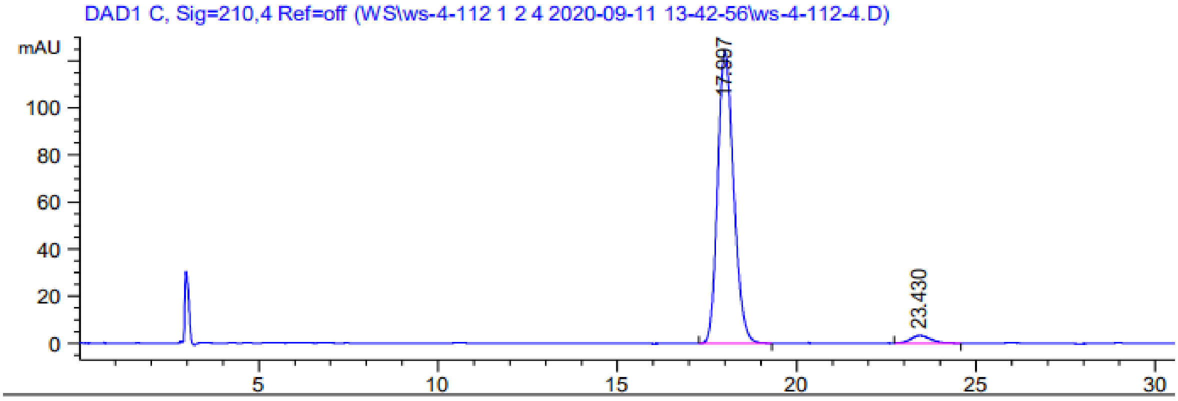

Signal 2: DAD1 C, Sig=210,4 Ref=off

Peak #	RetTime [min]	Type	Width [min]	Area [mAU*s]	Height [mAU]	Area %
1	17.997	BB	0.4783	3856.76074	124.21100	96.8151
2	23.430	BB	0.5098	126.87569	3.29093	3.1849

Supplementary Figure 173 HPLC spectra for **4b**

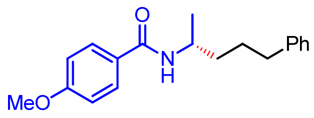

4c

HPLC data using *rac*-**L41**

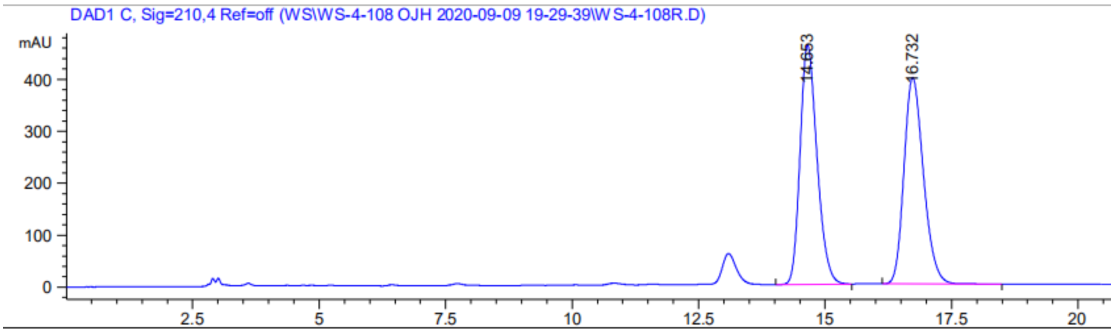

Signal 1: DAD1 B, Sig=254,4 Ref=off

Peak #	RetTime [min]	Type	Width [min]	Area [mAU*s]	Height [mAU]	Area %
1	14.653	BB	0.3558	6103.52783	263.89728	50.1762
2	16.732	BB	0.4114	6060.66162	227.08655	49.8238

HPLC data using (1*S*, 2*R*)-**L41**

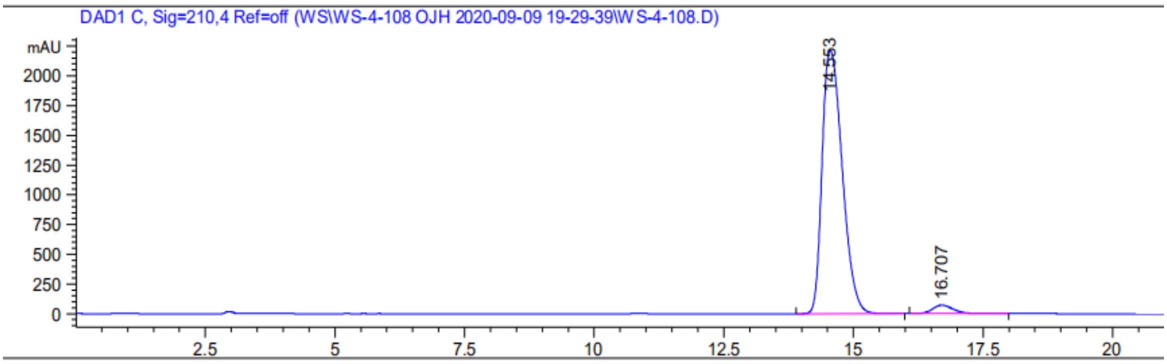

Signal 1: DAD1 B, Sig=254,4 Ref=off

Peak #	RetTime [min]	Type	Width [min]	Area [mAU*s]	Height [mAU]	Area %
1	14.552	BB	0.3789	3.73830e4	1509.63159	97.1056
2	16.707	BB	0.4241	1114.27856	40.36271	2.8944

Supplementary Figure 174 HPLC spectra for **4c**

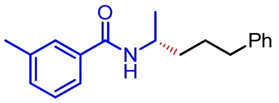

4d

HPLC data using *rac*-**L41**

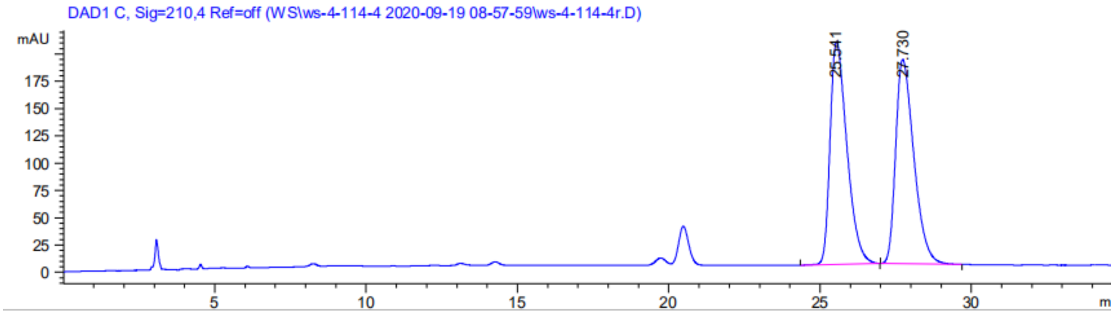

Signal 2: DAD1 C, Sig=210,4 Ref=off

Peak #	RetTime [min]	Type	Width [min]	Area [mAU*s]	Height [mAU]	Area %
1	25.541	BB	0.5911	7892.07764	204.10544	50.2065
2	27.730	BB	0.6456	7827.16846	187.28938	49.7935

HPLC data using (1*S*, 2*R*)-**L41**

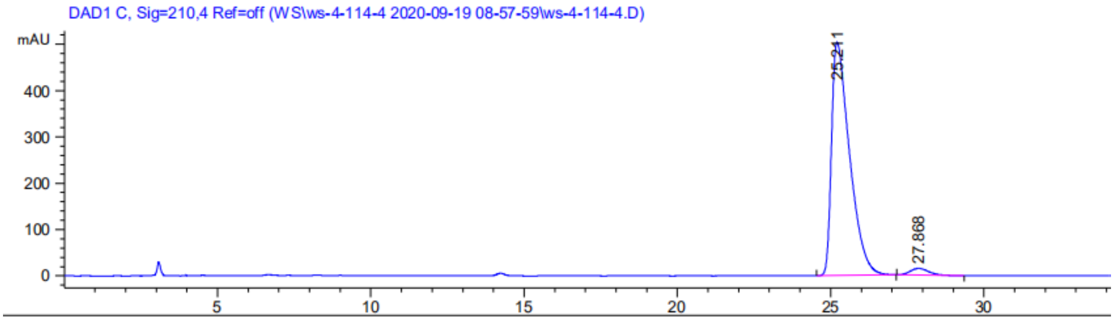

Signal 2: DAD1 C, Sig=210,4 Ref=off

Peak #	RetTime [min]	Type	Width [min]	Area [mAU*s]	Height [mAU]	Area %
1	25.211	BB	0.6043	2.05457e4	503.30212	97.1074
2	27.868	BB	0.6405	612.00598	14.61739	2.8926

Supplementary Figure 175 HPLC spectra for **4d**

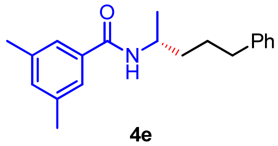

4e

HPLC data using *rac*-**L41**

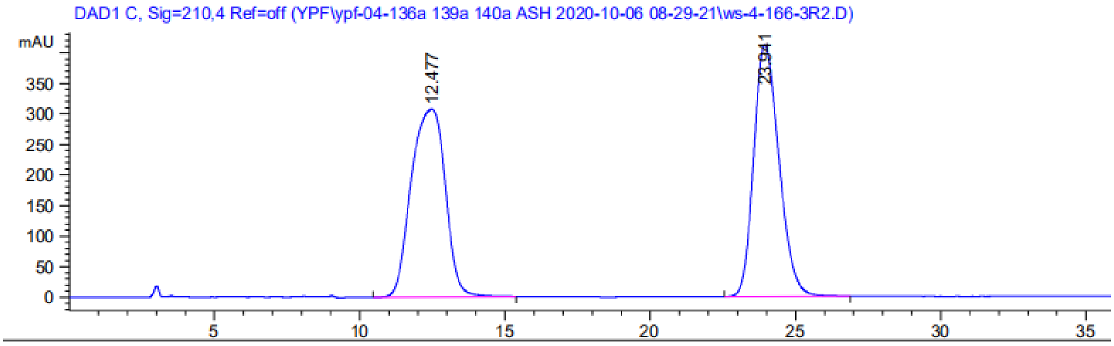

Signal 2: DAD1 C, Sig=210,4 Ref=off

Peak #	RetTime [min]	Type	Width [min]	Area [mAU*s]	Height [mAU]	Area %
1	12.477	BB	1.3437	2.50287e4	307.46527	50.0349
2	23.941	BB	0.9483	2.49938e4	411.68857	49.9651

HPLC data using (1*S*, 2*R*)-**L41**

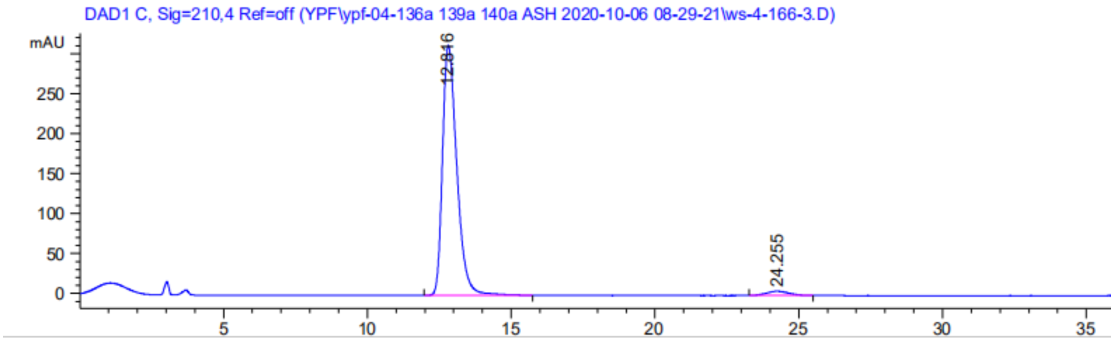

Signal 2: DAD1 C, Sig=210,4 Ref=off

Peak #	RetTime [min]	Type	Width [min]	Area [mAU*s]	Height [mAU]	Area %
1	12.816	BB	0.5314	1.08086e4	312.58813	97.4213
2	24.255	BB	0.6445	286.10043	5.29397	2.5787

Supplementary Figure 176 HPLC spectra for **4e**

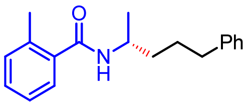

4f

HPLC data using *rac*-**L41**

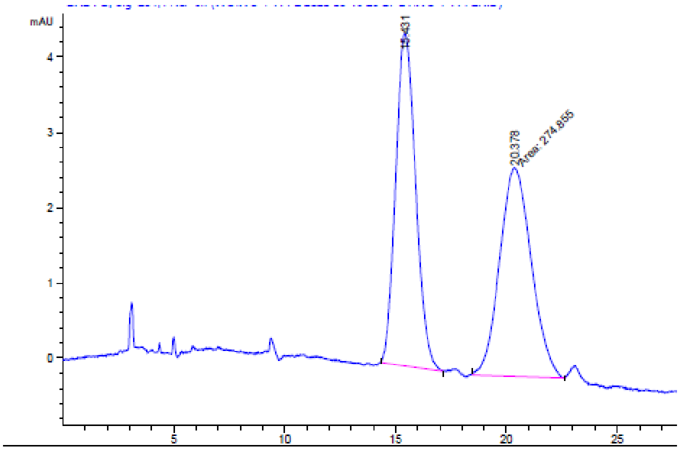

Signal 1: DAD1 B, Sig=254,4 Ref=off

Peak #	RetTime [min]	Type	Width [min]	Area [mAU*s]	Height [mAU]	Area %
1	15.431	BB	0.7620	277.23209	4.41861	50.2152
2	20.378	MM	1.6578	274.85541	2.76325	49.7848

HPLC data using (1*S*, 2*R*)-**L41**

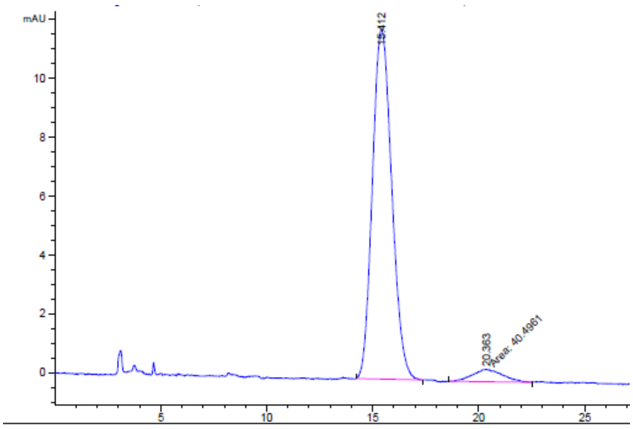

Signal 1: DAD1 B, Sig=254,4 Ref=off

Peak #	RetTime [min]	Type	Width [min]	Area [mAU*s]	Height [mAU]	Area %
1	15.412	BB	0.8419	754.12616	11.85839	94.9037
2	20.363	MM	1.6287	40.49613	4.14401e-1	5.0963

Supplementary Figure 177 HPLC spectra for **4f**

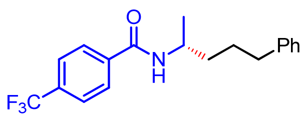

4g

HPLC data using *rac*-**L41**

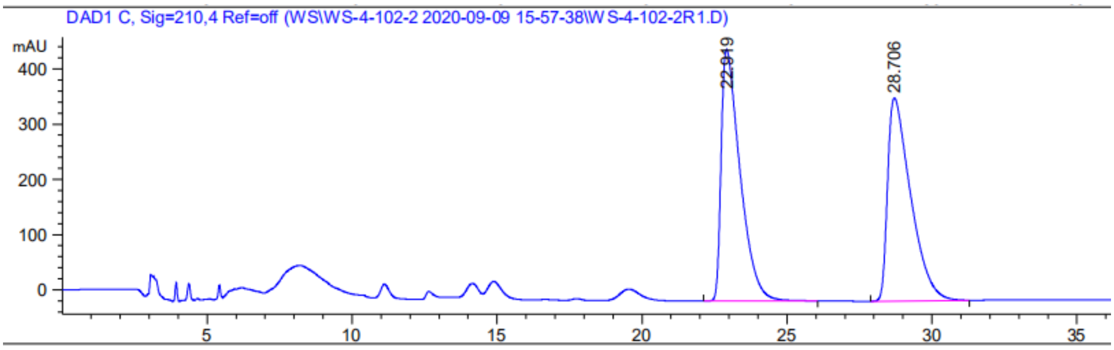

Signal 2: DAD1 C, Sig=210,4 Ref=off

Peak #	RetTime [min]	Type	Width [min]	Area [mAU*s]	Height [mAU]	Area %
1	22.919	BB	0.6790	2.06464e4	455.32178	50.4032
2	28.706	BB	0.8192	2.03161e4	367.37448	49.5968

HPLC data using (1*S*, 2*R*)-**L41**

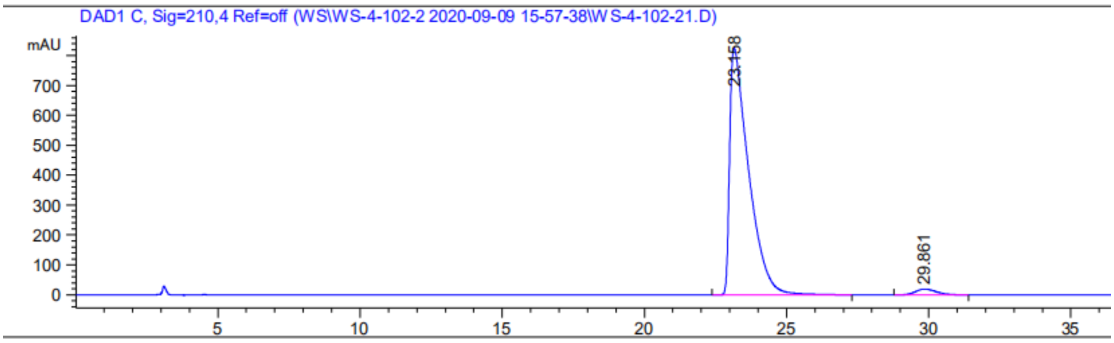

Signal 2: DAD1 C, Sig=210,4 Ref=off

Peak #	RetTime [min]	Type	Width [min]	Area [mAU*s]	Height [mAU]	Area %
1	23.158	BB	0.6670	3.83487e4	826.95801	97.3885
2	29.861	BB	0.7564	1028.32739	19.98254	2.6115

Supplementary Figure 178 HPLC spectra for **4g**

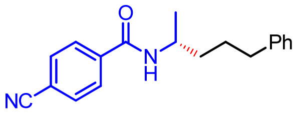

4h

HPLC data using *rac*-**L41**

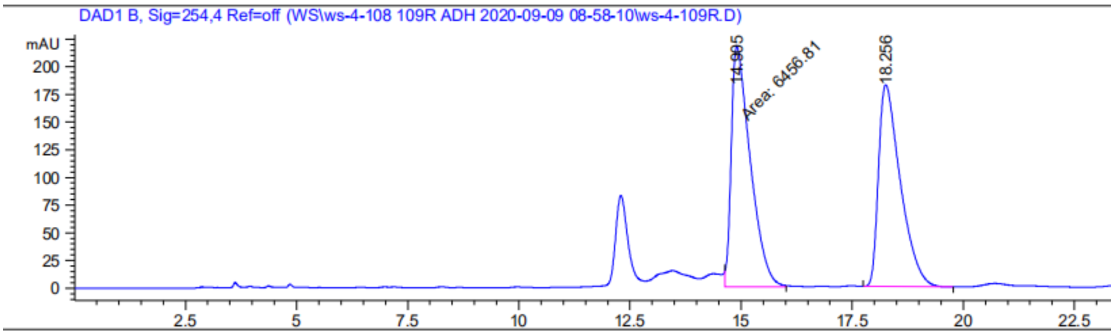

Signal 1: DAD1 B, Sig=254,4 Ref=off

Peak #	RetTime [min]	Type	Width [min]	Area [mAU*s]	Height [mAU]	Area %
1	14.905	MM	0.4960	6456.80518	216.96210	50.8167
2	18.256	BB	0.5098	6249.27002	182.36842	49.1833

HPLC data using (1*S*, 2*R*)-**L41**

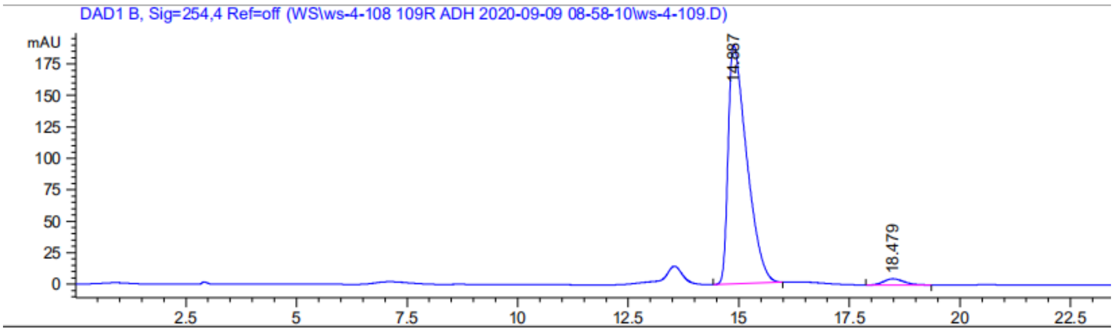

Signal 1: DAD1 B, Sig=254,4 Ref=off

Peak #	RetTime [min]	Type	Width [min]	Area [mAU*s]	Height [mAU]	Area %
1	14.887	BB	0.4361	5541.29883	190.07423	97.2838
2	18.479	BB	0.4571	154.71616	4.94406	2.7162

Supplementary Figure 179 HPLC spectra for **4h**

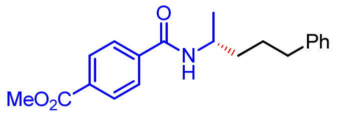

4i

HPLC data using *rac*-**L41**

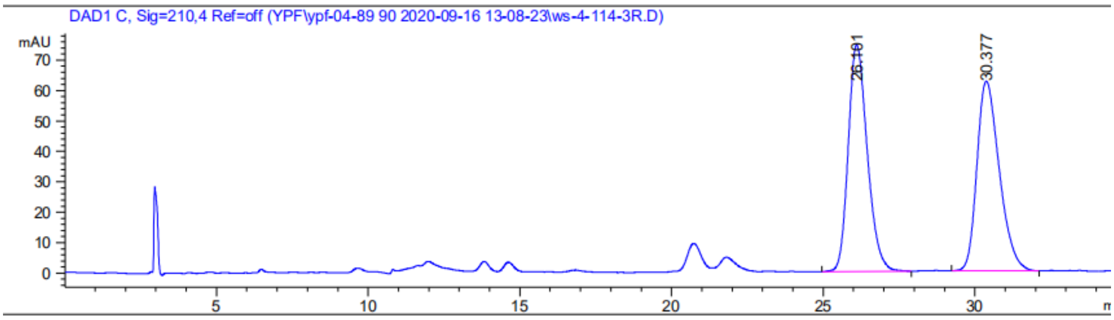

Signal 1: DAD1 B, Sig=254,4 Ref=off

Peak #	RetTime [min]	Type	Width [min]	Area [mAU*s]	Height [mAU]	Area %
1	26.101	BB	0.6706	2409.75073	55.49414	50.2112
2	30.376	BB	0.7825	2389.47510	46.58204	49.7888

HPLC data using (1*S*, 2*R*)-**L41**

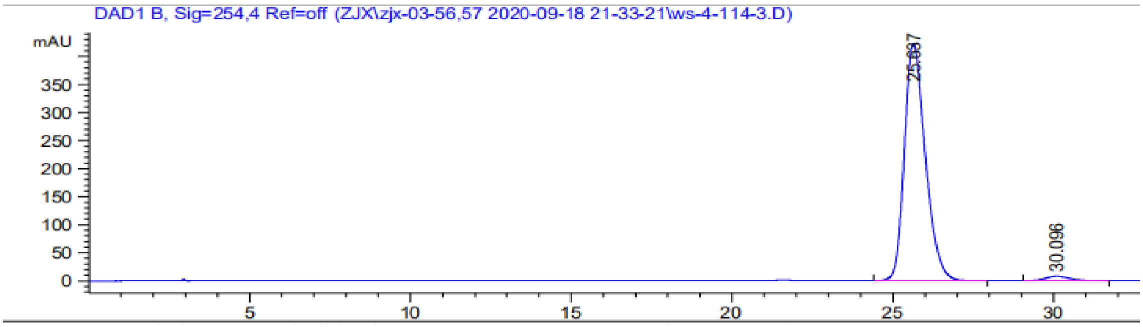

Signal 1: DAD1 B, Sig=254,4 Ref=off

Peak #	RetTime [min]	Type	Width [min]	Area [mAU*s]	Height [mAU]	Area %
1	25.637	BB	0.6950	1.91254e4	421.77936	97.9454
2	30.096	BB	0.7208	401.18561	7.92278	2.0546

Supplementary Figure 180 HPLC spectra for **4i**

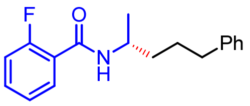

4j

HPLC data using *rac*-**L41**

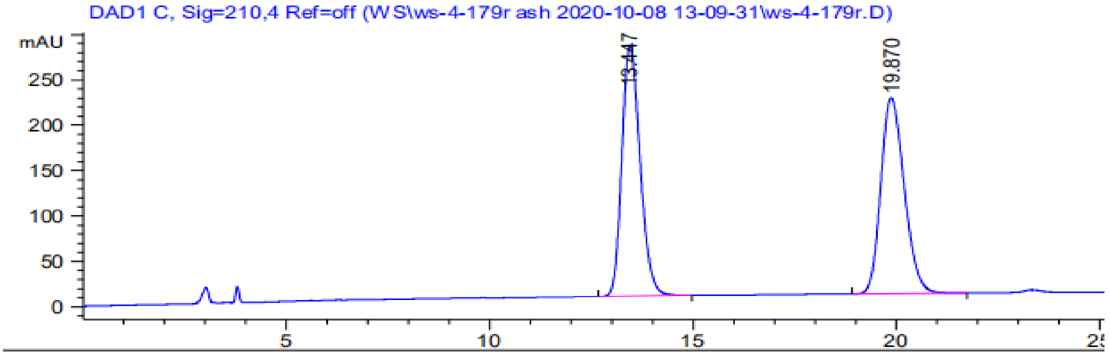

Signal 2: DAD1 C, Sig=210,4 Ref=off

Peak #	RetTime [min]	Type	Width [min]	Area [mAU*s]	Height [mAU]	Area %
1	13.447	BB	0.4829	8595.27930	276.31097	49.9337
2	19.870	BB	0.6219	8618.10742	215.79897	50.0663

HPLC data using (1*S*, 2*R*)-**L41**

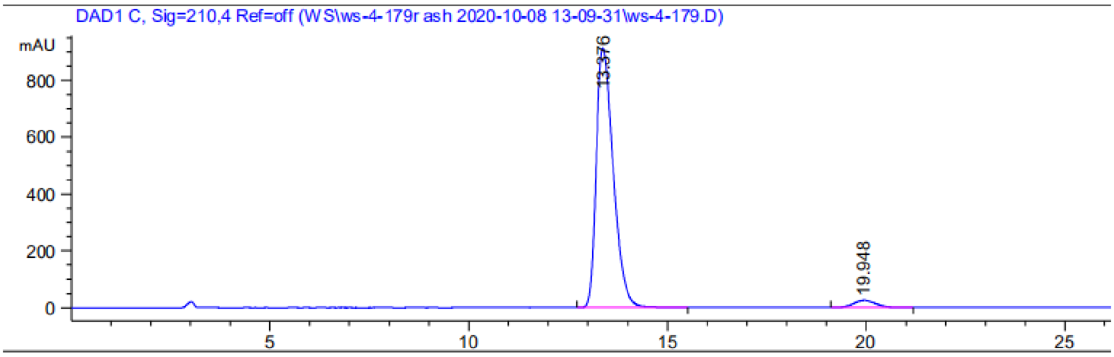

Signal 2: DAD1 C, Sig=210,4 Ref=off

Peak #	RetTime [min]	Type	Width [min]	Area [mAU*s]	Height [mAU]	Area %
1	13.376	BB	0.4586	2.70995e4	912.13721	96.5877
2	19.948	BB	0.5781	957.39355	24.92624	3.4123

Supplementary Figure 181 HPLC spectra for **4j**

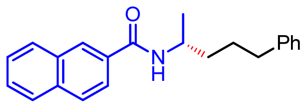

4k

HPLC data using *rac*-**L41**

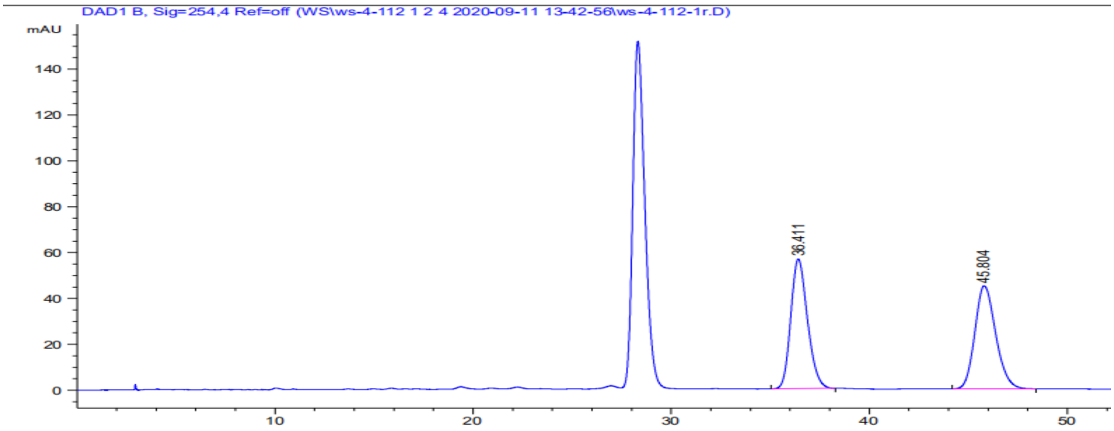

Signal 1: DAD1 B, Sig=254,4 Ref=off

Peak #	RetTime [min]	Type	Width [min]	Area [mAU*s]	Height [mAU]	Area %
1	36.411	BB	0.9017	3316.05762	56.58816	50.2606
2	45.804	BB	1.1018	3281.66406	44.94928	49.7394

HPLC data using (1*S*, 2*R*)-**L41**

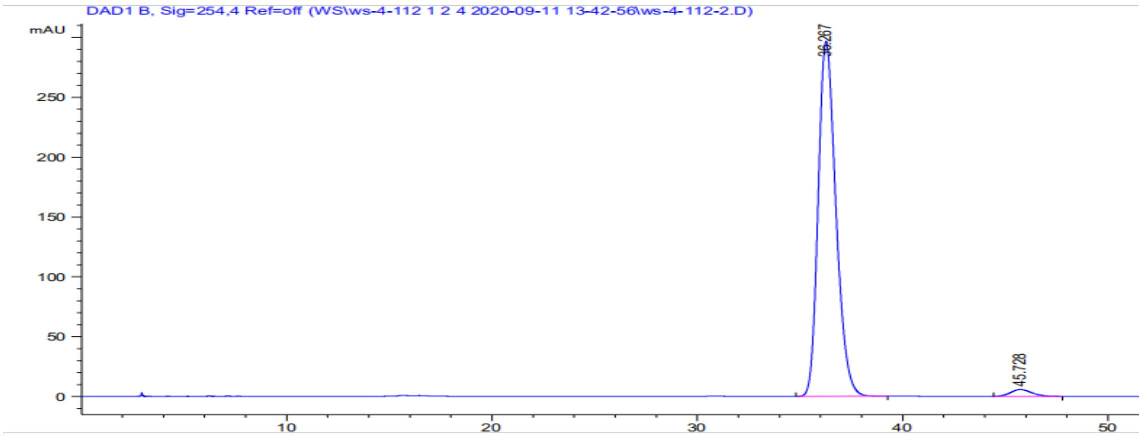

Signal 1: DAD1 B, Sig=254,4 Ref=off

Peak #	RetTime [min]	Type	Width [min]	Area [mAU*s]	Height [mAU]	Area %
1	36.267	BB	0.9257	1.77881e4	296.58603	97.7423
2	45.728	BB	0.8575	410.86990	5.71010	2.2577

Supplementary Figure 182 HPLC spectra for **4k**

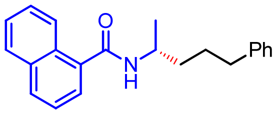

4l

HPLC data using *rac*-**L41**

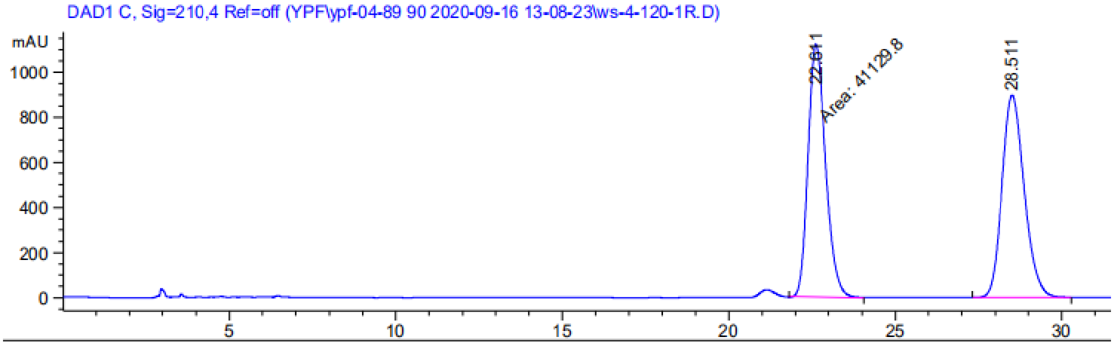

Signal 1: DAD1 B, Sig=254,4 Ref=off

Peak #	RetTime [min]	Type	Width [min]	Area [mAU*s]	Height [mAU]	Area %
1	22.612	BB	0.5704	2727.05957	73.92720	50.0175
2	28.510	BB	0.7073	2725.15259	58.95829	49.9825

HPLC data using (1*S*, 2*R*)-**L41**

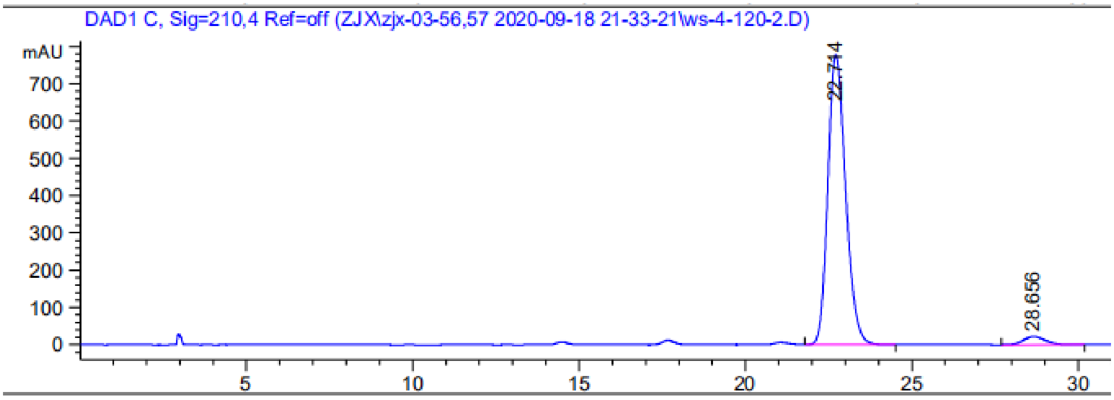

Signal 1: DAD1 B, Sig=254,4 Ref=off

Peak #	RetTime [min]	Type	Width [min]	Area [mAU*s]	Height [mAU]	Area %
1	22.714	BB	0.5743	1894.43262	51.12692	96.4242
2	28.653	MM	0.7778	70.25419	1.50546	3.5758

Supplementary Figure 183 HPLC spectra for **4l**

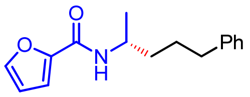

4m

HPLC data using *rac*-**L41**

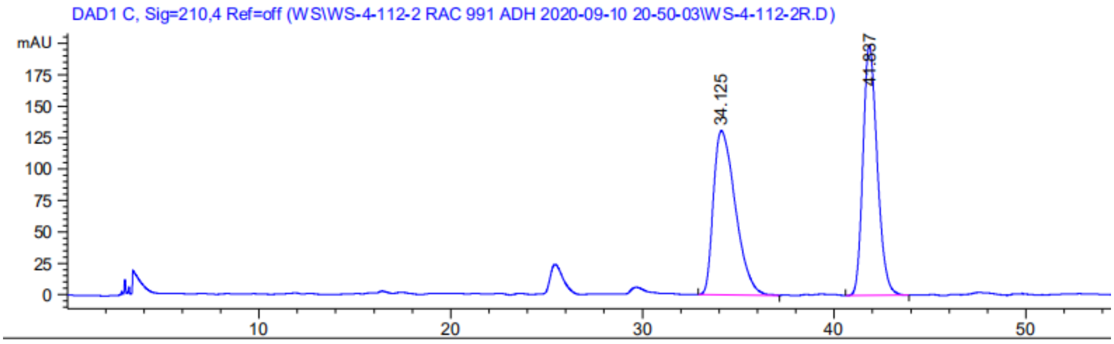

Signal 1: DAD1 B, Sig=254,4 Ref=off

Peak #	RetTime [min]	Type	Width [min]	Area [mAU*s]	Height [mAU]	Area %
1	34.125	BB	1.2268	7566.26465	95.65382	50.2497
2	41.837	BB	0.8038	7491.07910	145.22229	49.7503

HPLC data using (1*S*, 2*R*)-**L41**

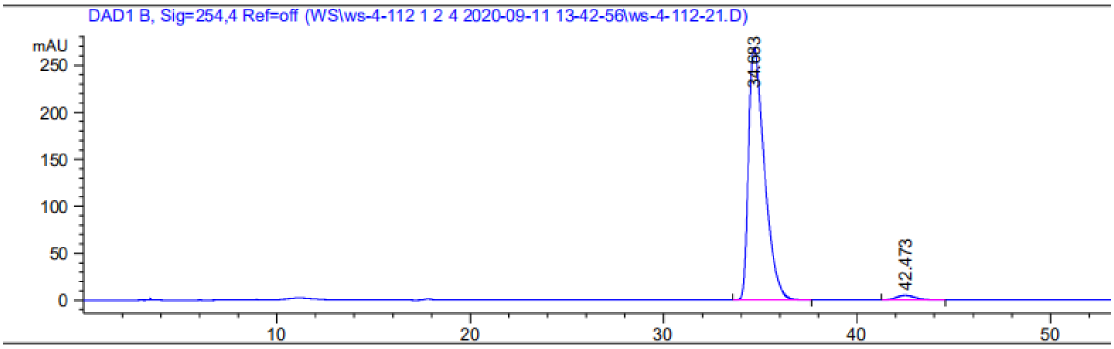

Signal 1: DAD1 B, Sig=254,4 Ref=off

Peak #	RetTime [min]	Type	Width [min]	Area [mAU*s]	Height [mAU]	Area %
1	34.683	BB	0.8724	1.53074e4	267.97458	97.7630
2	42.473	BB	0.8239	350.25467	5.08300	2.2370

Supplementary Figure 184 HPLC spectra for **4m**

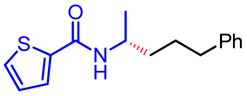

4n

HPLC data using *rac*-**L41**

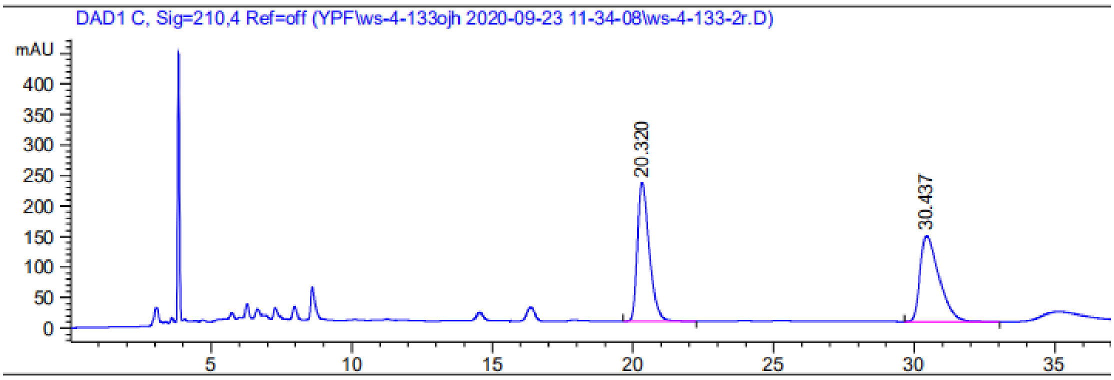

Signal 1: DAD1 B, Sig=254,4 Ref=off

Peak #	RetTime [min]	Type	Width [min]	Area [mAU*s]	Height [mAU]	Area %
1	20.320	BB	0.4468	6260.10156	214.31544	50.2614
2	30.436	BB	0.6956	6194.99756	132.96785	49.7386

HPLC data using (1*S*, 2*R*)-**L41**

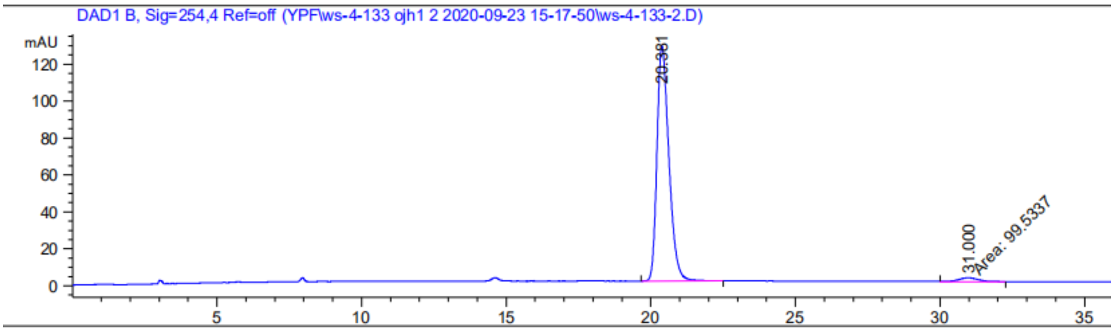

Signal 1: DAD1 B, Sig=254,4 Ref=off

Peak #	RetTime [min]	Type	Width [min]	Area [mAU*s]	Height [mAU]	Area %
1	20.381	BB	0.4408	3652.73340	127.27570	97.3474
2	31.000	MM	0.8027	99.53368	2.06668	2.6526

Supplementary Figure 185 HPLC spectra for **4n**

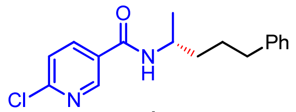

4o

HPLC data using *rac*-**L41**

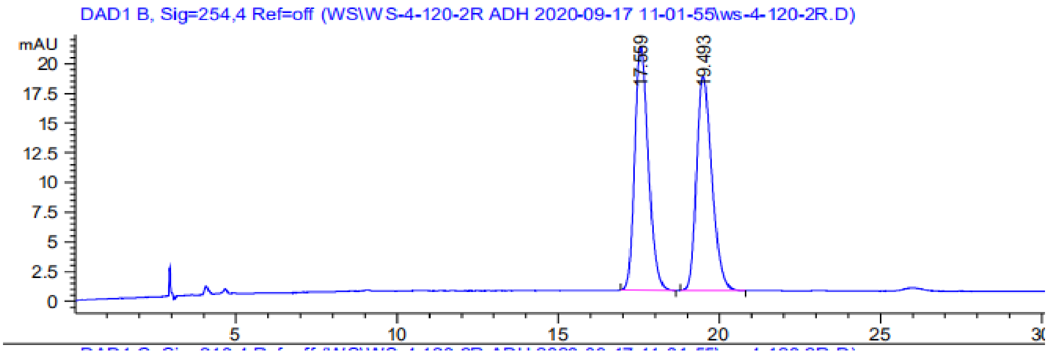

Signal 1: DAD1 B, Sig=254,4 Ref=off

Peak #	RetTime [min]	Type	Width [min]	Area [mAU*s]	Height [mAU]	Area %
1	17.559	BB	0.4518	603.29633	20.46991	50.2942
2	19.493	BB	0.5064	596.23773	18.00327	49.7058

HPLC data using (1*S*, 2*R*)-**L41**

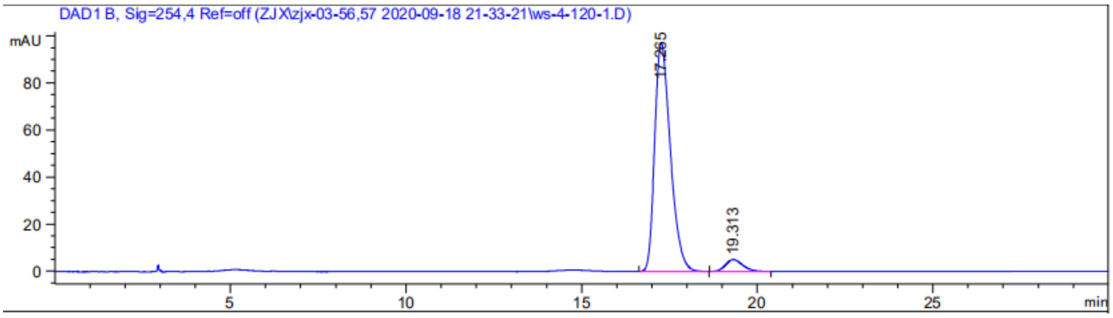

Signal 1: DAD1 B, Sig=254,4 Ref=off

Peak #	RetTime [min]	Type	Width [min]	Area [mAU*s]	Height [mAU]	Area %
1	17.265	BB	0.4788	3016.78931	96.99947	94.6410
2	19.313	BB	0.5029	170.82443	5.02174	5.3590

Supplementary Figure 186 HPLC spectra for **4o**

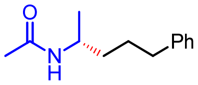

4p

HPLC data using *rac*-**L41**

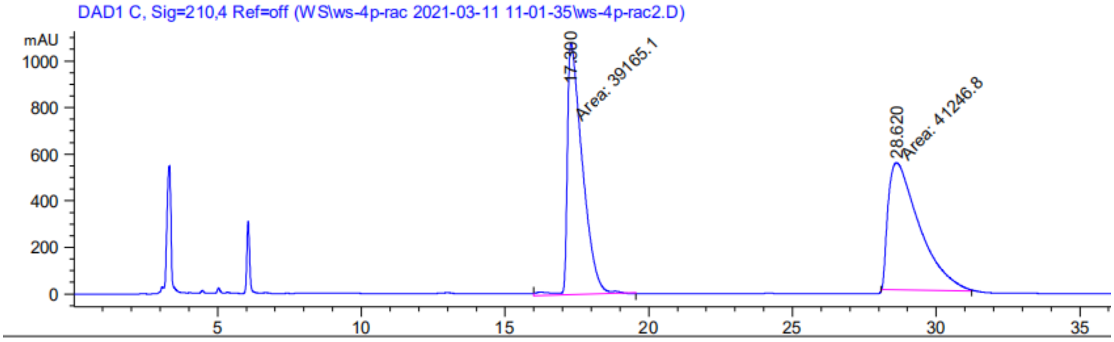

Signal 2: DAD1 C, Sig=210,4 Ref=off

Peak #	RetTime [min]	Type	Width [min]	Area [mAU*s]	Height [mAU]	Area %
1	17.300	MM	0.6060	3.91651e4	1077.20618	48.7056
2	28.620	MM	1.2630	4.12468e4	544.28412	51.2944

HPLC data using (1*S*, 2*R*)-**L41**

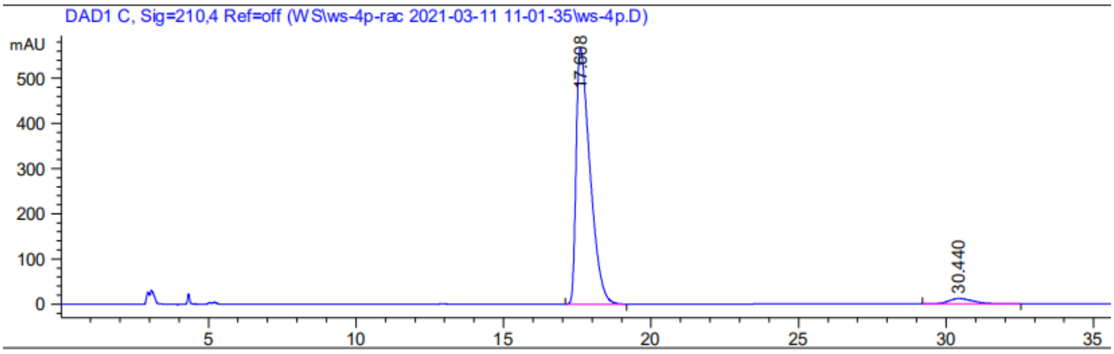

Signal 2: DAD1 C, Sig=210,4 Ref=off

Peak #	RetTime [min]	Type	Width [min]	Area [mAU*s]	Height [mAU]	Area %
1	17.608	BB	0.4924	1.84919e4	567.06903	96.4493
2	30.440	BB	0.8187	680.77148	11.84172	3.5507

Supplementary Figure 187 HPLC spectra for **4p**

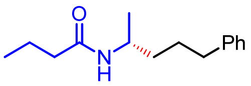

4q

HPLC data using *rac*-**L41**

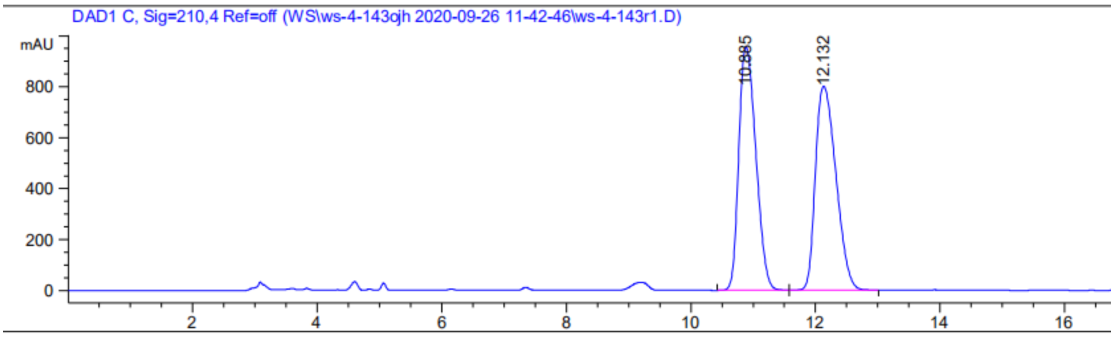

Signal 2: DAD1 C, Sig=210,4 Ref=off

Peak #	RetTime [min]	Type	Width [min]	Area [mAU*s]	Height [mAU]	Area %
1	10.885	BB	0.3019	1.81712e4	955.35120	49.8140
2	12.132	BB	0.3667	1.83069e4	800.82587	50.1860

HPLC data using (1*S*, 2*R*)-**L41**

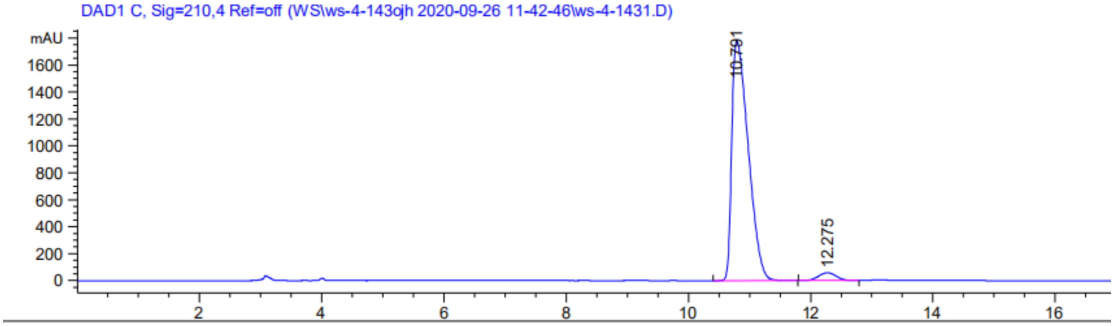

Signal 2: DAD1 C, Sig=210,4 Ref=off

Peak #	RetTime [min]	Type	Width [min]	Area [mAU*s]	Height [mAU]	Area %
1	10.791	BB	0.2943	3.32358e4	1776.50806	96.6480
2	12.275	BB	0.3172	1152.69360	57.67520	3.3520

Supplementary Figure 188 HPLC spectra for **4q**

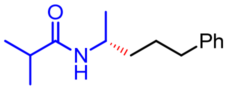

4r

HPLC data using *rac*-**L41**

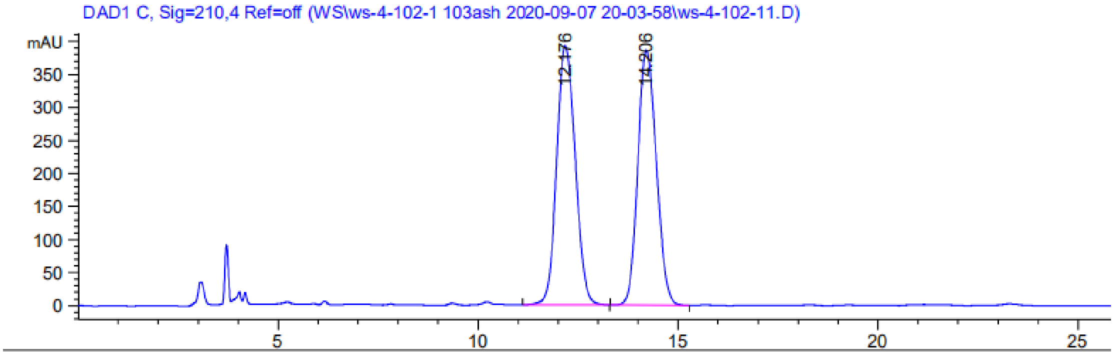

Signal 2: DAD1 C, Sig=210,4 Ref=off

Peak #	RetTime [min]	Type	Width [min]	Area [mAU*s]	Height [mAU]	Area %
1	12.176	BB	0.5121	1.26382e4	392.05344	50.4535
2	14.206	BB	0.5093	1.24110e4	385.77988	49.5465

HPLC data using (1*S*, 2*R*)-**L41**

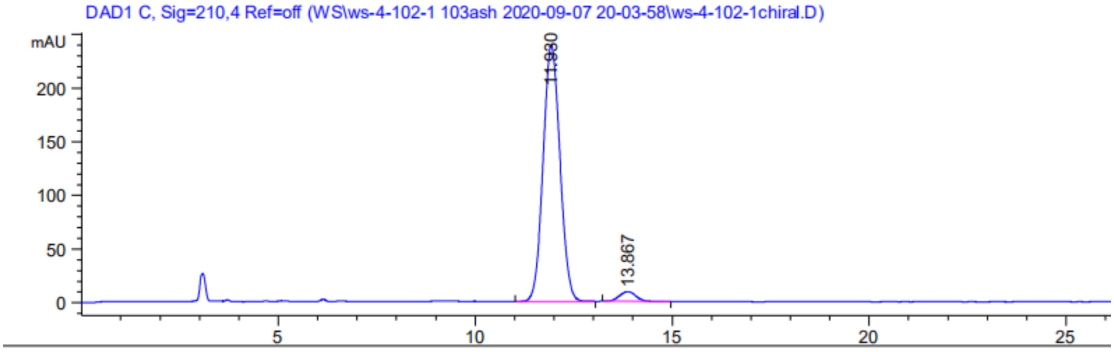

Signal 2: DAD1 C, Sig=210,4 Ref=off

Peak #	RetTime [min]	Type	Width [min]	Area [mAU*s]	Height [mAU]	Area %
1	11.930	BB	0.4633	7102.89697	238.58983	96.3932
2	13.867	BB	0.4581	265.77328	9.06378	3.6068

Supplementary Figure 189 HPLC spectra for**4r**

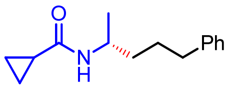

4s

HPLC data using *rac*-**L41**

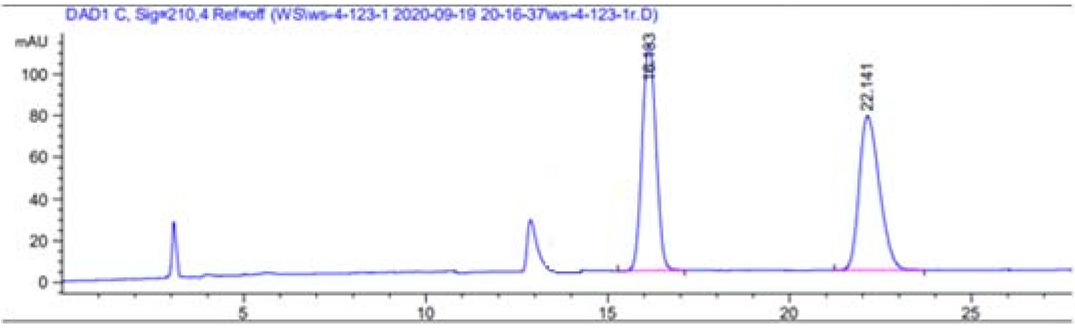

Signal 2: DAD1 C, Sig=210,4 Ref=off

Peak #	RetTime [min]	Type	Width [min]	Area [mAU*s]	Height [mAU]	Area %
1	16.133	BB	0.4225	2891.29932	108.62558	50.2074
2	22.141	BB	0.6059	2867.40771	74.02425	49.7926

HPLC data using (1*S*, 2*R*)-**L41**

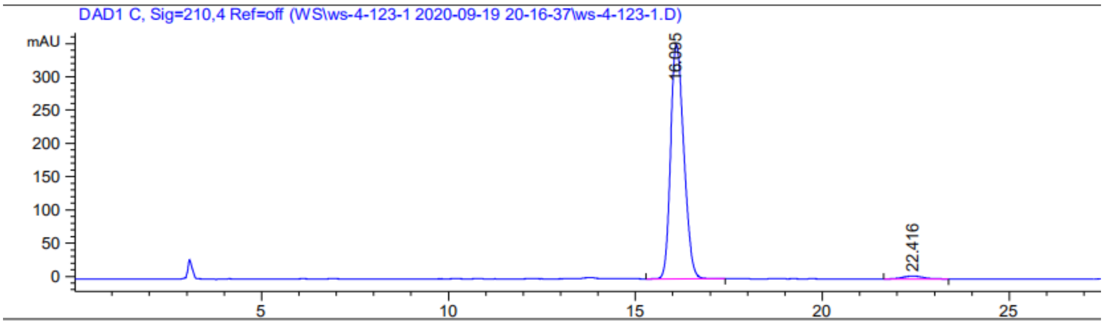

Signal 2: DAD1 C, Sig=210,4 Ref=off

Peak #	RetTime [min]	Type	Width [min]	Area [mAU*s]	Height [mAU]	Area %
1	16.095	BB	0.3851	8676.32910	352.51907	98.1317
2	22.416	BB	0.4985	165.18613	4.33569	1.8683

Supplementary Figure 190 HPLC spectra for **4s**

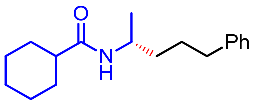

4t

HPLC data using *rac*-**L41**

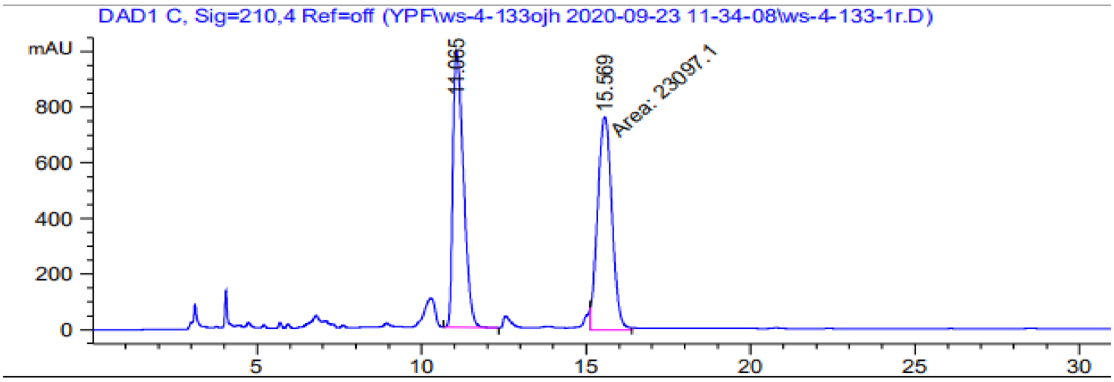

Signal 2: DAD1 C, Sig=210,4 Ref=off

Peak #	RetTime [min]	Type	Width [min]	Area [mAU*s]	Height [mAU]	Area %
1	11.065	VB	0.3502	2.23079e4	992.78485	49.1310
2	15.569	MM	0.5036	2.30971e4	764.47174	50.8690

HPLC data using (1*S*, 2*R*)-**L41**

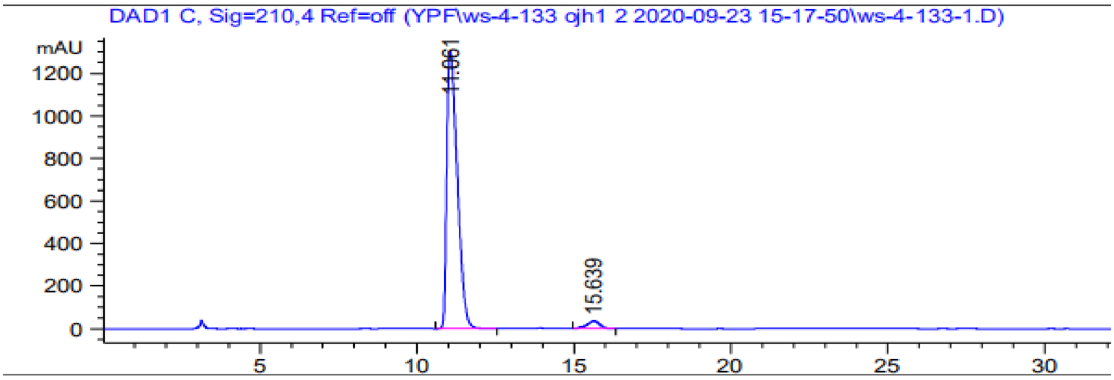

Signal 2: DAD1 C, Sig=210,4 Ref=off

Peak #	RetTime [min]	Type	Width [min]	Area [mAU*s]	Height [mAU]	Area %
1	11.061	BB	0.3616	3.03003e4	1301.64209	96.7727
2	15.639	BB	0.4329	1010.47668	35.40841	3.2273

Supplementary Figure 191 HPLC spectra for **4t**

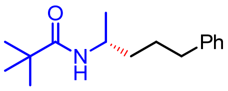

4u

HPLC data using *rac*-**L41**

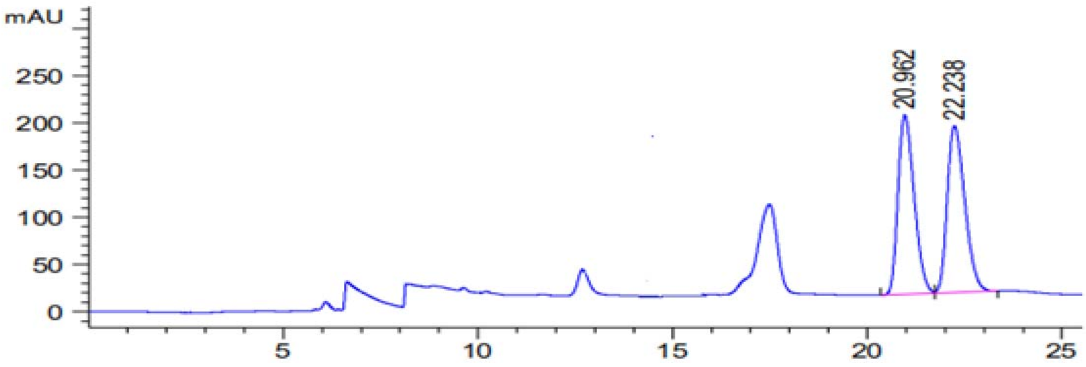

Signal 2: DAD1 C, Sig=210,4 Ref=off

Peak #	RetTime [min]	Type	Width [min]	Area [mAU*s]	Height [mAU]	Area %
1	20.962	BV	0.4514	5491.43555	189.90656	49.6866
2	22.238	VB	0.4912	5560.70264	176.65302	50.3134

S

HPLC data using (1*S*, 2*R*)-**L41**

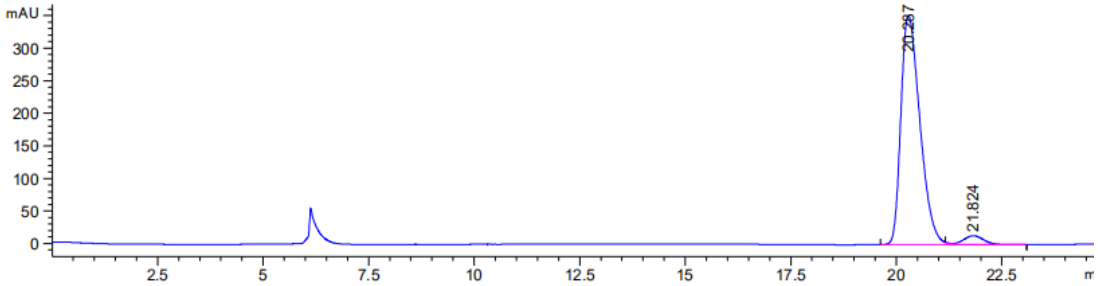

Signal 2: DAD1 C, Sig=210,4 Ref=off

Peak #	RetTime [min]	Type	Width [min]	Area [mAU*s]	Height [mAU]	Area %
1	20.287	BV R	0.4871	1.10070e4	351.68823	96.0912
2	21.824	VB E	0.4938	447.74805	13.68104	3.9088

Supplementary Figure 192 HPLC spectra for **4u**

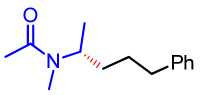

4v

HPLC data using *rac*-**L41**

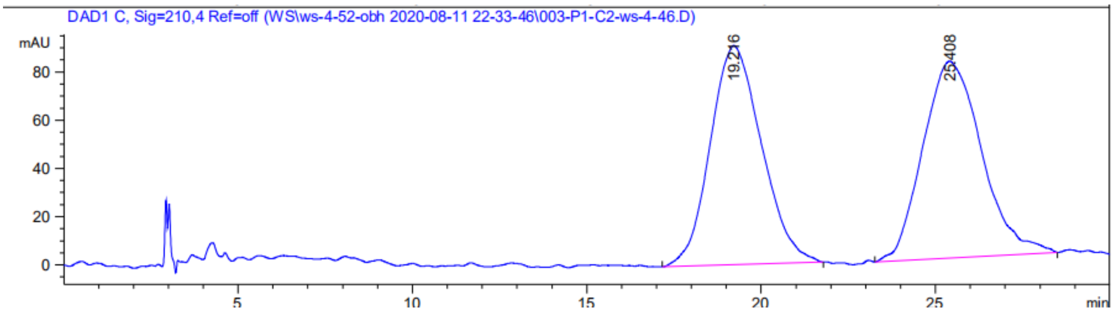

Peak #	RetTime [min]	Type	Width [min]	Area [mAU*s]	Height [mAU]	Area %
1	19.216	BB	1.3873	9298.86719	90.91682	49.2960
2	25.408	BB	1.5249	9564.47559	81.63287	50.7040

HPLC data using (1*S*, 2*R*)-**L41**

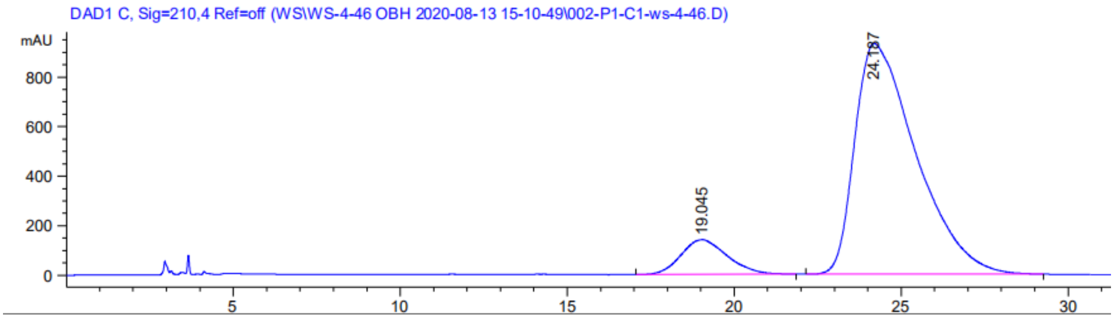

Signal 2: DAD1 C, Sig=210,4 Ref=off

Peak #	RetTime [min]	Type	Width [min]	Area [mAU*s]	Height [mAU]	Area %
1	19.045	BB	1.3915	1.34477e4	140.14211	10.1610
2	24.187	BB	1.7549	1.18899e5	931.66510	89.8390

Supplementary Figure 193 HPLC spectra for **4v**

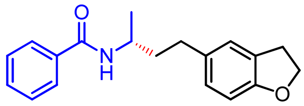

5a

HPLC data using *rac*-**L41**

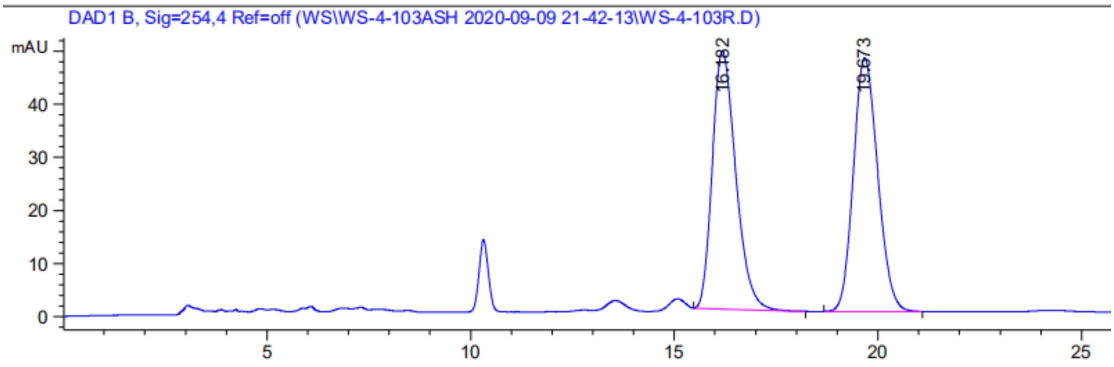

Signal 1: DAD1 B, Sig=254,4 Ref=off

Peak #	RetTime [min]	Type	Width [min]	Area [mAU*s]	Height [mAU]	Area %
1	16.182	BB	0.6145	1943.34436	48.60138	49.5143
2	19.673	BB	0.6320	1981.47253	47.75810	50.4857

HPLC data using (1*S*, 2*R*)-**L41**

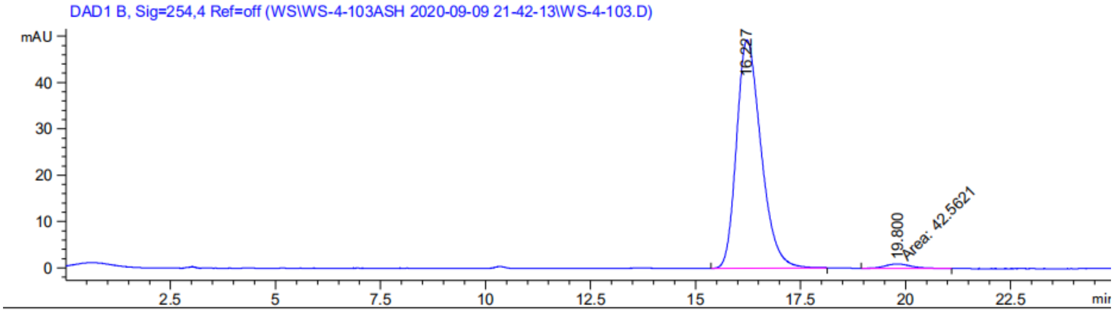

Signal 1: DAD1 B, Sig=254,4 Ref=off

Peak #	RetTime [min]	Type	Width [min]	Area [mAU*s]	Height [mAU]	Area %
1	16.227	BB	0.6217	1999.33875	49.24481	97.9156
2	19.800	MM	0.7344	42.56210	9.65926e-1	2.0844

Supplementary Figure 194 HPLC spectra for **5a**

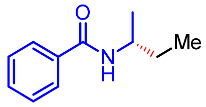

5b

HPLC data using *rac*-**L41**

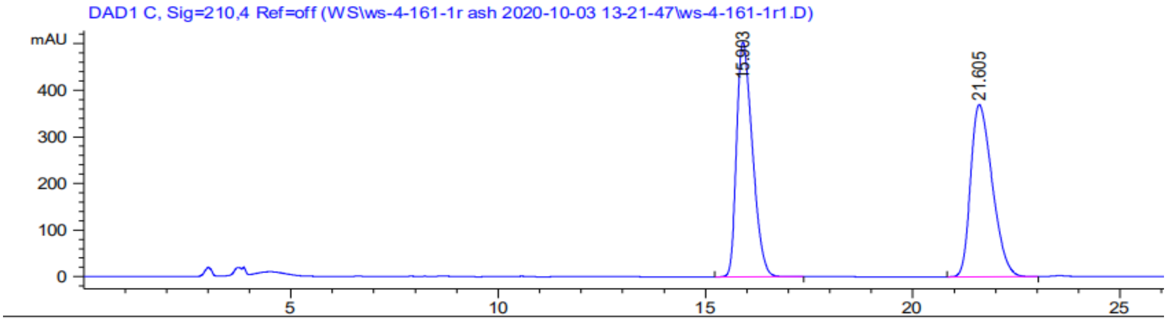

Signal 2: DAD1 C, Sig=210,4 Ref=off

Peak #	RetTime [min]	Type	Width [min]	Area [mAU*s]	Height [mAU]	Area %
1	15.903	BB	0.4141	1.33816e4	503.39813	49.9719
2	21.605	BB	0.5649	1.33966e4	369.59964	50.0281

HPLC data using (1*S*, 2*R*)-**L41**

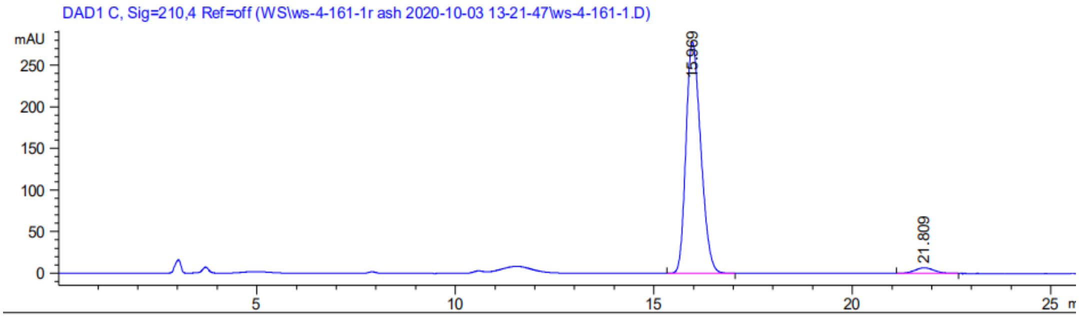

Signal 2: DAD1 C, Sig=210,4 Ref=off

Peak #	RetTime [min]	Type	Width [min]	Area [mAU*s]	Height [mAU]	Area %
1	15.969	BB	0.4043	7207.59229	278.15912	96.8920
2	21.809	BB	0.4820	231.20079	6.81149	3.1080

Supplementary Figure 195 HPLC spectra for **5b**

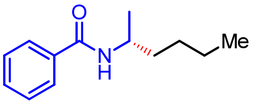

5c

HPLC data using *rac*-**L41**

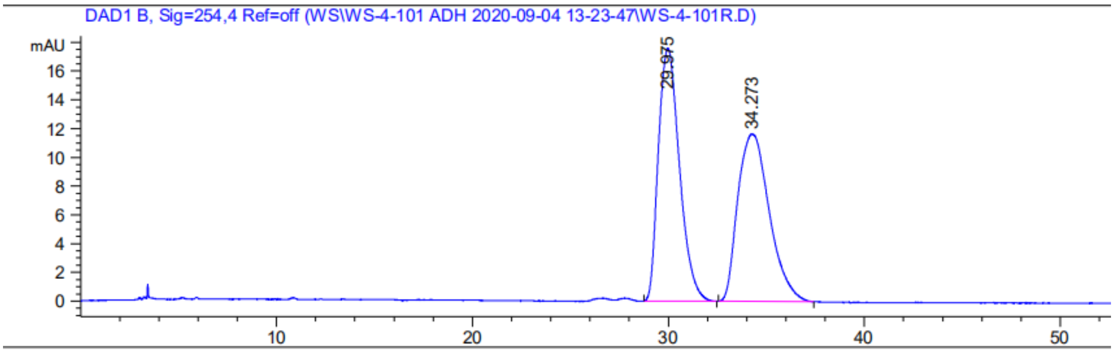

Signal 2: DAD1 C, Sig=210,4 Ref=off

Peak #	RetTime [min]	Type	Width [min]	Area [mAU*s]	Height [mAU]	Area %
1	29.977	BB	1.1872	7311.20215	96.79460	51.0938
2	34.277	BB	1.5082	6998.15967	63.28129	48.9062

HPLC data using (1*S*, 2*R*)-**L41**

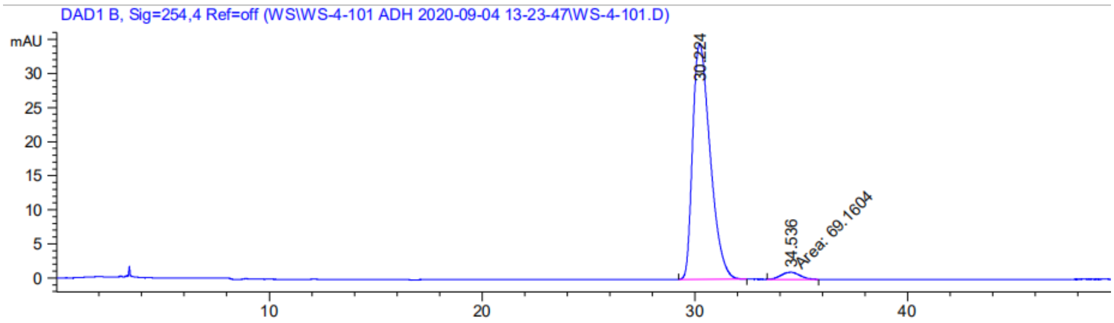

Signal 2: DAD1 C, Sig=210,4 Ref=off

Peak #	RetTime [min]	Type	Width [min]	Area [mAU*s]	Height [mAU]	Area %
1	30.224	BB	0.8879	1.08673e4	189.27000	96.7884
2	34.626	BB	0.7631	360.60077	5.91582	3.2116

Supplementary Figure 196 HPLC spectra for **5c**

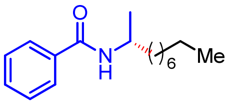

5d

HPLC data using *rac*-**L41**

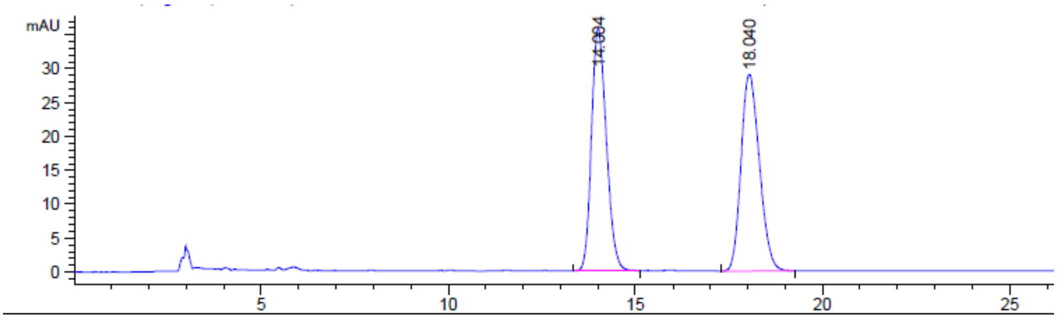

Signal 1: DAD1 B, Sig=254,4 Ref=off

Peak #	RetTime [min]	Type	Width [min]	Area [mAU*s]	Height [mAU]	Area %
1	14.004	BB	0.4272	985.20825	35.78544	49.8646
2	18.040	BB	0.5264	990.55731	29.00645	50.1354

HPLC data using (1*S*, 2*R*)-**L41**

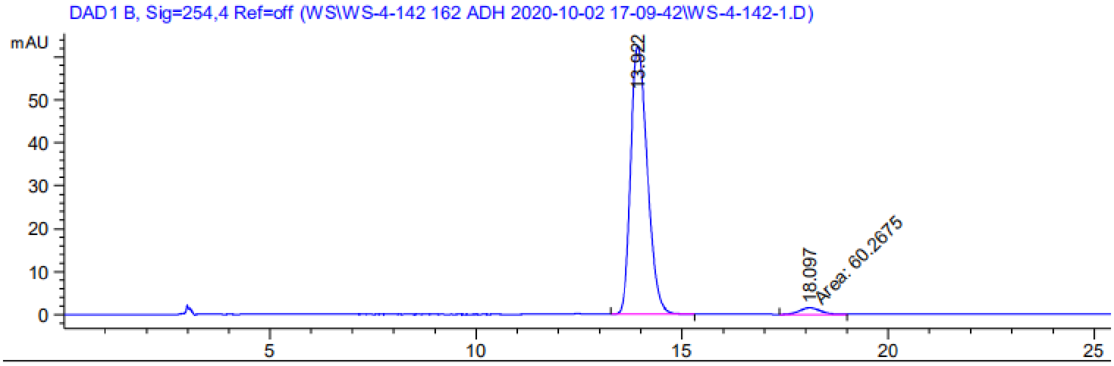

Signal 1: DAD1 B, Sig=254,4 Ref=off

Peak #	RetTime [min]	Type	Width [min]	Area [mAU*s]	Height [mAU]	Area %
1	13.922	BB	0.4367	1746.95288	62.38653	96.6652
2	18.097	MM	0.6379	60.26748	1.57464	3.3348

Supplementary Figure 197 HPLC spectra for **5d**

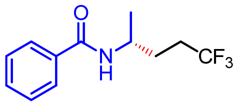

5e

HPLC data using *rac*-**L41**

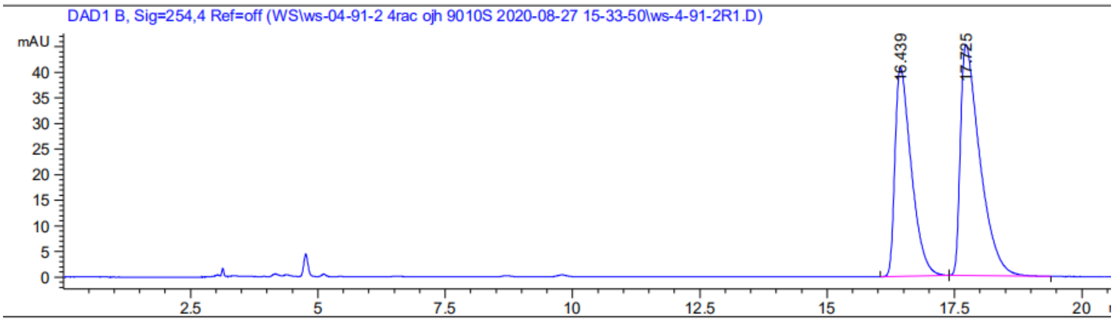

Signal 2: DAD1 C, Sig=210,4 Ref=off

Peak #	RetTime [min]	Type	Width [min]	Area [mAU*s]	Height [mAU]	Area %
1	16.439	BV	0.3382	4761.71582	211.86380	42.9387
2	17.725	VB	0.3981	6327.86230	235.07181	57.0613

HPLC data using (1*S*, 2*R*)-**L41**

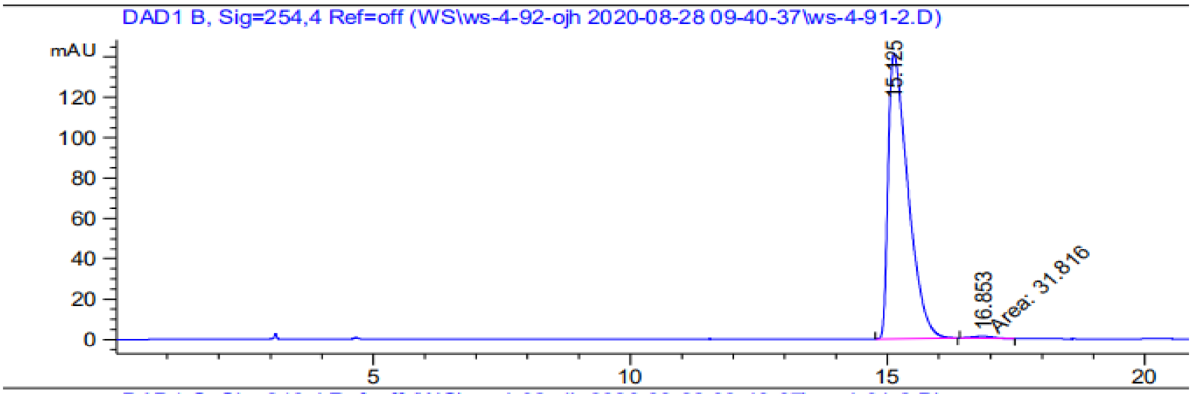

Signal 2: DAD1 C, Sig=210,4 Ref=off

Peak #	RetTime [min]	Type	Width [min]	Area [mAU*s]	Height [mAU]	Area %
1	15.125	BB	0.4014	1.93352e4	724.44727	99.1831
2	16.834	BB	0.4255	159.25655	5.31581	0.8169

Supplementary Figure 198 HPLC spectra for **5e**

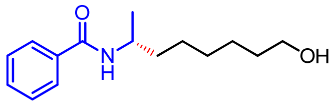

5f

HPLC data using *rac*-**L41**

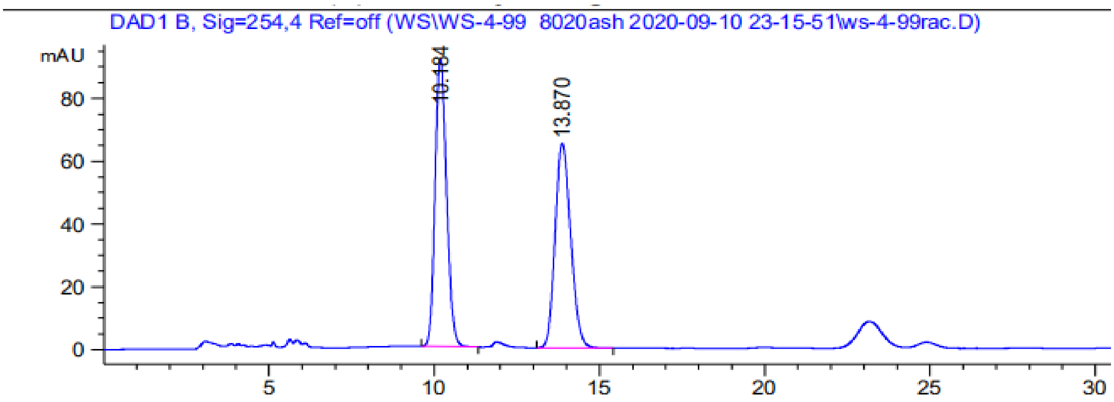

Signal 1: DAD1 B, Sig=254,4 Ref=off

Peak #	RetTime [min]	Type	Width [min]	Area [mAU*s]	Height [mAU]	Area %
1	10.184	BB	0.3739	2195.90479	91.51944	50.6886
2	13.870	BB	0.5130	2136.23877	65.07289	49.3114

HPLC data using (1*S*, 2*R*)-**L41**

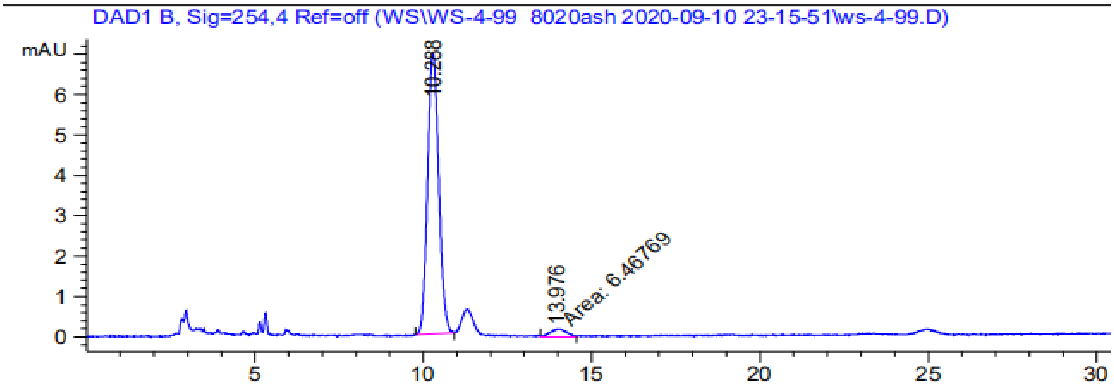

Signal 1: DAD1 B, Sig=254,4 Ref=off

Peak #	RetTime [min]	Type	Width [min]	Area [mAU*s]	Height [mAU]	Area %
1	10.288	BB	0.3574	159.32875	6.95129	96.0990
2	13.976	MM	0.5603	6.46769	1.92372e-1	3.9010

Supplementary Figure 199 HPLC spectra for **5f**

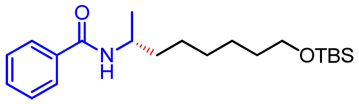

5g

HPLC data using *rac*-**L41**

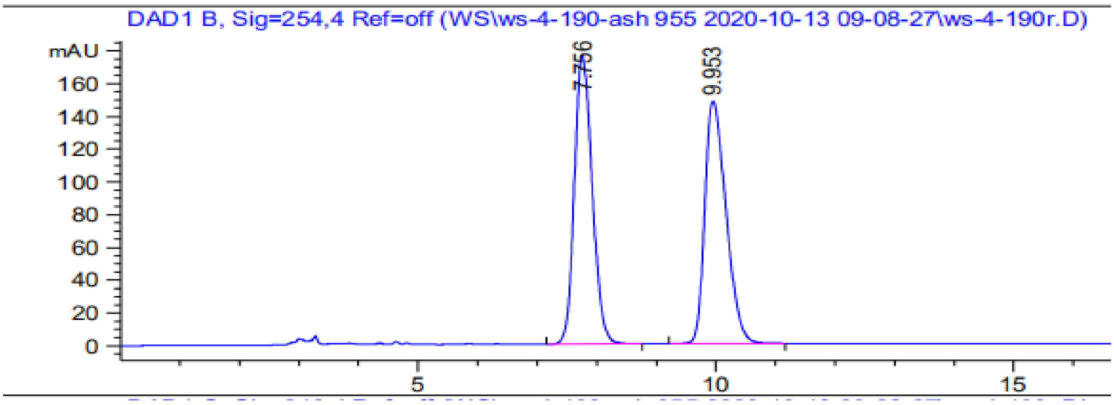

Signal 1: DAD1 B, Sig=254,4 Ref=off

Peak #	RetTime [min]	Type	Width [min]	Area [mAU*s]	Height [mAU]	Area %
1	7.756	BB	0.3238	3652.94385	176.24637	49.9202
2	9.953	BB	0.3877	3664.62720	147.59634	50.0798

HPLC data using (1*S*, 2*R*)-**L41**

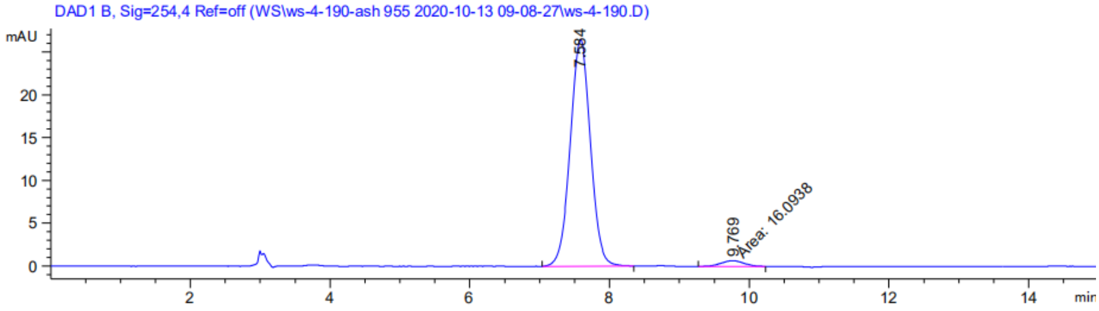

Signal 1: DAD1 B, Sig=254,4 Ref=off

Peak #	RetTime [min]	Type	Width [min]	Area [mAU*s]	Height [mAU]	Area %
1	7.584	BB	0.3109	532.78992	26.46369	97.0679
2	9.769	MM	0.3914	16.09383	6.85277e-1	2.9321

Supplementary Figure 200 HPLC spectra for **5g**

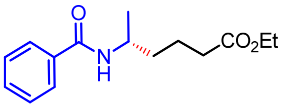

5h

HPLC data using *rac*-**L41**

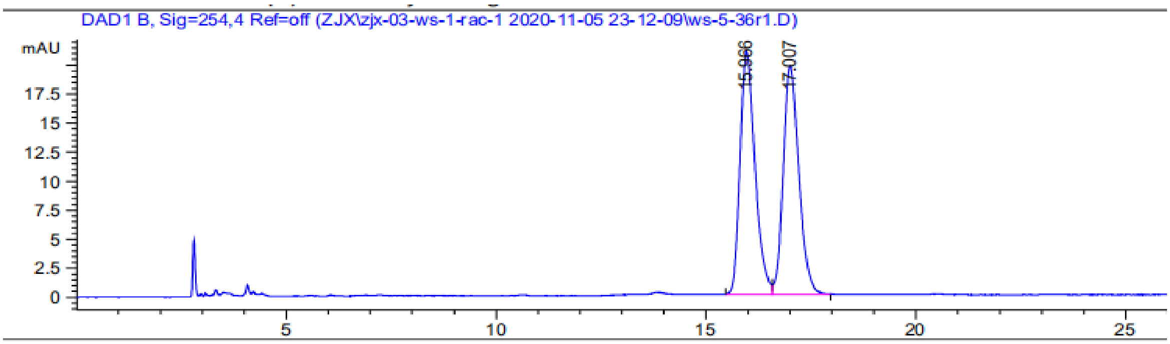

Signal 2: DAD1 C, Sig=210,4 Ref=off

Peak #	RetTime [min]	Type	Width [min]	Area [mAU*s]	Height [mAU]	Area %
1	15.966	BV	0.3770	2159.55518	87.19139	50.3951
2	17.006	VB	0.3961	2125.69629	82.08575	49.6049

HPLC data using (1*S*, 2*R*)-**L41**

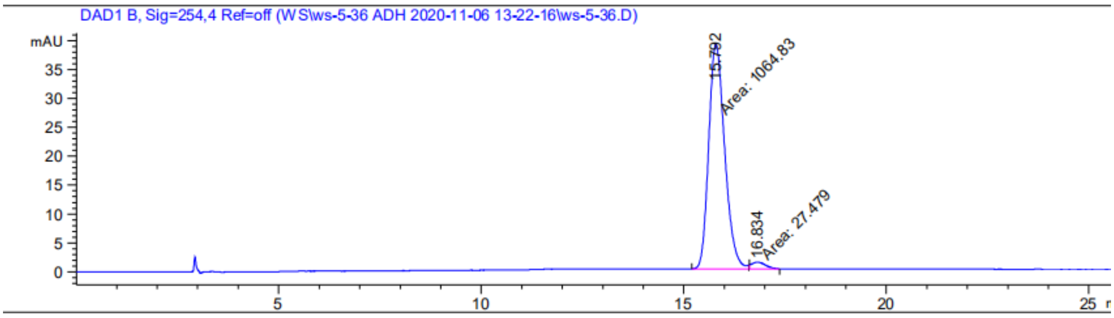

Signal 2: DAD1 C, Sig=210,4 Ref=off

Peak #	RetTime [min]	Type	Width [min]	Area [mAU*s]	Height [mAU]	Area %
1	15.792	MM	0.4524	4373.87598	161.13235	96.8327
2	16.830	MM	0.4891	143.06313	4.87486	3.1673

Supplementary Figure 201 HPLC spectra for **5h**

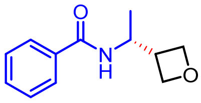

5i

HPLC data using *rac*-**L41**

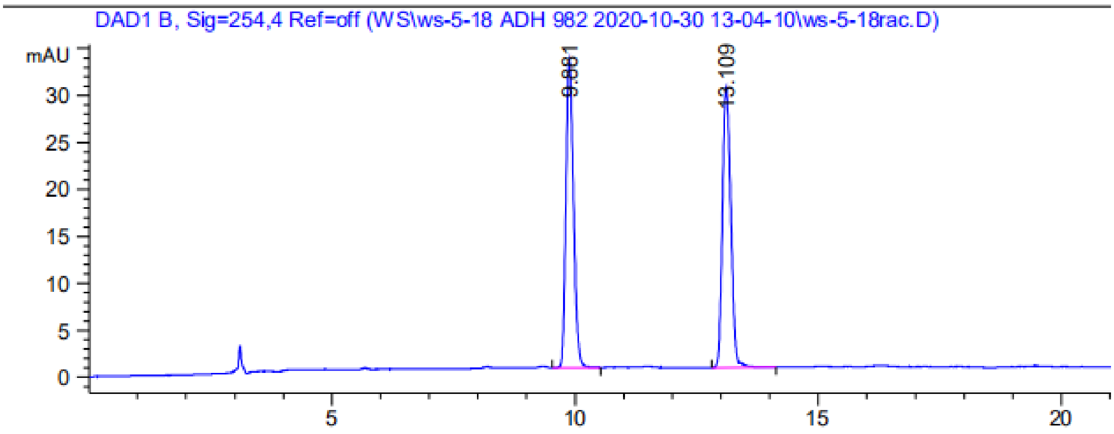

Signal 1: DAD1 B, Sig=254,4 Ref=off

Peak #	RetTime [min]	Type	Width [min]	Area [mAU*s]	Height [mAU]	Area %
1	9.881	BB	0.1645	349.79935	32.78701	49.9216
2	13.109	BB	0.1833	350.89832	29.81392	50.0784

HPLC data using (1*S*, 2*R*)-**L41**

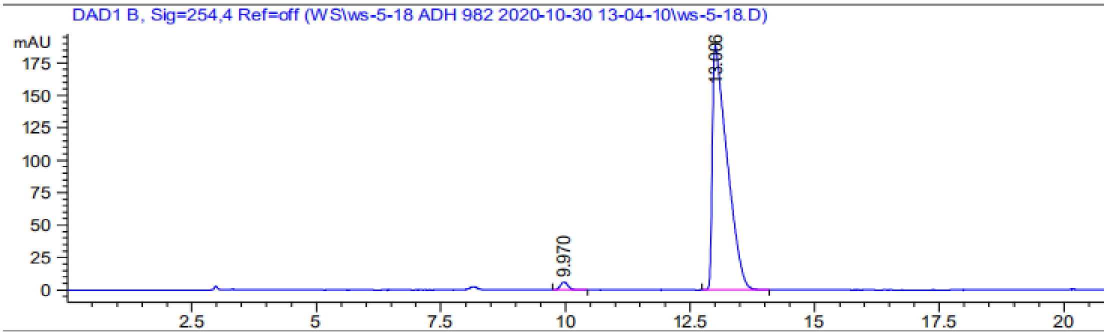

Signal 1: DAD1 B, Sig=254,4 Ref=off

Peak #	RetTime [min]	Type	Width [min]	Area [mAU*s]	Height [mAU]	Area %
1	9.970	BB	0.1590	63.86474	6.15614	1.6173
2	13.006	BB	0.2929	3884.94702	188.15816	98.3827

Supplementary Figure 202 HPLC spectra for **5i**

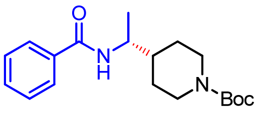

5j

HPLC data using *rac*-**L41**

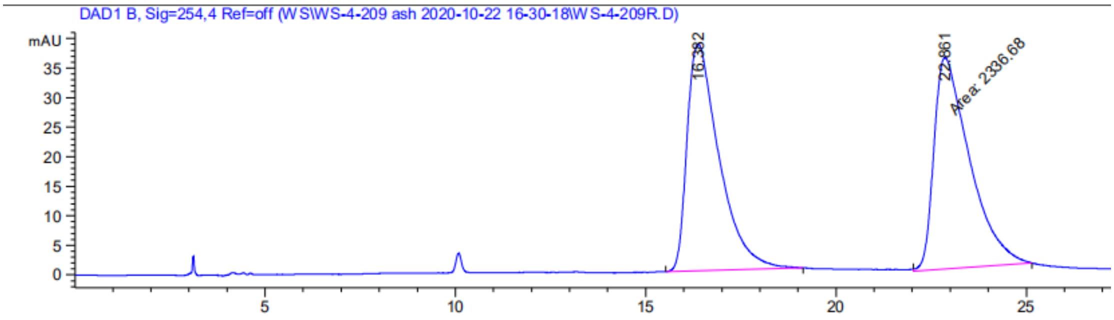

Signal 1: DAD1 B, Sig=254,4 Ref=off

Peak #	RetTime [min]	Type	Width [min]	Area [mAU*s]	Height [mAU]	Area %
1	16.382	BB	0.8465	2254.39160	38.42409	49.1038
2	22.861	MM	1.0871	2336.68237	35.82564	50.8962

HPLC data using (1*S*, 2*R*)-**L41**

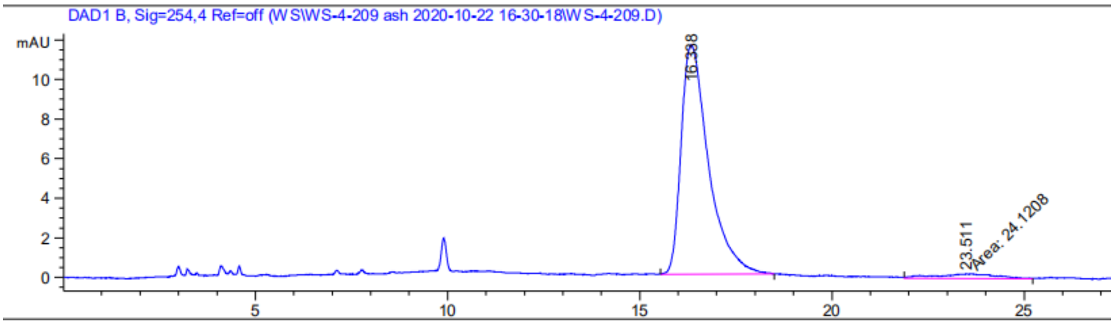

Signal 1: DAD1 B, Sig=254,4 Ref=off

Peak #	RetTime [min]	Type	Width [min]	Area [mAU*s]	Height [mAU]	Area %
1	16.338	BB	0.7152	573.97437	11.56221	95.9671
2	23.511	MM	1.7776	24.12084	2.26153e-1	4.0329

Supplementary Figure 203 HPLC spectra for **5j**

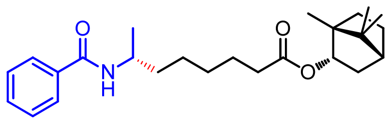

5k

HPLC data using *rac*-**L41**

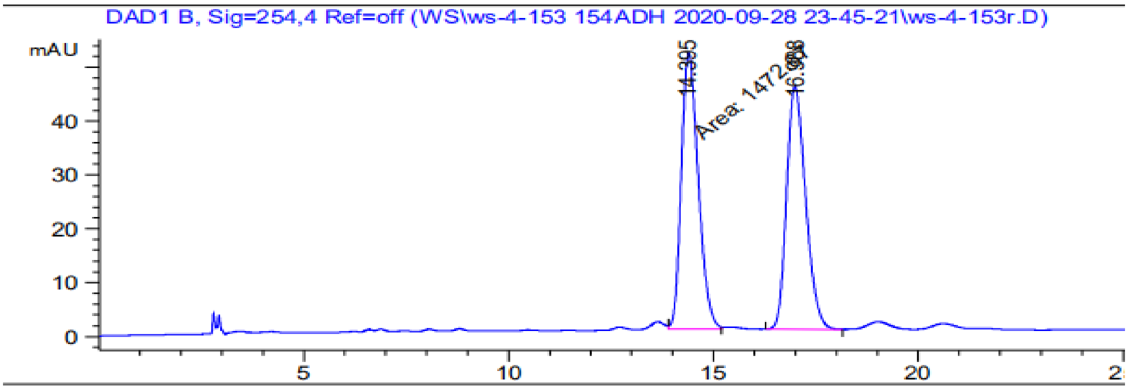

Signal 2: DAD1 C, Sig=210,4 Ref=off

Peak #	RetTime [min]	Type	Width [min]	Area [mAU*s]	Height [mAU]	Area %
1	14.395	VB R	0.4444	6465.11084	223.30618	50.4968
2	16.988	BB	0.5013	6337.90576	195.98576	49.5032

HPLC data using (1*S*, 2*R*)-**L41**

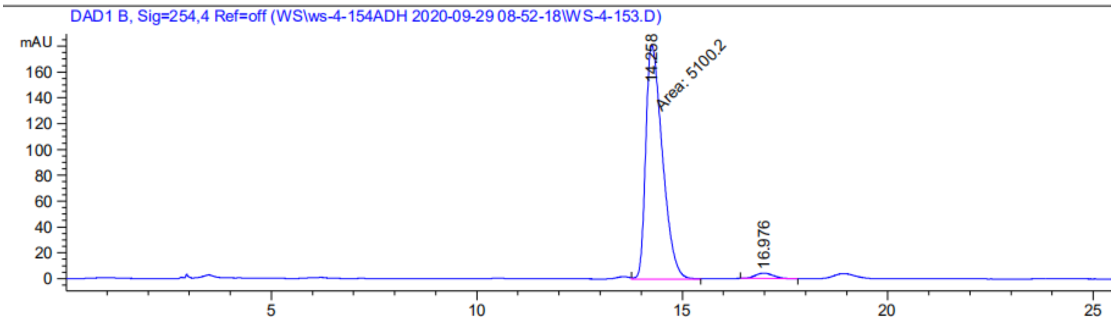

Signal 2: DAD1 C, Sig=210,4 Ref=off

Peak #	RetTime [min]	Type	Width [min]	Area [mAU*s]	Height [mAU]	Area %
1	14.262	MM	0.4734	2.26557e4	797.57513	97.4540
2	16.981	BB	0.4860	591.87872	18.96924	2.5460

Supplementary Figure 204 HPLC spectra for **5k**

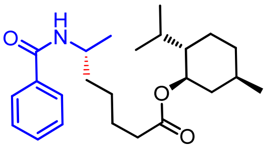

5l

HPLC data using *rac*-**L41**

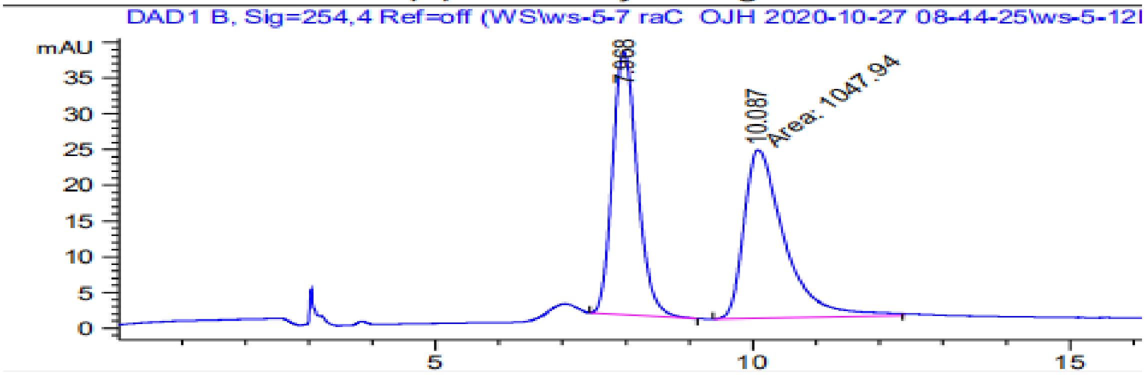

Signal 1: DAD1 B, Sig=254,4 Ref=off

Peak #	RetTime [min]	Type	Width [min]	Area [mAU*s]	Height [mAU]	Area %
1	7.968	BB	0.4232	1001.21869	36.82213	48.8600
2	10.087	MM	0.7407	1047.94153	23.57918	51.1400

HPLC data using (1*S*, 2*R*)-**L41**

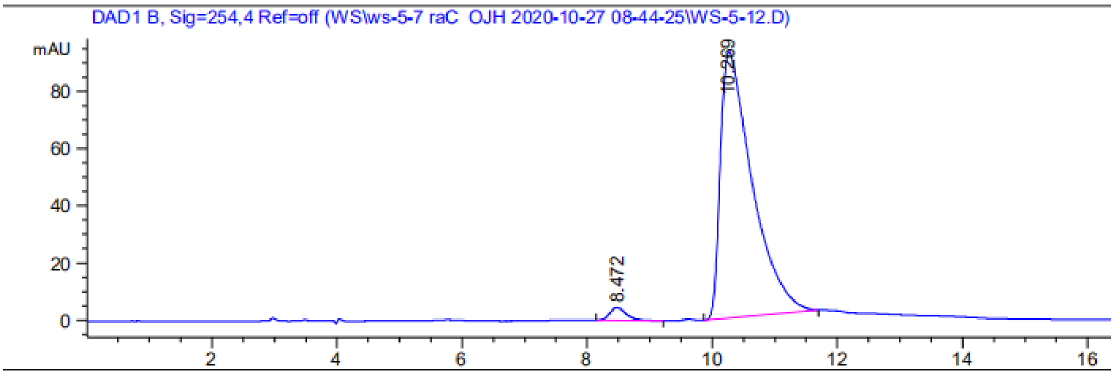

Signal 1: DAD1 B, Sig=254,4 Ref=off

Peak #	RetTime [min]	Type	Width [min]	Area [mAU*s]	Height [mAU]	Area %
1	8.472	BB	0.2776	82.29990	4.49610	2.4379
2	10.269	BB	0.5360	3293.58105	93.27233	97.5621

Supplementary Figure 205 HPLC spectra for **5l**

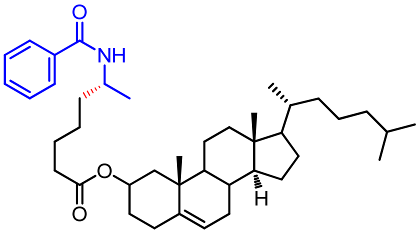

5m

HPLC data using *rac*-**L41**

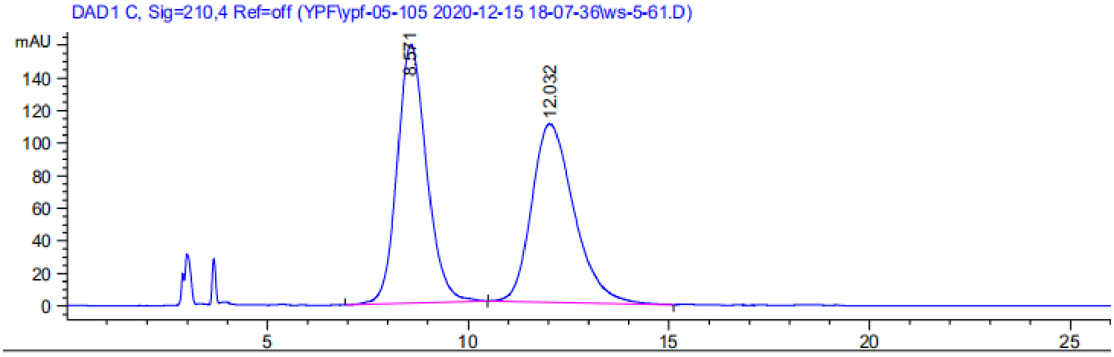

Signal 2: DAD1 C, Sig=210,4 Ref=off

Peak #	RetTime [min]	Type	Width [min]	Area [mAU*s]	Height [mAU]	Area %
1	8.571	BB	0.7838	8102.63184	158.64911	50.2821
2	12.032	BB	1.0861	8011.72900	109.43575	49.7179

HPLC data using (1*S*, 2*R*)-**L41**

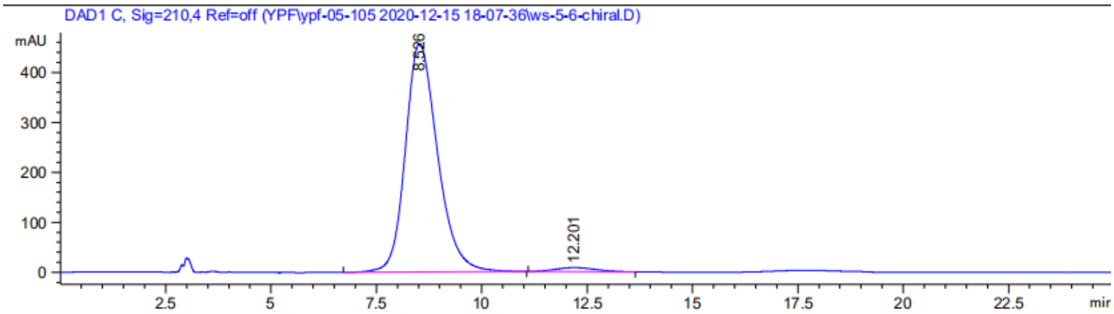

Signal 2: DAD1 C, Sig=210,4 Ref=off

Peak #	RetTime [min]	Type	Width [min]	Area [mAU*s]	Height [mAU]	Area %
1	8.526	BB	0.8054	2.43212e4	456.65717	97.8597
2	12.201	BB	0.7732	531.92609	8.08928	2.1403

Supplementary Figure 206 HPLC spectra for **5m**

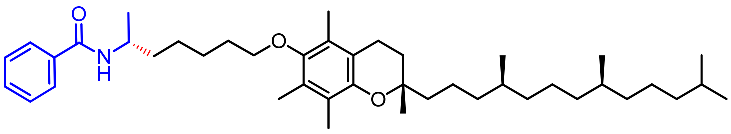

5n

HPLC data using *rac*-**L41**

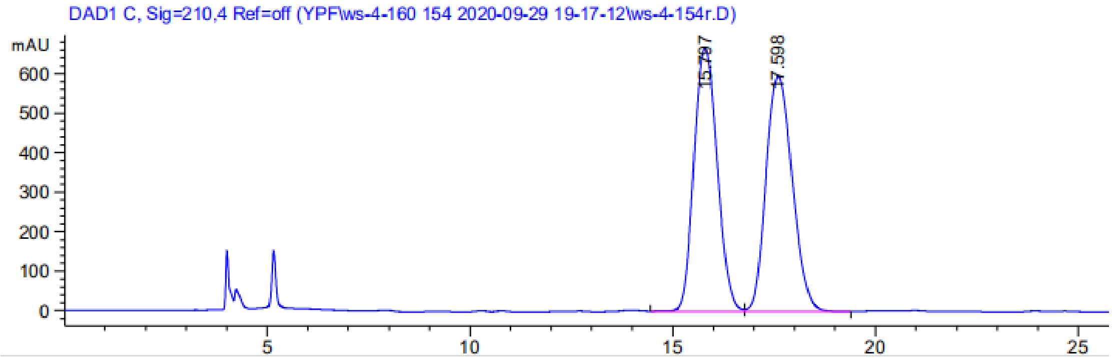

Signal 2: DAD1 C, Sig=210,4 Ref=off

Peak #	RetTime [min]	Type	Width [min]	Area [mAU*s]	Height [mAU]	Area %
1	15.797	BV	0.6373	2.66135e4	667.33929	49.6939
2	17.598	VB	0.7194	2.69413e4	595.93646	50.3061

HPLC data using (1*S*, 2*R*)-**L41**

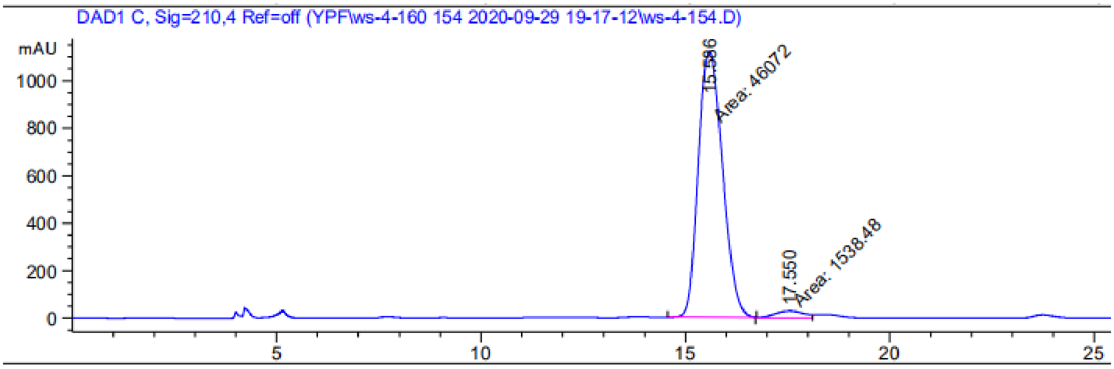

Signal 2: DAD1 C, Sig=210,4 Ref=off

Peak #	RetTime [min]	Type	Width [min]	Area [mAU*s]	Height [mAU]	Area %
1	15.586	MM	0.6862	4.60720e4	1119.00928	96.7686
2	17.550	MM	0.8440	1538.47949	30.38098	3.2314

Supplementary Figure 207 HPLC spectra for **5n**

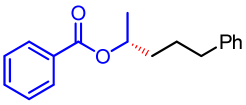

6a

HPLC data using *rac*-**L41**

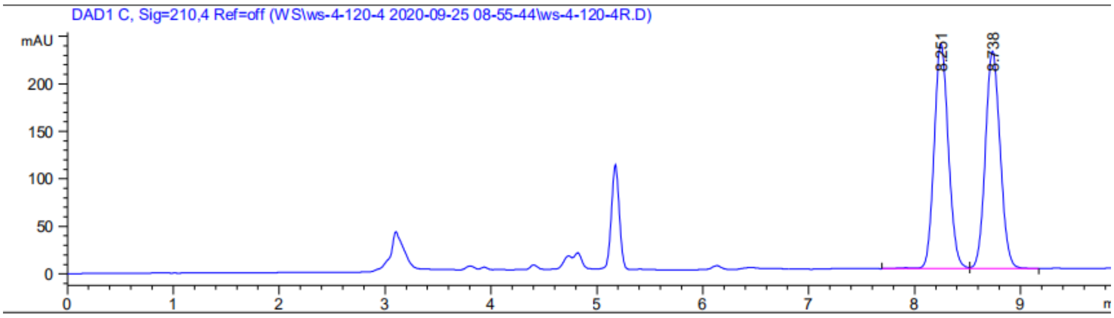

Signal 2: DAD1 C, Sig=210,4 Ref=off

Peak #	RetTime [min]	Type	Width [min]	Area [mAU*s]	Height [mAU]	Area %
1	8.251	VV R	0.1450	2213.07593	236.78926	49.9689
2	8.738	VB	0.1510	2215.83154	228.70476	50.0311

HPLC data using (1*S*, 2*R*)-**L41**

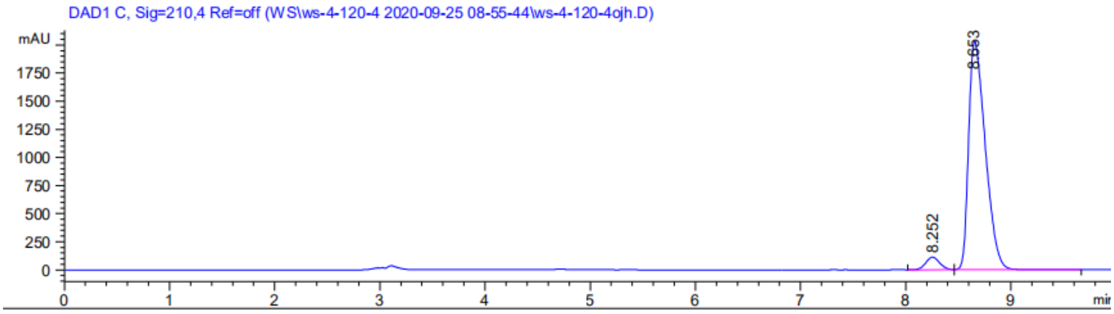

Signal 2: DAD1 C, Sig=210,4 Ref=off

Peak #	RetTime [min]	Type	Width [min]	Area [mAU*s]	Height [mAU]	Area %
1	8.252	BV	0.1448	1047.33826	112.34211	4.4822
2	8.653	VB	0.1701	2.23193e4	2032.77490	95.5178

Supplementary Figure 208 HPLC spectra for **6a**

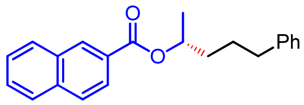

6b

HPLC data using *rac*-**L41**

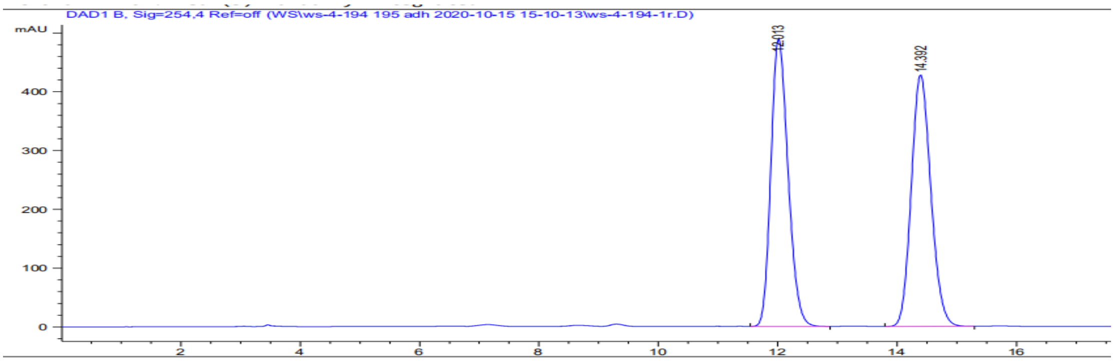

Signal 1: DAD1 B, Sig=254,4 Ref=off

Peak #	RetTime [min]	Type	Width [min]	Area [mAU*s]	Height [mAU]	Area %
1	12.013	BB	0.3091	9759.21777	488.51837	50.0293
2	14.392	BB	0.3540	9747.77246	427.57886	49.9707

HPLC data using (1*S*, 2*R*)-**L41**

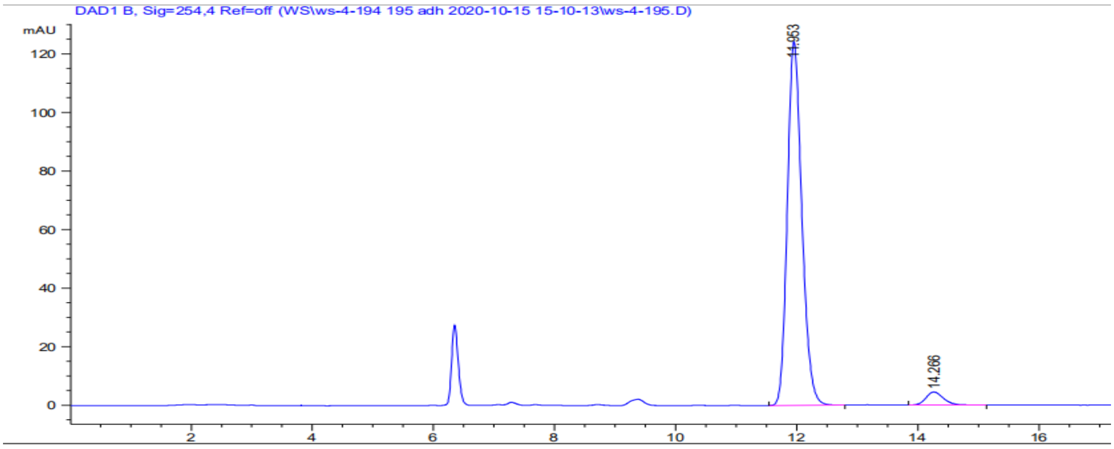

Signal 1: DAD1 B, Sig=254,4 Ref=off

Peak #	RetTime [min]	Type	Width [min]	Area [mAU*s]	Height [mAU]	Area %
1	11.953	BB	0.2564	2068.55371	124.12770	95.8855
2	14.266	BB	0.3013	88.76351	4.43817	4.1145

Supplementary Figure 209 HPLC spectra for **6b**

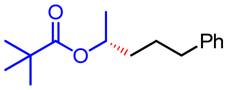

6c

HPLC data using *rac*-**L41**

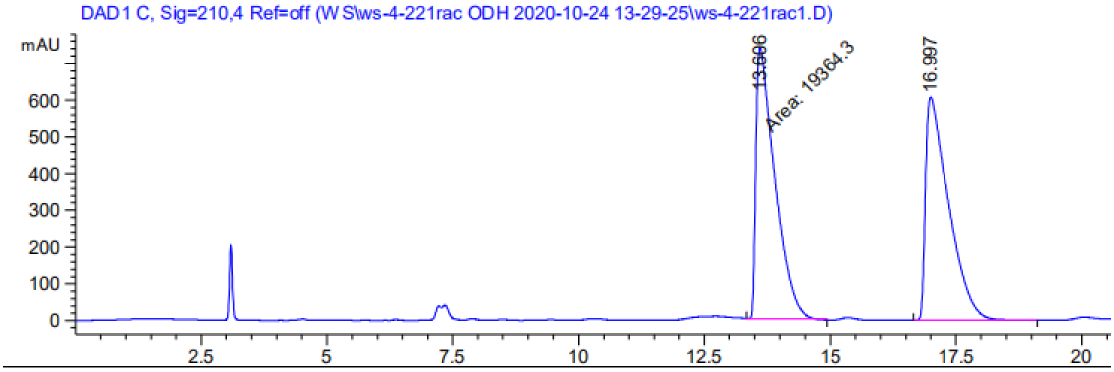

Signal 2: DAD1 C, Sig=210,4 Ref=off

Peak #	RetTime [min]	Type	Width [min]	Area [mAU*s]	Height [mAU]	Area %
1	13.606	MM	0.4368	1.93643e4	738.82959	49.8335
2	16.997	BB	0.4663	1.94936e4	607.65295	50.1665

HPLC data using (1*S*, 2*R*)-**L41**

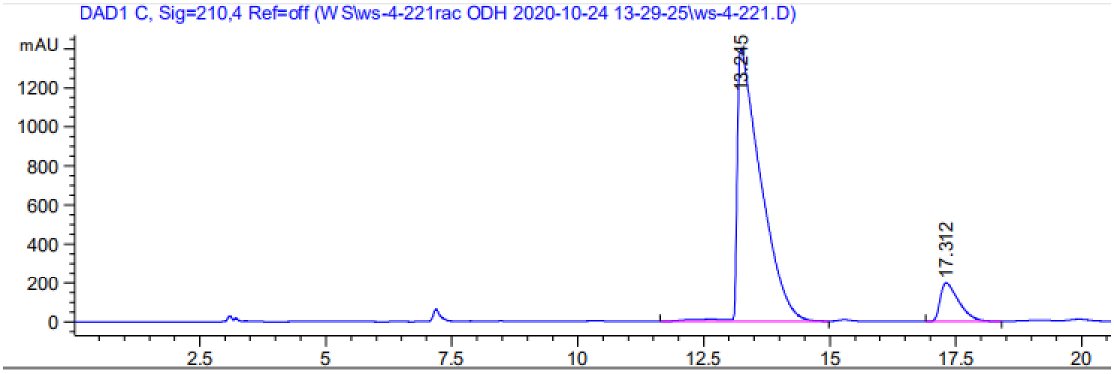

Signal 2: DAD1 C, Sig=210,4 Ref=off

Peak #	RetTime [min]	Type	Width [min]	Area [mAU*s]	Height [mAU]	Area %
1	13.245	VB R	0.4550	4.56656e4	1400.86584	90.0646
2	17.312	BB	0.3846	5037.58398	196.85522	9.9354

Supplementary Figure 210 HPLC spectra for **6c**

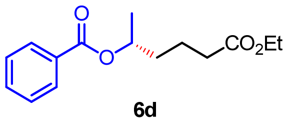

6d

HPLC data using *rac*-**L41**

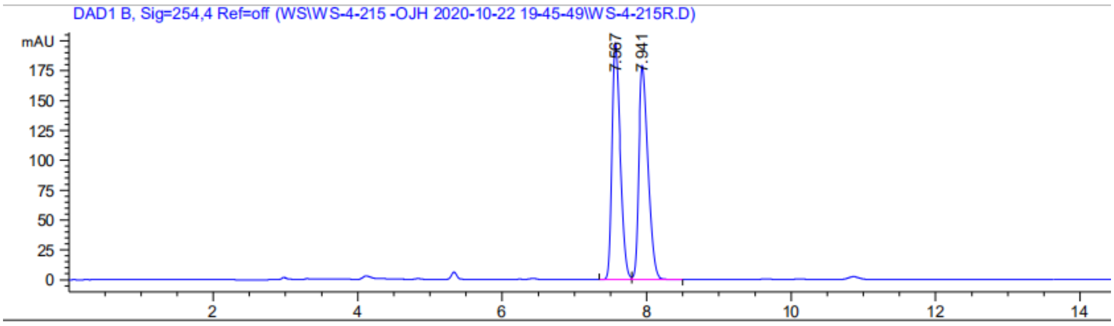

Signal 1: DAD1 B, Sig=254,4 Ref=off

Peak #	RetTime [min]	Type	Width [min]	Area [mAU*s]	Height [mAU]	Area %
1	7.567	BV	0.1256	1581.40112	197.21460	49.8847
2	7.941	VB	0.1378	1588.70935	178.61713	50.1153

HPLC data using (1*S*, 2*R*)-**L41**

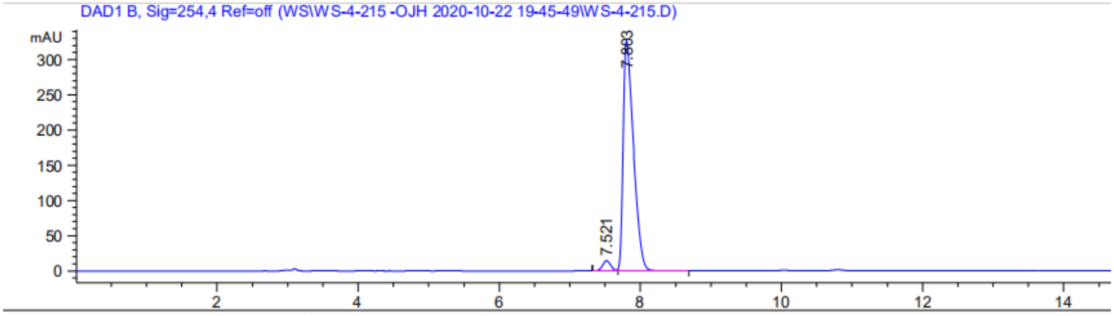

Signal 1: DAD1 B, Sig=254,4 Ref=off

Peak #	RetTime [min]	Type	Width [min]	Area [mAU*s]	Height [mAU]	Area %
1	7.521	BV E	0.1224	111.63396	14.40316	3.2829
2	7.803	VB R	0.1515	3288.85498	326.52649	96.7171

Supplementary Figure 211 HPLC spectra for **6d**

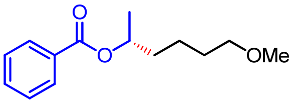

6e

HPLC data using *rac*-**L41**

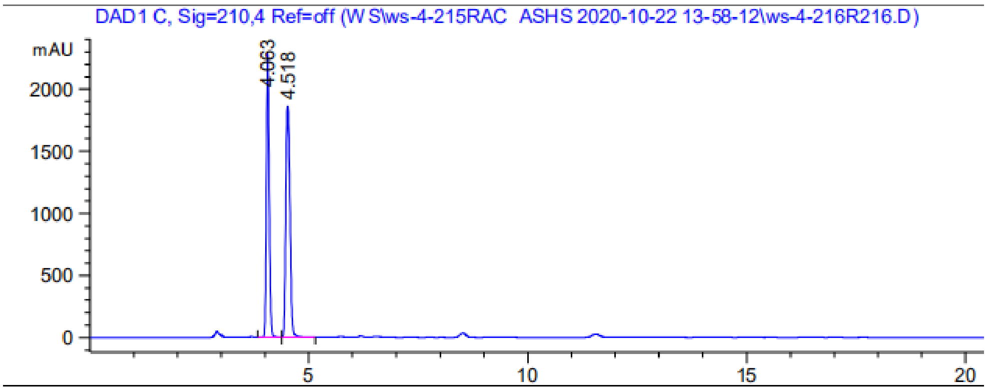

Signal 1: DAD1 B, Sig=254,4 Ref=off

Peak #	RetTime [min]	Type	Width [min]	Area [mAU*s]	Height [mAU]	Area %
1	4.063	VB R	0.0735	1478.21960	310.64825	50.0087
2	4.518	BB	0.0954	1477.70813	241.58676	49.9913

HPLC data using (1*S*, 2*R*)-**L41**

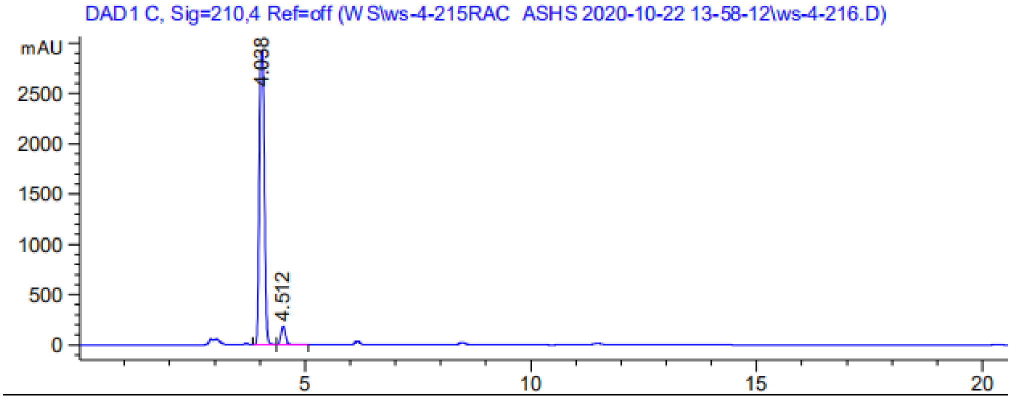

Signal 1: DAD1 B, Sig=254,4 Ref=off

Peak #	RetTime [min]	Type	Width [min]	Area [mAU*s]	Height [mAU]	Area %
1	4.038	BV	0.1010	4097.41211	637.57410	96.3072
2	4.512	VB	0.1056	157.11017	23.03662	3.6928

Supplementary Figure 212 HPLC spectra for **6e**

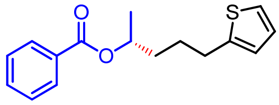

6f

HPLC data using *rac*-**L41**

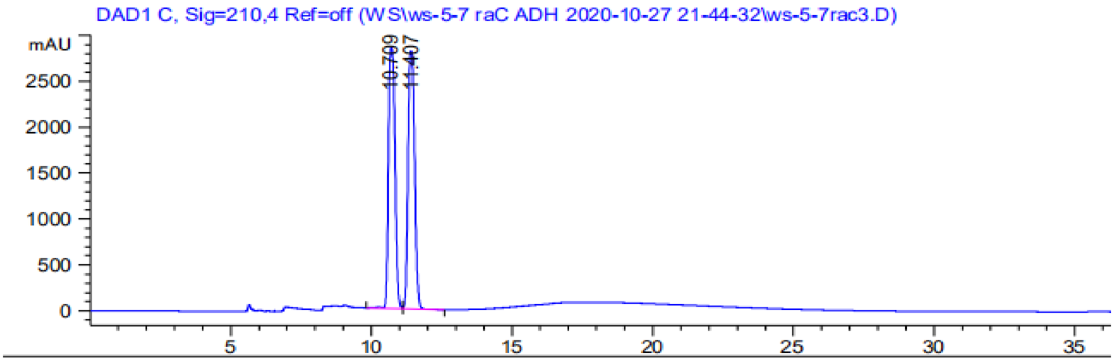

Signal 2: DAD1 C, Sig=210,4 Ref=off

Peak #	RetTime [min]	Type	Width [min]	Area [mAU*s]	Height [mAU]	Area %
1	10.709	VV R	0.2528	4.51964e4	2840.24585	49.5736
2	11.407	VB	0.2606	4.59739e4	2813.40503	50.4264

HPLC data using (1*S*, 2*R*)-**L41**

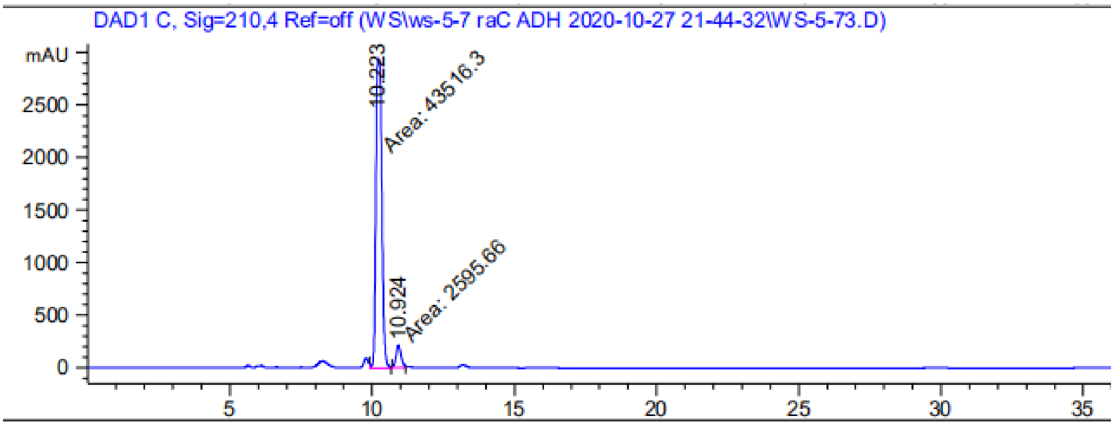

Signal 2: DAD1 C, Sig=210,4 Ref=off

Peak #	RetTime [min]	Type	Width [min]	Area [mAU*s]	Height [mAU]	Area %
1	10.223	MM	0.2466	4.35163e4	2940.49609	94.3710
2	10.924	MM	0.2076	2595.66333	208.43431	5.6290

Supplementary Figure 213 HPLC spectra for **6f**

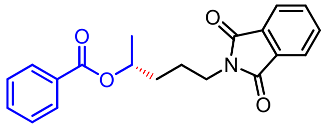

6g

HPLC data using *rac*-**L41**

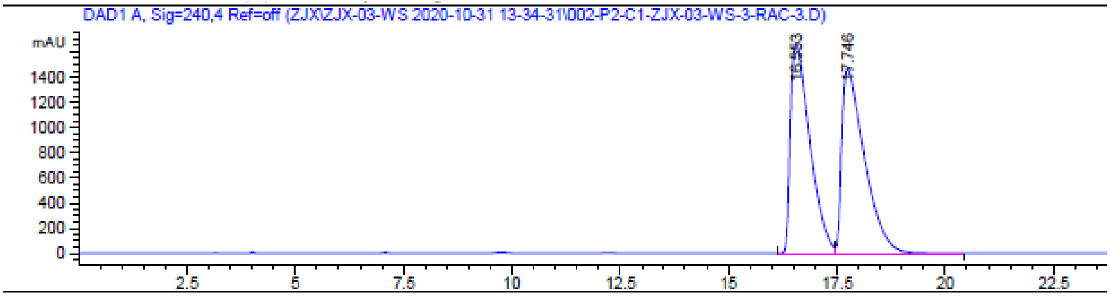

Signal 1: DAD1 A, Sig=240,4 Ref=off

Peak #	RetTime [min]	Type	Width [min]	Area [mAU*s]	Height [mAU]	Area %
1	16.553	BV	0.4733	5.18937e4	1666.46716	49.3326
2	17.746	VB	0.5435	5.32978e4	1461.43628	50.6674

HPLC data using (1*S*, 2*R*)-**L41**

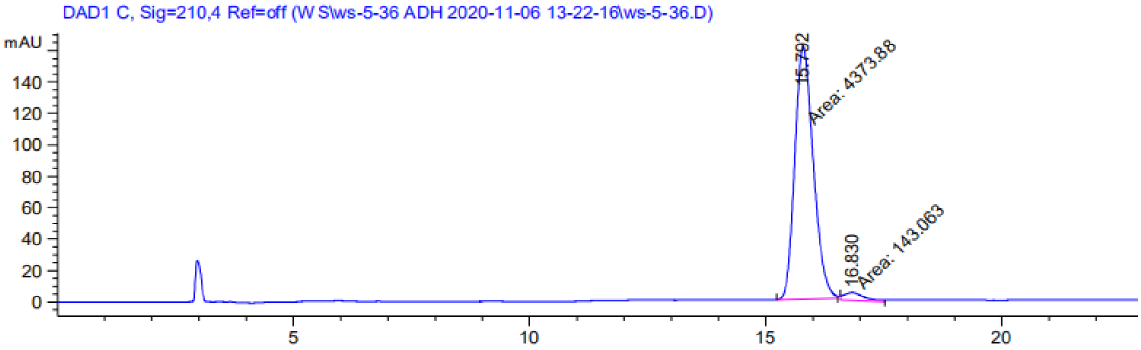

Signal 1: DAD1 A, Sig=240,4 Ref=off

Peak #	RetTime [min]	Type	Width [min]	Area [mAU*s]	Height [mAU]	Area %
1	16.463	BV R	0.4534	3.18415e4	1057.30420	97.5246
2	18.101	VB E	0.4693	808.20422	26.38796	2.4754

Supplementary Figure 214 HPLC spectra for **6g**

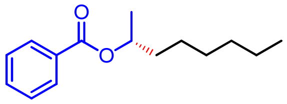

6h

HPLC data using *rac*-**L41**

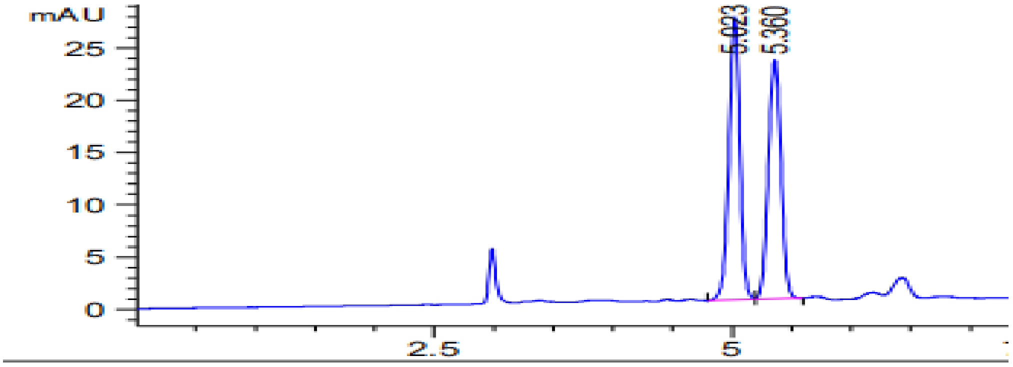

Signal 1: DAD1 B, Sig=254,4 Ref=off

Peak #	RetTime [min]	Type	Width [min]	Area [mAU*s]	Height [mAU]	Area %
1	5.023	BV	0.0996	174.30157	26.89838	50.8293
2	5.360	VB	0.1162	168.61382	22.82166	49.1707

HPLC data using (1*S*, 2*R*)-**L41**

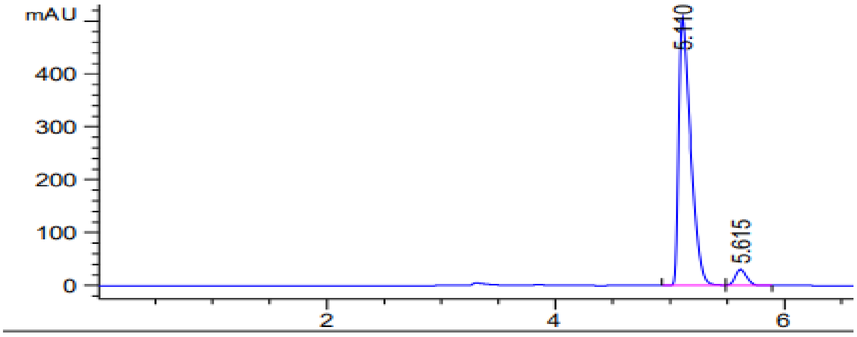

Signal 1: DAD1 B, Sig=254,4 Ref=off

Peak #	RetTime [min]	Type	Width [min]	Area [mAU*s]	Height [mAU]	Area %
1	5.110	BV	0.1086	3673.16821	506.42834	94.7523
2	5.615	VB	0.1057	203.43361	29.78746	5.2477

Supplementary Figure 215 HPLC spectra for **6h**

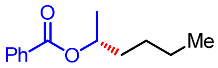

6i

HPLC data using *rac*-**L41**

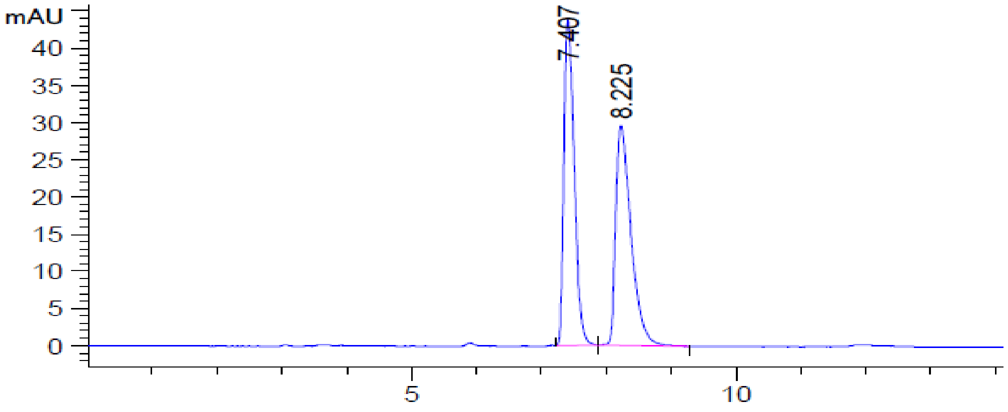

Signal 2: DAD1 C, Sig=210,4 Ref=off

Peak #	RetTime [min]	Type	Width [min]	Area [mAU*s]	Height [mAU]	Area %
1	7.407	VV R	0.1766	4226.16553	366.59167	50.9938
2	8.225	VB	0.2487	4061.44873	245.88205	49.0062

HPLC data using (1*S*, 2*R*)-**L41**

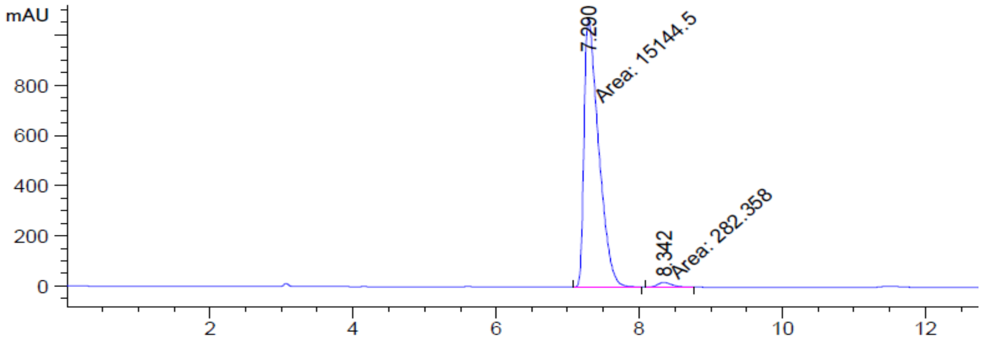

Signal 2: DAD1 C, Sig=210,4 Ref=off

Peak #	RetTime [min]	Type	Width [min]	Area [mAU*s]	Height [mAU]	Area %
1	7.290	MM	0.2360	1.51445e4	1069.36194	98.1697
2	8.342	MM	0.2344	282.35843	20.07823	1.8303

Supplementary Figure 216 HPLC spectra for **6i**

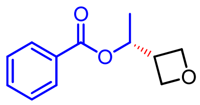

6j

HPLC data using *rac*-**L41**

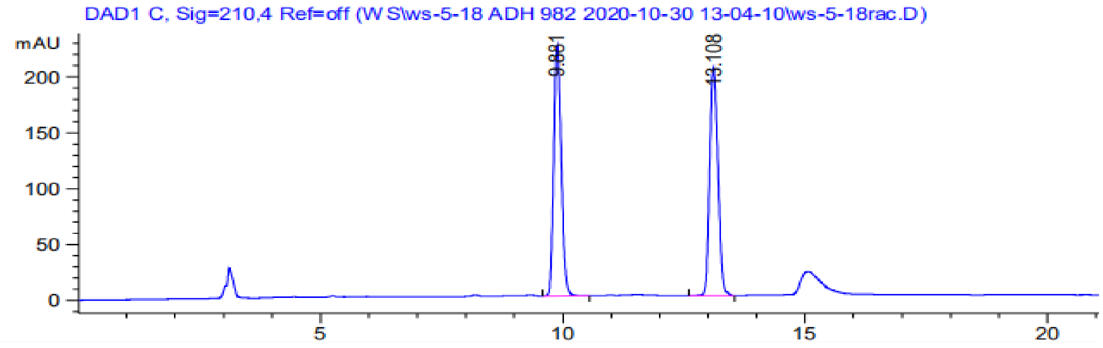

Signal 2: DAD1 C, Sig=210,4 Ref=off

Peak #	RetTime [min]	Type	Width [min]	Area [mAU*s]	Height [mAU]	Area %
1	9.881	BB	0.1637	2370.44067	223.62671	49.9043
2	13.108	BB	0.1822	2379.53613	203.78828	50.0957

HPLC data using (1*S*, 2*R*)-**L41**

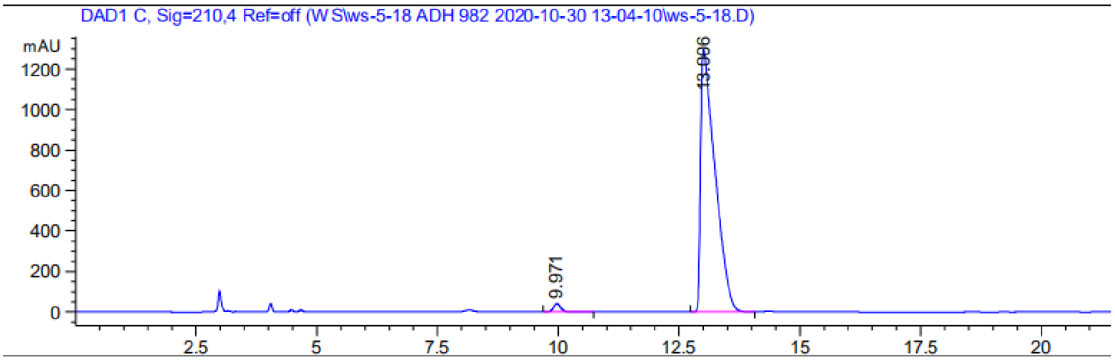

Signal 2: DAD1 C, Sig=210,4 Ref=off

Peak #	RetTime [min]	Type	Width [min]	Area [mAU*s]	Height [mAU]	Area %
1	9.971	BB	0.1622	436.38495	41.67864	1.6045
2	13.006	BB	0.2930	2.67619e4	1295.74292	98.3955

Supplementary Figure 217HPLC spectra for **6j**

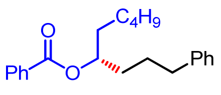

HPLC data using *rac*-**L41**

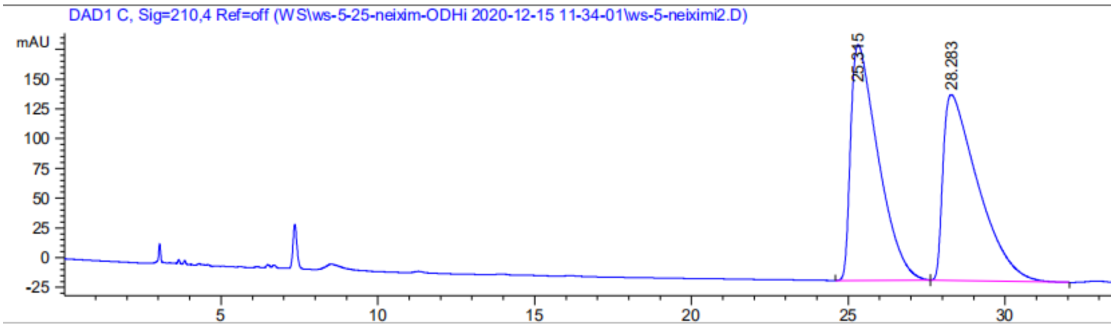

Signal 2: DAD1 C, Sig=210,4 Ref=off

Peak #	RetTime [min]	Type	Width [min]	Area [mAU*s]	Height [mAU]	Area %
1	25.315	BB	0.8693	1.20617e4	197.84622	49.9462
2	28.283	BB	1.1073	1.20877e4	156.11850	50.0538

HPLC data using (1*S*, 2*R*)-**L41**

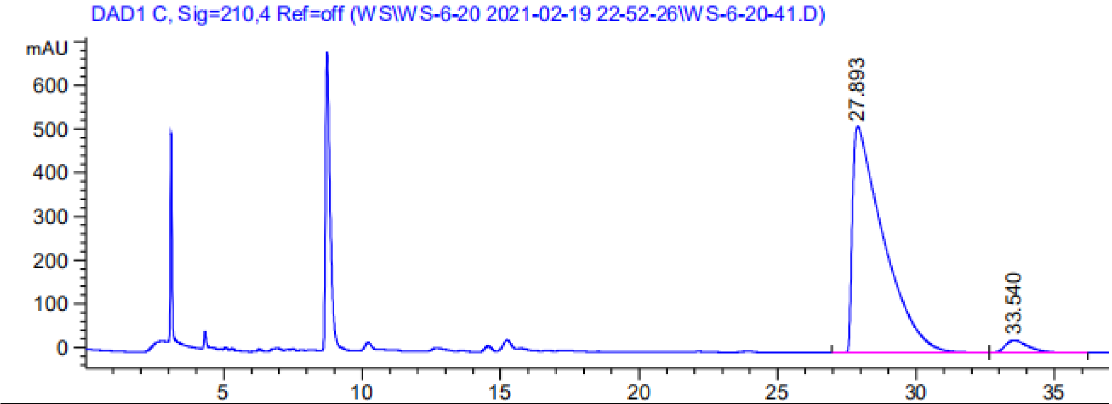

Signal 2: DAD1 C, Sig=210,4 Ref=off

Peak #	RetTime [min]	Type	Width [min]	Area [mAU*s]	Height [mAU]	Area %
1	28.034	BB	1.0839	4.02236e4	513.45953	96.1351
2	33.742	BB	0.8219	1617.09766	26.72605	3.8649

Supplementary Figure 218 HPLC spectra for **6k**

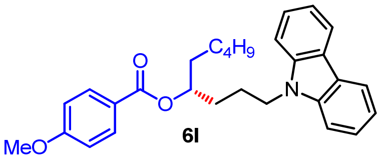

6l

HPLC data using *rac*-**L41**

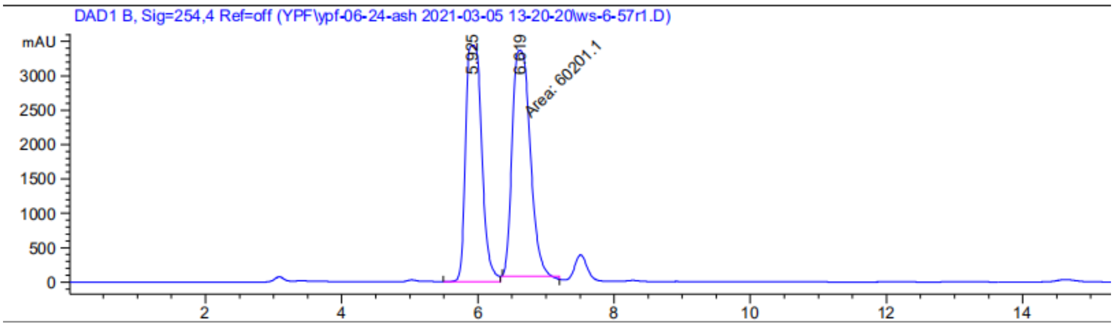

Signal 1: DAD1 B, Sig=254,4 Ref=off

Peak #	RetTime [min]	Type	Width [min]	Area [mAU*s]	Height [mAU]	Area %
1	7.306	BB	0.2185	1.79599e4	1273.77197	50.3851
2	8.736	VB R	0.2902	1.76853e4	942.81458	49.6149

HPLC data using (1*S*, 2*R*)-**L41**

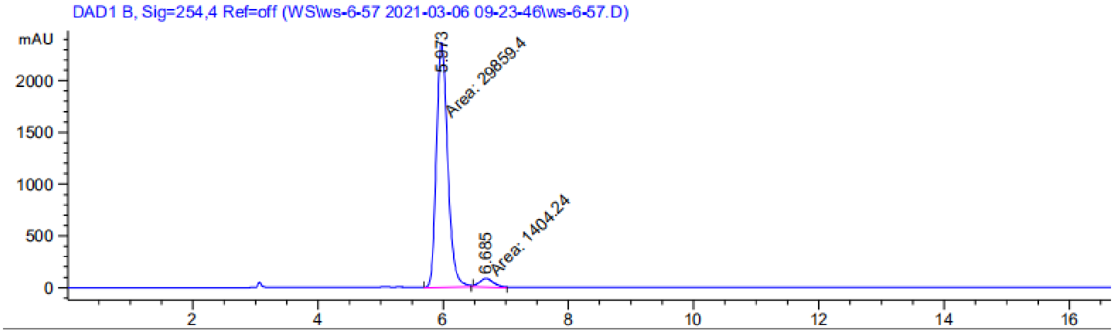

Signal 1: DAD1 B, Sig=254,4 Ref=off

Peak #	RetTime [min]	Type	Width [min]	Area [mAU*s]	Height [mAU]	Area %
1	7.315	BB	0.2045	1.34766e4	1017.90680	97.1241
2	8.828	VB R	0.2668	399.04367	21.91365	2.8759

Supplementary Figure 219 HPLC spectra for **6l**

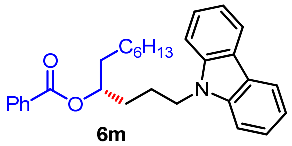

6m

HPLC data using *rac*-**L41**

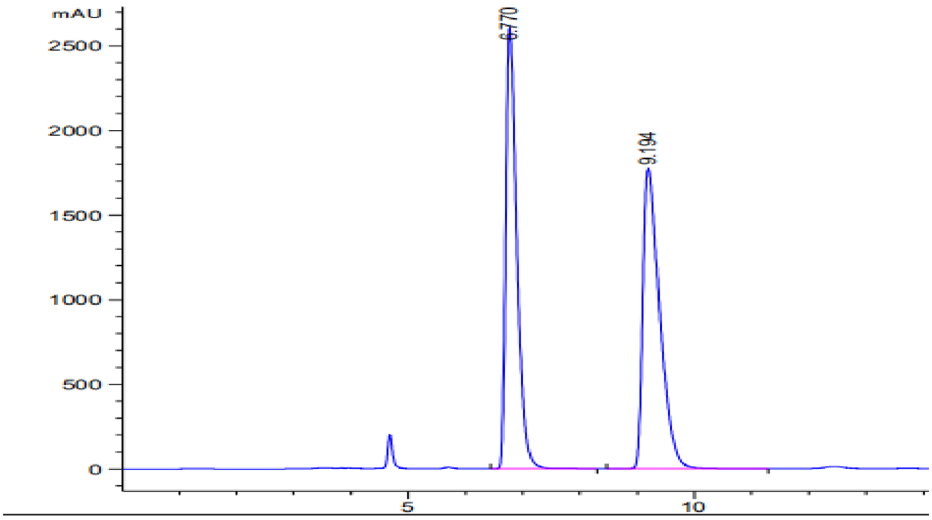

Signal 1: DAD1 B, Sig=254,4 Ref=off

Peak #	RetTime [min]	Type	Width [min]	Area [mAU*s]	Height [mAU]	Area %
1	6.770	BV R	0.2058	3.52517e4	2601.43335	49.1341
2	9.194	BB	0.3103	3.64942e4	1771.46008	50.8659

HPLC data using (1*S*, 2*R*)-**L41**

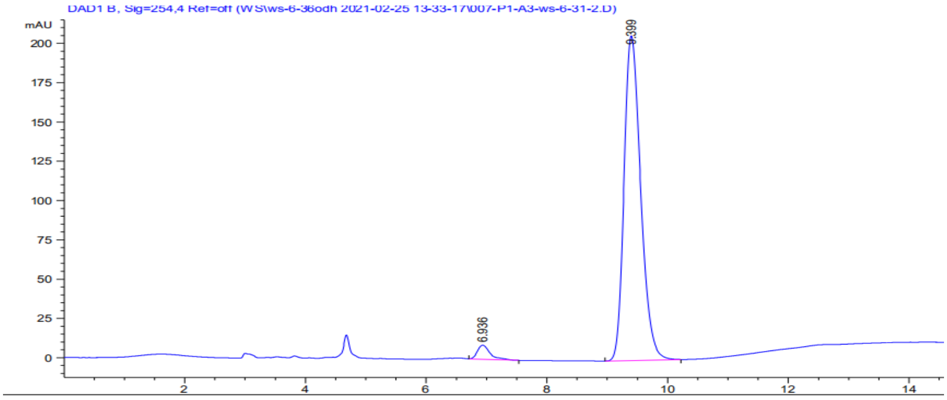

Signal 1: DAD1 B, Sig=254,4 Ref=off

Peak #	RetTime [min]	Type	Width [min]	Area [mAU*s]	Height [mAU]	Area %
1	6.936	BB	0.2198	129.55046	9.00575	3.1806
2	9.399	BB	0.2949	3943.60083	206.45001	96.8194

Supplementary Figure 220 HPLC spectra for **6m**

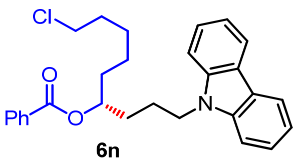

6n

HPLC data using *rac*-**L41**

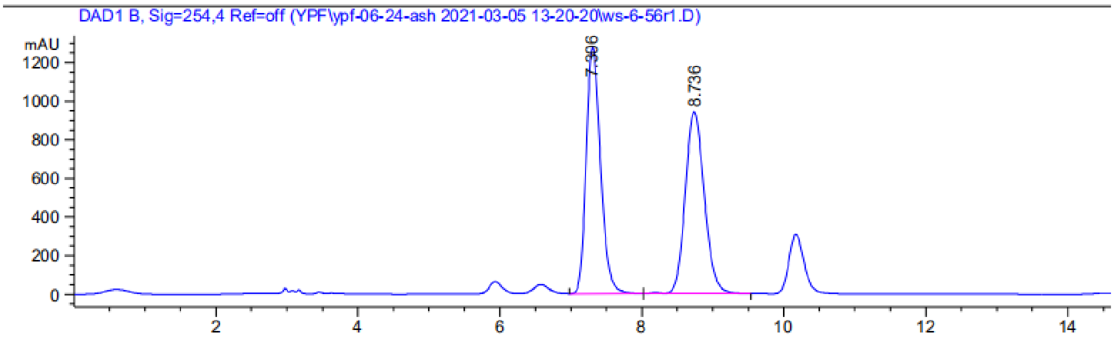

Signal 1: DAD1 B, Sig=254,4 Ref=off

Peak #	RetTime [min]	Type	Width [min]	Area [mAU*s]	Height [mAU]	Area %
1	5.925	BV	0.2597	5.65267e4	3439.31177	48.4261
2	6.619	MM	0.3054	6.02011e4	3285.69385	51.5739

HPLC data using (1*S*, 2*R*)-**L41**

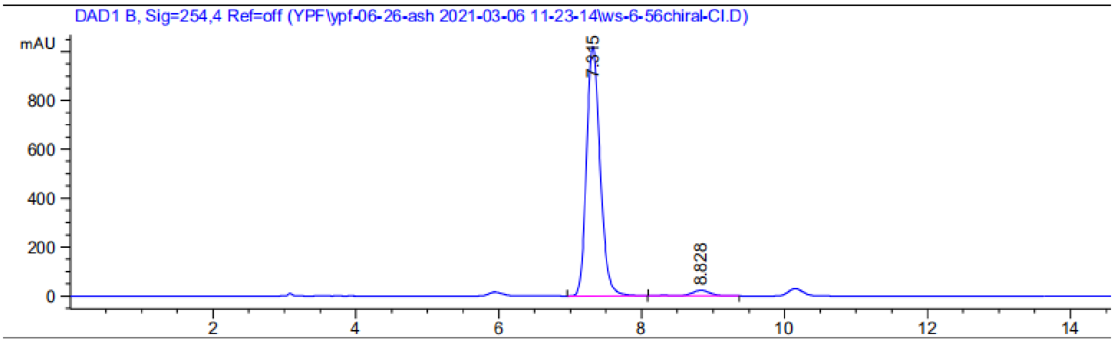

Signal 1: DAD1 B, Sig=254,4 Ref=off

Peak #	RetTime [min]	Type	Width [min]	Area [mAU*s]	Height [mAU]	Area %
1	5.973	MM	0.2102	2.98594e4	2367.85449	95.5084
2	6.685	MM	0.2719	1404.23669	86.09063	4.4916

Supplementary Figure 221 HPLC spectra for **6n**

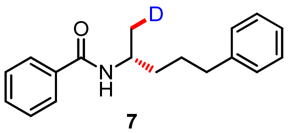

7

HPLC data using *rac*-**L41**

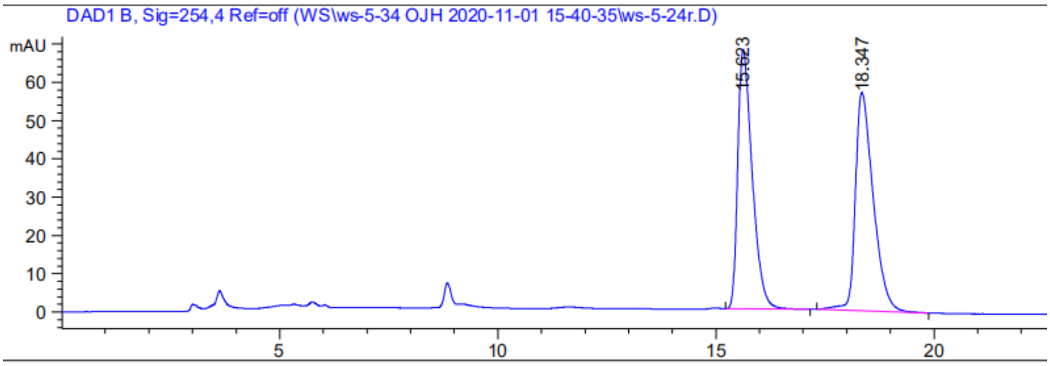

Signal 1: DAD1 B, Sig=254,4 Ref=off

Peak #	RetTime [min]	Type	Width [min]	Area [mAU*s]	Height [mAU]	Area %
1	15.623	BB	0.3515	1564.46973	67.71703	49.0091
2	18.347	BB	0.4274	1627.73132	56.95971	50.9909

HPLC data using (1*S*, 2*R*)-**L41**

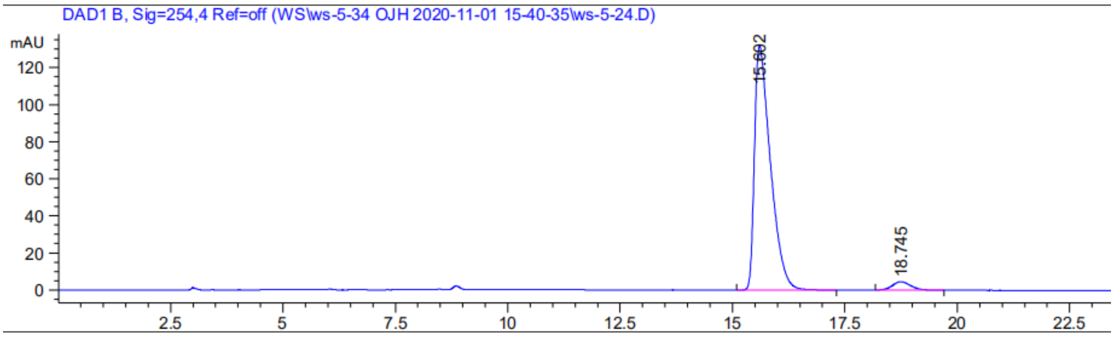

Signal 1: DAD1 B, Sig=254,4 Ref=off

Peak #	RetTime [min]	Type	Width [min]	Area [mAU*s]	Height [mAU]	Area %
1	15.602	BB	0.3704	3254.71924	131.68410	96.3095
2	18.745	BB	0.4193	124.71962	4.47305	3.6905

Supplementary Figure 222 HPLC spectra for **7**

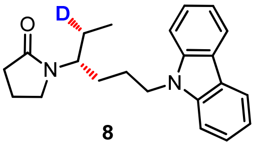

8
from (*Z*) acyl enamine

HPLC data using *rac*-**L4**

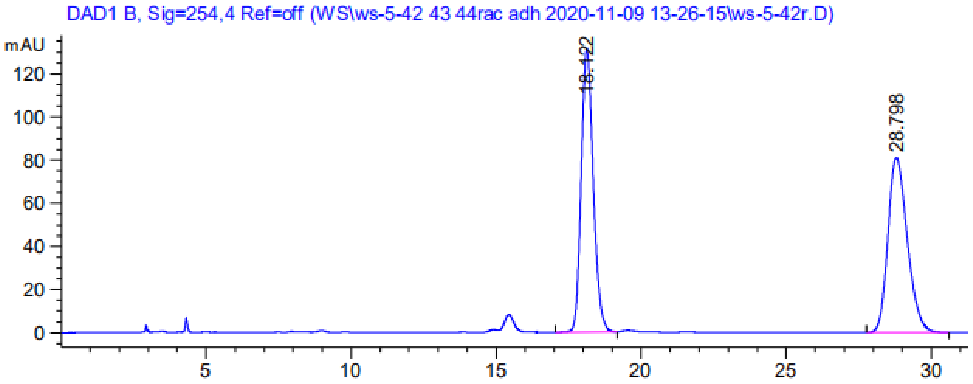

Signal 1: DAD1 B, Sig=254,4 Ref=off

Peak #	RetTime [min]	Type	Width [min]	Area [mAU*s]	Height [mAU]	Area %
1	18.122	BB	0.4444	3780.10547	131.09605	50.1377
2	28.798	BB	0.7135	3759.33765	80.99487	49.8623

HPLC data using (1*S*, 2*R*)-**L41**

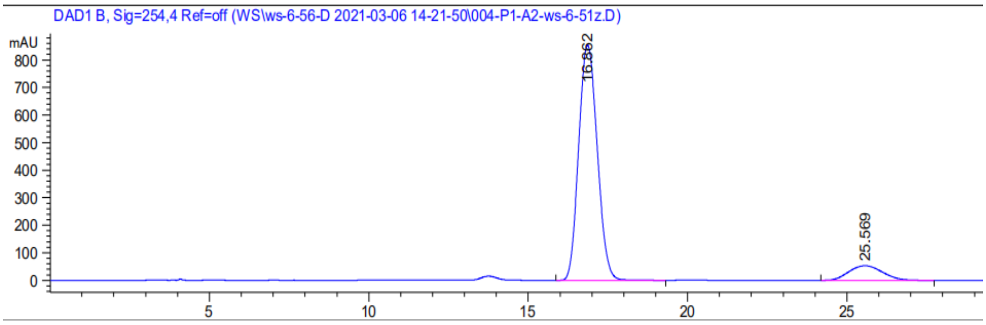

Signal 1: DAD1 B, Sig=254,4 Ref=off

Peak #	RetTime [min]	Type	Width [min]	Area [mAU*s]	Height [mAU]	Area %
1	16.862	BB	0.6448	3.46129e4	854.17151	89.9257
2	25.569	BB	1.1302	3877.67651	52.95941	10.0743

Supplementary Figure 223 HPLC spectra for **8**

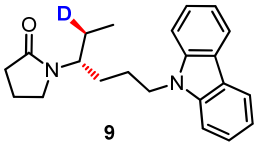

9
from (*E*) acyl enamine

HPLC data using *rac*-**L4**

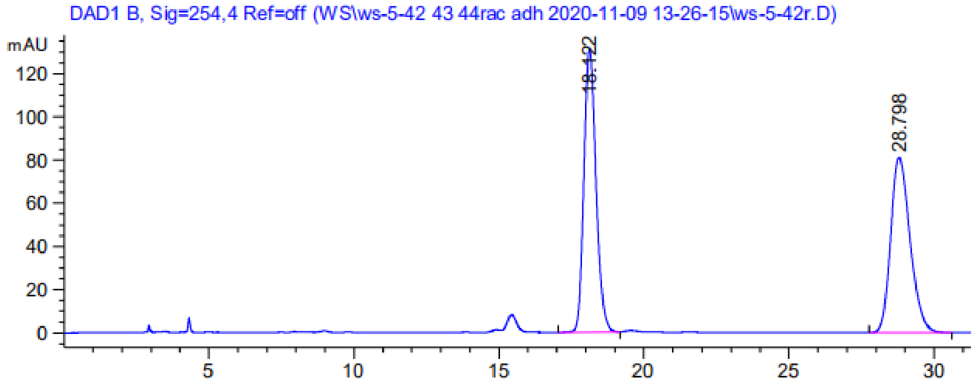

Signal 1: DAD1 B, Sig=254,4 Ref=off

Peak #	RetTime [min]	Type	Width [min]	Area [mAU*s]	Height [mAU]	Area %
1	18.122	BB	0.4444	3780.10547	131.09605	50.1377
2	28.798	BB	0.7135	3759.33765	80.99487	49.8623

HPLC data using (1*S*, 2*R*)-**L41**

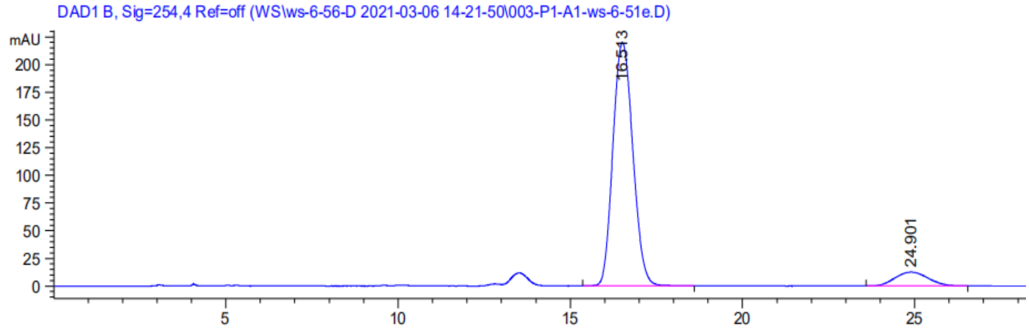

Signal 1: DAD1 B, Sig=254,4 Ref=off

Peak #	RetTime [min]	Type	Width [min]	Area [mAU*s]	Height [mAU]	Area %
1	16.513	BB	0.6372	8842.15039	219.86369	91.4088
2	24.901	BB	0.8156	831.04749	12.46898	8.5912

Supplementary Figure 224 HPLC spectra for **9**

Supplementary References

1. Tu, S. & Zhang, C. Facile Preparation of *N*-Vinylisobutyramide and *N*-Vinyl-2-pyrrolidinone. *Org. Process Res. Dev.* **19**, 2045-2049 (2015).

2. Zhang, G.-Y., Zhou, S., Fu, L., Chen, P.-H., Li, Y.-B., Zou, J.-P. & Liu, G.-S. Asymmetric Coupling of Carbon-Centered Radicals Adjacent to Nitrogen: Copper-Catalyzed Cyanation and Etherification of Enamides. *Angew. Chem. Int. Ed*. **59**, 20439-20444 (2020).

3. Dehimat, Z. I., Pasahan, A., Tebbani, D., Yasar, S. & Ozdemir, I. Synthesis of Sterically Hindered *N*-Benzyladamantyl Substituted Benzimidazol-2-ylidene Palladium Complexes and Investigation of Their Catalytic Activity in Aqueous Medium. *Tetrahedron* **73**, 5940-5945 (2017).

4. Mou, X.-Q., Rong, F.-M., Zhang, H., Chen, G. & He, G. Copper(I)-Catalyzed Enantioselective Intramolecular Aminotrifluoromethylation of *O*-Homoallyl Benzimidates. *Org. Lett.* **21**, 4657-4661 (2019).

5. Shi, L., Xing, L.-L., Hu, W.-B. & Shu, W. Regio- and Enantioselective Ni-Catalyzed Formal Hydroalkylation, Hydrobenzylation, and Hydropropargylation of Acrylamides to a Tertiary Amides. *Angew. Chem. Int. Ed.* **60**, 1599-1604 (2021).

6. Yi, Y., Gholami, H., Morrow, M. G. & Borhan, B. XtalFluor-E® Mediated Proto-Functionalization of N-Vinyl Amides: Access to N-Acetyl N,O-Acetals. *Org. Biomol. Chem*. **15**, 9570-9574 (2017).

7. Goossen, L. J., Paetzold, J. & Koley, D. Regiocontrolled Ru-Catalyzed Addition of Carboxylic Acids to Alkynes: Practical Protocols for the Synthesis of Vinyl Esters. *Chem. Commun*. 706-707 (2003).
